# The *Staphylococcus aureus* Network Adaptive Platform Trial Protocol: New Tools for an Old Foe

**DOI:** 10.1093/cid/ciac476

**Published:** 2022-06-19

**Authors:** Steven Y C Tong, Jocelyn Mora, Asha C Bowen, Matthew P Cheng, Nick Daneman, Anna L Goodman, George S Heriot, Todd C Lee, Roger J Lewis, David C Lye, Robert K Mahar, Julie Marsh, Anna McGlothlin, Zoe McQuilten, Susan C Morpeth, David L Paterson, David J Price, Jason A Roberts, J Owen Robinson, Sebastiaan J van Hal, Genevieve Walls, Steve A Webb, Lyn Whiteway, Dafna Yahav, Joshua S Davis, for the Staphylococcus aureus Network Adaptive Platform (SNAP) Study Group, Nick Anagnostou, Sophia Archuleta, Eugene Athan, Lauren Barina, Emma Best, Max Bloomfield, Jennifer Bostock, Carly Botheras, Asha Bowen, Philip Britton, Hannah Burden, Anita Campbell, Hannah Carter, Matthew Cheng, Ka Lip Chew, Russel Lee Ming Chong, Geoff Coombs, Peter Daley, Nick Daneman, Jane Davies, Joshua Davis, Yael Dishon, Ravindra Dotel, Adrian Dunlop, Felicity Flack, Katie Flanagan, Hong Foo, Nesrin Ghanem-Zoubi, Stefano Giulieri, Anna Goodman, Jennifer Grant, Dan Gregson, Stephen Guy, Amanda Gwee, Erica Hardy, Andrew Henderson, George Heriot, Benjamin Howden, Fleur Hudson, Jennie Johnstone, Shirin Kalimuddin, Dana de Kretser, Andrea Kwa, Todd Lee, Amy Legg, Roger Lewis, Martin Llewelyn, Thomas Lumley, David Lye, Derek MacFadden, Robert Mahar, Isabelle Malhamé, Michael Marks, Julie Marsh, Marianne Martinello, Gail Matthews, Colin McArthur, Anna McGlothlin, Genevieve McKew, Brendan McMullan, Zoe McQuilten, Eliza Milliken, Jocelyn Mora, Susan Morpeth, Srinivas Murthy, Clare Nourse, Matthew O'Sullivan, David Paterson, Mical Paul, Neta Petersiel, Lina Petrella, Sarah Pett, David Price, Jason Roberts, Owen Robinson, Ben Rogers, Benjamin Saville, Matthew Scarborough, Marc Scheetz, Oded Scheuerman, Kevin Schwartz, Simon Smith, Tom Snelling, Marta Soares, Christine Sommerville, Andrew Stewardson, Neil Stone, Archana Sud, Robert Tilley, Steven Tong, Rebecca Turner, Jonathan Underwood, Sebastiaan van Hal, Lesley Voss, Genevieve Walls, Rachel Webb, Steve Webb, Lynda Whiteway, Heather Wilson, Terry Wuerz, Dafna Yahav

**Affiliations:** Department of Infectious Diseases University of Melbourne, Peter Doherty Institute for Infection and Immunity, Melbourne, Australia; Department of Infectious Diseases University of Melbourne, Peter Doherty Institute for Infection and Immunity, Melbourne, Australia; Department of Infectious Diseases, Perth Children’s Hospital, Perth, Australia; Wesfarmers Centre for Vaccines and Infectious Diseases, Telethon Kids Institute, University of Western Australia, Perth, Australia; Divisions of Infectious Diseases and Medical Microbiology, McGill University Health Centre, Montreal, Canada; Division of Infectious Diseases, Department of Medicine, Sunnybrook Health Sciences Centre, University of Toronto, Toronto, Canada; Medical Research Council Clinical Trials Unit, University College London, London, United Kingdom; Department of Infection, St Thomas Hospital, Guy’s and St Thomas NHS Foundation Trust, London, United Kingdom; Department of Infectious Diseases University of Melbourne, Peter Doherty Institute for Infection and Immunity, Melbourne, Australia; Clinical Practice Assessment Unit and Division of Infectious Diseases, McGill University, Montreal, Canada; Berry Consultants, LLC, Austin, Texas, USA; Department of Emergency Medicine, Harbor-UCLA Medical Center, Torrance, California, USA; Department of Emergency Medicine, David Geffen School of Medicine at UCLA, Los Angeles, California, USA; National Centre for Infectious Diseases, Singapore; Department of Infectious Diseases, Tan Tock Seng Hospital, Singapore; Yong Loo Lin School of Medicine, Singapore; Lee Kong Chian School of Medicine, Singapore; Centre for Epidemiology and Biostatistics, Melbourne School of Population and Global Health, University of Melbourne, Parkville, Australia; Clinical Epidemiology and Biostatistics Unit, Murdoch Children’s Research Institute, Parkville, Australia; Telethon Kids Institute, Perth Children’s Hospital, Perth, Australia; Berry Consultants, LLC, Austin, Texas, USA; Department of Epidemiology and Preventive Medicine, Monash University, Melbourne, Australia; Department of Haematology, Monash Health, Melbourne, Australia; Department of Infectious Diseases, Middlemore Hospital, Auckland, New Zealand; University of Queensland Centre for Clinical Research, Royal Brisbane and Women’s Hospital Campus, Brisbane, Australia; Department of Infectious Diseases University of Melbourne, Peter Doherty Institute for Infection and Immunity, Melbourne, Australia; Centre for Epidemiology and Biostatistics, Melbourne School of Population and Global Health, University of Melbourne, Parkville, Australia; University of Queensland Centre for Clinical Research, Faculty of Medicine, University of Queensland, Brisbane, Australia; Departments of Pharmacy and Intensive Care Medicine, Royal Brisbane and Women’s Hospital, Brisbane, Australia; Department of Infectious Diseases, Royal Perth Hospital, Perth, Australia; Department of Infectious Diseases, Fiona Stanley Hospital, Murdoch, Australia; PathWest Laboratory Medicine, Perth, Australia; College of Science, Health, Engineering and Education, Murdoch University, Murdoch, Australia; Department of Microbiology and Infectious Diseases Royal Prince Alfred Hospital, Sydney, Australia; School of Medicine, University of Sydney, Sydney, Australia; Department of Infectious Diseases, Middlemore Hospital, Auckland, New Zealand; Australian and New Zealand Intensive Care Research Centre, Monash University, Melbourne, Australia; Freelance Health Consumer Advocate, Adealide, South Australia, Australia; Infectious Diseases Unit, Rabin Medical Center, Beilinson Hospital, Petah-Tikva, Israel; School of Medicine and Public Health and Hunter Medical Research Institute, University of Newcastle, Newcastle, Australia

**Keywords:** *Staphylococcus aureus*, bacteremia, bloodstream infection, randomized controlled trial, adaptive platform

## Abstract

*Staphylococcus aureus* bloodstream (SAB) infection is a common and severe infectious disease, with a 90-day mortality of 15%–30%. Despite this, <3000 people have been randomized into clinical trials of treatments for SAB infection. The limited evidence base partly results from clinical trials for SAB infections being difficult to complete at scale using traditional clinical trial methods. Here we provide the rationale and framework for an adaptive platform trial applied to SAB infections. We detail the design features of the *Staphylococcus aureus* Network Adaptive Platform (SNAP) trial that will enable multiple questions to be answered as efficiently as possible. The SNAP trial commenced enrolling patients across multiple countries in 2022 with an estimated target sample size of 7000 participants. This approach may serve as an exemplar to increase efficiency of clinical trials for other infectious disease syndromes.

## Teaser

The *Staphylococcus aureus* Network Adaptive Platform (SNAP) trial is a multicenter international adaptive platform trial that will simultaneously address multiple key questions in the management of *S. aureus* bloodstream infections and involve >7000 adult and child participants.

## *STAPHYLOCOCCUS AUREUS* BLOODSTREAM INFECTION—THE SCOPE OF THE PROBLEM

*Staphylococcus aureus* bloodstream infections (SABs) are common worldwide and considered among the “bread and butter” of clinical infectious diseases practice. More than 120 000 episodes occur per year in the United States [1], with a 30-day mortality per episode of 20%–30% in high-income countries [2]. Almost all patients with SAB require hospitalization, typically receive a minimum of 2 weeks of intravenous antibiotics, and remain in hospital an average of 20 days [3]. For such a common condition, a surprising number of basic therapeutic questions remain unanswered. Among others, these include: Is an anti-staphylococcal penicillin or cefazolin preferred for methicillin-susceptible SAB? Can combination therapy for methicillin-resistant SAB improve therapeutic efficacy without significant increases in toxicity? Is switching to oral antibiotics as safe and efficacious as continued intravenous therapy?

### Traditional Fixed Randomized Trial Designs Are Blunt Tools

Clinical trials for SAB are challenging [4] and expensive. Despite the burden of disease, <3000 participants have been enrolled in completed randomized clinical trials (n = 15) for SAB from 2000 to 2021 [4]. The sample size of these trials ranges from 15 to 758. Challenges include the heterogeneity of the disease, variability in therapeutic and diagnostic approaches, and difficulties in recruitment. The limited evidence results in considerable variability in clinical practice [5, 6]. Traditional trial designs are based on achieving a sample size calculated a priori according to a series of assumptions, which often prove to be inaccurate or are manipulated to align with available funding, so underpowered trials may be completed without providing definitive answers.

### Adaptive Platform Trials—Cutting Through Uncertainty to Answer Questions Efficiently

Recently, trials making use of disease-based platforms and within-trial adaptations (ie, platform trials) have gained prominence [7]. These trials can answer multiple questions simultaneously and adapt to make the most efficient use of a given budget and sample size. The RECOVERY (Randomised Evaluation of COVID-19 Therapy), REMAP-CAP (Randomised Embedded Multifactorial Adaptive Platform-Community Acquired Pneumonia), and the National Institutes of Health–funded ACTIV (Acceperating COVID-19 Therapeutic Interventions and Vaccines) and ACTT (Adaptive COVID-19 Treatment Trial) trials are examples of platform trials that have provided clinically relevant answers to multiple therapeutic questions for coronavirus disease 2019 [8–11].

## SUMMARY OF *STAPHYLOCOCCUS AUREUS* NETWORK ADAPTIVE PLATFORM (SNAP) TRIAL PROTOCOL

### Design Features of the SNAP Trial to Address Existing Challenges

Here we outline the key features of a currently active adaptive platform trial for *S. aureus* bloodstream infections: the *Staphylococcus aureus* Network Adaptive Platform (SNAP) trial. The full trial protocol and relevant appendices are provided as Supplementary Materials.

**Figure 1.**
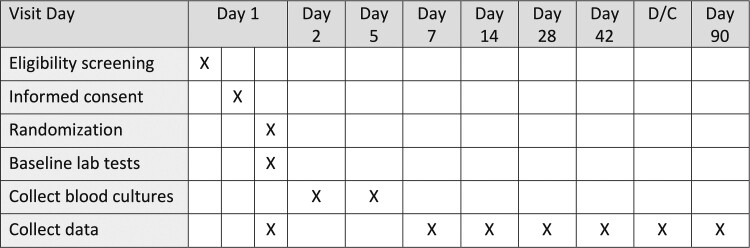
Trial procedures and schedules. Abbreviation: D/C, discharge.

Several critical design features of SNAP contribute to enhanced trial efficiency. First, the trial is highly pragmatic. The inclusion criteria are easily identified, and exclusion criteria are minimal, so most patients with SAB will be eligible (Table 1). Recruitment uses a simplified and tiered consent process developed in conjunction with healthcare consumers with experience of the disease (see Supplementary Appendix and the SNAP website, https://www.snaptrial.com.au/). All initial interventions reflect current standard care. Most interventions will be open label, as the advantages of blinding and use of placebo have been judged to be offset by increased cost and complexity when considering multiple parallel interventions. Wherever possible, routinely collected clinical and administrative data are used (Figure 1). This approach facilitates low operational complexity at the bedside, even though the internal clinical trial machinery is complex.

**Table 1.** Inclusion and Exclusion Criteria

Inclusion Criteria	Exclusion Criteria
1. *Staphylococcus aureus* complex grown from ≥1 blood culture	1. Time of anticipated platform entry is >72 hours after collection of the index blood culture
2. Admitted to participating hospital at anticipated time of eligibility assessment	2. Polymicrobial bacteremia
	3. Patient currently being treated with a systemic antibacterial agent that cannot be ceased
	4. Known previous participation in SNAP
	5. Known positive blood culture for *S. aureus* (of the same silo: PSSA, MSSA, or MRSA) between 72 hours and 180 days prior to the time of eligibility assessment
	6. Treating team deems that enrollment in the study is not in the best interest of the patient
	7. Treating clinician believes that death is imminent and inevitable
	8. Patient is for end-of-life care and antibiotic treatment is considered not appropriate
	9. Patient <18 years of age and pediatric recruitment not approved at recruiting site
	10. Patient has died

Abbreviations: MRSA, methicillin-resistant *Staphylococcus aureus*; MSSA, penicillin-resistant, methicillin-susceptible *Staphylococcus aureus*; PSSA, penicillin-susceptible *Staphylococcus aureus*; *S. aureus*, *Staphylococcus aureus*; SNAP, *Staphylococcus aureus* Network Adaptive Platform.

**Figure 2.**
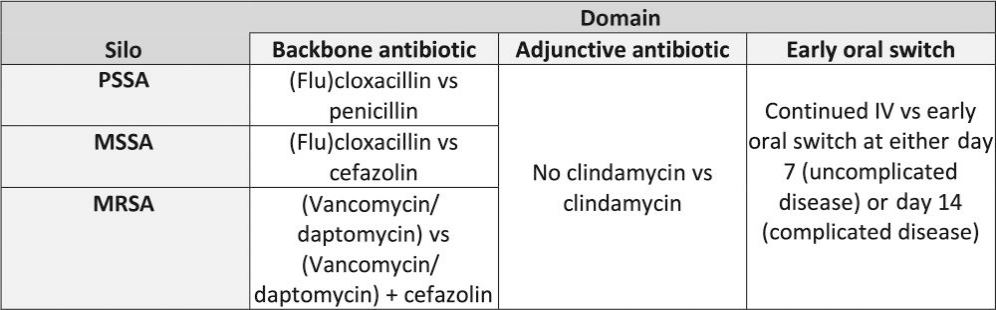
Initial trial design. In each cell, the treatment that is often considered the usual standard of care is listed first and the “intervention” is listed second. Abbreviations: IV, intravenous; MRSA, methicillin-resistant *Staphylococcus aureus*; MSSA, penicillin-resistant, methicillin-susceptible *Staphylococcus aureus*; PSSA, penicillin-susceptible *Staphylococcus aureus*.

Second, SNAP will implement a core (master) protocol together with a flexible modular domain structure (Figure 2), where a “domain” denotes a group of interventions with comparable modes of action. Each patient may be randomized within 1 or more domains. In the core protocol, we prespecify mutually exclusive subgroups (“silos”) according to the antibiotic susceptibility of the *S. aureus* isolate (penicillin-susceptible *S. aureus* [PSSA], penicillin-resistant, methicillin-susceptible *S. aureus* [MSSA], and methicillin-resistant *S. aureus* [MRSA]). The same core primary (90-day mortality) and secondary endpoints apply for all silos and domains (Table 2). Each domain is detailed in separate domain-specific appendices (Supplementary Materials) that each function as trial protocols nested under the core protocol. Domains can be added or removed during the life of the platform. The flexibility extends to whether regions, trial sites, and individual participants choose to participate in each domain. By addressing multiple questions in parallel, the platform can reduce the time, cost and sample size required to reach definitive conclusions compared to sequentially executed, traditionally designed trials.

**Table 2.** Core Primary and Secondary Outcome Measures

Outcome	Outcome Measure
Primary	All-cause mortality 90 days after platform entry.
Secondary	1. All-cause mortality at 14, 28, and 42 days
	2. Duration of survival censored at 90 days
	3. Length of stay of acute index inpatient hospitalization for those surviving until hospital discharge (excluding HITH/COPAT/OPAT/rehab)
	4. Length of stay of total index hospitalization for those surviving until hospital discharge (including HITH/COPAT/OPAT/rehab)
	5. Time to being discharged alive from the total index hospitalization (including HITH/COPAT/OPAT/rehab)
	6. Microbiological treatment failure (positive sterile site culture for *Staphylococcus aureus* (of the same silo as the index isolate) between 14 and 90 days after platform entry)
	7. Diagnosis of new foci between 14 and 90 days after platform entry
	8. *Clostridioides difficile* diarrhea as determined by a clinical laboratory in the 90 days following platform entry for participants ≥2 years of age
	9. Serious adverse reactions in the 90 days following platform entry
	10. Health economic costs
	11. Proportion of participants who have returned to their usual level of function at day 90 as determined by the modified functional bloodstream infection score
	12. Desirability of outcome ranking 1 (modified ARLG version) at 90 days
	13. Desirability of outcome ranking 2 (SNAP version) at 90 days

Abbreviations: ARLG, Antibiotic Resistance Leadership Group; COPAT, Complex Out Patient Antibiotic Therapy; HITH, Hospital in the Home; OPAT, Outpatient Parenteral Antibiotic Therapy; SNAP, *Staphylococcus aureus* Network Adaptive Platform.

Third, frequent planned interim analyses (Bayesian updates) will be performed on the accumulating data and prespecified decision criteria for noninferiority, superiority, or futility will be evaluated. Questions are concluded as soon as stringent probability thresholds are met. Extensive pretrial simulations have been conducted, under a range of plausible trial scenarios, to inform the trial design and ensure an acceptable level of type I error (false positive) for each domain and across the entire platform. The primary analysis is structured to accommodate trial adaptations, such as the inclusion or removal of domains or interventions within domains. In addition to the covariate of age, sensitivity analyses will include model-based time trend adjustment in the anticipation that *S. aureus* genotypes or outcomes may vary over time [12].

Fourth, the platform has global scope and unprecedented sample size. The platform will initially operate in Australia, Singapore, Canada, Israel, New Zealand, and the United Kingdom, with an estimated 100 hospital trial sites. Further countries and sites may join. We plan to enroll at least 7000 patients, an order of magnitude larger than any previous pathogen-specific bloodstream infection trial [13]. The availability and low cost of currently included interventions are well suited to involvement of low- and middle-income countries. Regional appendices to the protocol detail region-specific regulatory, insurance, reimbursement, and drug availability aspects.

Fifth, we will prospectively include children and adults (including pregnant women). Pediatric data will be used to estimate the effects of interventions in children using a hierarchical statistical model that also borrows information from the adult cohort. This model for generating evidence for pediatrics is a significant advance over extrapolating evidence from adults to pediatric populations in the absence of pediatric trial data [14].

### Primary Outcome

The trial primary outcome is all-cause mortality 90 days after enrollment. We considered this as most relevant to patients and clinicians and most likely to influence practice. Other outcomes that may be more proximate to the trial interventions such as treatment failure, microbiological relapse, and serious adverse reactions are captured as secondary outcomes.

### Trial Structure—Domains and Silos

Within the silos and initial domains (Figure 2) the SNAP trial will address the following questions that are considered clinical priorities and for which there is clinician equipoise [6]:

#### In the Backbone Antibiotic Domain:

1. For PSSA, is benzylpenicillin noninferior to (flu)cloxacillin?
2. For MSSA, is cefazolin noninferior to (flu)cloxacillin?
3. For MRSA, is the combination of usual care (vancomycin or daptomycin) plus cefazolin for 7 days superior to usual care alone?

Although not all laboratories routinely test for or report penicillin susceptibility, those that do find it in up to 20% of *S. aureus* bloodstream isolates [15]. Retrospective observational data suggest that benzylpenicillin is as effective as anti-staphylococcal penicillins for PSSA and has the theoretical benefits of less protein binding (thus higher free concentrations), lower minimum inhibitory concentrations, and fewer adverse effects [16]. Demonstrating noninferiority of benzylpenicillin would likely change practice.

MSSA accounts for most *S. aureus* bloodstream isolates. Historically, anti-staphylococcal penicillins like (flu)cloxacillin or nafcillin have been preferred to cefazolin due to concerns regarding cefazolin stability in the presence of high levels of penicillinase, an in vitro phenomenon termed the inoculum effect [17]. However, retrospective observational data suggest that cefazolin may be superior to anti-staphylococcal penicillins [18, 19]. Simpler dosing regimens and likely fewer adverse effects mean that demonstrating noninferiority (or superiority) of cefazolin to anti-staphylococcal penicillins would change practice.

MRSA continues to be difficult to treat with limitations to the current internationally accepted standard of care of vancomycin. However, no clinical trials have convincingly demonstrated improved outcomes with other drugs or with combination therapy. Combining cefazolin with vancomycin or daptomycin may lead to more rapid clearance of bacteremia without the toxicity seen when combining vancomycin with anti-staphylococcal penicillins [20]. Demonstration of superiority of combination therapy would change clinical practice.

### In the Adjunctive Treatment Domain: Is the Addition of Clindamycin for 5 Days to Usual Care Superior to Usual Care Alone?

Adjunctive treatments for SAB have not been shown to be of clinical benefit to date [13, 21]. However, several guidelines recommend antitoxin adjunctive therapies such as clindamycin or linezolid for more severe staphylococcal disease syndromes [22, 23]. Early use of such an anti-toxin antibiotic when the organism burden is high may improve clinical outcomes. Given the potential toxicities, including risk for *Clostridioides difficile* infection, demonstration of superiority of adjunctive clindamycin would be required to change practice.

### In the Early Oral Switch Domain: For Patients Who Are Clinically Stable at Day 7 or Day 14 Following Platform Entry, Is Switching to an Oral Antibiotic Regimen Noninferior to Continued Intravenous Antibiotic Therapy?

The POET (Partial Oral Endocarditis Treatment) and OVIVA (Oral Versus Intravenous Antibiotics) trials have demonstrated the effectiveness and safety of switching to oral antibiotic regimens for carefully selected patients with serious infections such as infective endocarditis and osteoarticular infections [24, 25]. However, the number of patients in these trials with SAB was limited. Inclusion criteria for entry to this domain includes clinically stable disease (no longer bacteremic, adequate source control) and the ability to absorb or adhere to oral regimens. Those eligible at day 7 are participants typically defined as having uncomplicated disease, while those eligible at day 14 can include participants with complicated disease [26]. Demonstration of noninferiority of early oral switch for SAB would be a significant advance for the field. In particular, SNAP will include patients with complicated disease, a higher-risk population that has not been included in similar trials to date [27, 28].

### Randomization, Patient Allocation, and Blinding

**Figure 3.**
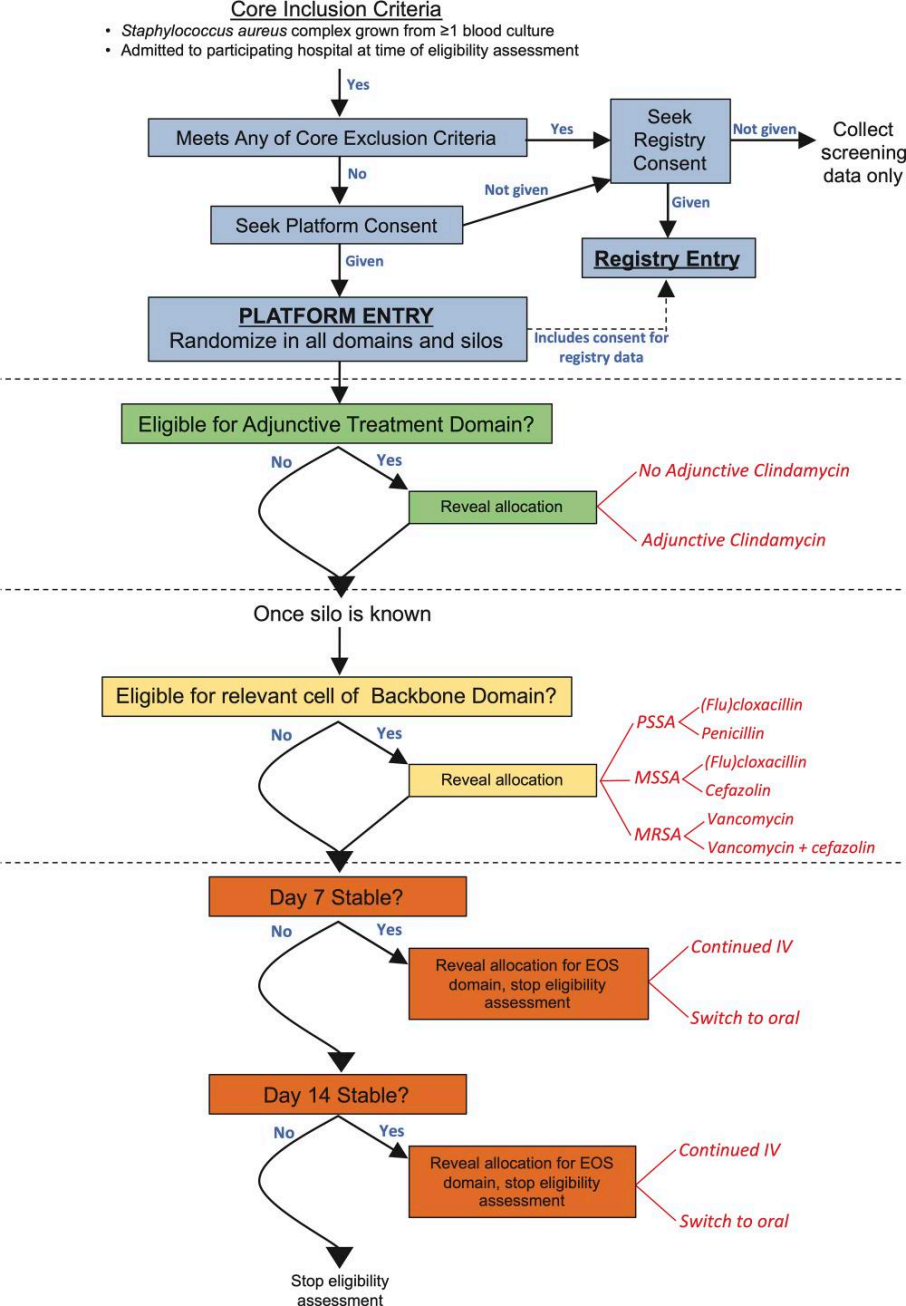
Participant flow, randomization, and reveal of allocation timings. Abbreviations: EOS, early oral switch; IV, intravenous; MRSA, methicillin-resistant *Staphylococcus aureus*; MSSA, penicillin-resistant, methicillin-susceptible *Staphylococcus aureus*; PSSA, penicillin-susceptible *Staphylococcus aureus.*

Participants will be randomly assigned to 1 arm within each domain for which they are eligible (and which their site is participating in) using a web-based module. Randomization in all possible silos and available domains will occur immediately following consent (which is considered the time of platform entry); however, the reveal of each treatment assignment(s) will be delayed subject to confirmation of eligibility, including availability of relevant microbiology. This design allows for flexibility in the timing of assignments being revealed (Figure 3). For example, the reveal for the adjunctive antibiotic domain can occur immediately following identification of *S. aureus* in blood cultures and participant consent. The silo (PSSA, MSSA, or MRSA) may not be known for a further 24–48 hours and thus reveal of assignment to the relevant backbone antibiotic will follow.

### Statistical Principles and Framework

There are 2 key elements in the statistical analysis plans: (1) repeated analyses of the core primary endpoint over the trial lifetime to evaluate prespecified decision criteria for stopping or continuing recruitment to a domain; and (2) analysis and reporting of core and domain-specific secondary endpoints once a decision threshold (superiority, noninferiority, or futility) is reached for a domain. The predefined decision criteria for a domain are:

1. Noninferiority—if the posterior probability of noninferiority of the investigational agent vs the standard of care is >99%; where noninferiority is defined as an odds ratio (OR) of <1.2 for the primary endpoint (where OR >1 indicates an increase in mortality). An OR of 1.2 corresponds to an absolute difference of 2.5% between intervention and standard care arms if the mortality rate in the standard care arm is 15%.
2. Superiority—if the posterior probability of superiority of the investigational agent vs the standard of care is >99%, where superiority is defined as an OR of <1.0 for the primary endpoint.
3. Futility for noninferiority—if the posterior probability of noninferiority is <1% within the maximal sample size of 7000.
4. Futility for superiority—if the posterior probability for superiority is <1% within the maximal sample size of 7000.

Where specified within a domain-specific appendix, agents that achieve noninferiority thresholds may be subsequently tested for superiority thresholds without a public declaration of the noninferiority result. For example, if cefazolin achieved the threshold for noninferiority to (flu)cloxacillin in the MSSA silo and backbone antibiotic domain, and the analysis indicated it would not be futile to assess for superiority, participants would continue to be randomized to cefazolin or (flu)cloxacillin.

The first platform Bayesian update will be performed after 500 eligible platform participants have completed 90 days of follow-up (“completers”); thereafter, updates will be performed with every 500 additional completers until the trial is concluded. A detailed description of the statistical design and principles and the trial simulations will be published separately.

Adaptive platform trials do not require a fixed sample size. Ideally, a platform can continue perpetually as long as clinical questions of public health significance remain. However, pretrial simulations incorporating a maximal anticipated sample size can indicate the probability of false-positive conclusions (the type I error) and the probability of reaching appropriate decision thresholds (equivalent to the power of the study). We simulated various scenarios, each with 1000 simulated trials, and a maximal sample size of 7000 (approximately 6000 adults and 1000 children). Examples are provided in Table 3 of scenarios where each intervention group has no effect (OR, 1.0; scenario 1) or a moderate effect size (OR, 0.75; scenario 2) and the decision thresholds are as previously stated. These simulations indicate that the probability of a type I error is <7% across all silos and domains (specifically see scenario 1, column for superiority and silos A and B), and the platform is adequately powered to declare noninferiority and/or superiority for moderate effect sizes (specifically see scenario 2, columns for noninferiority and superiority). For example, if there is no difference in effect of clindamycin over usual care (scenario 1) in the adjunctive treatment domain, the probability of declaring superiority (ie, a false-positive or type I error) is 6% (Table 3, scenario 1, superiority column B). However, if clindamycin confers an OR of 0.75 (scenario 2), the probability of declaring superiority (ie, power) is 92% (Table 3, scenario 2 superiority column B).

**Table 3.** Proportion of Simulated Trials Declaring Noninferiority, Superiority, or Futility for Each Domain^a^

Scenario	Silo	Noninferiority			Superiority			Futility for Noninferiority			Futility for Superiority		
		A	B	C	A	B	C	A	B	C	A	B	C
1	PSSA	0.22	NA	0.22	0.05	0.06	NA	0.00	NA	0.00	0.00	0.69	NA
	MSSA	0.46	NA	0.47	0.06	0.06	NA	0.01	NA	0.00	0.07	0.69	NA
	MRSA	NA	NA	0.26	0.06	0.06	NA	NA	NA	0.00	0.28	0.69	NA
2	PSSA	0.61	NA	0.78	0.30	0.92	NA	0.00	NA	0.00	0.00	0.01	NA
	MSSA	0.99	NA	0.95	0.76	0.92	NA	0.00	NA	0.00	0.00	0.01	NA
	MRSA	NA	NA	0.84	0.40	0.92	NA	NA	NA	0.00	0.03	0.01	NA

Assumptions for these simulations: Proportional representation of each silo: PSSA (16%), MSSA (64%), MRSA (20%). Baseline mortality rates for adults and children in each silo: PSSA (15% and 2.2%, respectively), MSSA (15% and 2.2%), MRSA (20% and 3.5%). The proportion of participants eligible for the early oral switch domain at day 7 (10%) and day 14 (45%). Scenario 1: No treatment effect for any domains (odds ratio [OR], 1.0). Scenario 2: OR for mortality of 0.75 for the interventions in each domain. For domain B, results from silos are pooled. For domain C, results for each silo are modeled with hierarchical Bayesian borrowing across the silos.

Abbreviations: MRSA, methicillin-resistant *Staphylococcus aureus*; MSSA, penicillin-resistant, methicillin-susceptible *Staphylococcus aureus*; NA, not applicable; PSSA, penicillin-susceptible *Staphylococcus aureus*.

^a^Domain A: Antibiotic backbone (PSSA: [flu]cloxacillin vs penicillin [assessing noninferiority of penicillin]; MSSA: [flu]cloxacillin vs cefazolin [assessing noninferiority of cefazolin]; MRSA: vancomycin or daptomycin vs (vancomycin or daptomycin) plus cefazolin [assessing superiority of combination]. Domain B: Adjunctive treatment domain (no clindamycin vs clindamycin [assessing superiority of clindamycin]). Domain C: Early oral switch domain (continued intravenous therapy vs oral switch [assessing noninferiority of oral switch]).

### Safety Monitoring and Reporting

The SNAP trial operates under International Conference on Harmonization Good Clinical Practice guidelines, as a comparative effectiveness trial. All included agents have established safety profiles and are registered with regulatory agencies for use in treatment of *S. aureus* infections. We have therefore taken a risk-based approach of targeted safety reporting. Only serious adverse events considered to be related to 1 of the randomized trial agents or strategies will be reported. Anticipated common adverse reactions such as acute kidney or liver injury and *C. difficile* diarrhea will be collected as prespecified secondary endpoints.

Central monitoring with source data verification of critical data points will occur for all patients. All serious adverse reactions will be assessed by a central safety team. If future domains include novel agents with limited existing safety data, the relevant domain-specific appendices (protocols) will specify additional safety data collection and reporting.

### Trial Oversight, Governance, and Funding

The Data and Safety Monitoring Committee (DSMC) will review the efficacy results from each Bayesian update and regular safety reports. Unless the trajectory of the trial unfolds in unexpected directions, the major role of the DSMC is anticipated to be ensuring that the trial follows the predefined adaptations. To maintain blinding of investigators, firewalls ensure that only named members of the trial analytic team and the DSMC have access to unblinded efficacy reports prior to the public disclosure of domain results.

The overall governance structure is described in the full protocol. In short, there are multiple working groups and committees reporting to a Global Trial Steering Committee. Regional committees and sponsors assume responsibility for trial conduct in each region. Proposals for new clinical questions can be presented to the Global Trial Steering Committee for consideration. Proposals are assessed for clinical priority, feasibility, funding, capacity within the existing framework, and interactions with existing trial domains.

The SNAP trial has currently secured funding from several national health research funding bodies: the Australian National Health and Medical Research Council, the Canadian Institutes of Health Research, the New Zealand Health Research Council, and the United Kingdom National Institute for Health Research.

## CHALLENGES

The challenges of conducting SNAP are similar to that of other pragmatic trials [29]. Data collection is simplified and limited to key data points relevant to the primary and secondary outcomes and identification of subgroups, and safety reporting is focused on serious adverse reactions rather than all adverse events. The trial therefore uses a quality-by-design approach that prioritizes aspects of reliability of data and results and the safety of patients, while removing extraneous requirements [30]. The open-label design risks some introduction of bias. However, the primary outcome of 90-day all-cause mortality is objective, and the complexity of the trial and multiple parallel interventions makes blinding difficult and expensive. The use and interpretation of Bayesian statistics will be unfamiliar to many readers. The reporting of an increasing number of trials that use Bayesian methods suggest that there is growing acceptability of these methods in the academic and clinical community [9, 31].

## CONCLUSIONS

The SNAP trial represents a paradigm shift in the approach to clinical trials by replacing random care with randomized care for *S. aureus* bloodstream infections. Rather than studying a single question (eg, daptomycin vs vancomycin), the adaptive platform trial approach studies a disease syndrome. Rather than separating adults and children, we are taking a consistent approach across the whole of life. The infrastructure developed can be used to address multiple questions in parallel and incorporate new questions. In taking a pragmatic approach to the interventions being studied, the data collection and safety reporting required, and the informed consent process, we anticipate this being an important step toward embedding such clinical trials within usual healthcare and creating learning healthcare systems. *Staphylococcus aureus* bloodstream infections are both common and deadly, and we hope that the adaptive platform trial approach detailed here can be an exemplar for future investigations of other infectious diseases.

## Supplementary Data

Supplementary materials are available at *Clinical Infectious Diseases* online. Consisting of data provided by the authors to benefit the reader, the posted materials are not copyedited and are the sole responsibility of the authors, so questions or comments should be addressed to the corresponding author.

## Notes

***Staphylococcus aureus Network Adaptive Platform*** (***SNAP) Study Group collaborating members.*** Nick Anagnostou, Sophia Archuleta, Eugene Athan, Lauren Barina, Emma Best, Max Bloomfield, Jennifer Bostock, Carly Botheras, Asha Bowen, Philip Britton, Hannah Burden, Anita Campbell, Hannah Carter, Matthew Cheng, Ka Lip Chew, Russel Lee Ming Chong, Geoff Coombs, Peter Daley, Nick Daneman, Jane Davies, Joshua Davis, Yael Dishon, Ravindra Dotel, Adrian Dunlop, Felicity Flack, Katie Flanagan, Hong Foo, Nesrin Ghanem-Zoubi, Stefano Giulieri, Anna Goodman, Jennifer Grant, Dan Gregson, Stephen Guy, Amanda Gwee, Erica Hardy, Andrew Henderson, George Heriot, Benjamin Howden, Fleur Hudson, Jennie Johnstone, Shirin Kalimuddin, Dana de Kretser, Andrea Kwa, Todd Lee, Amy Legg, Roger Lewis, Martin Llewelyn, Thomas Lumley, David Lye, Derek MacFadden, Robert Mahar, Isabelle Malhamé, Michael Marks, Julie Marsh, Marianne Martinello, Gail Matthews, Colin McArthur, Anna McGlothlin, Genevieve McKew, Brendan McMullan, Zoe McQuilten, Eliza Milliken, Jocelyn Mora, Susan Morpeth, Srinivas Murthy, Clare Nourse, Matthew O'Sullivan, David Paterson, Mical Paul, Neta Petersiel, Lina Petrella, Sarah Pett, David Price, Jason Roberts, Owen Robinson, Ben Rogers, Benjamin Saville, Matthew Scarborough, Marc Scheetz, Oded Scheuerman, Kevin Schwartz, Simon Smith, Tom Snelling, Marta Soares, Christine Sommerville, Andrew Stewardson, Neil Stone, Archana Sud, Robert Tilley, Steven Tong, Rebecca Turner, Jonathan Underwood, Sebastiaan van Hal, Lesley Voss, Genevieve Walls, Rachel Webb, Steve Webb, Lynda Whiteway, Heather Wilson, Terry Wuerz, Dafna Yahav.

***Financial support.*** This work is supported by the Australian National Health and Medical Research Council (NHMRC) (APP1184238); the Canadian Institutes of Health Research (CIHR) (APP437329); the Health Research Council of New Zealand (HRC) (20/344); and the United Kingdom National Institute for Health Research (NIHR133719). A. M. reports statistical design support for this manuscript from the University of Melbourne. A. C. B. receives an NHMRC Investigator grant (GNT1175509). G. M. reports a grant to institution (Middlemore Clinical Trials) from the HRC. J. M. reports an NHMRC Clinical Trial & Cohort grant for the SNAP platform (payments made to Telethon Kids Institute). M. P. C. reports research payments made to institution from the CIHR and research support from his institution, the McGill University Health Center Department of Medicine. R. K. M. reports personal salary support from study investigators. S. Y. C. T. reports grants to support clinical trial and Career Development Fellowship from the NHMRC. T. C. L. reports operating funds for SNAP from the CIHR. J. A. R. reports funding from the NHMRC for an Investigator Grant (APP2009736) and an Advancing Queensland Clinical Fellowship.

## Supplementary Material

ciac476_Supplementary_Data
