# Correction to: The *Staphylococcus aureus* Network Adaptive Platform Trial Protocol: New Tools for an Old Foe

**DOI:** 10.1093/cid/ciac730

**Published:** 2023-03-18

An error appeared in the corrected proof publication of this article (Tong et al. “The *Staphylococcus aureus* Network Adaptive Platform Trial Protocol: New Tools for an Old Foe.” *Clin Infect Dis*; https://doi.org/10.1093/cid/ciac476). The list of members of the *Staphylococcus aureus* Network Adaptive Platform (SNAP) Study Group was incomplete. An amended list of study group members appears below; names of collaborators who were initially left off the list appear in bold. Additionally, two researchers initially included in the study group, Kevin Brown and Nan Vasilunas, have been removed from the list of contributors.

Nick Anagnostou, Sophia Archuleta, Eugene Athan, **Lauren Barina**, Emma Best, Max Bloomfield, **Jennifer Bostock**, Carly Botheras, Asha Bowen, Philip Britton, **Hannah Burden**, Anita Campbell, Hannah Carter, Matthew Cheng, Ka Lip Chew, Ivor Russel Lee Ming Chong, Geoffrey Coombs, Peter Daley, Nick Daneman, Jane Davies, Joshua Davis, Yael Dishon-Benattar, Ravindra Dotel, Adrian Dunlop, Felicity Flack, Katie Flanagan, Hong Foo, Nesrin Ghanem-Zoubi, Stefano Giulieri, Anna Goodman, Jennifer Grant, Daniel Gregson, Stephen Guy, Amanda Gwee, Erica Hardy, Andrew Henderson, George Heriot, Benjamin Howden, **Fleur Hudson**, Jennie Johnstone, Shirin Kalimuddin, Dana de Kretser, Andrea Kwa, Todd Lee, Amy Legg, Roger Lewis, **Martin Llewelyn**, Thomas Lumley, David Lye, Derek MacFadden, Robert Mahar, Isabelle Malhamé, Michael Marks, Julie Marsh, Marianne Martinello, Gail Matthews, Colin McArthur, Anna McGlothlin, Genevieve McKew, Brendan McMullan, Zoe McQuilten, Eliza Milliken, Jocelyn Mora, Susan Morpeth, Srinivas Murthy, Clare Nourse, Matthew O'Sullivan, David Paterson, Mical Paul, Neta Petersiel, Lina Petrella, **Sarah Pett**, David Price, Jason Roberts, James Owen Robinson, Benjamin Rogers, Benjamin Saville, **Matthew Scarborough**, Marc Scheetz, Oded Scheuerman, Kevin Schwartz, Simon Smith, Thomas Snelling, **Marta Soares**, Christine Sommerville, Andrew Stewardson, **Neil Stone**, Archana Sud, **Robert Tilley**, Steven Tong, Rebecca Turner, **Jonathan Underwood**, Sebastiaan van Hal, Lesley Voss, Genevieve Walls, Rachel Webb, Steve Webb, Lynda Whiteway, Heather Wilson, Terence Wuerz, Dafna Yahav

The authors regret the error.

