# Supplementary material for: The Staphylococcus aureus Network Adaptive Platform Trial Protocol: New Tools for an Old Foe
Source: Clin Infect Dis. 2022 Jun 19;75(11):2027–34. doi: 10.1093/cid/ciac476 (PMC9710697; doi:10.1093/cid/ciac476)
Supplement: ciac476_Supplementary_Data [file ciac476_supplementary_data.zip › SNAP Adjunctive Treatment Domain-Specific Appendix.pdf]

Domain-Specific Appendix:

Adjunctive Treatment Domain

Staphylococcus aureus Network Adaptive Platform trial (SNAP)

Adjunctive Treatment Domain-Specific Appendix Version 1.0 dated 01 June 2021

Summary

In this domain of the SNAP trial, participants with *Staphylococcus aureus* bacteraemia (SAB) admitted to participating hospitals will be randomised to receive one of two interventions:

- Clindamycin (or lincomycin where clindamycin is not available) IV 600mg q8h OR clindamycin 450mg PO q8h for 5 days
- No adjunctive treatment

At this participating site the following interventions have been selected within this domain:

☐ Clindamycin/(lincomycin where clindamycin is not available)

☐ No adjunctive treatment

**SNAP: Adjunctive Treatment Domain Summary**	
Interventions	● Clindamycin (or lincomycin where clindamycin is not available) IV<br>● No Adjunctive Treatment
Silos, domains, and cells	Platform eligible participants within the MSSA, PSSA, or MRSA silos in the adjunctive treatment domain will be analysed. Participants in all three silos are randomised between the same set of adjunctive interventions. A single population parameter for the domain will be estimated and reported using data pooled across silos.
Evaluable treatment-by-treatment interactions	Treatment-treatment interactions will be evaluated between interventions in this domain, and interventions in the cell corresponding to the MRSA silo in the backbone antibiotic domain. No other interactions will be evaluated with any other domain.
Randomisation	Participants will be randomised at platform entry in a fixed 1:1 ratio across the domain. A participant's allocated intervention will be revealed at the time that domain-specific eligibility is confirmed. Response adaptive randomisation may be applied if additional interventions within this domain are added in future versions of this DSA.
Domain Specific Inclusions	Inclusion criteria are the same as the Platform (see Core Protocol Section 6.5).<br>Patients are eligible for this domain regardless of *S. aureus* susceptibility testing results to clindamycin.
Domain Specific Exclusions	Patients will be excluded from this domain if, at the time of eligibility assessment, they have any of the following:<br>1. Previous type 1 hypersensitivity reaction to lincosamides<br>2. Currently receiving clindamycin (lincomycin) or linezolid which cannot be ceased or substituted<br>3. Necrotising fasciitis<br>4. Current *C. difficile* associated diarrhoea (any severity) or severe diarrhoea from any cause<br>5. Known CDAD (*C. difficile* Associated Diarrhoea) in the past 3 months, or CDAD relapse in the past 12 months<br>6. At the time of domain eligibility assessment, more than 4 hours has elapsed since platform entry<br>7. Treating team deems enrolment in this domain is not in the best interest of the patient

Endpoints	Primary platform endpoint: All-cause mortality at 90 days from platform entry.<br>Secondary platform endpoints: refer to Core Protocol Section 6.8.<br>Secondary domain-specific endpoints:<br>1. All-cause diarrhoea any time from domain reveal up to platform day 14 or acute hospital discharge, whichever occurs first<br>2. Change in CRP from platform day 1 until day 5 (+/-1)<br>3. Persistent bacteraemia (positive blood culture at platform day 5 (+/-1)<br>4. Meeting 2 or more SIRS Criteria simultaneously on platform day 5
Decision criteria	The primary objective for this domain is to determine if adjunctive clindamycin is **superior** to no adjunctive treatment. Superiority is defined as an OR < 1 for the primary endpoint (where an OR > 1 indicates an increase in mortality for adjunctive antibiotic treatment compared to no adjunctive treatment). A domain stopping decision will be recommended for superiority if, at a pre-specified interim analysis, the posterior probability of superiority in this domain is greater than 99%. A domain stopping decision for futility of the superiority objective will be recommended if, at a pre-specified interim analysis, the posterior probability of OR <1/1.2 for the primary endpoint in this domain is less than 1%.<br>If, at any pre-specified interim analyses, the thresholds for the decision criteria are not met within a cell, then recruitment into the cell will continue.
Pre-specified secondary analyses	Pre-specified secondary analyses on the primary estimand will be performed by modifying the primary statistical model to account for the following covariates and their treatment interactions:<br>1. No resistance, inducible resistance, or constitutive resistance to clindamycin (3 groups)<br>2. Severe disease phenotype versus not (see full text for definition)<br>3. PVL-positive isolate versus not

TABLE OF CONTENTS

1. ABBREVIATIONS AND GLOSSARY 8

2. PROTOCOL APPENDIX STRUCTURE 10

3. ADJUNCTIVE TREATMENT DOMAIN-SPECIFIC APPENDIX VERSION 11

3.1. Version history 11

4. ADJUNCTIVE TREATMENT DOMAIN GOVERNANCE 11

4.1. Domain members 11

4.2. Contact Details 11

5. ADJUNCTIVE TREATMENT DOMAIN-SPECIFIC WORKING GROUP AUTHORISATION 12

6. BACKGROUND AND RATIONALE 13

6.1. Domain definition 13

6.2. Domain-specific background 13

6.2.1. Clindamycin background 13

6.2.2. Evidence for efficacy of clindamycin 14

6.2.3. Potential adverse effects of clindamycin 17

6.2.4. Need for a clinical trial of clindamycin 17

7. DOMAIN OBJECTIVES 17

8. TRIAL DESIGN 18

8.1. Population 18

8.2. Eligibility criteria 18

8.2.1. Domain inclusion criteria 18

8.2.2. Domain exclusion criteria 18

8.2.3. Intervention exclusion criteria 19

8.3. Interventions 19

8.3.1. Adjunctive Treatment Domain Interventions 19

8.3.2. Timing of initiation of Adjunctive Treatment Domain 20

8.3.3. Duration of administration of Adjunctive Treatment Domain 20

8.4. Concomitant care 20

8.4.1. Implications of allocation status for eligibility in other domains 20

8.5. Endpoints 20

8.5.1. Primary endpoint 20

8.5.2. Secondary endpoints 20

9. TRIAL CONDUCT 21

9.1. Domain-specific data collection 21
9.1.1. Microbiology 21
9.1.2. Clinical data and sample collection 22
9.1.3. Domain-specific study timeline 23
9.1.4. Domain-specific study visit day details 23
9.2. Criteria for discontinuation 24
9.3. Blinding 24
9.3.1. Blinding 24
9.3.2. Unblinding 25
10. STATISTICAL CONSIDERATIONS 25
10.1. Estimands, endpoints, and intercurrent events 25
10.1.1. Primary estimand 25
10.1.2. Secondary estimands 25
10.2. Statistical modelling 27
10.2.1. Primary model 27
10.2.2. Secondary models 27
10.3. Decision criteria 27
10.4. Randomisation 28
10.5. Interactions with other domains 28
10.6. Pre-specified secondary analyses 28
10.7. Principal stratum policy 29
10.7.1. If allocated to adjunctive treatment 29
10.7.2. If allocated to no adjunctive treatment 29
11. ETHICAL CONSIDERATIONS 29
11.1. Data Safety and Monitoring Board 29
11.2. Domain-specific consent issues 30
12. GOVERNANCE ISSUES 30
12.1. Funding of domain 30
12.2. Funding of domain interventions and outcome measures 30
12.3. Domain-specific declarations of interest 30
13. REFERENCES 31

TABLE OF TABLES

Table 1. Guideline recommendations for adjunctive protein synthesis inhibitors in *S. aureus* infections 14

Table 2. Human studies that have reported adjunctive clindamycin as an anti-toxin antibiotic for *Staph. aureus* infection 15

Table 3. Domain-specific schedule of visits, data collection and follow-up. 23

1. ABBREVIATIONS AND GLOSSARY

BC	Blood Culture
CDAD	*C. difficile* Associated Diarrhoea
CRP	C-reactive Protein
D/C	Discharge
DSA	Domain-Specific Appendix
DSWG	Domain-Specific Working Group
DSMB	Data Safety and Monitoring Board
EOS	Earl Oral Switch
EUCAST	European Committee on Antimicrobial Susceptibility Testing
GTSC	Global Trial Steering Committee
HDU	High Dependency Unit
HR	Heart Rate
ICU	Intensive Care Unit
IDSA	Infectious Diseases Society of America
IQR	Interquartile Range
IV	Intravenous
IVI	Intravenous Infusion
IVIG	Intravenous Immunoglobulin
MIC	Minimum Inhibitory Concentration
MRSA	Methicillin-resistant *Staphylococcus aureus*
MSSA	methicillin-susceptible *Staphylococcus aureus*
OPAT	Outpatient Parenteral Antimicrobial Therapy
OD	Odds Ratio
PO	Oral Administration
PSSA	Penicillin-susceptible *Staphylococcus aureus*
PSI	Protein Synthesis Inhibitor
PVL	Panton-Valentine Leucocidin
QID	Four times a day
RAR	Response Adaptive Randomisation
RCT	Randomised Controlled Trial

RR	Respiratory Rate
RSA	Region-Specific Appendix
SAB	*Staphylococcus aureus* Bacteraemia
SAE	Serious Adverse Event
SIRS	Systemic Inflammatory Response Syndrome
SNAP	*Staphylococcus aureus* Network Adaptive Platform trial
SSTIs	Skin and Soft Tissue Infections
TSST-1	Toxic Shock Syndrome Toxin 1
TDS	Three times a day
WBC	White Blood Cell Count

2. PROTOCOL APPENDIX STRUCTURE

The structure of this protocol is different to that used for conventional trials because this trial is highly adaptive and the description of these adaptations is better understood and specified using a 'modular' protocol design. While all adaptations are pre-specified, the structure of the protocol is designed to allow the trial to evolve over time, for example by the introduction of new domains or interventions or both, and commencement of the trial in new geographical regions.

The protocol has multiple modules, in brief, comprising a Core Protocol (overview and design features of the study), a Statistical Analysis Appendix (details of the current statistical analysis plan and models, including simulations to support trial design), multiple Domain-Specific Appendices (DSA) (detailing all interventions currently being studied in each domain), and multiple Region-Specific Appendices (RSA) (detailing regional management and governance).

The Core Protocol contains all information that is generic to the trial, irrespective of the regional location in which the trial is conducted and the domains or interventions that are being tested. The Core Protocol may be amended but it is anticipated that such amendments will be infrequent.

The Core Protocol does not contain information about the intervention(s) within each domain, because one of the trial adaptations is that domains and interventions will change over time. Information about interventions, within each domain, is covered in a DSA. These Appendices are anticipated to change over time, with removal and addition of options within an existing domain, at one level, and removal and addition of entire domains, at another level. Each modification to a DSA will be subject to a separate ethics application for approval.

The Core Protocol does not contain detailed information about the statistical analysis or simulations, because the analysis model will change over time in accordance with the domain and intervention trial adaptations but this information is contained in the Statistical Analysis Appendix. These Appendices are anticipated to change over time, as trial adaptations occur. Each modification will be subject to approval from the Global Trial Steering Committee (GTSC) in conjunction with advice from the Statistical Subcommittee and the Data and Safety Monitoring Committee (DSMC).

The Core Protocol also does not contain information that is specific to a particular region in which the trial is conducted, as the locations that participate in the trial are also anticipated to increase over time. Information that is specific to each region that conducts the trial is contained within a RSA. This includes information related to local management, governance, and ethical and regulatory aspects. It is planned

that, within each region, only that region's RSA, and any subsequent modifications, will be submitted for ethical review in that region.

The current version of the Core Protocol, DSAs, RSAs, and the Statistical Analysis Appendix is listed in the Protocol Summary and on the study website (https://www.snaptrial.com.au/).

3. ADJUNCTIVE TREATMENT DOMAIN-SPECIFIC APPENDIX VERSION

The version of the Adjunctive Treatment Domain-Specific Appendix is in this document's header and on the cover page.

3.1. Version history

Version 1: Approved by the Adjunctive Treatment Domain-Specific Working Group (DSWG) on the 11th of March 2021.

4. ADJUNCTIVE TREATMENT DOMAIN GOVERNANCE

4.1. Domain members

Chair: Associate Professor Asha Bowen

Members:
Professor Josh Davis
Associate Professor Steven Tong
Dr Ravindra Dotel
Dr Derek MacFadden
Dr Simon Smith
Dr Lesley Voss
Dr Neta Petersiel

4.2. Contact Details

Chair:

Associate Professor Asha Bowen

Perth Children's Hospital

15 Hospital Avenue, Nedlands, WA 6009

Australia

Phone 0061 412 608 003

Email asha.bowen@health.wa.gov.au

Project Manager:

Jocelyn Mora SNAP Clinical Trial Manager

The Peter Doherty Institute for Infection and Immunity

792 Elizabeth St Melbourne VIC AUSTRALIA

+61 (0) 38344 2554

jocelyn.mora@unimelb.edu.au

5. ADJUNCTIVE TREATMENT DOMAIN-SPECIFIC WORKING GROUP AUTHORISATION

The Adjunctive Treatment Domain-Specific Working Group (DSWG) have read the appendix and authorise it as the official Adjunctive Treatment Domain-Specific Appendix for the SNAP trial.

Signed on behalf of the committee,

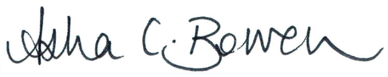

Chair ______________________ Date 29.03.2021
A/Prof Asha Bowen

6. BACKGROUND AND RATIONALE

6.1. Domain definition

This is a domain within the SNAP trial to test the effectiveness of adjunctive treatment in patients with *Staphylococcus aureus* bacteraemia who are admitted to a participating hospital.

6.2. Domain-specific background

S. aureus is a virulent pathogen (1). *S. aureus* bacteraemia results in a mortality of 15-20%, and complications such as abscess formation, infective endocarditis and bone/joint infections are common (1). The array of *S. aureus* exotoxins, surface virulence factors and enzymes contribute to its pathogenesis (2-5). All *S. aureus* strains may harbour haemolysins (e.g., alpha toxin), nucleases, proteases, lipases, hyaluronidase, and collagenase, while many strains may also produce leucocidins (e.g., Panton-Valentine Leucocidin or PVL), toxic shock syndrome toxin 1 (TSST-1), exfoliative toxins and various enterotoxins (2,3,6). Limiting exotoxin expression and release by *S. aureus* could theoretically limit its virulence and improve clinical outcomes (5).

6.2.1. Clindamycin background

Clindamycin is a protein synthesis inhibitor (PSI) antibiotic which decreases bacterial production of multiple exotoxins (4). This is achieved even with sub-minimum inhibitory concentration (MIC) levels of the antibiotic, which has little if any effect on bacterial growth. Its anti-toxin activity is retained in inducible clindamycin-resistant strains (7) but is unclear in constitutive clindamycin-resistant strains (8). The lincosamide antibiotic group has two antibiotics, of which clindamycin has a better pharmacokinetic profile and is more commonly used antitoxin agent than lincomycin (4). Hence, clindamycin is the preferred choice but if unavailable it can be substituted with lincomycin (9).

Several other antibiotics have the potential to inhibit protein production by *S. aureus* and other bacteria, including macrolides, linezolid, aminoglycosides and tetracyclines. However, of all PSI antibiotics, clindamycin is the most commonly used for this indication, as it has shown the most consistent PSI activity (4). Other potential advantages of clindamycin include lack of inoculum effect (unlike β-lactam antibiotics such as “penicillin”) (10), activity during the stationary phase of growth (10), and repression of penicillin-induced exotoxin production (11,12) (Note: vancomycin or daptomycin have a neutral effect on exotoxin production) (13,14).

6.2.2. Evidence for efficacy of clindamycin

Many guidelines recommend using clindamycin (or lincomycin) in serious *S. aureus* infections based on animal studies and observational reports (4,5) for the presumed anti-toxin benefits.

Table 1. Guideline recommendations for adjunctive protein synthesis inhibitors in *S. aureus* infections

(All recommendations are based on expert opinions with limited clinical evidence available)

Guidelines	Recommended adjunctive therapy	Indications	Remarks
Therapeutic Guidelines (Australia): Antibiotic. (2019)(9)	Clindamycin (or lincomycin): For the first 72 hours	Staphylococcal toxic shock syndrome and empirical treatment of necrotising skin and soft tissue infections	No recommendations provided for necrotising *S. aureus* pneumonia
SSTI guidelines, IDSA, USA (2014)(15)	Clindamycin	-	Not recommended in polymicrobial necrotising fasciitis, unless a suspected Group A Streptococcal infection
MRSA treatment guidelines, IDSA, USA (2011)(15)	Clindamycin, Linezolid	Necrotising pneumonia, severe sepsis or toxic shock syndrome	Only to be considered in specific scenarios
Health Protection Agency England (2008)(17)	Linezolid + Clindamycin (high dose, 1.2g QID IV) + Rifampicin	Empiric therapy for PVL *S. aureus* pneumonia or severe *S. aureus* sepsis	To suppress PVL and alpha-toxin. Rifampicin for better tissue penetration and an intracellular activity. Advises against flucloxacillin in severe toxin mediated MSSA infections, as it may augment toxin production
French recommendations (2011)(18)	Clindamycin, Linezolid, Rifampicin	Severe *S. aureus* SSTIs including *S. aureus* necrotising fasciitis, necrotising pneumonia, bone and joint infections	Recommendations are for suspected PVL positive *S. aureus* infections

SSTI: Skin and soft tissue infection; IDSA: Infectious Diseases Society of America

Campbell *et al* have previously described the studies evaluating exotoxin inhibition by clindamycin (4). The majority of these are in vitro or in vivo studies and have shown inhibition of various exotoxins. The available human studies are summarised in the below table. Unlike the laboratory studies, human studies have shown mixed results albeit with low level evidence arising from case reports or case series. The only randomised controlled trial using clindamycin as adjunctive therapy in addition to standard antibiotics was for patients with cellulitis (19). Hence, definitive evidence in a serious infection (like *S. aureus* bacteraemia) is lacking.

Table 2. Human studies that have reported adjunctive clindamycin as an anti-toxin antibiotic for *Staph. aureus* infection

Case report and in vitro study, (Stevens 2006)(20)	MSSA	Empiric treatment with linezolid for the first 48 hours followed by definitive treatment with clindamycin was used. In the in *vitro* arm of the study, both linezolid and clindamycin were shown to completely inhibit TSST-1 synthesis compared to untreated, nafcillin-treated, and vancomycin-treated cultures.
Case report (Rouzic 2010)(20)	MSSA, CA-MRSA	All three cases (2 MSSA, 1 MRSA) with severe necrotising pneumonia survived. Treatment included various antibiotics including clindamycin, linezolid and rifampicin. Two cases also received intravenous immunoglobulin (IVIG). One case underwent pleural decortication and the other had pleural fluid drainage. Decreased PVL in sputum with the start of anti-toxin therapy was described in one case.
Case report (Pasquier 2010)(22)	*S. aureus* (MSSA)	Two cases of severe *S. aureus* pneumonia described. Cases received clindamycin/linezolid and IVIG. Both cases survived despite severe disease. Decrease in PVL in expectoration was shown. The case report did not provide conclusive proof that the improvement and decrease in PVL was due to anti-toxin therapy.
Case series (Li 2011)(23)	MSSA, MRSA	A review of 93 cases of *S. aureus* necrotising pneumonia (80% isolates were MRSA) found that antibiotic therapy that included an anti-toxin agent (clindamycin or linezolid) was associated with lower mortality (p=0.007). Retrospective design, publication bias and lack of control group were the major drawbacks.
Case series (Boan 2015)(24)	MSSA and MRSA	A retrospective review. 79 MSSA and 62 MRSA isolates were PVL positive. 92 MSSA and 56 MRSA were PVL negative. There was no significant difference in 30-day mortality with adjunctive lincosamide/linezolid therapy in PVL positive compared to PVL negative *S. aureus* infections (2.7% vs. 5.3%, p = 0.534).

Randomised Controlled Trial (Brindle 2017)(19)	Nil micro evaluation	The only RCT evaluating adjunctive clindamycin as an anti-toxin agent. Treatment of cellulitis with 500mg QID flucloxacillin and either 300mg QID clindamycin or placebo for 2 days in 410 adults. Day 5 outcomes were similar between the two groups, with diarrhoea being much more common in clindamycin recipients.

However, there are no controlled trials testing this strategy in *S. aureus* bacteraemia, apart from a single pilot trial called CASSETTE (5). The CASSETTE trial was a pilot, open-label RCT which randomised 34 adults and children with severe *S. aureus* disease to clindamycin 600mg TDS IVI (or 450mg TDS PO as an optional step down) for 7 days or not (5). Pilot trials are useful to discover feasibility and to inform future trial design. They are not powered to test hypotheses. From the pilot trial, which took over 2.5 years at 6 sites to recruit the 34 participants from 127 screened, key lessons learned that have informed the Clindamycin DSA include:

- Participants appear to have equipoise with 34/40 (85%) eligible participants approached for consent said yes
- The severe disease phenotype is difficult to identify and enrol. The variability in severe disease and definitions that we applied were hard to recruit with. 27/127 (22%) screened did not meet severe criteria. In SNAP, all participants with SAB will be eligible which will minimise this problem.
- Patients should be identified by automated lab alerts where possible, not relying on clinicians (i.e. use sterile site culture positive as a criterion to make recruitment easier). 127 were screened, but this is a likely underestimate of the true potential pool of patients over 2.5 years at 6 sites.
- We can successfully design and run an RCT that incorporates both children and adults, but this presents challenges in presenting and interpreting data
- Minimise data collection wherever possible
- One of the most common reasons for participant ineligibility was not able to be randomised within 72 hours of index culture (38/127, 30%). This is likely to improve in SNAP as all participants with SAB are eligible and use of automated lab alerts will improve this further.
- Collect all data in a format to analyse primary and secondary outcomes. IV to Oral Switch date was not collected as a discrete endpoint and as such unable to be analysed.

The key results from CASSETTE are that there is no difference in days alive and free of SIRS Criteria within the first 14 days following randomisation. The mean was 4.76 and 4.59 days in groups A and B respectively. There was a mortality difference with 4/17 participants dying by day 90 in group B and

0/15 deaths in group A. There was a trend towards earlier resolution of CRP in group B at 5 days (IQR 3-7) compared to group A at 7 days (IQR 3-14). There was no difference in all cause diarrhoea between groups.

6.2.3. Potential adverse effects of clindamycin

Clindamycin is a registered therapeutic agent for use in susceptible infections (24). It is considered safe to use in children, pregnancy and breastfeeding. Nil dosage modification is required in renal impairment including patients receiving dialysis. Gastrointestinal side effects such as diarrhoea, nausea, vomiting and abdominal cramps are commonly reported, and *C. difficile* associated diarrhoea is a serious but uncommon adverse effect (<1%) (25). Rash or itch could occur but serious skin reactions are rare. Blood dyscrasias, polyarthritis and hepatotoxicity are also rare. Intravenous clindamycin if given very rapidly could lead to hypotension and even cardiac arrest, although this is extremely uncommon, and never occurs if the dose is given more slowly (24).

6.2.4. Need for a clinical trial of clindamycin

Given the theoretical benefits of clindamycin in *S. aureus* bacteraemia, it is surprising that no RCTs have definitively tested this therapy. There is a large variation in the attitudes and practices of clinicians with regard to this intervention, ranging from “I would never use it” to “every patient should receive it”. In a recent Australia and New Zealand practice and attitudes survey, 93% of infectious diseases physicians said they had equipoise and would be willing to randomise patients with *S. aureus* bacteraemia to receive adjunctive therapy with clindamycin or not (26). One difficulty of the CASSETTE trial was identifying a group of patients with a severe disease phenotype in whom to test the intervention (this resulted in a very low eligibility rate). Given that *S. aureus* bacteraemia is by definition an invasive and severe disease, and that all strains of *S. aureus* produce multiple exotoxins, SNAP is an ideal platform to test the effectiveness of this intervention versus no adjunctive therapy in a large number of patients with *S. aureus* bacteraemia.

7. DOMAIN OBJECTIVES

The objective of this domain is to determine the effectiveness of adjunctive therapy (meaning treatment given in addition to the primary, or “backbone” antibiotic, and which is aimed primarily at modifying bacterial function rather than killing bacteria *per se*) for patients with *S. aureus* bacteraemia requiring admission to hospital.

We hypothesise that the probability of all-cause mortality at 90 days after platform entry will be lower in those who are allocated to receive clindamycin compared with those who are not.

The following interventions will be available:

- Clindamycin (or lincomycin where clindamycin is not available)
- No adjunctive treatment

Only among participants in the MRSA silo, we hypothesise that the treatment effect of the adjunctive therapy domain may differ depending on allocation status in the backbone domain. This is an intervention by intervention interaction between this domain and the backbone therapy domain.

8. TRIAL DESIGN

This domain will be conducted as part of the SNAP trial (see Core Protocol Section 6). Treatment allocation will be randomly allocated, as described in the Core Protocol Section 6.7.

8.1. Population

Patients with *S. aureus* bacteraemia admitted to a participating hospital.

8.2. Eligibility criteria

Patients are eligible for this domain if they meet all of the platform-level inclusion and none of the platform-level exclusion criteria (see Core Protocol Section 6.5) AND all of the domain-level inclusion and none of the domain-level exclusion criteria. Patients eligible for SNAP may have conditions that exclude them from the Adjunctive Treatment Domain (see exclusion list) but are eligible for this domain regardless of *S. aureus* susceptibility testing results to clindamycin.

8.2.1. Domain inclusion criteria

Patients are eligible for this domain regardless of *S. aureus* susceptibility testing results to clindamycin (see microbiology section).

8.2.2. Domain exclusion criteria

Patients will be excluded from this domain if, at the time of eligibility assessment, they have any of the following:

1. Previous type 1 hypersensitivity reaction to lincosamides
2. Currently receiving clindamycin (lincomycin) or linezolid which cannot be ceased or substituted

3. Necrotising fasciitis
4. Current *C. difficile* associated diarrhoea (any severity) or severe diarrhoea from any cause (Severe diarrhoea is defined as Grade 3 or higher (CTCAE v5) = Increase of ≥7 stools per day over baseline)
5. Known CDAD (*C. difficile* Associated Diarrhoea) in the past 3 months, or CDAD relapse (new clinical episode of diarrhoea within 3 months of a previous diagnosis of CDAD, and thought by the treating clinician to be attributable to *C. difficile*) in the past 12 months
6. At the time of domain eligibility assessment, more than 4 hours has elapsed since platform entry
7. Treating team deems enrolment in this domain is not in the best interest of the patient

8.2.3. Intervention exclusion criteria

There are no intervention specific exclusions.

8.3. Interventions

8.3.1. Adjunctive Treatment Domain Interventions

- Clindamycin 600mg IV q8h for 5 days
    - Substitute with IV lincomycin 600mg q8h if clindamycin is not available
    - No dosage adjustment is needed in renal impairment
    - OR option for PO clindamycin 450mg PO q8h, for part of all of the treatment course, at discretion of site PI.
- No adjunctive treatment

600mg q8h IVI was chosen as the recommended dose, based on

i) French and UK guidelines recommending up to 900mg per dose (27, 28)
ii) A hollow fibre model suggesting 600mg is the optimal dose to inhibit exotoxin production (29, 30)

An oral alternative is allowed for sites and investigators who prefer oral dosing for cost or convenience reasons. However, the oral dose is capped at 450mg, as this is maximum licensed dose in most regions, and higher doses tend to have poor gastrointestinal tolerability.

8.3.2. Timing of initiation of Adjunctive Treatment Domain

Immediately after domain eligibility confirmed. Inclusion in this domain is not dependent upon clindamycin susceptibility testing results.

8.3.3. Duration of administration of Adjunctive Treatment Domain

5 calendar days (i.e. from platform days 1 to 5, acknowledging that many patients will only receive 1 or 2 doses on day 1).

8.4. Concomitant care

All enrolled patients will receive the highest-quality guideline concordant care as described in the Core Protocol Section 6.7.4.

If the treating team decides to add an antibiotic to the patient's treatment regimen during the total index hospital admission, they should avoid clindamycin, lincomycin and linezolid (acknowledging that some patients in the EOS domain may receive clindamycin or linezolid, and this will be recorded and adjusted for). Other PSI antibiotics have minimal, if any, antitoxin activity. These other antitoxin antibiotics include macrolides (erythromycin, azithromycin, roxithromycin, clarithromycin), Streptogramins (quinupristin-dalfopristin, pristinamycin), chloramphenicol, fusidic acid, aminoglycosides (e.g. gentamicin), tetracyclines (e.g. doxycycline) and tigecycline (31, 32).

8.4.1. Implications of allocation status for eligibility in other domains

Nil.

8.5. Endpoints

8.5.1. Primary endpoint

The primary endpoint for this domain is the platform primary endpoint (all-cause mortality at 90 days after platform entry) as specified in Core Protocol Section 6.8

8.5.2. Secondary endpoints

All secondary endpoints as specified in the Core Protocol Section 6.8 apply to the adjunctive treatment domain.

The domain-specific secondary endpoints are:

1. All-cause diarrhoea any time from domain reveal up to platform day 14 or acute hospital discharge, whichever occurs first
    a. Defined as at least 3 loose stools per day, as reported by the patient or a treating nurse or doctor or reported in medical records
2. Change in CRP from platform day 1 until day 5 (+/-1)
    a. Day 1 CRP means any blood CRP measurement taken on platform day 1 or the calendar day prior to platform entry. If there is more than one measurement, the value recorded is the one taken closest to the time of platform entry.
3. Persistent bacteraemia defined as a positive blood culture on platform day 5 +/- 1 day. If blood culture at day 2 or 3 is negative, then day 5 blood culture will be assumed to be negative.
4. Meeting 2 or more SIRS Criteria simultaneously on platform day 5
    a. Abnormal body temperature (<36 or >38 degrees C)
    b. Tachypnoea or mechanical ventilation (RR>20 breaths per minute in an adult, age dependent in children)
    c. Tachycardia (HR >90 beats per minute in an adult, age dependent in children)
    d. Abnormal leucocyte count (using WBC taken on day 5 +/- 1 day).

9. TRIAL CONDUCT

9.1. Domain-specific data collection

9.1.1. Microbiology

Inducible Clindamycin resistance

The prevalence of this phenomenon varies by location. Most automated systems have an inducible clindamycin phenotype detection well and therefore inducible resistance detection will only be an issue for laboratories that use disc diffusion as their primary susceptibility testing methodology. Inducible clindamycin resistance can be detected by antagonism of clindamycin activity by a macrolide agent using the D-test.

D-test:

Place the erythromycin and clindamycin disks 12-20 mm apart (edge to edge) and look for antagonism (the D phenomenon) to detect inducible clindamycin resistance.

Phenotype: requiring D-test.

Erythromycin	Clindamycin	D-test
R	S	Required

EUCAST: If inducible resistance is detected, then report as resistant and consider adding this comment to the report: "Clindamycin may still be used for short-term therapy of less serious skin and soft tissue infections as constitutive resistance is unlikely to develop during such therapy".

Resistance classification

Erythromycin	Clindamycin	D-test	
R	R	Not required	Constitutive Resistance
R	S	Positive	Inducible Resistance
R	S	Negative	Clindamycin Susceptibility

Enrolment into the clindamycin adjunctive therapy domain will be considered irrespective of susceptibility testing with analysis performed based on resistance classification.

Step-down to PO clindamycin therapy is an inappropriate option in the presence of inducible resistance irrespective of the source of bacteraemia.

9.1.2. Clinical data and sample collection

Additional domain-specific data will be collected:

- CRP on platform day 5
- SIRS criteria on platform day 5
- Diarrhoea during the acute index hospital admission up to platform day 14, or acute index hospital discharge (whichever is earlier)

9.1.3. Domain-specific study timeline

Table 3. Domain-specific schedule of visits and follow-up.

Platform Day	Day 1	Day 2-4	Day 5	Day 14	Acute D/C[a]
Administer clinda/linco (if in clinda group)	X	X	X		
Avoid clinda/linco (if in non-clinda group)	X	X	X	X	X
CRP			X		
SIRS criteria			X[b]		

[a] Acute discharge means the end of the acute index inpatient admission
[b] Use WBC taken on Day 5 +/-1 day

9.1.4. Domain-specific study visit day details

All core study visit details are specified in the Core Protocol (Section 8.8). Data will be collected, as per the CRFs, on platform day 1, day 8-10 (for data from platform days 1-7), day 15-18 (for data from platform days 8-14), day 28, day 42, day 90, and acute and total discharge.

Additional domain-specific study procedures are outlined below.

9.1.4.1. Day 0/ Screening

In addition to the screening procedures outlined in the Core Protocol (Section 8.8), additional domain-specific screening procedures will occur as per the eligibility criteria outlined in Section 8.2.

9.1.4.2. Day 1

In addition to the activities outlined in the Core Protocol (Section 8.8) additional domain-specific activities will be conducted, including:

- Administer clindamycin/lincomycin if in the adjunctive treatment arm

9.1.4.3. Day 2 - 4

Core activities on Day 2 - 4 are outlined in the Core Protocol (Section 8.8).

Additional domain-specific activities will be conducted on Day 2 - 4, including:

- Administer clindamycin/lincomycin if in the adjunctive treatment arm

9.1.4.4. Day 5

In addition to the activities outlined in the Core Protocol (Section 8.8) additional domain-specific activities will be conducted, including:

- CRP on day 5
- SIRS criteria on day 5
- Administer clindamycin/lincomycin if in the adjunctive treatment arm

9.1.4.5 Day 14

In addition to the activities outlined in the Core Protocol (Section 8.8) additional domain-specific activities will be conducted, including:

- Data collection for diarrhoea during platform days 1 - 14

9.1.4.6 Day of acute index hospital discharge

In addition to the activities outlined in the Core Protocol (Section 8.8) additional domain-specific activities will be conducted, including:

- Data collection for diarrhoea from domain entry to acute index hospital discharge

9.2. Criteria for discontinuation

Refer to Core Protocol Section 8.10 for criteria for discontinuation of participation in the SNAP trial.

9.3. Blinding

9.3.1. Blinding

At platform entry, participants will be randomised within this domain, irrespective of silo, to adjunctive therapy (clindamycin) or no adjunctive therapy. Participants will remain blinded to the allocation until the participant satisfies the domain eligibility criteria. Once a participant is eligible, the allocation will be revealed and the investigator and participant will be unblinded. The study drugs are used open label and therefore blinding is not relevant on a per patient basis. On a study-wide basis, investigators, site and study personnel will remain blinded to pooled domain outcomes and summaries until the DSMC has recommended terminating the domain for non-inferiority, superiority or futility.

9.3.2. Unblinding

Unblinding is not relevant at the individual participant and site investigator level as once eligibility is reached, the allocation is not blinded.

10. STATISTICAL CONSIDERATIONS

10.1. *Estimands, endpoints, and intercurrent events*

10.1.1. Primary estimand

The primary estimand, endpoint, and intercurrent events strategy for this domain is the core SNAP primary endpoint (i.e. all-cause mortality 90 days after platform entry) and a treatment policy strategy, as specified in Statistical Analysis Appendix.

10.1.2. Secondary estimands

All core secondary estimands, endpoints, and intercurrent events strategies are specified in the Statistical Analysis Appendix.

The domain-specific secondary estimands, endpoints, and intercurrent events are defined as follows:

Estimand/Objective/Target population	Endpoint/Population-level summaries	Intercurrent events strategy
**Estimand B.1**<br>To evaluate, within the domain, the effect of intervention compared to the domain control, on the probability of **all-cause mortality at 90 days after platform entry**, in platform eligible participants who adhered to domain-specific allocated treatment.	Endpoint: All-cause mortality at 90 days after platform entry#.<br>Population summary: Log-odds ratio of the stated event between intervention and control groups for the domain.	Principal stratum policy (per protocol principle), see Section 10.7.
**Estimand B.2**<br>To evaluate, within the domain, the effect of the revealed randomised intervention compared to the domain control, on the probability of **all-cause diarrhoea at any time from domain reveal up to 14 days following platform entry or until**	Endpoint: Three or more loose stools per day, as reported by the patient, a treating nurse or doctor, or reported in the patient's medical records.<br><br>Population summary: As for estimand B.1.	Treatment policy strategy (intent-to-treat principle)

**hospital/OPAT discharge, whichever occurs first**, in platform eligible participants.		
**Estimand B.3**<br>To evaluate, within the domain, the effect of the revealed randomised intervention compared to the domain control, on the **change in blood concentration of C-reactive protein at platform 5 day (+/- 1 day) compared to baseline**, in platform eligible participants.	Endpoint: Baseline CRP is defined as any blood CRP measurement taken on the calendar day of or day before platform entry. If there is more than one measurement, the baseline value is the one taken closest to the time of platform entry. Follow up CRP is measured on 5 day (1 +/- day).<br><br>Population summary: Mean difference in endpoint between intervention and control groups within each relevant cell.	Treatment policy strategy (intent-to-treat principle)
**Estimand B.4**<br>To evaluate, within the domain, the effect of revealed randomised intervention compared to the domain control, on the **probability of a persistent bacteraemia at platform day 5 (+/- 1 day)**, in platform eligible participants.	Endpoint: Persistent bacteraemia at day 5 (+/- 1 day) following platform entry. A patient with a negative blood culture at day 2 will be assigned no persistent bacteraemia at day 5 if that patient has no subsequent positive blood cultures from day 3 to day 5.<br><br>Population summary: As for estimand B.1.	Treatment policy strategy (intent-to-treat principle)
**Estimand B.5**<br>To evaluate, within the domain, the effect of the revealed randomised intervention compared to the domain control, on the probability of **meeting two or more SIRS Criteria simultaneously on day 5 following platform entry, whichever occurs first**, in platform eligible participants.	Endpoint: Simultaneously, on day 5 following platform entry, meeting two or more of the following SIRS Criteria:<br>- Abnormal body temperature (< 36 or > 38 degrees C)<br>- Tachypnoea or mechanical ventilation (RR > 20 per minute in adults, age dependent in children)<br>- Tachycardia (HR > 90 per minute in adults, age dependent in children)<br>- Abnormal leucocyte count (using WBC taken on day 5 +/- 1 day).	Treatment policy strategy (intent-to-treat principle)

	Population summary: As for estimands B.1.	

Platform entry is defined as the date that consent was obtained.

10.2. Statistical modelling

10.2.1. Primary model

The population summary (log-odds ratio) for the binary primary endpoint (all-cause mortality at 90 days) will be modelled using a Bayesian binomial model with a logit link. See the Statistical Analysis Appendix.

10.2.2. Secondary models

The population summaries for all core secondary endpoints as specified in the Core Protocol (Section 6.8.2) will be modelled as specified in the Statistical Analysis Appendix.

The domain-specific endpoints B.1, B.2, B.4, and B.5 described in Section 10.1 are all binary and have log-odds population summaries that will be modelled using a Bayesian binomial model with a logit link. The domain-specific endpoint B.3 described in Section 10.1 is continuous and, after any necessary transformations, will be modelled using a Bayesian linear model with normally distributed errors. See the Statistical Analysis Appendix.

10.3. Decision criteria

Stopping decisions in this domain are based on whether the posterior probability that the intervention is **superior,** with respect to the domain control, is above, or below, pre-specified thresholds. Where a domain stopping decision is recommended because a posterior probability is below the pre-specified threshold, we say that it is futile to continue with the objective of demonstrating superiority.

The primary objective for this domain is to determine if adjunctive clindamycin is superior to no adjunctive treatment. Superiority is defined as an OR < 1 for the primary endpoint (where an OR > 1 indicates an increase in mortality for adjunctive treatment compared to no adjunctive treatment). A domain stopping decision will be recommended for superiority if, at a pre-specified interim analysis, the posterior probability of superiority is greater than 99%. A domain stopping decision of futility will be declared if, at a pre-specified interim analysis, the posterior probability of OR <1/1.2 for the primary endpoint in this domain is less than 1%.

If, at any interim analysis, the thresholds for the decision criteria are not met within the domain, then recruitment into the domain will continue. In all other respects the stopping rules for this domain are those outlined in the Core Protocol (Section 9.12) and the Statistical Appendix.

10.4. Randomisation

Participants will be randomised at platform entry in a fixed 1:1 ratio. A participant's allocated intervention will be revealed at the time that domain-specific eligibility criteria are confirmed. Response adaptive randomisation may be applied if additional interventions within this domain are included in future versions of this DSA.

10.5. Interactions with other domains

An *a priori* interaction with the MRSA-backbone antibiotic cell and the adjunctive antibiotic domain is considered possible and will be incorporated into the statistical models used to analyse these domains. The rationale for this interaction is that beta-lactam antibiotics (including cefazolin) can upregulate bacterial toxin production (*in-vitro*) and thus the benefit of clindamycin may be larger in those allocated to vancomycin/daptomycin plus cefazolin than in those randomised to vancomycin/daptomycin alone.

A *priori* interaction with the early oral switch domain and the PSSA and MSSA Backbone Domain are not considered likely and will not be incorporated into the statistical models used to analyse this domain.

10.6. Pre-specified secondary analyses

Pre-specified secondary analyses on the primary estimand will be performed by modifying the primary statistical model to include the following covariates and their treatment interactions:

1) No resistance, inducible resistance, or constitutive resistance to clindamycin
   No clindamycin resistance is an isolate that tests fully susceptible to clindamycin on standard antimicrobial susceptibility testing.
   Inducible resistance to clindamycin. It is tested for in the laboratory according to the microbiology appendix.
   The effect of this covariate will be explored as it is unknown whether the resistance profile of clindamycin impacts on its ability to inhibit protein synthesis. This clinical trial will enable us to answer this question in vivo.

2) Severe disease phenotype versus not (defined as ICU/HDU admission at the time of platform entry).

   From the CASSETTE trial, the severe disease phenotype was hypothesised to have greater benefit from clindamycin than no adjunctive treatment. In CASSETTE, 22/37 (59.4%) participants were admitted to the ICU. The pilot trial was unable to answer this question but identified the difficulties in identifying and enrolling this small cohort in a clinical trial. As such, SNAP will include anyone with SAB who is eligible to be in this DSA.

   Severe Disease is defined as ICU and/or HDU admission, at the time of platform entry, as this is pragmatic and easy to collect.

3) PVL-positive isolate versus not

   Panton valentine leucocidin (PVL) is a *S. aureus* toxin encoded by *lukSF-PV*. Not all *S. aureus* carry *lukSF-PV*. The literature is unclear on whether carriage of *lukSF-PV* is associated with a more severe disease phenotype. It is plausible that if clindamycin adjunctive therapy has a clinical benefit, it will be more evident in the PVL positive sub-group.

10.7. Principal stratum policy

The principal stratum policy (also known as a 'per protocol principle') for Estimand B.1 uses the population as described in the following subsections.

10.7.1. If allocated to adjunctive treatment

In the clindamycin group, those participants who have received at least 10 doses of clindamycin/lincomycin between confirmation of domain eligibility and the end of platform day 5.

10.7.2. If allocated to no adjunctive treatment

In the non-clindamycin, per protocol is defined as <2 doses of clindamycin/lincomycin/linezolid between confirmation of domain eligibility and the end of platform day 14.

11. ETHICAL CONSIDERATIONS

11.1. Data Safety and Monitoring Board

The DSMC should be aware of the pre-specified decision criteria for superiority, inferiority, or equivalence of different interventions with respect to the primary endpoint. Determination of the optimal intervention may also involve secondary endpoints (Section 8.5.2).

11.2. Domain-specific consent issues

'Nil'

12.GOVERNANCE ISSUES

12.1. Funding of domain

Interventions are considered standard of care and will be covered by hospital operating budgets. Outcome measures are pragmatic and do not deviate from routine testing performed during usual care for SAB. Funding sources for the SNAP trial are specified in the Core Protocol Section 2.5. This domain has not received any additional domain-specific funding.

12.2. Funding of domain interventions and outcome measures

PVL testing will occur as part of whole genome sequencing of all included *S. aureus* isolates, and will be funded from the SNAP budget, not by sites.

12.3. Domain-specific declarations of interest

All investigators involved in SNAP maintain a registry of interests on the SNAP website. These are updated periodically and publicly accessible on the study website.

13. REFERENCES

1. Tong SYC, Davis JS, Eichenberger E, Holland TL, Fowler VG. Staphylococcus aureus infections: epidemiology, pathophysiology, clinical manifestations, and management. Clin Microbiol Rev [Internet]. 2015 Jul;28(3):603–61. Available from: http://www.ncbi.nlm.nih.gov/pubmed/26016486
2. Dinges MM, Orwin PM, Schlievert PM. Exotoxins of Staphylococcus aureus. Clin Microbiol Rev [Internet]. 2000 Jan;13(1):16–34, table of contents. Available from: http://www.ncbi.nlm.nih.gov/pubmed/10627489
3. Kong C, Neoh H, Nathan S. Targeting Staphylococcus aureus Toxins: A Potential form of Anti-Virulence Therapy. Toxins (Basel) [Internet]. 2016 Mar 15;8(3). Available from: http://www.ncbi.nlm.nih.gov/pubmed/26999200
4. Campbell AJ, Dotel R, Blyth CC, Davis JS, Tong SYC, Bowen AC. Adjunctive protein synthesis inhibitor antibiotics for toxin suppression in Staphylococcus aureus infections: a systematic appraisal. J Antimicrob Chemother [Internet]. 2019;74(1):1–5. Available from: http://www.ncbi.nlm.nih.gov/pubmed/30307507
5. Dotel R, Tong SYC, Bowen A, Nelson JN, O'Sullivan MVN, Campbell AJ, et al. CASSETTE-clindamycin adjunctive therapy for severe Staphylococcus aureus treatment evaluation: study protocol for a randomised controlled trial. Trials [Internet]. 2019 Jun 13;20(1):353. Available from: http://www.ncbi.nlm.nih.gov/pubmed/31196132
6. Peacock SJ, Moore CE, Justice A, Kantzanou M, Story L, Mackie K, et al. Virulent combinations of adhesin and toxin genes in natural populations of Staphylococcus aureus. Infect Immun [Internet]. 2002 Sep;70(9):4987–96. Available from: http://www.ncbi.nlm.nih.gov/pubmed/12183545
7. Hodille E, Badiou C, Bouveyron C, Bes M, Tristan A, Vandenesch F, et al. Clindamycin suppresses virulence expression in inducible clindamycin-resistant Staphylococcus aureus strains. Ann Clin Microbiol Antimicrob [Internet]. 2018 Oct 20;17(1):38. Available from: http://www.ncbi.nlm.nih.gov/pubmed/30342546
8. Herbert S, Barry P, Novick RP. Subinhibitory clindamycin differentially inhibits transcription of exoprotein genes in Staphylococcus aureus. Infect Immun [Internet]. 2001 May;69(5):2996–3003. Available from: http://www.ncbi.nlm.nih.gov/pubmed/11292717
9. eTG complete [digital]. Melbourne: Therapeutic Guidelines Limited; 2019 Jun. <https://www.tg.org.au>.
10. Stevens DL, Gibbons AE, Bergstrom R, Winn V. The Eagle effect revisited: efficacy of clindamycin, erythromycin, and penicillin in the treatment of streptococcal myositis. J Infect Dis [Internet]. 1988 Jul;158(1):23–8. Available from: http://www.ncbi.nlm.nih.gov/pubmed/3292661
11. Dumitrescu O, Badiou C, Bes M, Reverdy M-E, Vandenesch F, Etienne J, et al. Effect of antibiotics, alone and in combination, on Panton-Valentine leukocidin production by a Staphylococcus aureus reference strain. Clin Microbiol Infect [Internet]. 2008 Apr;14(4):384–8. Available from: http://www.ncbi.nlm.nih.gov/pubmed/18261123
12. Stevens DL, Ma Y, Salmi DB, McIndoo E, Wallace RJ, Bryant AE. Impact of antibiotics on expression of virulence-associated exotoxin genes in methicillin-sensitive and methicillin-resistant Staphylococcus aureus. J Infect Dis [Internet]. 2007 Jan 15;195(2):202–11. Available from: http://www.ncbi.nlm.nih.gov/pubmed/17191165
13. Dumitrescu O, Boisset S, Badiou C, Bes M, Benito Y, Reverdy M-E, et al. Effect of antibiotics on Staphylococcus aureus producing Panton-Valentine leukocidin. Antimicrob Agents Chemother [Internet]. 2007 Apr;51(4):1515–9. Available from: http://www.ncbi.nlm.nih.gov/pubmed/17242137
14. Otto MP, Martin E, Badiou C, Lebrun S, Bes M, Vandenesch F, et al. Effects of subinhibitory concentrations of antibiotics on virulence factor expression by community-acquired methicillin-resistant

Staphylococcus aureus. J Antimicrob Chemother [Internet]. 2013 Jul;68(7):1524–32. Available from: http://www.ncbi.nlm.nih.gov/pubmed/23508621

15. Stevens DL, Bisno AL, Chambers HF, Dellinger EP, Goldstein EJC, Gorbach SL, et al. Practice guidelines for the diagnosis and management of skin and soft tissue infections: 2014 update by the Infectious Diseases Society of America. Clin Infect Dis [Internet]. 2014 Jul 15;59(2):e10-52. Available from: http://www.ncbi.nlm.nih.gov/pubmed/24973422
16. Liu C, Bayer A, Cosgrove SE, Daum RS, Fridkin SK, Gorwitz RJ, et al. Clinical practice guidelines by the infectious diseases society of america for the treatment of methicillin-resistant Staphylococcus aureus infections in adults and children: executive summary. Clin Infect Dis [Internet]. 2011 Feb 1;52(3):285–92. Available from: http://www.ncbi.nlm.nih.gov/pubmed/21217178
17. Guidance on the Diagnosis and Management of PVL-Associated Staphylococcus aureus Infections (PVL-SA) in England. 2nd edition Nov 2008. In: Agency HP, editor. London, UK: HPA; 2008.
18. Gillet Y, Dumitrescu O, Tristan A, Dauwalder O, Javouhey E, Floret D, et al. Pragmatic management of Panton-Valentine leukocidin-associated staphylococcal diseases. Int J Antimicrob Agents [Internet]. 2011 Dec;38(6):457–64. Available from: http://www.ncbi.nlm.nih.gov/pubmed/21733661
19. Brindle R, Williams OM, Davies P, Harris T, Jarman H, Hay AD, et al. Adjunctive clindamycin for cellulitis: a clinical trial comparing flucloxacillin with or without clindamycin for the treatment of limb cellulitis. BMJ Open [Internet]. 2017 Mar 17;7(3):e013260. Available from: http://www.ncbi.nlm.nih.gov/pubmed/28314743
20. Stevens DL, Wallace RJ, Hamilton SM, Bryant AE. Successful treatment of staphylococcal toxic shock syndrome with linezolid: a case report and in vitro evaluation of the production of toxic shock syndrome toxin type 1 in the presence of antibiotics. Clin Infect Dis [Internet]. 2006 Mar 1;42(5):729–30. Available from: http://www.ncbi.nlm.nih.gov/pubmed/16447124
21. Rouzic N, Janvier F, Libert N, Javouhey E, Lina G, Nizou J-Y, et al. Prompt and successful toxin-targeting treatment of three patients with necrotizing pneumonia due to Staphylococcus aureus strains carrying the Panton-Valentine leukocidin genes. J Clin Microbiol [Internet]. 2010 May;48(5):1952–5. Available from: http://www.ncbi.nlm.nih.gov/pubmed/20129956
22. Pasquier P, Muller V, Villevieille T, Rousseau JM, Janvier F, Etienne J. Panton-Valentine leukocidin-producing Staphylococcus aureus necrotising pneumonia: measuring toxin levels in microbiological samples to attest of linezolid clinical efficacy. Int J Antimicrob Agents [Internet]. 2010 Jun;35(6):613–4. Available from: http://www.ncbi.nlm.nih.gov/pubmed/20206478
23. Li H-T, Zhang T-T, Huang J, Zhou Y-Q, Zhu J-X, Wu B-Q. Factors associated with the outcome of life-threatening necrotizing pneumonia due to community-acquired Staphylococcus aureus in adult and adolescent patients. Respiration [Internet]. 2011;81(6):448–60. Available from: http://www.ncbi.nlm.nih.gov/pubmed/21051855
24. Boan P, Tan H-L, Pearson J, Coombs G, Heath CH, Robinson JO. Epidemiological, clinical, outcome and antibiotic susceptibility differences between PVL positive and PVL negative Staphylococcus aureus infections in Western Australia: a case control study. BMC Infect Dis [Internet]. 2015 Jan 9;15:10. Available from: http://www.ncbi.nlm.nih.gov/pubmed/25572896
25. Australian Medicines Handbook 2020 (online). Adelaide: Australian Medicines Handbook Pty Ltd; 2020 July. Available from: https://amhonline.amh.net.au/.
26. Tong SYC, Campbell A, Bowen AC, Davis JS. A survey of infectious diseases and microbiology clinicians in Australia and New Zealand about the management of Staphylococcus aureus bacteremia. Clin Infect Dis [Internet]. 2019;69(10):1835–6. Available from: http://www.ncbi.nlm.nih.gov/pubmed/30953050
27. Guidance on the Diagnosis and Management of PVL-Associated Staphylococcus aureus Infections (PVL-SA) in England. In: Agency HP, editor. London, UK: HPA; 2008.
28. Gillet Y, Dumitrescu O, Tristan A, et al. Pragmatic management of Panton–Valentine leukocidin-associated staphylococcal diseases. International Journal of Antimicrobial Agents 2011; 38(6): 457-64

29. Shukla SK, Carter TC, Ye Z, Pantrangi M, Rose WE. Modeling of Effective Antimicrobials to Reduce Staphylococcus aureus Virulence Gene Expression Using a Two-Compartment Hollow Fiber Infection Model. Toxins (Basel). 2020;12(2):69-72
30. Pichereau S, Pantrangi M, Couet W, Badiou C, Lina G, Shukla SK, et al. Simulated antibiotic exposures in an in vitro hollow-fiber infection model influence toxin gene expression and production in community-associated methicillin-resistant Staphylococcus aureus strain MW2. Antimicrob Agents Chemother. 2012;56(1):140-7
31. Poehlsgaard J, Douthwaite S. The bacterial ribosome as a target for antibiotics. Nat Rev Microbiol [Internet]. 2005 Nov;3(11):870–81. Available from: http://www.ncbi.nlm.nih.gov/pubmed/16261170
32. Wilson DN. The A-Z of bacterial translation inhibitors. Crit Rev Biochem Mol Biol [Internet]. 2009;44(6):393–433. Available from: http://www.ncbi.nlm.nih.gov/pubmed/19929179
