# Supplementary material for: The Staphylococcus aureus Network Adaptive Platform Trial Protocol: New Tools for an Old Foe
Source: Clin Infect Dis. 2022 Jun 19;75(11):2027–34. doi: 10.1093/cid/ciac476 (PMC9710697; doi:10.1093/cid/ciac476)
Supplement: ciac476_Supplementary_Data [file ciac476_supplementary_data.zip › SNAP Core Protocol_v1.0_01JUNE2021.pdf]

# CORE PROTOCOL

*Staphylococcus aureus* Network Adaptive Platform trial (SNAP)

| | |
|---|---|
| Study Title<br>Abbreviated Title | Staphylococcus aureus Network Adaptive Platform trial<br>SNAP |
| Clinical trials registration | To be assigned |
| Universal trial number | U1111-1257-3950 |
| Protocol version/date | Version 1.0, dated 01 June 2021 |
| Protocol number | CT19029 |
| Funding source | Australia: National Health and Medical Research Council (NHMRC) Clinical Trials and Cohort Studies funding grant (Award ID: APP1184238) |
| Overarching study sponsor<br>Sponsor for Australian sites<br>Sponsor for NZ sites<br>Sponsor for Canadian sites<br>Sponsor for Israel sites<br>Sponsor for Singapore sites<br><br>Sponsor for UK sites | University of Melbourne<br>University of Melbourne<br>Middlemore Clinical Trials Trust<br>Research Institute of the McGill University Health Centre<br>TBC<br>National Centre for Infectious Diseases, Tan Tock Seng Hospital<br>MRC Clinical Trials Unit at University College London |
| Co-ordination | |
| Overarching Co-ordinating centre | The Peter Doherty Institute for Infection and Immunity, University of Melbourne |
| Collaborating bodies | The Australasian Society for Infectious Diseases Clinical Research Network (ASID CRN) |
| Investigators | |
| Co-ordinating Principal Investigators | Associate Professor Steven Tong[1,2,3,] |

| | |
|---|---|
| | Professor Joshua Davis[3,4] |
| Chief Investigators (Trial Steering Committee) | Associate Professor Asha Bowen[5]<br>Associate Professor Nick Daneman[6]<br>Doctor Anna Goodman[7]<br>Professor Sebastiaan van Hal[8]<br>Doctor George Heriot[2]<br>Doctor Todd Lee[9]<br>Associate Professor David Lye[10]<br>Doctor Anna McGlothlin[11]<br>Associate Professor Zoe McQuilten[12]<br>Doctor Julie Marsh[13]<br>Ms Jocelyn Mora[1]<br>Doctor Susan Morpeth[14]<br>Professor David Paterson[15]<br>Doctor David Price[1]<br>Professor Jason Roberts[15,16]<br>Doctor Genevieve Walls[14]<br>Professor Steve Webb[17]<br>Professor Dafna Yahav[18]<br>Lynda Whiteway, Consumer representative |
| Coordinating and Chief Investigators' Affiliations | 1. Peter Doherty Institute for Infection and Immunity, Victoria, 3000, Australia<br>2. Royal Melbourne Hospital, Melbourne Health, Victoria, 3050, Australia<br>3. Menzies School of Health Research, Casuarina NT 0811<br>4. Hunter New England Health, Lookout Road, New Lambton Heights, NSW, 2305<br>5. Department of Infectious Diseases, Perth Children's Hospital. Wesfarmers Centre for Vaccines and Infectious Diseases, Telethon Kids Institute. Division of Paediatrics, School of Medicine, University of Western Australia. Menzies School of Health Research, Charles Darwin University. Institute for Health Research, The University of Notre Dame, Australia<br>6. Sunnybrook Health Sciences Centre, University of Toronto. Institute for Clinical Evaluative Sciences. Public Health Ontario. 2075 Bayview Ave, TO ON, M4N 3M5<br>7. Department of Infection, Guy's and St Thomas' NHS Foundation Trust, London, SE1 7EH. MRC Clinical Trials Unit at University College London, London, WC1V 6LJ<br>8. Department of Microbiology and Infectious Diseases Royal Prince Alfred Hospital, University of Sydney<br>9. McGill University Health Centre, Montreal, Canada |

10. National Centre for Infectious Diseases; Tan Tock Seng Hospital; Yong Loo Lin School of Medicine; Lee Kong Chian School of Medicine; Singapore
11. Berry Consultants, LLC, Austin, TX
12. Department of Epidemiology and Preventive Medicine, Monash University, Melbourne, Australia
13. Telethon Kids Institute, Perth Children's Hospital, WA, Australia
14. Middlemore Hospital, Counties Manukau Health, Auckland, New Zealand
15. University of Queensland Centre for Clinical Research, Brisbane, Australia
16. Departments of Pharmacy and Intensive Care Medicine, Royal Brisbane and Women's Hospital, Brisbane, Australia
17. Royal Perth Hospital, Perth WA, Australia
18. Infectious Diseases Unit, Rabin Medical Centre, Beilinson Hospital, Petah Tikva, Israel


**Document history:**

| **Version Number** | **Date** | **Summary of changes** |
|---|---|---|
| 1.0 | 29 March 2021 | Final approved version submitted to HREC |
| | | |

**Authorisation:**

| **Co-PI** | **Date** | **Signature** |
|---|---|---|
| Steven Tong | 29 March 2021 | |
| Joshua Davis | 29 March 2021 | |


CONFIDENTIAL

This document is confidential and the property of the SNAP Global Trial Steering Committee. No part of it may be transmitted, reproduced, published, or used without prior written authorisation from the committee.


STATEMENT OF COMPLIANCE

This document is a protocol for a research project. This study will be conducted in compliance with all stipulation of this protocol, the conditions of the ethics committee approval, the NHMRC National Statement on ethical Conduct in Human Research (2007 and all updates), the Integrated Addendum to ICH E6 (R1): Guideline for Good Clinical Practice E6 (R2), dated 9 November 2016 annotated with TGA

comments, and the NHMRC guidance Safety monitoring and reporting in clinical trials involving therapeutic goods (EH59, 2016).

# Table of Contents


**1. ABBREVIATIONS AND GLOSSARY ........ 9**

**2. INTRODUCTION ........ 11**

2.1. Synopsis ........ 11

*2.1.1 Overview of initial trial design ........ 11*

*2.1.2 Full synopsis ........ 11*

2.2. Protocol structure ........ 17

*2.2.1. Core protocol ........ 18*

*2.2.2. Statistical Appendix ........ 18*

*2.2.3. Region-specific appendices (RSAs) ........ 19*

*2.2.4. Domain-specific appendices (DSAs) ........ 19*

*2.2.5. Appendices for sub-studies and for special patient populations ........ 20*

2.3. Lay description ........ 20

2.4. Trial registration ........ 21

2.5. Funding ........ 21

**3. STUDY GOVERNANCE ........ 21**

3.1. Global trial steering committee (GTSC) ........ 22

*3.1.1. Responsibilities ........ 22*

*3.1.2. Members ........ 23*

3.2. Regional trial steering committees (RTSC) ........ 23

*3.2.1. Responsibilities ........ 23*

3.3. Data and safety monitoring committee ........ 24

3.4. Domain Specific Working Groups (DSWGs) ........ 24

*3.4.1. Responsibilities ........ 24*

*3.4.2. Members ........ 24*

3.5. Subcommittees other than DSWGs ........ 25

3.6. Trial sponsors ........ 25

**4. BACKGROUND AND RATIONALE ........ 25**

4.1. *Staphylococcus aureus* bacteraemia ........ 25

4.2. Adaptive platform trials ........ 27

**5. OBJECTIVES ........ 28**

5.1. Primary objective ........ 28

5.2. Secondary objectives ........ 28

**6. STUDY DESIGN ........ 29**

6.1. OVERVIEW ........ 29
6.2. DEFINITIONS FOR SILOS, DOMAINS, CELLS, AND REGIMENS ........ 31
6.3. DEFINITIONS FOR *S. AUREUS* BACTERAEMIA RELATED CLINICAL SYNDROMES ........ 31
*6.3.1. Complicated S. aureus bacteraemia ........ 31*
*6.3.2. Infective Endocarditis ........ 32*
6.4. STUDY SETTING AND PARTICIPATING REGIONS ........ 33
6.5. CORE ELIGIBILITY CRITERIA ........ 33
*6.5.1. INCLUSION CRITERIA ........ 33*
*6.5.2. EXCLUSION CRITERIA ........ 34*
6.6. DOMAIN-SPECIFIC ELIGIBILITY CRITERIA ........ 35
6.7. TRIAL INTERVENTIONS ........ 35
*6.7.1. Domain-specific information and availability ........ 35*
*6.7.2. Treatment allocation ........ 35*
*6.7.3. Adaptation of interventions and domains ........ 36*
*6.7.4. Concomitant Care ........ 36*
6.8. TRIAL ENDPOINTS ........ 37
*6.8.1. Primary endpoint ........ 37*
*6.8.2. Secondary endpoints ........ 37*
6.9. CONTROL OF BIAS ........ 39
*6.9.1. Randomisation ........ 39*
*6.9.2. Allocation concealment ........ 39*
*6.9.3. Blinding of treatment allocation ........ 39*
*6.9.4. Blinding of outcome adjudication ........ 39*
*6.9.5. Follow-up and missing data ........ 40*

**7. CLINICAL QUALITY REGISTRY ........ 40**

7.1. DATA LINKAGE ........ 40

**8. TRIAL CONDUCT ........ 40**

8.1. SITE PARTICIPATION ........ 40
8.2. RECRUITMENT OF PARTICIPANTS ........ 41
8.3. SCREENING ........ 41
8.4. INFORMED CONSENT ........ 42
8.5. TREATMENT ALLOCATION ........ 43
8.6. DELIVERY OF INTERVENTIONS ........ 43
8.7. PARTICIPANT TIMELINE ........ 43
8.8. STUDY DAILY VISIT DETAILS ........ 44
*8.8.1. Screening ........ 44*
*8.8.2. Day 1 ........ 44*

*8.8.3. Day 2* ... *44*
*8.8.4. Day 5 (±1 day)* ... *45*
*8.8.5. Day 7, 14, 28 and 42* ... *45*
*8.8.6. Acute Hospital discharge* ... *45*
*8.8.7. Total Hospital discharge* ... *45*
*8.8.8. Day 90-100* ... *45*
8.9. BLINDING OF ALLOCATION STATUS ... 45
8.10. DISCONTINUATION OF PARTICIPATION ... 46
8.11. DATA COLLECTION ... 46
*8.11.1. Principles of data collection* ... *46*
8.12. DATA MANAGEMENT ... 47
*8.12.1. Source documents* ... *47*
*8.12.2. Data Integrity* ... *47*
*8.12.3. Confidentiality* ... *48*
*8.12.4. Access to Data* ... *48*
*8.12.5. Dissemination Policy* ... *48*
8.13. QUALITY ASSURANCE AND MONITORING ... 49
*8.13.1. Plans for ensuring protocol adherence* ... *49*
*8.13.2. Protocol Deviations and Serious Breaches* ... *50*

**9. PRINCIPLES OF STATISTICAL ANALYSIS ... 50**

9.1. INTRODUCTION ... 51
9.2. BAYESIAN STATISTICAL MODELLING ... 51
9.3. STATISTICAL HANDLING OF INELIGIBLE PATIENTS ... 52
9.4. SUPERIORITY STATISTICAL TRIGGER ... 52
9.5. INFERIORITY STATISTICAL TRIGGER ... 52
9.6. NON-INFERIORITY STATISTICAL TRIGGER ... 52
9.7. DOMAIN FUTILITY TRIGGER ... 52
9.8. ACTION WHEN A STATISTICAL TRIGGER IS ACHIEVED ... 53
9.9. ANALYSIS SET FOR REPORTING ... 54
9.10. SIMULATIONS AND STATISTICAL POWER ... 54
9.11. CO-ENROLMENT WITH OTHER TRIALS ... 54
9.12. CRITERIA FOR TERMINATION OF THE TRIAL ... 55

**10. SAFETY MONITORING AND REPORTING ... 55**

10.1. DEFINITIONS ... 55
10.2. ASSESSMENT OF ADVERSE EVENTS (AES) ... 56
10.3. RECORDING ... 57
10.4. REPORTING ... 57

*10.4.1. Site Responsibilities ........ 58*
*10.4.2. SAEs/SARs not needing expedited reporting ........ 59*
*10.4.3. Sponsor Reporting Procedures ........ 59*

**11. GOVERNANCE AND ETHICAL ISSUES ........ 59**

11.1. MANAGEMENT OF PARTICIPATING SITES AND TRIAL COORDINATION ........ 59
11.2. ETHICS AND REGULATORY ISSUES ........ 59
*11.2.1. Overarching principals ........ 59*
*11.2.2. Approvals ........ 60*
11.3. PROTOCOL MODIFICATIONS- SUBSTANTIAL AMENDMENTS ........ 60
11.4. DECLARATIONS OF INTEREST ........ 61
11.5. COMMUNICATION ........ 61
*11.5.1. Communication of trial results and publication policy ........ 61*
*11.5.2. Authorship policy ........ 61*

**12. REFERENCES ........ 62**

# 1. ABBREVIATIONS AND GLOSSARY

| | |
|---|---|
| Acute index hospitalisation | Initial hospital admission to an acute inpatient facility, this **does not** include HITH/OPAT/COPAT and stepdown inpatient rehabilitation/post-acute care |
| AE | Adverse event |
| AKI | Acute Kidney Injury |
| AR | Adverse reaction |
| ASID CRN | The Australasian Society for Infectious Diseases Clinical Research Network |
| ASP | Antistaphylococcal Penicillin |
| DSMC | Data and Safety Monitoring Committee |
| COPAT | Complex Outpatient Parenteral Antimicrobial Therapy |
| CRF | Case Report Form |
| CT | Computed Tomography |
| DSA | Domain Specific Appendix |
| DSWG | Domain Specific Working Group |
| eCRF | Electronic Case Report Form |
| ESC | European Society of Cardiology |
| FBIS | Functional Bloodstream Infection Score |
| GTSC | Global Trial Steering Committee |
| HITH | Hospital in the home |
| HREC | Human Research Ethics Committee |
| ICMJE | International Committee of Medical Journal Editors |
| ID physician | Infectious Disease physician |
| IDSA | Infectious Diseases Society of America |
| IE | Infective endocarditis |
| IIG | International Interest Group |
| ITT | Intention-to-treat |
| MSSA | Methicillin-Susceptible *Staphylococcus aureus* |
| MRSA | Methicillin-Resistant *Staphylococcus aureus* |
| NHMRC | National Health and Medical Research Council |
| OPAT | Outpatient Parenteral Antimicrobial Therapy |
| PET | Positron Emission Tomography |
| Platform | Patients in the platform are those who meet all core eligibility criteria and consent to inclusion in the platform. Occasional patients in the platform will not receive any randomised intervention (if they are not eligible for any available domain). |
| Platform entry | "Platform entry" is the timepoint when the patient has met core eligibility criteria, given informed consent for the platform, and been randomised |
| PSSA | Penicillin-Susceptible *Staphylococcus aureus* |
| PWID | People Who Inject Drugs |
| RAR | Response Adaptive Randomisation |
| Registry | Patients in the registry include all those in the platform (as defined above) PLUS the "registry only" patients. Registry only patients are those who are not in the platform, but who have consented to being in the registry. |
| RCT | Randomised Control Trial |
| REMAP | Randomised Embedded Multifactorial Adaptive Platform |
| RSA | Region Specific Appendix |
| RTSC | Regional Trial Steering Committee |
| RSI | Reference Safety Information |
| SAB | *Staphylococcus aureus* Bacteraemia |
| SAE | Serious Adverse Event |

| | |
|---|---|
| SAP | Statistical Analysis Plan |
| SAR | Serious Adverse Reaction |
| SAS | Statistical Analysis Subcommittee |
| SNAP | *Staphylococcus aureus* Network Adaptive Platform trial |
| SOP | Standard Operating Procedure |
| SPECT | Single-photon emission computed tomography |
| SSI | Significant Safety Issue |
| SUSAR | Suspected Unexpected Serious Adverse Reaction |
| Total index hospitalisation | Initial hospital admission to an acute inpatient facility, including HITH/OPAT/COPAT and stepdown inpatient rehabilitation/post-acute care (if continuous with the initial inpatient admission) |
| TMG | Trial Management Group |
| TOE | Trans-Oesophageal Echocardiogram |
| TTE | Transthoracic Echocardiogram |
| UAR | Unexpected Adverse Reactions |
| USM | Urgent Safety Measure |

# 2. INTRODUCTION

## 2.1. Synopsis

### 2.1.1 Overview of initial trial design

| Silo | Antibiotic Backbone Domain | Adjunctive Treatment Domain | Early Oral Switch Domain |
|---|---|---|---|
| ***PSSA*** | (Flu)cloxacillin*<br>Penicillin | Clindamycin vs<br>No clindamycin* | Usual care* (*initial 2-6 week antibiotic backbone treatment course given intravenously*) versus early oral switch algorithm (*as detailed in the relevant DSA*) |
| ***MSSA*** | (Flu)cloxacillin*<br>Cefazolin | | |
| ***MRSA*** | Vancomycin* vs<br>Vancomycin plus cefazolin | | |

Note that domains and interventions may be added or dropped during the life of the platform. This initial design is given only as an illustration of the trial's structure.
*=Comparator/control group

### 2.1.2 Full synopsis

| | |
|---|---|
| **TITLE** | SNAP – *Staphylococcus aureus* Network Adaptive Platform trial, |
| **OBJECTIVE** | The objective of SNAP is to identify the effect of a range of clinical interventions on all-cause 90-day mortality in patients with SAB |
| **BACKGROUND** | *Staphylococcus aureus* bacteraemia (SAB) is a common and severe infection with a 90-day mortality of 15-30% (mortality lower in children but up to 5%) despite current best available therapies. There are few high-quality data to inform the management of this infection, with less than 3000 patients randomised into any therapeutic trial for SAB prior to 2020. The current standard treatment for MSSA (methicillin-susceptible *Staphylococcus aureus*) and PSSA (penicillin-susceptible *S. aureus)* is (flu)cloxacillin monotherapy, and for MRSA (methicillin-resistant *S. aureus)* is vancomycin monotherapy, each given for 2-6 weeks intravenously. *In-vitro*, pharmacokinetic and observational data suggest that penicillin may be superior to (flu)cloxacillin for PSSA and that cefazolin may be superior to (flu)cloxacillin for MSSA. The CAMERA1 and 2 trials suggest that adding a beta-lactam to vancomycin for MRSA clears bacteraemia faster, but that the combination of vancomycin and (flu)cloxacillin is nephrotoxic. Preliminary data also suggest that clindamycin, which switches off exotoxin production by *S. aureus*, may improve outcomes, and that it may be possible to switch from intravenous to oral antibiotics part way through the treatment course without |

| | |
|---|---|
| | compromising outcomes. We are using an adaptive platform trial to allow us to simultaneously address these questions in the management of SAB. The trial will include 3 silos (PSSA, MSSA, and MRSA). We plan to test interventions within 3 initial domains (see section 2.1.1), with the potential to add further domains to the platform. |
| **CORE PRIMARY OUTCOME MEASURE** | All-cause mortality 90 days after platform entry. |
| **CORE SECONDARY OUTCOME MEASURES** | A core set of secondary endpoints will be evaluated in each domain:<br>1. All-cause mortality at 14, 28 and 42 days after platform entry<br>2. Duration of survival censored at 90 days after platform entry<br>3. Length of stay of acute index inpatient hospitalisation for those surviving until hospital discharge (**excluding** HITH/COPAT/OPAT/rehab) truncated at 90 days after platform entry<br>4. Length of stay of total index hospitalisation for those surviving until hospital discharge (**including** HITH/COPAT/OPAT/rehab) truncated at 90 days after platform entry<br>5. Time to being discharged alive from the total index hospitalisation (including HITH/COPAT/OPAT/rehab) truncated at 90 days after platform entry (and all deaths within 90 days will be considered ’90 days’)<br>6. Microbiological treatment failure (Positive sterile site culture for *S. aureus* [of the same silo as the index isolate] between 14 and 90 days after platform entry)<br>7. Diagnosis of new foci between 14 and 90 days after platform entry. The presence of new foci will be determined by the site investigator and can incorporate clinical, radiological, microbiological and pathological findings.<br>8. *C. difficile* diarrhea as determined by a clinical laboratory in the 90 days following platform entry for participants ≥2 years of age<br>9. Serious adverse reactions in the 90 days following platform entry<br>10. Health economic costs as detailed in the cost utility analysis appendix<br>11. Proportion of participants who have returned to their usual level of function at day 90 as determined by whether the modified functional bloodstream infection score (FBIS) remained the same or improved between baseline and 90 days after platform entry |

| | |
|---|---|
| | 12. Desirability of outcome ranking 1 (modified Antibiotic Resistance Leadership Group version) at 90 days after platform entry<br>13. Desirability of outcome ranking 2 (SNAP version) at 90 days after platform entry |
| **STUDY DESIGN** | Investigator initiated, Randomised Embedded Multifactorial Adaptive Platform (REMAP) trial, conducted across multiple hospitals in several regions of the world.<br>Domains:<br>• Antibiotic Backbone Domain<br>• Adjunctive Treatment Domain<br>• Early Oral Switch Domain<br>• Other domains to be determined |
| **STUDY DURATION** | Pre-trial set up: Jan 2020 to June 2021<br>Anticipated recruitment period: May 2021 to December 2025, with possibility of extending the study and adding more domains or interventions over time, dependent on funding and the ongoing existence of clinically important addressable unanswered questions. |
| **NUMBER OF PARTICIPANTS** | The initial trial funding and infrastructure will aim to enrol up to 6,000 participants into the platform.<br><br>The number of participants randomised into each domain will be determined by pre-specified repeated analyses on the accumulating data and the application of Bayesian inference using pre-determined rules to trigger platform adaptations, including ceasing recruitment early due to futility, non-inferiority or superiority of an intervention within a domain/silo combination (e.g. the PSSA backbone domain, also known as a “cell”). The number of participants in each cell will be that required to answer the relevant question or to determine futility depending on pre-determined decision criteria). |
| **PLATFORM INCLUSION CRITERIA** | 1. *Staphylococcus aureus* complex grown from ≥1 blood culture<br>2. Admitted to participating hospital at anticipated time of eligibility assessment (OR if patient has died, they were admitted to this site anytime from the time of blood culture collection until the time of eligibility assessment) |

| | |
|---|---|
| **PLATFORM EXCLUSION CRITERIA** | 1. Time of anticipated platform entry is greater than 72 hours post collection of the index blood culture<br>2. Polymicrobial bacteraemia, defined as more than one organism (at species level) in the index blood cultures, excluding those organisms judged to be contaminants by the treating clinicians<br>3. Patient currently being treated with a **systemic** antibacterial agent that cannot be ceased (unless antibiotic is listed in Table 1, which specifies allowed antibiotics with limited absorption from the gastrointestinal tract or negligible antimicrobial activity against *S. aureus*)<br>4. Known previous participation in SNAP<br>5. Known positive blood culture for *S. aureus* (of the same silo: PSSA, MSSA or MRSA) between 72 hours and 180 days prior to the time of eligibility assessment<br>6. Treating team deems enrolment in the study is not in the best interest of the patient<br>7. Treating clinician believes that death is imminent and inevitable<br>8. Patient is for end-of-life care and antibiotic treatment is considered not appropriate<br>9. Patient <18 years of age and paediatric recruitment not approved at recruiting site |
| **RANDOMISATION** | Participants will be randomly allocated to one arm within each domain for which they are eligible (and which their site is participating in) using a web-based module available 24h a day 7 days a week. Randomisation in all possible silos and available domains will occur immediately following provision of consent (which is considered the time of platform entry), however, the reveal of each treatment allocation(s) will be delayed subject to confirmation of domain eligibility, including availability of domain-relevant microbiology and other results or assessments. |
| **BLINDING** | This will be an open-label study unless otherwise specified in a domain-specific appendix. For the overall data and results, only specified members of the statistical analytical team and DSMC will have access to unblinded results, with other trial investigators and staff remaining blinded to the aggregate results until completion of final analysis for a domain or cell. |

| | |
|---|---|
| **ANALYSIS** | The Statistical Appendix contains a detailed description of the statistical models used for estimating the effect of interventions and for assessing interactions between treatment groups. In brief, the SNAP trial will repeatedly fit Bayesian hierarchical logistic models to the accumulating data (updates), over the life of the trial, to estimate model parameters and evaluate pre-specified decision criteria either within a cell or a domain. Hypotheses tested within each cell may include whether an intervention is superior, inferior, equivalent or non-inferior to comparator or control interventions within that cell or domain. Within this design, Bayesian inferences are made using pre-specified statistical models incorporating non-intervention variables that may influence the probability of the primary endpoint, as well as intervention variables across all domains and biologically plausible interactions. When a decision threshold is met for a cell or the domain as a whole, demonstrating non-inferiority, superiority or futility, then recruitment to that domain within that silo (i.e., that cell) or potentially across all silos may be stopped. The life of a domain is defined as the period from the start of recruitment to the domain until decision criteria are satisfied for all silos or trial resources are exhausted.<br><br>For this pragmatic trial, the primary population is all platform eligible participants, analysed in the intervention groups to which they were allocated (intent-to-treat principle). The frequency and timing of the Bayesian updates and the choice of decision quantities and thresholds for trial adaptations and domain conclusions are documented in the Statistical Appendix. They are designed, using simulation, to control the type 1 and type 2 error rates over a plausible range of intervention effects, as summarised by the trial operating characteristics. |

## Site Principal Investigator Agreement

I have read the protocol entitled SNAP – *Staphylococcus aureus* Network Adaptive Platform trial.

By signing this protocol, I agree to conduct the clinical trial, after approval by a Human Research Ethics Committee or Institutional Review Board, in accordance with the protocol and the principles laid down in the Declaration of Helsinki and Good Clinical Practice [Guideline for Good Clinical Practice E6 (R2), dated 9 November 2016].

With the exception of medical emergencies, changes to the protocol will only be implemented after written approval is received from the relevant Human Research Ethics Committee or Institutional Review Board.

I will ensure that my trial staff fully understand and follow the protocol and evidence of their training is documented on the trial training log.

| **Name of Site** | **Name of site PI** | **Signature and date** ***(dd-mmm-yyyy format)*** |
|---|---|---|
| | | |
| | | |
| | | |

## 2.2. Protocol structure

The SNAP trial protocol is presented in a modular format, reflecting the complexity of the adaptive platform trial structure. Modules within the protocol include the Core Protocol, multiple domain-specific appendices (DSAs), multiple region-specific appendices (RSAs), and a Statistical appendix. Figure 1 provides an overview of this structure.

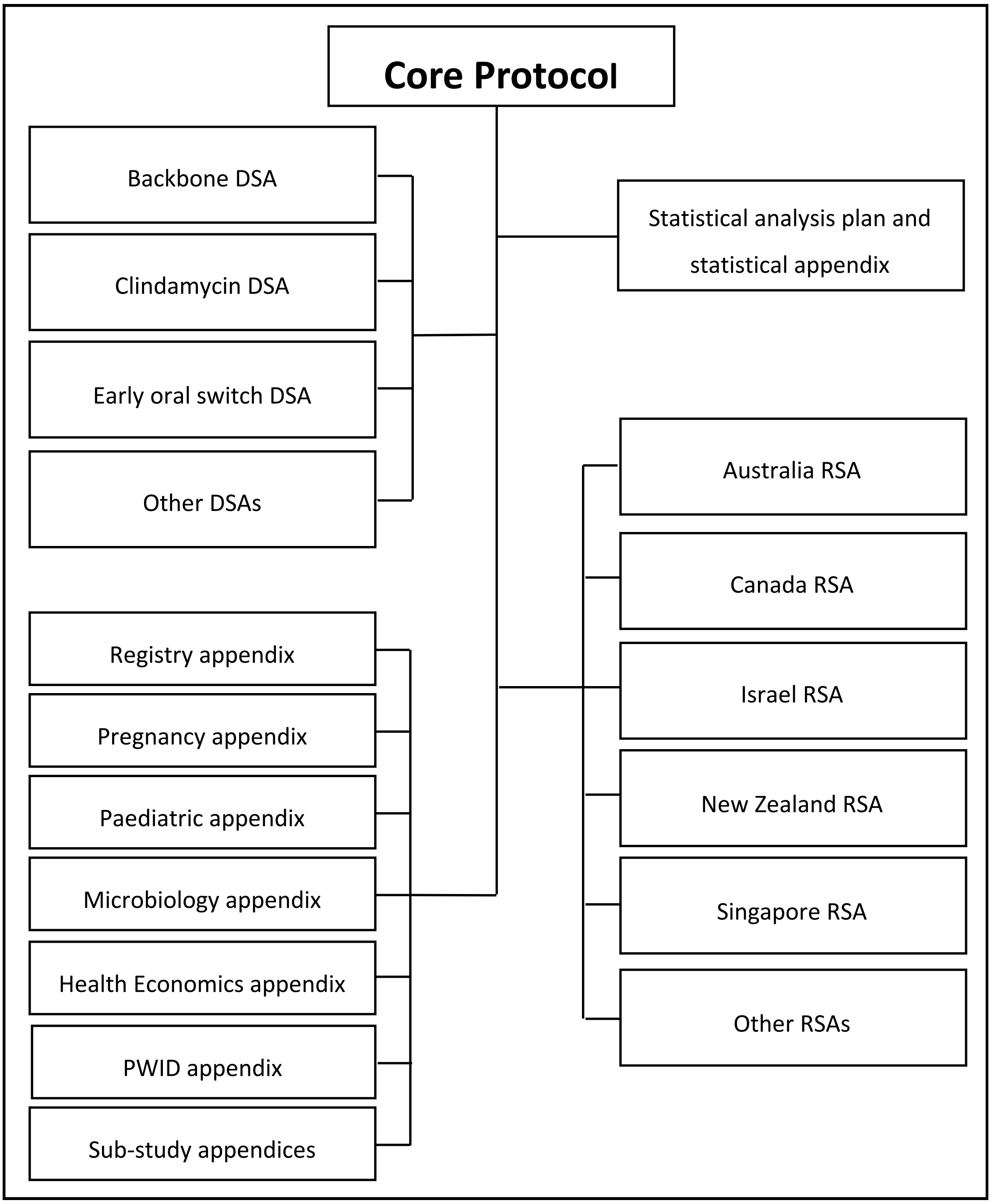


*Figure 1: Protocol structure*

### 2.2.1. Core protocol

The Core Protocol contains all information relevant to trial processes and applies to all regions and to all domains for the duration of the trial. The Core Protocol is anticipated to require only rare modification. It contains the following information:

- The background and rationale for studying SAB as a problem of public health interest
- The rationale for the use of an adaptive platform trial structure
- The trial design, including the structure and processes for an associated clinical quality registry, eligibility criteria for entry into trial platform, randomisation and treatment allocation procedures, core trial endpoints, methods to minimise bias, principles of the statistical analysis, and pre-specified decision criteria that trigger trial adaptations, including ceasing recruitment early due to futility or superiority of an intervention within a cell
- The trial conduct, including methods of recruitment, site-specific timelines, delivery of trial interventions, data collection and management, and procedures related to participant safety and monitoring
- The global trial governance structures

Note that the eligibility criteria, data collection, and secondary outcomes in the Core Protocol are a subset of those in the DSAs. Each domain generally has additional eligibility criteria, data points and secondary outcome measures. Hence each DSA needs to be read in conjunction with the Core Protocol and selected other appendices (e.g. statistical, regional) in order to approximate a typical full trial protocol.

### 2.2.2. Statistical Appendix

The Statistical Appendix contains a detailed description of the statistical methods used for estimating within a cell or a domain the effect of interventions and for assessing interactions between treatment groups. In brief, the SNAP trial will repeatedly fit Bayesian hierarchical logistic models to accumulating data (updates), over the life of the trial, to estimate model parameters and evaluate pre-specified decision criteria that determine for a silo, or across silos, whether an intervention is superior, inferior or non-inferior to comparator or control interventions within that domain. When a decision threshold is met for a cell or the domain as a whole, then recruitment to that domain within that silo (i.e., that cell), or potentially across all silos, may be stopped. The life of a domain is defined as the period from the start of recruitment to the domain until decision criteria are satisfied for all silos or trial resources are exhausted.

The appendix also contains a record of the Monte Carlo simulations used to describe the operating characteristics of the SNAP trial across a range of plausible assumptions regarding outcomes, treatment effects, and interactions between interventions in different domains. The statistical power of each domain and silo (likelihood of [1 – type II error]) and the likelihood of type I error are evaluated using these

simulations. Results from the simulations will be maintained as an operational document publicly accessible on the study website throughout the duration of the trial. Conclusions from the simulations will be included in the Statistical Analysis appendix, which will be amended as required.

The Statistical Appendix is written to be flexible to include new interventions or domains in the SNAP platform trial and will contain information specific to the design of the trial and conduct of the statistical analysis, including:

- The definitions of the core estimands
- Summary of plausible assumptions and scenarios for the simulations
- Summary of the statistical analyses and models
- Summary of decision quantities and thresholds
- Summaries of trial operating characteristics for trial design
- The definition of intervention effect estimate (population level summary)
- Summary of strategies for handling anticipated post-randomisation (intercurrent) events

In addition, a Statistical Analysis Plan (SAP) will be developed providing a detailed and technical description of the principal features of the analysis that is outlined in the Statistical Appendix, in addition to the content of routinely generated statistical reports.

### 2.2.3. Region-specific appendices (RSAs)

The SNAP trial will be conducted in multiple countries around the world with varying legislative, ethical and governance requirements. Each RSA contains information specific to the conduct of the trial in that region, including:

- The definition of the region
- The governance structure within a region
- Ethical and governance issues relevant to a region not covered in the Core Protocol
- The availability of trial domains and interventions within a region
- Region-specific treatment allocation and data management procedures

### 2.2.4. Domain-specific appendices (DSAs)

Each intervention examined within the SNAP trial will be fully described within a DSA. Domains within the SNAP trial will evolve over time, with the potential for progressive additions and removals of both interventions within domains and entire domains as outcome data are accrued. Each DSA (and modifications) will be the subject of a separate ethics application or amendment as per regional requirements. DSAs contain the following information relevant to a domain:

- The background and rationale for each intervention examined

- Domain-specific eligibility criteria
- A description of the interventions and procedures for their delivery
- Domain-specific data and endpoints not included in the Core Protocol
- Domain-specific safety and ethical considerations
- Domain-specific organisational considerations

#### 2.2.5. Appendices for sub-studies and for special patient populations

Sub-studies will also be fully described within appendices. These will contain the following information relevant to a sub-study:

- Background and rationale for the sub-study
- Sub-study eligibility criteria
- Description of the procedures required for conduct of the sub-study

Appendices may also be required for special populations (e.g. pregnant women, children, people who inject drugs). For these special populations, appendices will include any additional exclusion criteria, data collection and ethical/legal considerations which are not already included in the Core Protocol.

### 2.3. Lay description

Infection of the bloodstream with the bacterium *Staphylococcus aureus* (*Staphylococcus aureus* bacteraemia, SAB) is a serious infection that results in 15-30% (mortality lower in children but up to 5%) of affected patients dying within three months of acquiring the infection. Treatment of this infection requires patients to be hospitalised, treated with prolonged antibiotics through an intravenous line, and carefully examined for the occurrence of complications associated with this condition. At present, there are many treatment options in current use, with no clear agreement as to which of these is best. The SNAP trial aims to identify which treatment options for SAB results in the fewest patients dying within the first 90 days after an infection.

In contrast to a conventional clinical trial, the SNAP trial will examine multiple different treatment options at once. Patients will be randomly assigned to different concurrent treatment options currently considered acceptable in routine medical care. The trial will adapt to accumulating trial evidence, on a regular basis, by removing treatment options found to be inferior, incorporating new treatment options, and ensuring that all patients in the trial receive the best treatments once they have been identified. Over time, we hope to determine the best combination of treatment options for patients with SAB.

### 2.4. Trial registration

The SNAP trial will be registered at clinicaltrials.gov (registration number to be assigned)

### 2.5. Funding

Funding will be detailed in region specific appendices.

## 3. STUDY GOVERNANCE

The study governance structure is designed to provide appropriate management of all aspects of the study, taking into account multiple stakeholders and factors, including representation from regions that are participating in the trial, availability of skills and expertise related to trial conduct and statistical analysis, and content knowledge regarding SAB and the interventions that are being evaluated. The governance model is designed to provide effective operational and strategic management of SNAP that operates in multiple regions, is supported by multiple funding bodies and sponsors, and will evolve with addition of further regions and funding bodies as well as changes in the domains and interventions that are being evaluated. The organisational chart for SNAP is outlined in Figure 2.

The Global Trial Steering Committee (GTSC) is the key decision-making body and takes overall responsibility for the trial design, conduct and reporting. Each participating region has a Regional Trial Steering Committee (RTSC) that takes primary responsibility for trial execution in that region. An internationally based Domain-Specific Working Group (DSWG) exists for each domain (or for several domains that are closely related) and has responsibility for design and oversight of each domain.

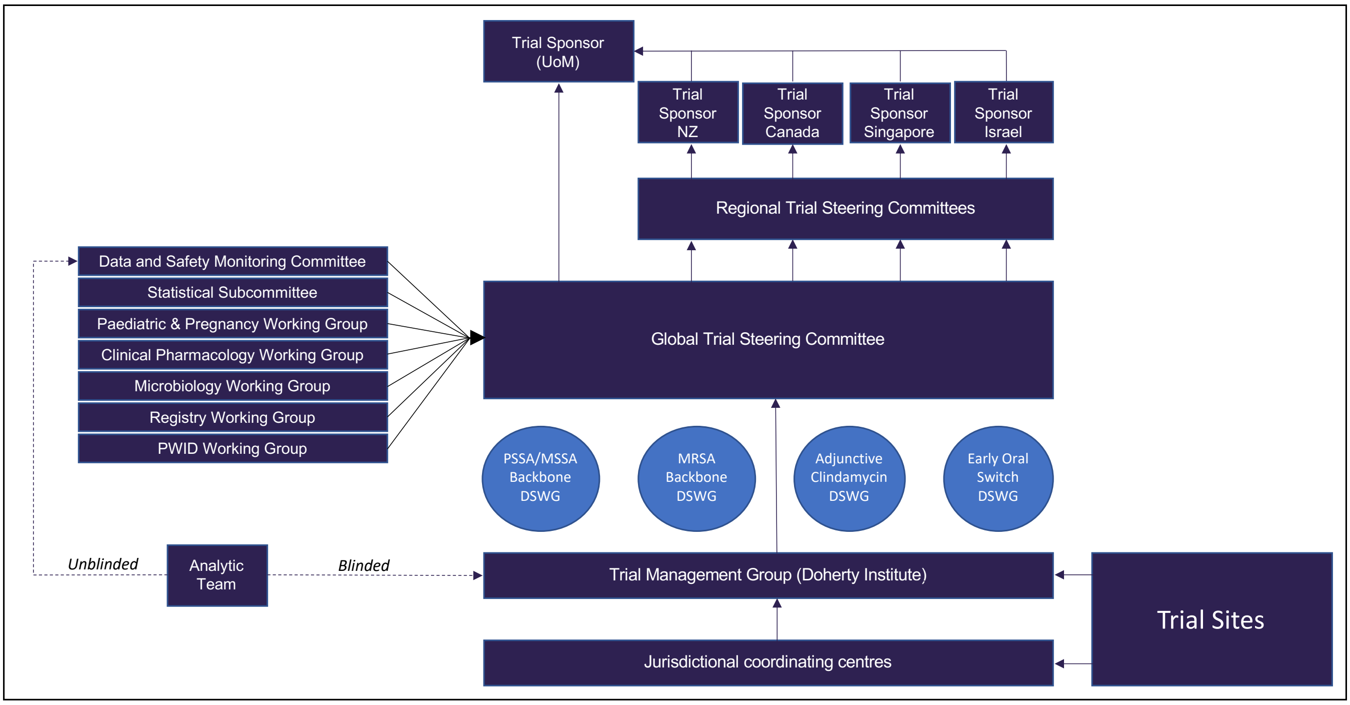


*Figure 2. Study administration and governance structure*

## 3.1. Global trial steering committee (GTSC)

The intent of the GTSC is to have both theoretical and practical experience and knowledge regarding overall design, domain-specific expertise, and regional-specific expertise. As such the GTSC will include clinical trialists, biostatisticians, regional lead investigators, domain lead investigators, trial coordinators, microbiologists and a consumer representative.

### 3.1.1. Responsibilities

The responsibilities of the GTSC are:

- Executive decision-making based on recommendations from the DSMC and other subcommittees and accounting for the interests of all trial participants and stakeholders
- Development and amendment of the Core Protocol
- Approval of all Core Protocol appendices (including domain-specific appendices, statistical appendix and other appendices, and their subsequent amendments)
- Recruitment and approval of new regions to SNAP
- Liaison with the DSMC including, where appropriate, decisions regarding platform amendments and conclusions

- Consideration of requests and approval of additional domains and their nested interventions to SNAP including prioritisation of new domains, new interventions within a domain or both
- Consideration of requests and approval of trial sub-studies
- Liaison with the academic community including the International Committee of Medical Journal Editors (ICMJE) regarding issues such as data sharing, governance and reporting of platform trials including SNAP
- In conjunction with DSMC and DSWGs, the communication and reporting of results from domains
- Approval of manuscripts reporting results that are submitted by DSWGs
- Obtaining funding for SNAP
- Determining the strategic direction of SNAP

#### 3.1.2. Members

Membership of the GTSC comprises an independent chair, investigators from each region, the project manager, at least 1 investigator from Berry Consultants, the chairs of active working groups, at least 1 independent member (defined as an individual who is not a trial investigator named on the grant and is not a site investigator), and a consumer representative. The operation of the GTSC will be specified by Terms of Reference that will be developed and modified, as required, by the GTSC.

### 3.2. Regional trial steering committees (RTSC)

The operation of SNAP in each region is undertaken by that region's RTSC, the composition of which is to be determined by investigators in each region with membership listed in each RSA. Cross-representation between RTSCs is strongly encouraged.

#### 3.2.1. Responsibilities

The responsibilities of each RTSC are:

- Development and amendment of the RSA for that region
- Identification and management of sites in that region
- Obtaining funding for that region
- Liaison with regional funding bodies
- Consideration of the feasibility and suitability of interventions (and domains) for that region
- Liaison with the sponsor(s) for that region
- Management of systems for randomisation and data management for that region

## 3.3. Data and safety monitoring committee

Monitoring of the trial progress and recommendations concerning the overall conduct of the trial will be provided by a single DSMC for all participating sites and regions. The DSMC will operate under a Charter presented separately to this protocol, which will be approved by both the DSMC and the GTSC prior to the commencement of the trial. The DSMC will comprise 3-6 independent members; the chair will have expertise in clinical trial methodology and experience in adaptive trial designs. The DSMC will be unblinded and operate in accordance with current regulatory recommendations.

The DSMC will receive regular blinded reports from the Trial Management Group (TMG) on the trial progress and unblinded reports from the SNAP analytic team on safety and efficacy outcomes (unblinded reports will not be available to those outside of the DSMC or the SNAP analytic team). They will monitor the decision criteria at each Bayesian update (‘interim’), as detailed in the Statistical Appendix to the Core Protocol, and recommend pre-specified trial adaptations to the GTSC if decision thresholds are met. The DSMC will be responsible for communicating to the GTSC the achievement of a decision threshold that may prompt the public declaration of a domain conclusion.

## 3.4. Domain Specific Working Groups (DSWGs)

Each active and future planned domain (or closely related set of domains) will be administered by a DSWG.

### 3.4.1. Responsibilities

The responsibilities of each DSWG are:

- Development and amendment of the DSA
- Proposal and development of new interventions within a domain
- In conjunction with the GTSC, and following a recommendation from the DSMC, reporting results from the domain
- Obtaining funding to support the domain, with a requirement that, if such funds are obtained, that an appropriate contribution to the conduct of the SNAP platform is also made.

### 3.4.2. Members

Membership of each DSWG is set out in the corresponding DSA but should comprise individuals that provide broad international representation, content knowledge of the domain, and expertise of trial conduct and design or policy implementation. Membership selection should take into account gender,

geographical and craft group (infectious diseases, pharmacology, biostatistics, microbiology etc) equity and diversity.

### 3.5. Subcommittees other than DSWGs

Other subcommittees contributing to the trial include:

- SNAP statistical subcommittee
- SNAP analytic team
- SNAP paediatric and pregnancy working group
- SNAP microbiology working group
- SNAP registry working group
- SNAP clinical pharmacology working group
- SNAP people who inject drugs (PWID) working group

### 3.6. Trial sponsors

The overarching sponsor of SNAP is the University of Melbourne. Each region will have a regional sponsor who will take responsibility for regional conduct, governance and insurance of the trial. Regional sponsors can be found in each region’s RSA.

## 4. BACKGROUND AND RATIONALE

### 4.1. *Staphylococcus aureus* bacteraemia

***Staphylococcus aureus* bacteraemia (SAB) is a major public health problem**

SAB is among the most common and serious bacterial infections with an estimated 5,000 episodes per year in Australia with attributable mortality being 15-20% (1-3). The median age of infection is 62 years. All patients require hospitalisation, receive a minimum of two weeks of intravenous antibiotics and the average length of stay in hospital is 22 days (3). SAB affects 13,000 people in England alone each year (4). The average length of stay in hospital in the UK is 22 days, with 23% being readmitted within a 12-week period (5, 6). In Canada, there are 22.4 cases of SAB per 100,000 population/year, and this infection is associated with substantial short- and long-term mortality: 12.0% at 7 days, 22.5% at 30 days, 27.9% at 60 days, 30.4% at 90 days, and 39.7% at 365 days (personal communication, N.Daneman, 2021). In Israel, 2,300 cases of SAB were reported in 2019, 27% of them were MRSA (7). Median age of SAB in Israel has been reported to be 73 years and 30-day mortality approached 40% among SAB hospitalised patients in one study (8). In a Singaporean national point prevalence survey conducted between July 2015 and February 2016, SAB was the commonest pathogen implicated in Singaporean healthcare-associated infections (9). In a matched case-control study of 181 MRSA infection cases and 351 non-infected controls,

MRSA infection was independently associated with mortality (14.4% vs. 1.4%, OR 5.54, 95% CI 1.63–18.79, p = 0.006), longer hospital length of stay (LOS; median of 32 days vs. 7 days, coefficient: 1.21, 95% CI 1.02–1.40, p < 0.001), higher hospitalisation bills (median of US$18,129.89 vs. US$4,490.47, coefficient: 1.14, 95% CI 0.93–1.35, p < 0.001), higher post-discharge healthcare financial costs (median of US$337.24 vs. US$259.29, coefficient: 0.39, 95% CI 0.06–0.72, p = 0.021), and poorer health-associated quality of life (coefficient: 0.14, 95% CI 0.21 to 0.08,p < 0.001) (10).

In children, the incidence in Australia is between 4 and 8 per 100,000 and in New Zealand between 8 and 14 per 100,000. In both countries, Indigenous children are over-represented in the cohort. The median age of SAB is 6.3 years (IQR 1 – 1.3). The median length of hospitalisation is 15 days (IQR 9 – 31) with median duration of IV therapy also 15 days. Ninety-day all-cause mortality for children is 3-5% (ISAIAH cohort pers communication) (11).

The cost of treatment of SAB exceeds $220 million per year in Australia and £12 000 per patient in the UK (6).

**Existing clinical evidence is limited and practice varies**

Among Australian Infectious Diseases (ID) physicians, RCTs for SAB were ranked in the top 5 highest priorities (12). This priority is a combination of the public health impact and the low quality of existing evidence. There are only 3 high quality RCTs ever published (5, 13, 14). Remarkably, the combined sample size of all published RCTs for patients with SAB is less than 3,000 (5, 15).

There is wide variation in practice among ID physicians. In our recent survey (16), for a typical case of community-acquired SAB, among 168 respondents there were 26 different empiric antibiotic regimens proposed for initial treatment, 17 different regimens proposed once antibiotic-susceptibility information was available, with the most frequent regimen being specified by 83 respondents, and the duration of therapy ranging from 14 to 88 days.

There are no international guidelines specifically for MSSA or PSSA bacteraemia. For MSSA endocarditis, the Infectious Diseases Society of America (IDSA) (17) and European (18) guidelines recommend an anti-staphylococcal penicillin (ASP) (nafcillin or flucloxacillin). For PSSA endocarditis, the IDSA guidelines recommend an ASP (and recommend against penicillin) and the European guidelines make no specific recommendation. For MRSA bacteraemia, IDSA and European guidelines recommend vancomycin or daptomycin. None of these recommendations achieves Level A evidence (multiple populations evaluated, data from multiple RCTs). Australian guidelines recommend flucloxacillin or cefazolin for MSSA, penicillin for PSSA, and vancomycin for MRSA bacteraemia (19).

Practice variation is associated with variation in mortality. An observational analysis of more than 36,000 SAB patients, found that the selection of an antibiotic regimen compliant with guidelines was associated with adjusted odds ratio for mortality of 0.74 (95% CI 0.68-0.79) compared to other therapies (20).

## 4.2. Adaptive platform trials

Adaptive Platform Trials are an innovative trials methodology (21-23) now established for oncology trials (24) and recently funded for infectious disease syndromes of community-acquired pneumonia (REMAP-CAP, NHMRC #1101719, CIA Webb), cystic fibrosis (BEAT-CF, NHMRC #1152376, CIA Snelling), COVID-19 (ASCOT, CIs Tong, Davis, Morpeth, NZ HRC #20/1068 and philanthropy) and *S. aureus* bacteraemia (SNAP, NHMRC #1184238, CIA Tong). Berry Consultants LLC (CI Berry) are at the forefront in design and implementation of platform trials for industry as well as academic investigators and provide the statistical support for SNAP, REMAP-CAP and BEAT-CF.

Conventional RCTs, at the time of design, make assumptions about plausible effect size, incidence of the primary outcome, and sample size; holding these assumptions constant until trial completion. Adaptive Platform Trials incorporate multiple statistical and design features that are not reliant on these types of pre-trial assumptions, instead they allow for trial adaptations based on accumulating data and pre-specified decision criteria that maximise trial efficiency, such as the dropping of ineffective treatments and early stopping for treatment superiority. Our investigator group have completed several SAB RCTs, and learned that serially testing treatment options, while rigorous, is inefficient (and expensive). Platform Trials allow multiple questions to be evaluated simultaneously and sequentially within the platform, and evaluate interactions between different treatment options, to achieve the goal of determining the optimal combination of treatments for the disease as rapidly as possible.

**Design features of SNAP that enhance trial efficiency**

There are 4 critical design features of SNAP that will contribute substantially to enhanced trial efficiency and rapid implementation of trial findings.

Firstly, the trial is highly **pragmatic and embedded** within routine care. The inclusion criteria are easily identified and exclusion criteria minimal. Recruitment will be via a simplified consent process developed in conjunction with health consumers with experience of the disease. All interventions are within the spectrum of current standard care, will be delivered by routine clinical staff, and will be delivered in exactly the same way that superior interventions would be implemented into clinical practice. Wherever possible, routine clinical and administrative data will be used for data collection.

Secondly, we will implement a **universal trial master protocol (Core Protocol)**, identifying pre-specified sub-groups ('silos') according to the antibiotic susceptibility of the *S. aureus* strain. The optimal antibiotic regimen will be determined for each silo but the same primary (90-day mortality) and core secondary

endpoints will apply. Additional questions in other domains will be asked in all patients (i.e., across all silos). By addressing multiple questions in parallel and evaluating interactions between interventions, the platform will reduce the time, cost and sample size required to reach definitive conclusions on optimal therapy compared to sequentially executed, traditionally designed trials.

Thirdly, **frequent analyses on the accumulated data (Bayesian updates)** will be performed so that questions are concluded as soon as there is robust statistical confidence, thereby, not waiting until a fixed sample size has been recruited. This allows the platform to evaluate the estimated treatment effect, including no effect, against pre-specified decision thresholds to conclude superiority and/or non-inferiority (within a clinically accepted margin) as soon as warranted by accrued data. Regular Bayesian updates will be undertaken using a Bayesian Hierarchical Model (25) that estimates the probability of superiority and/or non-inferiority of every intervention that is being evaluated within a cell or domain. Superiority will be declared when an intervention has greater than a pre-specified probability (i.e., threshold) of the primary endpoint being superior compared to control, and non-inferiority will be declared when an intervention has greater than a pre-specified probability (i.e., threshold) of the primary endpoint being less than the clinically accepted margin. The results of the Bayesian updates will be reviewed by an independent DSMC responsible for recommending trial adaptations to the GTSC based on objective pre-specified rules (informed by pre-trial simulations that minimise the risk of type 1 and type 2 errors). Details of pre-trial simulations are provided in the Statistical Analysis appendix.

Lastly, the platform has **global scope and unprecedented sample size**. The platform will initially operate in Australia, Singapore, Canada, Israel, and New Zealand. Further countries may be added. Local funding mechanisms are being sought in each country. We will enrol an estimated 6,000 patients, an order of magnitude larger than any previous pathogen-specific bloodstream infection trial. For the first time in an international global trial, it is planned to include children and adults in the same study.

# 5. OBJECTIVES

## 5.1. Primary objective

The primary objective of the SNAP trial is to examine the effect on all-cause mortality at 90 days of a range of interventions in patients with SAB.

## 5.2. Secondary objectives

The secondary objectives of the SNAP trial include:

- Examining the effect of a range of interventions on several secondary endpoints including mortality, hospital length of stay, treatment failure, treatment complications, and healthcare costs.

- Establishing a biobank of *S. aureus* isolates and patient samples linked to clinical outcomes to elucidate the host- and pathogen-specific mechanisms that underlie responses to different therapies.

# 6. STUDY DESIGN

## 6.1. Overview

The SNAP trial is a multicentre, pragmatic, multi-arm, open-label adaptive platform trial addressing multiple therapeutic questions in patients with SAB. An overview of the SNAP trial design is presented in Figure 3. Patients not eligible for, or not consenting to, randomisation within the SNAP platform, will be asked for consent to participate in the SNAP registry (see registry appendix).

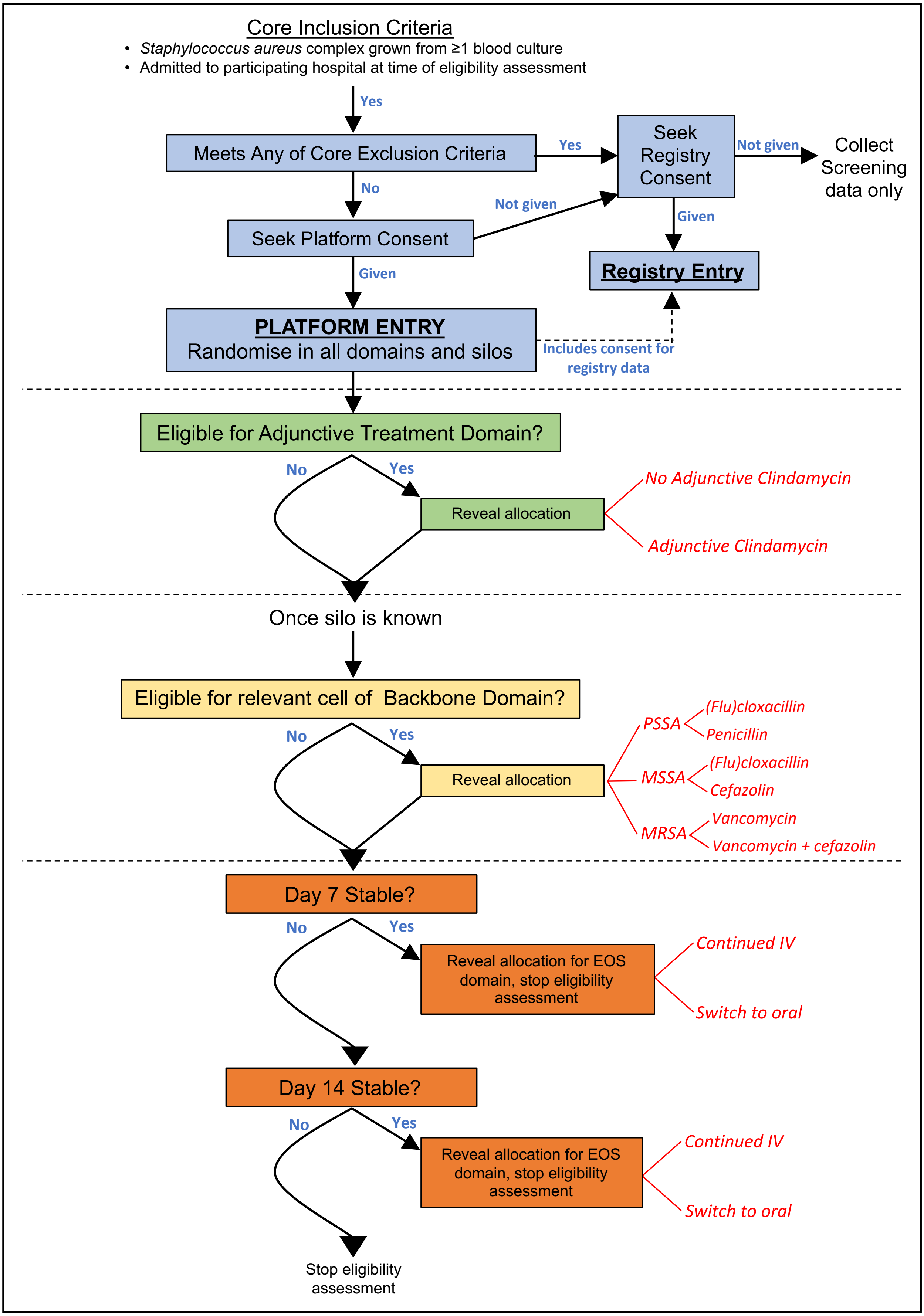


*Figure 3. Overview of initial trial design*

## 6.2. Definitions for silos, domains, cells, and regimens

The SNAP trial is organised into "silos" (subgroups of patients defined by the antibiotic susceptibility of their infecting isolate) and "domains" (groups of alternative interventions to which patients can be randomised). A combination of a domain and a silo will be known as a "cell". For the backbone domain, there are 3 different cells. For the clindamycin and early oral switch domain, there is only one cell in each (i.e., all 3 silos will have the same interventions available to be randomised to).

An example of the design structure is provided here to illustrate the relevant definitions:

| | DOMAIN | | |
|---|---|---|---|
| **SILO** | **Backbone antibiotic** | **Adjunctive antibiotic** | **Early oral switch** |
| *PSSA* | (Flu)cloxacillin vs penicillin | Clindamycin vs no clindamycin | Early oral switch vs continued IV |
| *MSSA* | (Flu)cloxacillin vs cefazolin | | |
| *MRSA* | Vancomycin vs vancomycin + cefazolin | | |

Silo: refers to the antibiotic susceptibility profile of the causative *S. aureus* isolate. There are 3 silos, PSSA, MSSA and MRSA.
Domain: refers to the broad intervention modality. Initially there will be 3 domains, backbone antibiotic, adjunctive antibiotic, and early oral switch.
Cell: refers to the intersection of a silo and domain.

Regimen: refers to the combination of interventions an individual is allocated to receive. In the above example, for each silo, there are eight potential regimens. In the MRSA silo these would be:

| | **Backbone antibiotic** | **Adjunctive antibiotic** | **Antibiotic route** |
|---|---|---|---|
| 1 | Vancomycin | No Clindamycin | Continued IV |
| 2 | Vancomycin | No Clindamycin | Oral switch |
| 3 | Vancomycin | Clindamycin | Continued IV |
| 4 | Vancomycin | Clindamycin | Oral switch |
| 5 | Vancomycin + cefazolin | No Clindamycin | Continued IV |
| 6 | Vancomycin + cefazolin | No Clindamycin | Oral switch |
| 7 | Vancomycin + cefazolin | Clindamycin | Continued IV |
| 8 | Vancomycin + cefazolin | Clindamycin | Oral switch |

## 6.3. Definitions for *S. aureus* bacteraemia related clinical syndromes

### 6.3.1. Complicated *S. aureus* bacteraemia

For the purposes of this trial, complicated *S. aureus* bacteraemia is defined as patients with positive blood culture results with *S. aureus* and one or more of the following:

- An implanted intravascular prosthesis or endovascular device

- Day 2 (from platform entry) blood cultures positive
- Fever (any temperature 37.8°C or above on platform day 2)
- Evidence of deep seated (i.e., not just line related or skin and soft tissue related) or metastatic infection. This includes evidence of endocarditis.

### 6.3.2. Infective Endocarditis

Infective endocarditis (IE) will be defined using the European Society of Cardiology (ESC) 2015 modified criteria (18). Compared to the modified Duke criteria, the ESC criteria include the use of additional imaging modalities that allow the diagnosis of embolic events and cardiac involvement when transthoracic echocardiogram (TTE)/trans-oesophageal echocardiogram (TOE) findings are negative or doubtful. The criteria to be used in SNAP are:

**Definite IE**

Pathological criteria:

- Microorganisms demonstrated by culture or on histological examination of a vegetation, a vegetation that has embolised, or an intracardiac abscess specimen; or
- Pathological lesions; vegetation or intracardiac abscess by histological examination showing active endocarditis

Clinical criteria

- 2 major criteria; or
- 1 major criterion and 3 minor criteria; or
- 5 minor criteria

<u>Major criteria</u>

1. Blood cultures positive for *S. aureus*
2. Imaging positive for IE
    i. Echocardiogram positive for IE:
        - Vegetation
        - Abscess, pseudoaneurysm, intracardiac fistula
        - Valvular perforation or aneurysm
        - New partial dehiscence of prosthetic valve.
    ii. Abnormal activity around the site of prosthetic valve implantation detected by $^{18}$F-FDG PET/CT (only if the prosthesis was implanted for >3 months) or radiolabelled leukocytes SPECT/CT
    iii. Definite paravalvular lesions by cardiac CT

Minor criteria

1. Predisposition such as predisposing heart condition, or injection drug use.
2. Fever defined as temperature >38°C.
3. Vascular phenomena (including those detected by imaging only):
    - Major arterial emboli, septic pulmonary infarcts, infectious (mycotic) aneurysm, intracranial haemorrhage, conjunctival haemorrhages, and Janeway's lesions.
4. Immunological phenomena: glomerulonephritis, Osler's nodes, Roth's spots, and rheumatoid factor.
5. Microbiological evidence: positive blood culture but does not meet a major criterion as noted above

Patients with left and right sided IE will be categorised as left sided IE.

## 6.4. Study setting and participating regions

The SNAP trial will recruit patients with SAB who are admitted to a participating hospital. Hospitals will be considered for participation according to local investigator interest and prespecified criteria, including the annual number of cases of SAB, the available resources to support research activities, and experience in conducting investigator-initiated trials.

Regions are defined as a country or collection of countries with study sites for which a single regional committee is responsible. The trial will be launched in the following regions, and others may be added over time:

- Australia
- Canada
- Singapore
- New Zealand
- Israel

## 6.5. Core Eligibility criteria

### 6.5.1. INCLUSION CRITERIA

Patients must fulfil all of the following criteria to be eligible to enter the SNAP trial:

1. *Staphylococcus aureus* complex grown from ≥1 blood culture
2. Admitted to a participating hospital at the time of eligibility assessment (OR if patient has died, they were admitted to this site anytime from the time of blood culture collection until the time of eligibility assessment)

### 6.5.2. EXCLUSION CRITERIA

Potentially eligible participants meeting any of the following criteria **at the time of eligibility assessment for platform entry** will be excluded from the trial:

1. Time of anticipated platform entry is greater than 72 hours post collection of the index blood culture
   i. Where the time of culture collection is not recorded, the time of laboratory registration of the sample will be used as an alternative
2. Polymicrobial bacteraemia, defined as more than one organism (at species level) in the index blood cultures, excluding those organisms judged to be contaminants by either the microbiology laboratory or treating clinician
3. Patient currently being treated with a **systemic** antibacterial agent that cannot be ceased (unless antibiotic is listed in Table 1, which specifies allowed antibiotics with limited absorption from the gastrointestinal tract or negligible antimicrobial activity against *S. aureus*)
4. Known previous participation in SNAP
5. Known positive blood culture for S. *aureus* (of the same silo: PSSA, MSSA or MRSA) between 72 hours and 180 days prior to the time of eligibility assessment
6. Treating team deems enrolment in the study is not in the best interest of the patient
7. Treating team believes that death is imminent and inevitable
8. Patient is for end-of-life care and antibiotic treatment is considered not appropriate
9. Patient <18 years of age and paediatric recruitment not approved at recruiting site

*Table 1 - Antibacterial agents not fulfilling Exclusion Criterion 3.*

Continued use of the following antibacterial agents after the time of eligibility assessment will not constitute an exclusion criterion for enrolment in the trial. Topical administration of these or any other antibacterial agents is acceptable.

| ***Class*** | ***Agent*** |
|---|---|
| *Antimycobacterial agents* | Clofazimine<br>Dapsone<br>Capreomycin<br>Cycloserine<br>Ethambutol<br>Ethionamide<br>Isoniazid<br>Pyrazinamide |
| *Miscellaneous* | Colistin (enteral)<br>Fidaxomicin<br>Furazolidone |

| | |
|---|---|
| | Neomycin (enteral)<br>Nitrofurantoin<br>Paromomycin<br>Rifaximin<br>Tobramycin (enteral)<br>Trimethoprim (≤300mg/d)<br>Trimethoprim-sulfamethoxazole (prophylaxis i.e. ≤160mg/d TMP component average daily dose in adults or <4mg/kg/d in children)<br>Vancomycin (enteral) |

## 6.6. Domain-specific eligibility criteria

Each domain may have additional criteria for eligibility. Participants who fulfil the above criteria will be assessed for enrolment into all domains active at a participating site. The relevant criteria are provided in each domain's DSA. At least 2 interventions (which may include standard of care) within a domain must be available to an eligible participant in order for that participant to enter the domain.

## 6.7. Trial interventions

### 6.7.1. Domain-specific information and availability

All information regarding the background and delivery of interventions will be contained within the relevant DSA. The minimum number of interventions within a domain is 2. The availability of interventions within domains will be region- and site-specific, although the default position is that all interventions within a domain will be available at all sites.

### 6.7.2. Treatment allocation

The SNAP trial will use random allocation of interventions to participants. Where there are only 2 intervention options within a domain, that domain will be most efficiently powered by equal allocation (1:1 ratio) to the interventions, thereby minimising the time needed to reach a trial conclusion for that domain.

In domains with more than two interventions response adaptive randomisation (RAR) may be considered. Where RAR is implemented, patient- or site-specific issues may result in one or more interventions being unavailable or inappropriate for a particular participant. In this case, provided domain eligibility is retained through the availability of at least 2 interventions, for a given participant the randomisation proportions will be adjusted by blocking the unavailable/contraindicated interventions and dividing the remaining allocation proportions by one minus the sum of the unavailable allocation proportions.

At the time of enrolment, the participant's *S. aureus* isolate antibiotic susceptibility profile (silo) may be unknown, however, some domains may start interventions prior to this information being available. Therefore, participants will be randomised to an intervention in each available (for that trial site) domain for which the participant is currently eligible or might subsequently become eligible. These randomisations are concealed and an allocation is only deemed to have occurred if it is revealed. The timing of reveal of treatment allocation in domains for which a participant is not yet eligible at enrolment will be domain-specific, with some domains employing immediate reveal at the time of enrolment and others delaying reveal until a participant meets the domain eligibility criteria. The rationale, mechanism and implications for statistical analysis of these specifications are described in the DSA of domains that include the potential for delayed eligibility.

### 6.7.3. Adaptation of interventions and domains

The ongoing randomisation of participants to an intervention within a cell will be terminated by the declaration of a cell or domain conclusion (i.e., a decision threshold has been met; see section 9). Cells where a domain conclusion is declared, identifying one intervention as superior will continue to recruit, but with all future participants allocated to the superior intervention. Where a cell decision threshold is reached for noninferiority, the DSMC will recommend to the GTSC whether to stop recruitment into the cell (cell termination, with public declaration) or continue recruitment into the cell to collect more information on potential superiority. For cells with ≥2 interventions, inferior interventions will be dropped and may be replaced. New domains and cells may be added after consideration by the GTSC of clinical relevance and the available statistical power and resources within the trial. All new interventions and domains will be the subject of ethics and regulatory approval prior to initiation.

### 6.7.4. Concomitant Care

Sites will be encouraged to provide the highest-quality guideline concordant care to all enrolled patients, including the following key elements (13):

- Consultation by an infectious diseases physician or clinical microbiologist
- Repeat blood cultures at day 2 of the study (equates to day 3-5 post blood culture collection)
- Source control where relevant and possible (e.g. removal of intravenous lines, drainage of abscesses)
- Echocardiography
- Reasonably frequent laboratory measurements as required for clinical care and for drug safety (creatinine, liver enzymes, complete blood count) (26)

## 6.8. Trial endpoints

### 6.8.1. Primary endpoint

The primary endpoint for all cells and domains will be all-cause mortality at 90 days after platform entry. The primary endpoint will be determined through a search of hospital databases for a record of a participant's death, or follow-up contact with the participant's community healthcare provider, or follow-up contact with the patient or their nominated carer, or linkage with death registries.

### 6.8.2. Secondary endpoints

A core set of secondary endpoints will be evaluated in each cell or domain:

1. All-cause mortality at 14, 28 and 42 days after platform entry
2. Duration of survival censored at 90 days after platform entry
3. Length of stay of acute index inpatient hospitalisation for those surviving until hospital discharge (**excluding** HITH/COPAT/OPAT/rehab) truncated at 90 days after platform entry. Acute index hospitalisation is defined as a continuous admission to an inpatient healthcare facility where the patient was recruited.
4. Length of stay of total index hospitalisation for those surviving until hospital discharge (**including** HITH/COPAT/OPAT/rehab) truncated at 90 days after platform entry. Total index hospitalisation is defined as a continuous admission to any healthcare facility, including rehabilitation hospitals, and hospital-in-the-home or outpatient parenteral antimicrobial therapy services.
5. Time to being discharged alive from the total index hospitalisation (including HITH/COPAT/OPAT/rehab) truncated at 90 days after platform entry (and all deaths within 90 days will be considered '90 days').
6. Microbiological treatment failure defined as positive sterile site culture for *S. aureus* [of the same silo as the index isolate] between 14 and 90 days after platform entry). A sterile site means any sites deep to the skin and skin structures, including deep visceral and musculoskeletal abscesses that have been obtained in a sterile manner.
7. Diagnosis of new foci between 14 and 90 days after platform entry. The presence of new foci will be determined by the site investigator and can incorporate clinical, radiological, microbiological and pathological findings.
8. *C. difficile* diarrhoea as determined by a clinical laboratory in the 90 days following platform entry for participants ≥2 years of age. This means a stool submitted to a clinical laboratory has tested positive for *C. difficile* toxin or toxin gene.
9. Serious adverse reactions (SARs) in the 90 days following platform entry.

10. Health economic costs as detailed in the cost utility analysis appendix.
11. Proportion of participants who have returned to their usual level of function at day 90 as determined by whether the modified functional bloodstream infection score (FBIS) remained the same or improved between baseline and 90 days after platform entry

    Baseline=best within the 4 weeks prior to platform entry

**Modified FBIS:**

| Rank | Description |
|---|---|
| 4 | Out of hospital; able to complete daily activities without assistance |
| 3 | Out of hospital; unable to complete daily activities without assistance |
| 2 | Out of hospital; significant disability; requires a high level of care and assistance daily (this includes residential aged care) |
| 1 | Hospitalised (or equivalent, such as hospice) |

12. Desirability of outcome ranking 1 (modified Antibiotic Resistance Leadership Group version) at 90 days after platform entry

**Modified ARLG DOOR:**

| **Rank** | **Alive at 90 days** | **How many of:**<br>• **Microbiological treatment failure**<br>• **Infectious Complication***<br>• **Any SAR OR AE leading to study drug discontinuation**** | **QoL** |
|---|---|---|---|
| **1** | Yes | 0 of 3 | Tiebreaker based on the modified FBIS |
| **2** | Yes | 1 of 3 | |
| **3** | Yes | 2 of 3 | |
| **4** | Yes | 3 of 3 | |
| **5** | No | Any | |

*New metastatic focus OR change in antibiotic due to inadequate clinical response. Change in antibiotic due to inadequate clinical response will be determined using only data already collected as part of the backbone (up to day 14) and EOS (up to day 28) domains.

**Any SAR (core 2ry endpoint) OR change in antibiotic due to AE. Change in antibiotic due to adverse event will be determined using only data already collected as part of the backbone (up to day 14) and EOS (up to day 28) domains.

13. Desirability of outcome ranking 2 (SNAP version) at 90 days after platform entry

**SNAP DOOR:**

| **Rank** | **Alive at 90 days** | **Return to usual level of function by day 90** | **At least one SAR or specified safety outcome*** | **QoL** |
|---|---|---|---|---|

| | | | | |
|---|---|---|---|---|
| **1** | Yes | Yes | No | Tiebreaker based on hospital length of index admission (including OPAT/HITH) |
| **2** | Yes | Yes | Yes | |
| **3** | Yes | No | No | |
| **4** | Yes | No | Yes | |
| **5** | No | N/A | N/A | |

*Any SAR and/or one or more of: AKI, new RRT days 1-90, persistent RRT at day 90, CDAD, using definitions and data collected as part of pre-specified core secondary outcome measures.

## 6.9. Control of Bias

### 6.9.1. Randomisation

Once a patient is judged as platform eligible and randomisation occurs via the trial database, the patient has entered the platform. Trial timepoint day 1 is the day of platform entry. Baseline is considered day 1 (there is no day 0).

Randomisation will be conducted through a secure website using a computer-based central randomisation program. Randomisation will occur for all cells and domains; these randomisations are initially concealed and an allocation is only deemed to have occurred if it is subsequently revealed. Sites will receive a participant’s allocation to an intervention within a domain or cell once a participant is confirmed eligible for that domain or cell.

### 6.9.2. Allocation concealment

Allocation concealment will be maintained by using centralised randomisation remote from study sites.

### 6.9.3. Blinding of treatment allocation

Most interventions determined by randomisation in the SNAP trial are anticipated to be provided on an open-label basis, however, blinding of treatment allocation is not precluded for specific domains. If required, procedures to maintain blinding of treatment allocation will be described in the relevant DSA.

### 6.9.4. Blinding of outcome adjudication

The primary endpoint of the SNAP trial (all-cause mortality at 90 days) is not subject to ascertainment bias. Where possible, trial management personnel unaware of treatment allocations will conduct follow-up assessments.

Where secondary endpoints (either generic or domain-specific) are considered to be subjective, blinded outcome adjudication may be required and will be specified in the domain specific appendix as needed.

#### 6.9.5. Follow-up and missing data

Regional trial management personnel will perform timely validation of data, and address queries and corrections. Missing and erroneous data will be minimized through a clear and comprehensive data dictionary with online data entry including logical consistency rules. Common patterns of error will be circulated to all sites. Data management centre study personnel performing site checks will be blind to the study allocations. Imputation may be considered for missing data in any statistical analyses and planned methods will be detailed in the SAP.

# 7. Clinical quality registry

Patients who meet platform inclusion criteria (*Staphylococcus aureus* grown from ≥1 blood culture and admitted to a participating hospital) who are excluded according to core exclusion criteria, or who decline consent for randomisation, may be included in the clinical quality registry associated to the SNAP trial. Eligibility and consent procedures are detailed in the registry appendix. This registry will include data from all patients entering the platform and will operate as a multicentre population-based prospective cohort.

## 7.1. Data Linkage

Participants will be invited to consent to link their data collected with routinely collected data from a range of population databases and registers.

The collection of participant names, date of birth, sex, and address is essential for accurate data linkage. Participant data will be linked to a variety of health variables including information on hospitalisations, emergency department use, and mortality through jurisdictional or national registries.

Linkage will be retrospective, with the time period covered dependent on the properties of the specific data set.

Participants are given the option to opt out of the data linkage component of this study on the Participant Consent Form. Participants not wishing to have their data used in future data linkage studies may still enrol in the main study.

# 8. Trial conduct

## 8.1. Site participation

Sites will be encouraged and supported to commence participation in all domains. If there is a strong local rationale, participation in a subset of domains will be permitted. Site feasibility will be assessed prior to

participation and will take into account number of *S. aureus* bacteraemias per year, availability of an engaged site principal investigator, and local research capacity.

## 8.2. Recruitment of participants

The SNAP trial is designed to be embedded into usual clinical routines at participating sites, such that treatment decisions made by clinical staff can be replaced by randomisation in a streamlined fashion. Although not essential to participation in the trial, sites will be encouraged to develop processes that allow effective embedding of recruitment into normal workflow, with guidance provided by regional committees. The success of embedding of recruitment procedures at each site will be assessed by the proportion of eligible patients successfully randomised, and the timing of randomisation after the achievement of eligibility criteria.

Standard operating procedures will be developed at each site to guide clinical and research staff through the process of screening and randomisation.

## 8.3. Screening

All patients with SAB admitted to hospital will be referred by the pathology laboratory or the treating doctor to the site investigator or their delegate (sub-investigator or properly qualified research nurse) as soon as identified. All patients with SAB should be screened for enrolment via the electronic data capture system, with three groups of patients: 1) Eligible and consenting to platform entry (platform participants); 2) Ineligible to platform entry, or eligible and declining consent to platform entry, but consenting to registry data collection (registry only participants); 3) Ineligible to platform entry, or eligible and declining consent to platform entry, and declining consent to registry data collection (non-trial and non-registry).

The data collected for groups 1, 2 and 3 above will depend on local regulations and will be detailed in region specific appendices. As a general rule, groups 1 and 2 will require sufficient identifiers to allow future follow-up. Group 3 will not have identifiable information collected. If the patient is eligible, the investigator will document this in the medical records unless specifically not required by local policy.

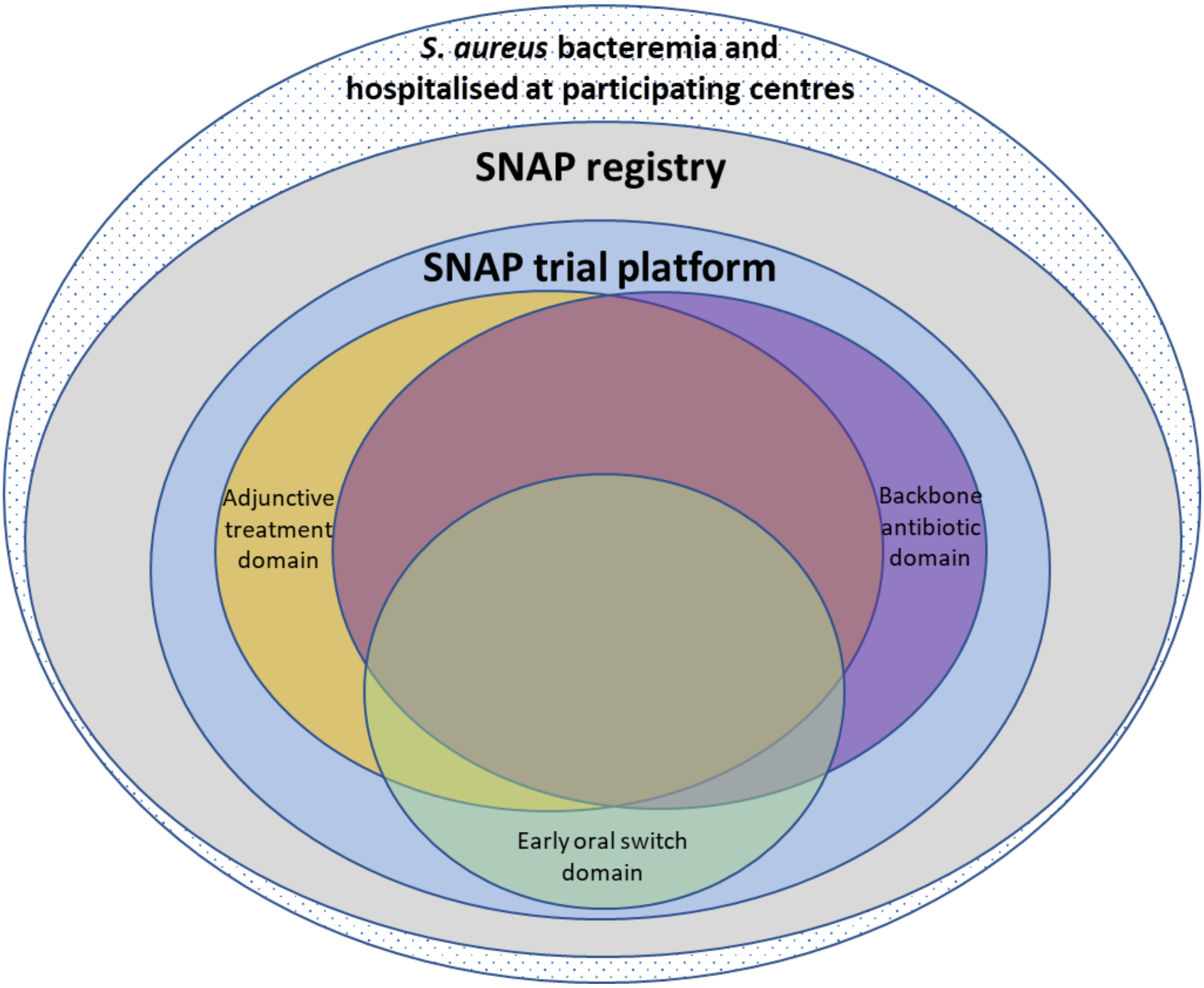


*Figure 4. Patient recruitment: Registry and Platform entry*

## 8.4. Informed Consent

All consent procedures will accord with local jurisdictional requirements. General principles to guide the consent procedures in local jurisdictions are discussed below and further details can be found in the Region-Specific Appendices.

Written informed consent will be obtained from all participants or their surrogate decision makers prior to entry into the platform. Where a potential participant is competent to give consent, they will be directly approached for a consent discussion, covering the whole platform. An interpreter should be utilised where required, and this will be documented on the consent form.

If a participant does not have the capacity to consent to the trial (e.g., due to delirium or sedation), then a surrogate decision maker will be approached for consent / assent if regulatory and legal frameworks allow it in the relevant jurisdiction. Where a surrogate decision maker has provided consent / assent, the participant themselves will be approached to confirm consent (or withdraw) as soon as practicable if they

regain capacity. Capacity to consent will be judged by the site investigator using their clinical expertise and in discussion with the treating clinical team. In the case where the surrogate decision maker is not physically present within the recruitment time frame, the consent discussion can take place over the telephone, and the surrogate decision maker can give verbal consent via the telephone. The person conducting the consent discussion should then document this in the medical record. The surrogate decision maker will then need to sign the consent form as soon as possible afterwards.

If the participant is illiterate, an impartial witness should be present during the entire consent discussion. Once the discussion is complete, the participant must sign and date the informed consent form, if capable. The impartial witness must also sign and date the consent form along with the person who conducted the consent discussion.

Deferred consent strategies may be used in jurisdictions where local regulations permit. This would apply to patients who are severely ill and for whom a surrogate cannot be identified in a timely fashion. Deferred consent would be subject to standard participant or surrogate consent as soon as possible thereafter.

Issues relating to consent and assent of children will be detailed in a Paediatric Specific Appendix.

There will be a single upfront consent process that covers the SNAP platform and all domains. Opt-in consent will be sought for each domain. Individuals may therefore consent to participate in one or more domains.

## 8.5. Treatment allocation

As noted above (see section 6.9.1) randomisation will be performed centrally via a web portal. At platform entry, participants will be randomised for all potential cells and domains. Allocation within a domain or cell will be revealed once a participant is confirmed eligible for that domain or cell (e.g., when a participant's antibiotic susceptibility profile [silo] is determined, or when the participant is deemed eligible for early oral switch at Day 7 or 14). A participant is only deemed to have entered a domain or cell once an allocation has been revealed within that domain or cell.

## 8.6. Delivery of interventions

Specific protocols for the delivery of trial interventions will be outlined in each domain's DSA. Operational documents outlining processes for implementation of interventions will be developed for each site to facilitate protocol adherence. These processes will, wherever possible, reflect usual practices at each site.

## 8.7. Participant timeline

See Figure 3 and Table 2.

*Table 2. Schedule of visits, data collection and follow-up.*

| Visit Day | Day 1 | | | Day 2 | Day 5 (± 1 day) | Day 7 | Day 14 | Day 28 | Day 42 | Acute D/C | Total D/C[1] | Day 90 |
|---|---|---|---|---|---|---|---|---|---|---|---|---|
| Eligibility screening | X | | | | | | | | | | | |
| Informed consent | | X | | | | | | | | | | |
| Randomisation | | | X | | | | | | | | | |
| Collect blood cultures[2] | | | | X | X[3] | | | | | | | |
| Measure creatinine, CRP, ALT and/or GGT[4] | | | X | | | | | | | | | |
| Collect data as per CRFs[5] | | | X | | | X | X | X | X | X | X | X |

[1] Total index hospital discharge (includes HITH/OPAT/COPAT and stepdown inpatient rehabilitation/post-acute care, if continuous with the initial inpatient admission)
[2] Ensure blood culture (BC) is ordered by the treating clinician.
[3] Only required if day 2 BC is positive, or record if done as part of standard of care.
[4] On calendar day 1 or day before
[5] Data will be collected, as per the CRFs, on platform day 1, day 8-10 (for data from platform days 1-7), day 15-18 (for data from platform days 8-14), day 28, day 42, day 90, and acute and total discharge. Domain-specific data collection is detailed in the relevant appendices. Investigators are encouraged to frequently check patient progress and progressively collect data throughout the hospital stay.

## 8.8. Study Daily Visit Details

### 8.8.1. Screening

Screening to evaluate eligibility. Refer to Figure 3.

### 8.8.2. Day 1

These activities will occur on the local calendar day of randomisation.

- Once eligibility has been confirmed and informed consent obtained, the patient will be randomised. The allocated treatment regimen will be initiated as soon as practically possible after domain eligibility confirmed and allocation is revealed.
- Ensure to notify local clinical microbiology laboratory of patient enrolment and need to store index bacterial isolate.
- Collect data as per the CRFs.
- Measure and record serum creatinine, CRP, ALT and/or GGT (on calendar day 1, or record results from the calendar day before platform entry).
- Review of safety events.

### 8.8.3. Day 2

- Ensure blood culture is ordered by the treating clinician.
- Review of safety events.

#### 8.8.4. Day 5 (± 1 day)

- If blood culture was positive on day 2, either ensure blood culture is ordered by the treating clinician or record the result if done as part of standard of care.
- Review of safety events.

#### 8.8.5. Day 7, 14, 28 and 42

- Complete data collection as per the CRFs.

#### 8.8.6. Acute Hospital discharge

- Complete data collection as per the acute discharge CRF. Data will include occurrences of the following during the index hospitalisation:
  Vital status, microbiological treatment failure, metastatic complications, clinical course and management of the infection and results of selected investigations.

#### 8.8.7. Total Hospital discharge

- Complete data collection as per the total discharge CRF. Data will include occurrences of the following during the index hospitalisation:
  Vital status, microbiological treatment failure, metastatic complications, clinical course and management of the infection and results of selected investigations.

#### 8.8.8. Day 90-100

- Ascertain vital status via hospital records, GP and specialist letters, outpatient attendance (including for pathology and radiology).
- If confirmed alive or vital status unable to be confirmed, then contact patient.
- Return to usual level of function and workforce status questions
- Complete data collection as per Day 90 CRF. Data will include occurrences of the following:
  Vital status, microbiological treatment failure, metastatic complications, *C. difficile* diarrhea.

### 8.9. Blinding of allocation status

Most trial interventions are anticipated to be delivered on an open-label basis. For these domains, all members of the GTSC and regional committees will remain blinded to participant allocations and aggregate results until a domain conclusion has been reported by the DSMC.

Interventions delivered in a blinded fashion are not precluded within the trial – the blinding status and the associated processes will be detailed in each domain's DSA. Unblinding of allocation status should only occur when knowledge of the actual interventions received by a patient is necessary for further management. A system for emergency unblinding will be provided in the DSA of domains incorporating blinding of allocation status. These events and the reasons for unblinding will be documented in participants' CRF. Unblinding is not necessarily an indication for study drug discontinuation.

## 8.10. Discontinuation of participation

Trial participants may discontinue their participation from the entire trial based on the criteria presented below, or from individual domains based on criteria detailed in the relevant DSA. Therefore, participants may choose to withdraw from the entire platform, or from specific domains while remaining enrolled in other domains within the platform.

The participants have the right to choose to withdraw from the study at any time and the investigator may discontinue a participant from the study or from treatment if deemed appropriate at any time.

Reasons why a participant may be withdrawn from the entire platform include, but are not limited to:

- participant request (or request by their legal representative)
- determination by the treating clinician that participation in the SNAP trial is no longer in the participant's medical best interests
- participant was enrolled and was later found to be ineligible. For example, if the index blood culture had been mistakenly reported as growing *S. aureus* but was in fact growing a coagulase negative *Staphylococcus*.

Where withdrawal occurs, the reasons will be documented in the participant CRF. All data collected up till the time of withdrawal will remain part of the study data set and included in analyses. Permission for ongoing collection of study data until day 90 will be requested from participants or their representative.

## 8.11. Data collection

### 8.11.1. Principles of data collection

All data will be collected using standardised instruments developed by the GTSC. The CRF will be made available to sites in both electronic (web-based) and paper form for initial data collection, but all data will ultimately be entered onto the electronic form for submission. The electronic CRF (eCRF) will be the official data and the paper CRFs are provided as a tool to facilitate data collection (and thus sites may choose not to use the paper CRFs). Staff collecting data will all undergo training and have access to a comprehensive data dictionary.

Data collected for the trial must accurately reflect the participant's clinical record and be entered in a timely manner.

## 8.12. Data management

### 8.12.1. Source documents

Source documents contain the original record of clinical data, from which data is abstracted to complete the CRF. These documents include but are not limited to hospital records, outpatient and office records, laboratory and pharmacy records, medical images and reports, and clinical correspondence. A further data source will be through telephone conversations with the study participant or GP. If there are data points collected directly from the patient and which are not part of the medical record, then the eCRF will be considered the source data for these data points. Details of any Day 90 follow-up phone calls will be recorded in the eCRF and considered source data.

Required data for the SNAP trial will be entered by site personnel into a web-based portal containing an electronic version of the CRF. It can be transcribed by the PI or their delegate from the paper CRFs onto the eCRF (in no case is the paper CRF to be considered as source data for this trial).

### 8.12.2. Data Integrity

The site PI should ensure the accuracy, completeness and timeliness of the data reported to the sponsor in the eCRFs and in all required reports. Data will be stored in a re-identifiable manner in the database, using a unique screening number for each patient.

The database will contain validation ranges for each variable to minimise the chance of data entry errors. An audit trail will maintain a record of initial entries and changes made, reasons for change, time and date of entry, and username of person who made the change. Data queries will be raised by the project manager and study monitor, and missing data or suspected errors will be raised as data queries and resolved as the trial progresses to enable Bayesian updates on cleaned data. The database will contain in-line capability so that these queries and answers are logged as part of the audit trail.

For each potential participant screened (even those who are found not to be eligible on screening), the screening eCRF will be completed by the site PI or their delegate. For each participant enrolled, eCRFs must be completed. This also applies to records for those patients who fail to complete the study. Access to the web portal will be via site- or investigator-specific passwords.

In addition, accurate and reliable data collection will be assured by verification of the eCRFs against the investigator's records by the study monitor (source document verification) for selected data points as per the regional monitoring plan.

The quality of data collected in the SNAP trial will be supported by a number of mechanisms, including:

1. Start-up meetings for new sites
2. Training of staff responsible for data collection

3. Availability of a detailed data dictionary at all sites at the point of data collection
4. In-built data checking and validation steps in the electronic CRF
5. Regular data validation performed by the central data management centre

### 8.12.3. Confidentiality

All trial data will be stored in a secure fashion, accessible only to site personnel and trial staff via passwords. On all trial documents other than the signed participant consent form, participants will be referred to using a trial identification number. Information linking participants' medical records to trial data will only be available to site personnel and each region's central study personnel and will not be made available to members of the regional committee, DSMC or GTSC. No identifiable data will leave a participating country. Trial data will be held in confidence by all site and trial staff.

Authorised representatives of the sponsoring institution may inspect all documents and records required to be maintained by the Investigator, including but not limited to, medical records (office, clinic or hospital) and pharmacy records for the participants in this trial. The clinical trial site will permit access to such records.

Storage and archiving of hard-copy study documents (e.g., consent forms) will be the responsibility of the PI at each site and will remain at the site of recruitment following local security guidelines. Hard-copy study documents will be kept for a minimum of 15 years and confidentially destroyed at the end of this period only with the express consent of the study sponsor.

### 8.12.4. Access to Data

Access to data will be granted to authorised representatives of the GTSC, sponsors, and regulatory authorities for the purpose of trial monitoring. Regional committees will ensure that the trial also complies with all relevant regional regulatory and academic requirements.

Issues relating to access to data where a commercial organisation is involved in the trial will be set out in a contract between trial sponsors and the commercial organisation. These contracts will only be entered into providing that they guarantee academic independence with regards to the design and conduct of the trial (including analysis and reports), and that all data are owned by the trial sponsor.

### 8.12.5. Dissemination Policy

The trial results will be communicated to all site investigators prior to publication or presentation. The trial results will be submitted for presentation at national and international meetings and publications submitted to peer reviewed scientific journals, irrespective of the results. The GTSC may decide to

communicate results via media releases and pre-print submissions. A plain-language summary of the trial results will be made publicly available, and available on the SNAP website for all participants to access. Authorship of each publication will be determined by the GTSC and be guided by ICJME criteria for authorship. The site PI for hospitals contributing at least one case for analysis will be included as an author. Additional authors will be considered from trial sites that recruit a large number of participants. These individuals may be different from a centre's member of the GTSC. The SNAP study group will consist of all named site investigators, members of working groups relevant to the publication at hand, and members of the GTSC. These SNAP study group members will be listed in the collaborators section of the paper. The author by-line will include 'for the ASID Clinical Research Network' and other participating regional research networks as appropriate.

## 8.13. Quality assurance and monitoring

The trial will be conducted in accordance with the current approved protocol, Good Clinical Practice guidelines, relevant local law and regulations, and established trial standard operating procedures.

### 8.13.1. Plans for ensuring protocol adherence

*Monitoring*

Monitoring of site processes and adherence to the trial protocol will be the responsibility of regional committees. Committee members or regional project managers will undertake regular monitoring of sites through site visits, telephone or email contact, and review of data monitoring as described below. Monitoring reports will be prepared on a regular basis, as determined by recruitment rates at each site, and reviewed by the regional committee. Follow-up letters will be sent to site coordinators and retained in the investigator file.

*Principal Investigator (PI) training*

All site PIs will be trained in the study protocol, SOPs and their reporting requirements by the project manager, or a study chief investigator, prior to the site being opened for recruitment. All site PIs should complete an accredited Good Clinical Practice training course.

The project manager or their delegate will have regular contact with all enrolling site investigators.

*Documentation in patient's medical record and bedside chart*

A participant's involvement in SNAP will be documented in their medical record. This will alert clinicians that the patient has been enrolled in the SNAP trial with a brief explanation of the study.

If there is a bedside chart, a copy of the study synopsis will be placed in the bedside chart of the patient.

*Checking of drug charts*

The medication chart (be it paper or electronic) will be checked regularly by the PI or their delegate as long as the patient remains on at least one protocol-determined pharmaceutical intervention, and whilst they remain an inpatient to ensure adherence to the study protocol.

### 8.13.2. Protocol Deviations and Serious Breaches

The following protocol deviations will be documented and reported in the eCRF for this study:

- Incorrect study drug prescribed or administered
- Day 90 outcomes missing after Day 100

A serious breach of GCP or the protocol is a breach that is likely to affect to a significant degree: a) The safety or rights of a trial participant, or b) The reliability and robustness of the data generated in the clinical trial.

As a pragmatic study, it is unlikely that any serious breaches will occur. However, if a suspected serious breach is identified at the site, the PI or their delegate should e-mail details to the regional sponsor within 72 hours of becoming aware of the suspected breach. The regional sponsor will then assess the report. Whether a reported breach meets the definition of a serious breach will depend on many factors. For example, where the breach significantly impacts on the quality of key analysis parameters and excluding those data from the analysis significantly impacts the trial, a serious breach may be confirmed. The regional sponsor will report all serious breaches to the ethics board / institutional review board and the global coordination trial office in a timely manner, typically within 7 days (depending on regional requirements) and conduct a root cause analysis and implement any corrective and preventative actions. The PI or their delegate should report any confirmed serious breach to their governance office within 72 hours.

Where protocol deviations or serious breaches identify protocol-related issues, the protocol will be reviewed and, where indicated, amended.

## 9. Principles of statistical analysis

A detailed description of the Bayesian data analysis and associated decision criteria for trial adaptations used in the SNAP trial is located in the Statistical Appendix. The following section serves as an introduction and lay summary of these methods.

## 9.1. Introduction

The SNAP trial will repeatedly fit Bayesian hierarchical models to the accumulating data (updates), over the life of the trial, to estimate model parameters and evaluate pre-specified decision criteria that determine whether an intervention is superior, inferior or non-inferior to comparator or control interventions within that cell or domain. Within this design, inferences are made using pre-specified statistical models incorporating non-intervention variables that may influence the probability of the primary endpoint, as well as intervention variables across all domains and biologically plausible interactions. When a decision threshold is met for a cell or the domain as a whole, demonstrating non-inferiority, superiority or futility, then recruitment to that domain within that silo, or potentially across all silos, may be stopped. The life of a domain is defined as the period from the start of recruitment to the domain until either decision criteria are satisfied for all silos or trial resources are exhausted.

Threshold probabilities for superiority, inferiority or non-inferiority for the primary endpoint are pre-defined based on pre-trial simulations. Estimated decision quantities, from the statistical models, that return probabilities at or above these thresholds (sometimes termed "Statistical Triggers"), for a cell or a domain as a whole, will result in a report being prepared for the DSMC by the Analytic Team. The DSMC, after reviewing the data and its context will recommend whether the Statistical Trigger should result in declaration of a domain conclusion, which may result in public disclosure and appropriate modification of the interventions available for allocation to participants enrolled in the SNAP platform.

## 9.2. Bayesian statistical modelling

As noted above, a Bayesian hierarchical model of the primary endpoint and decision criteria will be used to assess Statistical Triggers within a cell or a domain, based on data accrued at each analysis (Bayesian update) and any pre-trial knowledge (the prior distribution, as specified in the Statistical Appendix). The estimates of intervention effects will be provided by the main effects from the primary model. Complete specification of the hierarchical models for primary and secondary endpoints can be found in the Statistical Appendix.

The primary Bayesian hierarchical model will be run repeatedly during the life of the trial, based on the accumulating number of participants with available 90-day outcomes (Bayesian updates). Participants dying prior to 90 days after randomisation will only be included in analyses conducted more than 90 days after their randomisation to prevent biases arising from differential outcome timing. The frequency and timing of the Bayesian updates is documented in the Statistical Appendix.

## 9.3. Statistical handling of ineligible patients

Participants fulfilling the entry criteria for the SNAP trial may be ineligible for one or more interventions or domains. In addition, some domains or interventions may be unavailable in particular regions. Where a participant is ineligible for a domain (for example, due to contraindication) an indicator variable will be created for each domain and included in the primary and secondary Bayesian hierarchical models (further details available in Statistical Appendix).

## 9.4. Superiority statistical trigger

At a Bayesian update, if any intervention within a domain has met the decision criteria for being superior for the domain as a whole or for a cell, the Analytic Team will prepare a report for the DSMC to consider whether to recommend to the GTSC the declaration of a domain conclusion that the intervention is superior to the comparator or control intervention in the cell or domain.

## 9.5. Inferiority statistical trigger

At a Bayesian update, if any intervention within a domain has fallen below the decision threshold for being inferior for the domain as a whole or for a cell, the Analytic Team will prepare a report for the DSMC to consider whether to recommend to the GTSC the declaration of a domain conclusion that the intervention is inferior to the comparator or control intervention in the cell or domain and whether the intervention should be dropped. Inferiority statistical triggers only apply to domains with more than two interventions.

## 9.6. Non-inferiority statistical trigger

At a Bayesian update, if any intervention within a domain has met the decision threshold for being non-inferior (i.e., below a pre-specified clinical minimally important difference) for the primary endpoint compared to the comparator or control intervention for the domain as a whole or for a cell, then the Analytic Team will prepare a report for the DSMC to consider whether to recommend the declaration of a domain conclusion that the intervention is non-inferior to the comparator or control intervention. Non-inferiority statistical triggers only apply to domains with two interventions, and the choice of intervention nominated as the non-control intervention will be pre-specified in the DSA.

## 9.7. Domain futility trigger

At each Bayesian update, an assessment of futility will be made, at the cell or domain level. For cells that evaluate superiority, the futility trigger for an intervention is considered to be met if the probability of superiority is below a pre-specified threshold. For cells that evaluate non-inferiority, the futility trigger for

an intervention is met if the probability of a clinically meaningful effect, relative to the comparator or control intervention, is below a pre-specified threshold (further details in Statistical Appendix).

When a futility trigger is met, this result will be communicated to the GTSC by the DSMC. The GTSC, in conjunction with the DSMC, will undertake to consider secondary endpoints, health economic implications, and potential intervention-intervention interactions to inform a decision on whether randomisation within the domain should continue and for how long. If randomisation is ceased, the GTSC will take responsibility to undertake a Public Disclosure as soon as practicable through presentations and/or publication unless a delay is recommended by the DSMC for the purpose of further data collection.

## 9.8. Action when a Statistical Trigger is achieved

If a Statistical Trigger (defined in sections 9.4-9.7) is achieved at a Bayesian update, the Analytic Team will prepare a report for the DSMC, who will oversee the following actions and make recommendations to the GTSC.

If a Statistical Trigger for superiority is judged to have been validly achieved, the DSMC may recommend declaring a domain conclusion. Once a domain conclusion has been declared, then randomisation to all comparator or control interventions within the domain overall or cell will be halted at sites where the superior intervention is available. Randomisation to comparator or control interventions may continue at sites where the superior intervention is not available and is not anticipated to become available in a reasonable timeframe as a result of the domain conclusion. At sites where the superior intervention is available, the domain or cell will continue with all participants recommended the superior intervention (i.e., although this is not anticipated to be a 100% randomization probability), pending the addition of any new interventions to be compared to the current superior intervention. After the declaration of a domain conclusion for superiority, the GTSC will take responsibility to undertake a Public Disclosure as soon as practicable through presentations and/or publication unless a delay is recommended by the DSMC for the purpose of further data collection.

If a Statistical Trigger for inferiority is judged to have been validly achieved, the DSMC will recommend declaring a domain conclusion. Once the domain conclusion has been declared then randomisation to the inferior intervention will be recommended to be halted. After the declaration of a domain conclusion for inferiority, the GTSC will take responsibility to undertake a Public Disclosure as soon as practicable through presentations and/or publication unless a delay is recommended by the DSMC for the purpose of further data collection. Where necessary, an alternative comparator regimen may be nominated by the GTSC for the purpose of futility assessments.

If a Statistical Trigger for non-inferiority is judged to have been validly achieved, this result will be communicated to the GTSC by the DSMC. The GTSC, in conjunction with the DSMC, will undertake consideration of secondary endpoints, health economic implications, and potential intervention-intervention interactions to inform a decision on whether randomisation should continue within the domain and for how long. These considerations may include an analysis of the likelihood of superiority. If randomisation is ceased, the GTSC will take responsibility to undertake a Public Disclosure as soon as practicable through presentations and/or publication unless a delay is recommended by the DSMC for the purpose of further data collection.

## 9.9. Analysis set for reporting

At the time of a domain conclusion, participants may have been randomised but not yet included in the analysis of the primary endpoint because they have not yet completed 90 days of follow up or because data for a participant who has completed 90 days of follow up has not yet been submitted. When a Public Disclosure is made, the Bayesian update resulting in the Platform Declaration and, if requested, secondary analyses including all evaluable randomised participants will be presented.

## 9.10. Simulations and statistical power

The design of the trial and its domains have been informed by the results of extensive Monte Carlo simulations, which are summarised in terms of trial operating characteristics, including type 1 error and statistical power, in the Statistical Appendix. These simulations will be updated each time a new domain is added to the platform, or where an intervention is added to a domain (but not where an intervention is removed). The results of simulations will be maintained as a publicly available operational document on the study website and updated as required.

## 9.11. Co-enrolment with other trials

Co-enrolment of participants in other research studies or trials is encouraged, except where there is a clear threat to the validity of either trial or where co-enrolment would materially increase the risk to participants. Decisions regarding the appropriateness of co-enrolment of SNAP participants, will be made on a trial-by-trial basis by the GTSC for trials conducted in more than one region participating in SNAP, and by the RTSC where the trial is being conducted in a single region. Cooperation between complementary trials and SNAP is also encouraged and may include sharing of recruitment and data collection infrastructure and sharing of data including allocation status. Decisions regarding co-enrolment and cooperation with other trials will be distributed to participating sites as operational documents.

Where co-enrolment with another trial is not appropriate, each site will need to establish rules that determine circumstances in which each trial has preferential recruitment.

### 9.12. Criteria for termination of the trial

Although the SNAP trial is designed as a platform allowing for perpetual recruitment and randomisation within domains that will evolve over time, the trial may be terminated if funding or other supports are no longer available, SAB is no longer deemed a problem of public health significance, or no new plausible interventions are available to test for effectiveness or cost-effectiveness.

Should the SNAP trial be terminated, the end date of the trial is the day of final follow-up for the last recruited participant.

## 10. Safety monitoring and reporting

Patients eligible for the SNAP trial are at significant risk of morbidity or mortality from SAB and its complications, regardless of their participation in the trial. Many such events would meet the conventional definitions of a serious adverse event (SAE). The strategy used for the reporting and attribution of SAEs in this trial aims to ensure that the safety and rights of participants are protected while recognising that many apparent SAEs will relate to the clinical course of patients with a severe infectious illness.

### 10.1. Definitions

| Term | Description |
| --- | --- |
| Adverse Event (AE) | Any untoward medical occurrence in a patient/trial participant administered a medicinal product, and which does not necessarily have a causal relationship with this treatment.<br><br>*An adverse event can therefore be any unfavourable and unintended sign (including an abnormal laboratory finding), symptom, or disease temporally associated with the use of a medicinal product, whether or not related to the product.* |
| Adverse Reaction (AR) | Any untoward and unintended response to a medicinal product related to any dose administered.<br><br>**Comment**: All adverse events judged by either the reporting investigator or the sponsor as having a reasonable causal relationship to a medicinal product would qualify as adverse reactions. The expression 'reasonable causal relationship' means to convey, in general, that there is evidence or argument to suggest a causal relationship. |
| Unexpected Adverse Reaction (UAR) | An adverse reaction, the nature or severity of which is not consistent with the Reference Safety Information (RSI).<br><br>**Note:** The RSI should be contained in the investigator's brochure for an unapproved medicinal product or Product Information (or another country's equivalent of the Product Information) for an approved medicinal product. |

| | |
|---|---|
| Reference Safety Information (RSI) | The information contained in an approved Australian Product Information (or other country equivalent) that contains the information used to determine what adverse reactions are to be considered expected adverse reactions and, on the frequency and nature of those adverse reactions. |
| Serious Adverse Event (SAE) | An SAE is any adverse event that:<br>○ Results in death<br>○ Is life-threatening<br>*The term "life threatening" refers to an event in which the participant was at risk of death at the time of the event. It does not refer to an event, which hypothetically may have caused death, if it were more serious.*<br>○ Results in unexpected prolongation of existing hospitalisation<br>○ Results in persistent or significant disability/incapacity<br>○ Is a medically important event or reaction<br>***Note**: Medical and scientific judgement should be exercised in deciding whether an adverse event/reaction should be classified as serious in other situations. Important medical events that are not immediately life-threatening or do not result in death or hospitalisation but may jeopardise the participant or may require intervention to prevent one of the other outcomes listed in the definition above should also be considered serious.* |
| Serious adverse reaction (SAR) | Any SAE that is suspected to be related to a medicinal product used for *S. aureus* bacteraemia (i.e., there is a reasonable causal relationship with that medicinal product). In other words, this is an event which qualifies as both a serious adverse event AND an adverse reaction. |
| Suspected Unexpected Serious Adverse Reaction (SUSAR) | Any SAE that is both unexpected (i.e. its nature or severity is not consistent with the Approved Product Information) and suspected to be related to the medicinal product used for *S. aureus* bacteraemia (i.e. there is a reasonable causal relationship with that medicinal product). |
| Significant Safety Issue (SSI) | A safety issue that could adversely affect the safety of participants or materially impact on the continued ethical acceptability or conduct of the trial. |
| Urgent Safety Measure (USM) | A measure required to be taken to eliminate an immediate hazard to a participant's health or safety. (A subset of significant safety issues).<br>**Note**: This type of significant safety issue can be instigated by either the investigator or sponsor and can be implemented before seeking approval from relevant ethics committees or institutions. |

## 10.2. Assessment of Adverse Events (AEs)

Each adverse event must be evaluated for:

1) **Seriousness**: An assessment of whether the AE meets the definition of a Serious Adverse Event (SAE).
2) **Causality (relatedness):** A clinical assessment of whether there is a reasonable causal relationship between the AE and the trial treatment.

   The PI (or medically qualified delegate) will make a judgement as to whether an AE has a *reasonable causal relationship* with the allocated treatment(s). The degree of certainty with which an AE is attributable to treatment or an alternative cause will be determined by how well the event can be understood in terms of:

- Temporal relationship with the administration of the treatment or cessation of treatment
- Reactions of a similar nature previously observed in the individual or others following treatment

The PI or delegate's opinion of the relationship between the AE and the trial treatment will be specified as follows:

| | |
|---|---|
| *Not related* | There is not a causal relationship. |
| *Unlikely* | The temporal association between treatment and the adverse event is such that treatment is not likely to have any reasonable association. |
| *Possibly* | The AE could have been caused by treatment. |
| *Probably* | The AE follows a temporal sequence from the time of treatment and cannot be reasonably explained by the known characteristics of the participant's clinical presentation/history. |
| *Definitely* | The AE follows a reasonable temporal sequence from the time of treatment or reappears when the treatment is repeated. |

3) **Expectedness:** An assessment against the AEs/SAEs listed in the trial's Reference Safety Information (the relevant Production Information) as expected occurrences (considering the nature and frequency of the event).

## 10.3. Recording

Adverse events anticipated to occur as a result of trial interventions will, in general, be included as secondary safety endpoints (see section 6.8.2). Where required, domain-specific endpoints will be included in DSAs to capture additional adverse events that may be specifically associated with a particular intervention.

Events will only be recorded if they are attributable to one or more study interventions (SARs; attributable to a protocol-determined drug or strategy). The treating clinical team will be asked to notify the local study team of any potential SARs, and the local study team will also perform a weekly review of the medical records for SARs occurring during the preceding period while the participant is in hospital, at discharge, and then at platform days 14, 28, 42 and 90. Data should be recorded as per the eCRFs.

## 10.4. Reporting

SARs do not require expedited reporting to the sponsor. All SARs will be reviewed periodically by the DSMC and will be reported to the reviewing ethics committee in the annual report.

Events meeting the definition of a SUSAR or a SSI must be reported to the trial management group and regional trial sponsor within 24 hours of site personnel becoming aware of the event, unless otherwise specified in a RSA.

The site will report the SUSAR or SSI by completing the Safety Reporting eCRF on the database as soon as possible. The minimum information will comprise the participant's trial identification number, the date of the event, the nature of the event and the reason for its attribution to a trial intervention, and the consequent clinical management. SUSARs should be followed up until the event has resolved or a final outcome has been reached or the participant reaches 90 day follow up. Any change of condition or other follow-up information for the SUSAR should be updated on the safety reporting eCRF as soon as it is available.

### 10.4.1. Site Responsibilities

The PI or their delegate should:

a. Assess all AEs
b. Ensure SARs are captured within the CRFs
c. For SARs that are determined to be SUSARs or any SSIs please ensure the following processes are followed:
    - report to the regional trial sponsor via the trial management group within 24 hours of becoming aware of the event. This occurs when the online Safety Report eCRF is submitted, if this is delayed, please email a written CRF to snap-trial@unimelb.edu.au.
    - report all SUSARs or SSIs occurring from platform entry up until 30 days post last dose of the intervention, or trial day 90, whichever is earlier.
    - report any occurrences of congenital anomaly/birth defect arising from any pregnancy of a participant (or partner). See pregnancy appendix for details.
d. Review of all safety communications from the sponsor (e.g. significant safety issues identified by the DSMC) and ensure any implications for trial participants are managed appropriately.
e. Report to their local governance office, within 24 hours of becoming aware of the event: i) all significant safety issues reported to the site by the sponsor and ii) any SUSARs arising from the local site (if required by local governance).

### 10.4.2. SAEs/SARs not needing expedited reporting

Sites are not required to report SARs or SAEs to the trial management team or sponsor. Only SUSARs are required to be reported. If required, there may be specific regional requirements for reporting of SAEs/SARs to regional sponsors. Such requirements will be detailed in the RSAs.

### 10.4.3. Sponsor Reporting Procedures

All SARs assigned by the site (or following central review) as both related to study treatment and unexpected will be classified as SUSARs and are subject to expedited reporting to the relevant medicines regulatory body.

The Sponsor will report all Significant Safety Issues (SSIs)* to sites (as well as the reviewing ethics committee and the regulatory body):

- SSIs that meet the definition of an urgent safety measure (USM) within 72 hours of becoming aware of the issue.
- All other SSIs within 15 calendar days or becoming aware of the issue.

** SSIs result in a change – either to the protocol (amendment) or a temporary or permanent halt to the trial comparison arm. SSIs may be single case events (e.g. certain SUSARs) or events that arise from an aggregate analysis of safety reports (e.g. increases in frequency or severity of known events). The sponsor will action all SSIs in accordance with the NHMRC Guidance.*

# 11. Governance and ethical issues

## 11.1. Management of participating sites and trial coordination

The primary responsibility for the management of sites, and the monitoring and coordination of trial process will rest with each region's steering committee. The processes by which each regional committee will discharge this responsibility are set out in the relevant RSA.

## 11.2. Ethics and regulatory issues

### 11.2.1. Overarching principals

The study will be conducted according to the Declaration of Helsinki, the principles of Good Clinical Practice (27), and in accordance with all relevant local ethical, regulatory, and legal requirements as specified in each RSA.

### 11.2.2. Approvals

Ethical approval will be sought from a properly constituted and accredited human research ethics committee (HREC) or institutional review board (IRB) for all trial sites. Where possible, a single lead committee will provide approval for all sites under its purview. Local site-specific approvals will also be sought where required by local regulations.

The study protocol, domain specific appendices, participant information materials, consent forms, and any other documents required for ethics approval will be submitted to the relevant HRECs for approval before the study commences. Approvals must specify the study title, version numbers, and identify all documents reviewed and state the date of review. No amendments to, or deviations from, the protocol must be initiated without prior written approval from the relevant HREC. The exceptions to this are:

- Administrative aspects that have no bearing on participants
- The need to address regulatory requirements; and/or
- The need to eliminate immediate hazards to the participants

The relevant HREC(s) will be notified of the following:

- All protocol amendments, informed consent changes or revisions of other documents originally submitted for review
- SUSARs and SSIs
- New information that may affect the safety of the participants or the proper conduct of the trial
- Annual updates of study progress
- Termination of the study including provision of a final study report.

## 11.3. Protocol modifications- Substantial amendments

A substantial amendment to either the Core Protocol, DSA or RSA is one which is likely to meaningfully affect:

- The safety of participants in the trial
- The scientific value of the trial
- The conduct of the trial
- The initiation or cessation of any intervention or domain for any reason

All substantial amendments to the original approved documents will be submitted for approval to all relevant ethical and regulatory review bodies required for the original approvals. Non-substantial amendments will be recorded and filed by the GTSC.

## 11.4. Declarations of interest

All members of trial committees will be required to maintain a register of interests that may influence, or be seen to influence, the conduct of the trial of the interpretations of its results. This register will be a publicly available document published on the study website. The register will be updated in each calendar year.

## 11.5. Communication

Each participating site will comply with all local reporting requirements, as specified by relevant local institution and regulatory authorities.

In the event of cessation of a domain for any reason, including through declaration of a domain conclusion, or of the entire trial, all relevant local and regulatory authorities will be notified within 90 days.

### 11.5.1. Communication of trial results and publication policy

Abstracts and manuscripts (including pre-prints) reporting results or other data from the SNAP trial will be prepared by a writing committee formed by the relevant trial committee(s) and/or working group(s) and the GTSC and must be approved by the GTSC prior to submission. Prior to public release, interim or final results will not be publicised, including in oral presentations without permission from the GTSC.

### 11.5.2. Authorship policy

Criteria for authorship for manuscripts arising will be those of the International Committee of Medical Journal Editors (ICJME). Specifically, authors should satisfy:

- Substantial contributions to the conception or design of the work; or the acquisition, analysis, or interpretation of data for the work; AND
- Drafting the work or revising it critically for important intellectual content; AND
- Final approval of the version to be published; AND
- Agreement to be accountable for all aspects of the work in ensuring that questions related to the accuracy or integrity of any part of the work are appropriately investigated and resolved.

The author by-line will include: 'the *Staphylococcus aureus* Network Adaptive Platform (SNAP) study group'. An updated database of members of the broader SNAP study group will be maintained and where appropriate the list of members will be provided as an appendix to submitted manuscripts.

The author by-line will include the key participating regional clinical trials networks. All sources of funding will be acknowledged.

# 12. References


1. Tong SY, Davis JS, Eichenberger E, Holland TL, Fowler VG, Jr. Staphylococcus aureus infections: epidemiology, pathophysiology, clinical manifestations, and management. Clinical microbiology reviews. 2015;28(3):603-61.
2. van Hal SJ, Jensen SO, Vaska VL, Espedido BA, Paterson DL, Gosbell IB. Predictors of mortality in Staphylococcus aureus Bacteremia. Clinical microbiology reviews. 2012;25(2):362-86.
3. Coombs GW, Daley DA, Lee YT, Pang S, Bell JM, Turnidge JD, et al. Australian Group on Antimicrobial Resistance (AGAR) Australian Staphylococcus aureus Sepsis Outcome Programme (ASSOP) Annual Report 2015. Commun Dis Intell (2018). 2018;42.
4. England PH. Annual epidemiological commentary: Gram-negative, MRSA and MSSA bacteraemia and C. difficile infection data, up to and including financial year April 2018 to March 2019. 2019.
5. Thwaites GE, Scarborough M, Szubert A, Nsutebu E, Tilley R, Greig J, et al. Adjunctive rifampicin for Staphylococcus aureus bacteraemia (ARREST): a multicentre, randomised, double-blind, placebo-controlled trial. Lancet. 2018;391(10121):668-78.
6. Thwaites GE, Scarborough M, Szubert A, Saramago Goncalves P, Soares M, Bostock J, et al. Adjunctive rifampicin to reduce early mortality from Staphylococcus aureus bacteraemia: the ARREST RCT. Health Technol Assess. 2018;22(59):1-148.
7. Ministry of Health Israel. National Center for Infection Control. Annual report of resistant pathogens in general hospitals 2019. Updated 24-May-2020. . 2020.
8. Yahav D, Yassin S, Shaked H, Goldberg E, Bishara J, Paul M, et al. Risk factors for long-term mortality of Staphylococcus aureus bacteremia. Eur J Clin Microbiol Infect Dis. 2016;35(5):785-90.
9. Cai Y, Venkatachalam I, Tee NW, Tan TY, Kurup A, Wong SY, et al. Prevalence of Healthcare-Associated Infections and Antimicrobial Use Among Adult Inpatients in Singapore Acute-Care Hospitals: Results From the First National Point Prevalence Survey. Clin Infect Dis. 2017;64(suppl_2):S61-S7.
10. Pada SK, Ding Y, Ling ML, Hsu LY, Earnest A, Lee TE, et al. Economic and clinical impact of nosocomial meticillin-resistant Staphylococcus aureus infections in Singapore: a matched case-control study. The Journal of hospital infection. 2011;78(1):36-40.
11. McMullan BJ, Bowen A, Blyth CC, Van Hal S, Korman TM, Buttery J, et al. Epidemiology and Mortality of Staphylococcus aureus Bacteremia in Australian and New Zealand Children. JAMA Pediatr. 2016;170(10):979-86.
12. Paterson DL, Steering Group of the Australasian Society for Infectious Diseases Clinical Research N. Determining research priorities for clinician-initiated trials in infectious diseases. The Medical journal of Australia. 2013;198(5):270-2.
13. Holland TL, Raad I, Boucher HW, Anderson DJ, Cosgrove SE, Aycock PS, et al. Effect of Algorithm-Based Therapy vs Usual Care on Clinical Success and Serious Adverse Events in Patients with Staphylococcal Bacteremia: A Randomized Clinical Trial. JAMA. 2018;320(12):1249-58.
14. Fowler VG, Jr., Boucher HW, Corey GR, Abrutyn E, Karchmer AW, Rupp ME, et al. Daptomycin versus standard therapy for bacteremia and endocarditis caused by Staphylococcus aureus. The New England journal of medicine. 2006;355(7):653-65.
15. Thwaites GE, Edgeworth JD, Gkrania-Klotsas E, Kirby A, Tilley R, Torok ME, et al. Clinical management of Staphylococcus aureus bacteraemia. Lancet Infect Dis. 2011;11(3):208-22.

16. Tong SYC, Campbell A, Bowen AC, Davis JS. A Survey of Infectious Diseases and Microbiology Clinicians in Australia and New Zealand About the Management of Staphylococcus aureus Bacteremia. Clin Infect Dis. 2019;69(10):1835-6.
17. Baddour LM, Wilson WR, Bayer AS, Fowler VG, Jr., Tleyjeh IM, Rybak MJ, et al. Infective Endocarditis in Adults: Diagnosis, Antimicrobial Therapy, and Management of Complications: A Scientific Statement for Healthcare Professionals From the American Heart Association. Circulation. 2015;132(15):1435-86.
18. Habib G, Lancellotti P, Antunes MJ, Bongiorni MG, Casalta JP, Del Zotti F, et al. 2015 ESC Guidelines for the management of infective endocarditis: The Task Force for the Management of Infective Endocarditis of the European Society of Cardiology (ESC). Endorsed by: European Association for Cardio-Thoracic Surgery (EACTS), the European Association of Nuclear Medicine (EANM). Eur Heart J. 2015;36(44):3075-128.
19. Antibiotic Expert Group. Therapeutic guidelines: Antibiotic. Version 16, 2019. Melbourne (VIC): Therapeutic Guidelines Limited. 2019.
20. Goto M, Schweizer ML, Vaughan-Sarrazin MS, Perencevich EN, Livorsi DJ, Diekema DJ, et al. Association of Evidence-Based Care Processes With Mortality in Staphylococcus aureus Bacteremia at Veterans Health Administration Hospitals, 2003-2014. JAMA internal medicine. 2017;177(10):1489-97.
21. Berry SM, Connor JT, Lewis RJ. The platform trial: an efficient strategy for evaluating multiple treatments. JAMA. 2015;313(16):1619-20.
22. Saville BR, Berry SM. Efficiencies of platform clinical trials: A vision of the future. Clin Trials. 2016;13(3):358-66.
23. Bhatt DL, Mehta C. Adaptive Designs for Clinical Trials. The New England journal of medicine. 2016;375(1):65-74.
24. Park JW, Liu MC, Yee D, Yau C, van 't Veer LJ, Symmans WF, et al. Adaptive Randomization of Neratinib in Early Breast Cancer. The New England journal of medicine. 2016;375(1):11-22.
25. McGlothlin AE, Viele K. Bayesian Hierarchical Models. JAMA. 2018;320(22):2365-6.
26. Ambasta A, Pancic S, Wong BM, Lee T, McCaughey D, Ma IWY. Expert Recommendations on Frequency of Utilization of Common Laboratory Tests in Medical Inpatients: a Canadian Consensus Study. J Gen Intern Med. 2019;34(12):2786-95.
27. ICH Harmonised Tripartite Guideline: Guideline for Good Clinical Practice. Journal of postgraduate medicine. 2001;47(3):199-203.
