# Supplementary material for: The Staphylococcus aureus Network Adaptive Platform Trial Protocol: New Tools for an Old Foe
Source: Clin Infect Dis. 2022 Jun 19;75(11):2027–34. doi: 10.1093/cid/ciac476 (PMC9710697; doi:10.1093/cid/ciac476)
Supplement: ciac476_Supplementary_Data [file ciac476_supplementary_data.zip › SNAP EOS Domain-Specific Appendix.pdf]

Domain-Specific Appendix:

Early Oral Switch Domain

Staphylococcus aureus **Network Adaptive Platform trial (SNAP)**

Early Oral Switch Domain-Specific Appendix Version 1.0 dated 01 June 2021

Summary

In this domain of the SNAP trial, eligible participants with *Staphylococcus aureus* bacteraemia (SAB) admitted to participating hospitals will be randomised to receive one of two interventions:

- Switch to oral antibiotic treatment at platform **Day 7** (+/- 2 days) OR platform **Day 14** (+/-2 days) ('early oral switch')
- Continued intravenous antibiotic treatment ('continued IV treatment') – the current standard of care

At this participating site the following interventions have been selected within this domain:

☐Early oral switch

☐Continued IV treatment

**SNAP: Early Oral Switch Domain Summary**	
Interventions	● Early oral switch<br>● Continued intravenous (IV) treatment
Silos, domains, and cells	Platform eligible participants within the MSSA, PSSA, or MRSA silos in the early oral switch domain intervention will be analysed. Domain-eligible participants in all three silos will be randomised equally to either an early oral switch or continued IV antibiotic treatment strategy. The population parameters in each 'cell' (a combination of silo and a domain) will be estimated using Bayesian hierarchical models that allow information about the treatment effects in one silo to inform inference in the other silos within the same domain (i.e. information borrowing).
Evaluable treatment-by-treatment interactions	Treatment-treatment interactions are considered unlikely between this domain and other domains and will not be incorporated into the statistical models used to compare interventions in this domain.
Randomisation	Participants will be randomised at platform entry in a fixed 1:1 ratio across the domain. A participant's allocated intervention will be revealed at the time that domain-specific eligibility criteria are met at either platform day 7 (+/- 2 days) or platform day 14 (+/- 2 days). Participants judged to be not eligible at Day 7 will be reassessed at Day 14. Response adaptive randomisation may be applied if additional interventions within this domain are included in future versions of this DSA.
Domain Specific Inclusions	Domain-specific inclusion criteria are:<br>When judging eligibility for early oral switch at platform **Day 7** (+/- 2 days):<br>● Clearance of SAB by platform Day 2: blood cultures negative for *S. aureus* on platform day 2, AND no known subsequent positive blood cultures<br>● Afebrile (<37.8°C) for the past 72 hours (at time of judging eligibility)<br>● Primary focus is either line-related (central or peripheral intravenous cannula) or skin and soft tissue, AND source control achieved (for 'line-related' this means line removed; for 'skin and soft tissue' this means site Principal Investigator (PI) considers source control has been achieved and any abscess more than 2cm diameter has been drained)<br>● No evidence of metastatic foci (on clinical or radiological examination, but radiological imaging is not required to exclude metastatic foci if not clinically indicated)<br><br>When judging eligibility for early oral switch at platform **Day 14** (+/- 2 days):<br>● Clearance of SAB by platform Day 5: blood cultures negative for *S. aureus* on platform Day 5 (+/-1 day) AND no known subsequent

	positive blood cultures. If the most recent blood culture from Day 2-4 is negative for *S. aureus*, blood cultures do not need to be repeated on Day 5 to fulfil eligibility criteria (Day 5 blood cultures will be assumed to be negative in this situation)<br>● Afebrile (<37.8°C) for the past 72 hours (at time of judging eligibility)<br>● Site PI has determined that source control is adequate (further defined below)
Domain Specific Exclusions	Domain-specific exclusion criteria are listed in the following order: first as general exclusion criteria for *all* patients in the early oral switch domain (i.e. when assessing eligibility at either time point); followed by exclusion criteria for patients having eligibility for early oral switch judged specifically at *Day 7.*<br>When judging eligibility for early oral switch at platform **Day 7** (+/- 2 days) and **Day 14** (+/- 2 days), exclusion criteria are:<br>● Adherence to oral agents unlikely<br>● Unreliable gastrointestinal absorption (e.g. vomiting, diarrhoea, nil by mouth, anatomical reasons)<br>● There are no appropriate oral antibiotics due to contraindications, drug interactions, drug availability, or antibiotic resistance<br>● Ongoing IV therapy unsuitable (e.g. no intravenous access)<br>● Clinician deems not appropriate for early oral switch (provide reason)<br>● Patient no longer willing to participate in domain<br>● Clinical team deems that sufficient duration of antibiotic therapy has already been provided<br><br>When judging eligibility for early oral switch at platform **Day 7** (+/- 2 days), additional exclusion criteria are:<br>● Presence of prosthetic cardiac valve, pacemaker or other intracardiac implant<br>● Presence of intravascular clot (excluding superficial peripheral IV line-related thrombophlebitis), graft or other intravascular prosthetic material<br>● Intravascular/intracardiac infections (e.g. endocarditis, mycotic aneurysm)<br>● Presence of other intracardiac abnormalities felt to put patient at increased risk of endocarditis (e.g., bicuspid aortic valve)
Intervention-specific exclusions	Not applicable
Endpoints	Primary platform endpoint: All-cause mortality at 90 days from platform entry.<br>Secondary platform endpoints: refer to Core Protocol Section 6.8.

	Secondary domain-specific endpoints:<br>1. Number of days of IV antibiotic therapy in the total index hospitalisation (which includes OPAT), starting from platform entry, for those surviving until hospital discharge<br>2. Number of days alive and free of antibiotics by Day 42 from platform entry<br>a. For all antibiotics<br>b. For IV antibiotics<br>3. Peripherally inserted central catheter (PICC)/other central venous catheter complications requiring line removal, during the total index hospitalisation (which includes OPAT), starting from platform entry.<br>a. This outcome will be collected at total index hospital discharge and may include any of the following: catheter-related bloodstream infection; exit site infection; catheter-related superficial or deep venous thrombosis/thrombophlebitis, catheter blockage. It will NOT include rupture, leakage, displacement, or splitting unless this results in or occurs in addition to one of the above events.<br>4. Clinician-initiated change in treatment strategy from allocated EOS domain intervention (e.g. changed to IV antibiotics when allocated to oral antibiotics or vice versa) from reveal of EOS allocation until platform day 28 due to an adverse event deemed by the treating doctor/team to be of sufficient severity to change strategy<br>5. Clinician-initiated change in treatment strategy from EOS domain allocated intervention (e.g. changed to IV antibiotics when allocated to oral antibiotics or vice versa) from reveal of EOS allocation until platform day 28 due to presumed lack of efficacy of strategy according to the treating doctor/team
Decision criteria	The primary objective for this domain is to determine if early oral switch is **non-inferior** to no early oral switch in each silo. Non-inferiority is defined as an OR < 1.2 for the primary endpoint (where an OR > 1 indicates an increase in mortality for early oral switch treatment compared to no early oral switch). Within each cell, a stopping decision for non-inferiority will be recommended if, at a pre-specified interim analysis, the posterior probability of non-inferiority for the primary endpoint in that cell is greater than 99%. A stopping decision will be recommended for futility if, at a pre-specified interim analysis, the posterior probability of non-inferiority for the primary endpoint in that cell is less than 1%.<br>If, at any pre-specified interim analyses, the thresholds for the decision criteria are not met within a cell, then recruitment into the cell will continue.
Pre-specified secondary analyses	Pre-specified secondary analyses on the primary estimand will be performed by modifying the primary statistical model to account for the following covariates and their treatment interactions:<br>1. Time at which eligibility occurred from platform entry

	a. Participants eligible for early oral switch at Day 7<br>b. Participants not eligible for early oral switch at Day 7 and eligible at Day 14<br>2. Endocarditis<br>a. Left-sided endocarditis<br>b. Right- sided endocarditis<br>c. No endocarditis<br>3. Clinician-intended oral antibiotic regimen if participant eligible for EOS domain, identified prior to reveal of EOS domain allocation<br>a. β-lactams<br>b. Linezolid<br>c. Rifampicin (rifampin)<br>d. Quinolones<br>e. Trimethoprim-sulfamethoxazole<br>f. Clindamycin<br>g. Probenecid

TABLE OF CONTENTS

1. ABBREVIATIONS AND GLOSSARY ... 10

2. PROTOCOL APPENDIX STRUCTURE ... 12

3. EARLY ORAL SWITCH DOMAIN-SPECIFIC APPENDIX VERSION ... 13

3.1. Version history ... 13

4. EARLY ORAL SWITCH DOMAIN GOVERNANCE ... 13

4.1. Domain members ... 13

4.2. Contact Details ... 14

5. EARLY ORAL SWITCH DOMAIN-SPECIFIC WORKING GROUP AUTHORISATION ... 14

6. BACKGROUND AND RATIONALE ... 15

6.1. Domain definition ... 15

6.2. Domain-specific background ... 15

6.2.1. Early oral switch: background ... 15

6.2.2. Evidence for specific antibiotics that could be used for early oral switch. ... 17

6.2.3. Potential adverse effects of early oral switch ... 21

6.2.4. Need for a clinical trial of early oral switch versus continued IV treatment in SAB ... 23

7. DOMAIN OBJECTIVES ... 23

8. TRIAL DESIGN ... 24

8.1. Population ... 24

8.2. Eligibility criteria ... 24

8.2.1. Domain inclusion criteria ... 24

8.2.2. Domain exclusion criteria ... 25

8.2.3. Intervention exclusion criteria ... 26

8.3. Interventions ... 26

8.3.1. Early oral switch Interventions ... 26

8.3.2. Timing of initiation of Early Oral Switch domain ... 36

8.3.3. Duration of administration of Early Oral Switch domain ... 36

8.4. Concomitant care ... 37

8.5. Endpoints ... 37
8.5.1. Primary endpoint ... 37
8.5.2. Secondary endpoints ... 37
9. TRIAL CONDUCT ... 38
9.1. Domain-specific data collection ... 38
9.1.1. Microbiology ... 38
9.1.2. Clinical data and sample collection ... 39
9.1.3. Domain-specific study timeline ... 40
9.1.4. Domain-specific study visit day details ... 40
9.2. Criteria for discontinuation ... 42
9.2.1. Blinding ... 42
9.2.2. Unblinding ... 42
10. STATISTICAL CONSIDERATIONS ... 43
10.1. Estimands, endpoints, and intercurrent events ... 43
10.1.1. Primary estimand ... 43
10.1.2. Secondary estimands ... 43
10.2. Statistical modelling ... 46
10.2.1. Primary model ... 46
10.2.2. Secondary models ... 46
10.3. Decision criteria ... 46
10.4. Randomisation ... 47
10.5. Interactions with other domains ... 47
10.6. Pre-specified secondary analyses ... 47
10.7. Principal stratum policy ... 49
10.7.1. If allocated to early oral switch ... 49
10.7.2. If allocated to continued IV treatment ... 49
11. ETHICAL CONSIDERATIONS ... 49

11.1. Data Safety and Monitoring Committee (DSMC) 49

11.2. Potential domain-specific adverse events 49

11.3. Domain-specific consent issues 50

12. GOVERNANCE ISSUES 50

12.1. Funding of domain 50

12.2. Funding of domain interventions and outcome measures 51

12.3. Domain-specific declarations of interest 51

13. REFERENCES 52

TABLE OF TABLES

Table 1. Antibiotic options for early oral switch in SAB – dosing, administration, pharmacological properties 28

Table 2. Hierarchy of recommended oral antibiotics for early oral switch by silo (i.e. susceptibility of S. aureus). Site PIs and treating clinicians are encouraged, but not mandated, to select the highest antibiotic on this list which is appropriate for a given patient. 35

Table 3. Domain-specific schedule of visits and follow-up. 40

1. ABBREVIATIONS AND GLOSSARY

AGEP	Acute Generalised Exanthematous Pustulosis
AIN	Acute Interstitial Nephritis
CI	Confidence Interval
COPAT	Complex Outpatient Antibiotic Therapy
CrCl	Creatinine Clearance
CRRT	Continuous Renal Replacement Therapy
DILI	Drug-Induced Liver Injury
PD	Peritoneal Dialysis
DRESS	Drug rash with eosinophilia and systemic symptoms
DSA	Domain-Specific Appendix
DSWG	Domain-Specific Working Group
DSMC	Data Safety and Monitoring Committee
ECOFF	Epidemiological cut-off values
EOS	Early Oral Switch
fT>MIC	Free drug concentration above the MIC of the organism
GTSC	Global Trial Steering Committee
HD	Haemodialysis
HR	Hazards Ratio
IE	Infective Endocarditis
IV	Intravenous
MIC	Minimum Inhibitory Concentration
MIC90	MIC required to inhibit the growth of 90% of organisms
MRSA	Methicillin-resistant *Staphylococcus aureus*
MSSA	Methicillin-susceptible *Staphylococcus aureus*
OPAT	Outpatient Parenteral Antibiotic Therapy
OR	Odds Ratio
PI	Principal Investigator
PICC	Peripherally Inserted Central Catheter
PK/PD	Pharmacokinetic/pharmacodynamic
PSSA	Penicillin-susceptible *Staphylococcus aureus*

PWID	People Who Inject Drugs
RCT	Randomised Controlled Trial
RSA	Region-Specific Appendix
SAB	*Staphylococcus aureus* bacteraemia
SAR	Serious Adverse Reactions
SJS	Stevens Johnson Syndrome
SNAP	*Staphylococcus aureus* Network Adaptive Platform trial
TEN	Toxic Epidermal Necrolysis
TMP-SMX	Trimethoprim-sulfamethoxazole

2. PROTOCOL APPENDIX STRUCTURE

The structure of this protocol is different to that used for conventional trials because this trial is highly adaptive and the description of these adaptations is better understood and specified using a 'modular' protocol design. While all adaptations are pre-specified, the structure of the protocol is designed to allow the trial to evolve over time, for example by the introduction of new domains or interventions or both, and commencement of the trial in new geographical regions.

The protocol has multiple modules, in brief, comprising a Core Protocol (overview and design features of the study), a Statistical Analysis Appendix (details of the current statistical analysis plan and models, including simulations to support trial design), multiple Domain-Specific Appendices (DSA) (detailing all interventions currently being studied in each domain), and multiple Region-Specific Appendices (RSA) (detailing regional management and governance).

The Core Protocol contains all information that is generic to the trial, irrespective of the regional location in which the trial is conducted and the domains or interventions that are being tested. The Core Protocol may be amended but it is anticipated that such amendments will be infrequent.

The Core Protocol does not contain information about the intervention(s) within each domain, because one of the trial adaptations is that domains and interventions will change over time. Information about interventions, within each domain, is covered in a DSA. These Appendices are anticipated to change over time, with removal and addition of options within an existing domain, at one level, and removal and addition of entire domains, at another level. Each modification to a DSA will be subject to a separate ethics application for approval.

The Core Protocol does not contain detailed information about the statistical analysis or simulations, because the analysis model will change over time in accordance with the domain and intervention trial adaptations but this information is contained in the Statistical Analysis Appendix. These Appendices are anticipated to change over time, as trial adaptations occur. Each modification will be subject to approval from the Global Trial Steering Committee (GTSC) in conjunction with advice from the Statistical Subcommittee and the Data and Safety Monitoring Committee (DSMC).

The Core Protocol also does not contain information that is specific to a particular region in which the trial is conducted, as the locations that participate in the trial are also anticipated to increase over time. Information that is specific to each region that conducts the trial is contained within a RSA. This includes information related to local management, governance, and ethical and regulatory aspects. It

is planned that, within each region, only that region's RSA, and any subsequent modifications, will be submitted for ethical review in that region.

The current version of the Core Protocol, DSAs, RSAs, and the Statistical Analysis Appendix is listed in the Protocol Summary and on the study website (https://www.snaptrial.com.au/).

3. EARLY ORAL SWITCH DOMAIN-SPECIFIC APPENDIX VERSION

The version of the Early Oral Switch Domain-Specific Appendix is in this document's header and on the cover page.

3.1. Version history

Version 1: Approved by the Early Oral Switch Domain-Specific Working Group (DSWG) on the 29th March 2021.

4. EARLY ORAL SWITCH DOMAIN GOVERNANCE

4.1. Domain members

Chairs: Genevieve Walls
Anna Goodman

Members: Steven Tong
Joshua Davis
Archana Sud
Stephen Guy
Todd Lee
Matthew Cheng
Shirin Kalimuddin
Max Bloomfield
Susan Morpeth
Dafna Yahav
Amy Legg
Michael Marks
Anita Campbell

4.2. Contact Details

Chairs:

Dr Genevieve Walls
Dept Medicine/Infectious Diseases, Middlemore Hospital
Private Bag 93311, Otahuhu, Auckland 1640, New Zealand
Phone +64 21 770029
Email Genevieve.Walls@middlemore.co.nz

Dr Anna Goodman
MRC Clinical Trials Unit at University College London
London, WC1V 6LJ, UK
Phone +44 7548153014
Email anna.goodman@ucl.ac.uk

Project Manager:

Jocelyn Mora SNAP Clinical Trial Manager
The Peter Doherty Institute for Infection and Immunity
792 Elizabeth St Melbourne VIC AUSTRALIA
+61 (0) 38344 2554
jocelyn.mora@unimelb.edu.au

5. EARLY ORAL SWITCH DOMAIN-SPECIFIC WORKING GROUP AUTHORISATION

The Early Oral Switch Domain-Specific Working Group (DSWG) have read the appendix and authorise it as the official Early Oral Switch Domain-Specific Appendix for the SNAP trial. Signed on behalf of the committee,

Chair 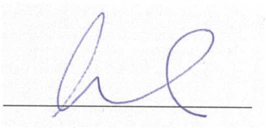 Date 29 March 2021

6. BACKGROUND AND RATIONALE

6.1. Domain definition

This is a domain within the SNAP trial to test the effectiveness of a strategy of early switch to oral antibiotics ('early oral switch') compared to a strategy of continued IV antibiotic treatment in patients with *Staphylococcus aureus* bacteraemia requiring admission to hospital.

6.2. Domain-specific background

6.2.1. Early oral switch: background

The recommended treatment for *Staphylococcus aureus* bacteraemia (SAB) is two to six weeks of intravenous (IV) antibiotics. The data supporting this recommendation are generally of low quality, predominantly observational and expert opinion. Prolonged IV antibiotic treatment has several disadvantages – adverse drug effects, venous catheter-associated complications, high cost, high healthcare resource use, and inconvenience to the patient. For these reasons there is interest in whether SAB treatment courses can be safely completed with oral antibiotics after a period of IV treatment ('early oral switch'). The pharmacokinetic/pharmacodynamic (PK/PD) properties of many antibiotics allow drug concentrations adequate for the treatment of *S. aureus* to be achieved by the oral route in appropriately selected patients. There are also a growing number of clinical studies adding to the evidence that early oral switch in the treatment of SAB is safe and effective.

It is generally accepted that IV administration is the best way to achieve instant therapeutic concentrations of an antibiotic in a sick patient, as factors such as gastrointestinal dysfunction and impaired level of consciousness may make the administration and absorption of oral antibiotics unreliable in this situation. Once the patient has stabilised and the source of infection is addressed, however, oral antibiotics may be adequate to continue treatment.

The tradition of prolonged IV treatment for SAB and other serious infections arose in the 1950s with the development of methicillin (which required IV administration due to its acid instability and insolubility) as the best treatment for hospital-based epidemics of penicillinase-producing staphylococcal infection. Many of the broad-spectrum antibiotics subsequently developed were not acid stable or had unacceptably low oral bioavailability, perpetuating an 'all-IV, all-the-time' culture for treatment of all but minor infections (1).

SAB has a propensity for metastatic spread and occult infection (2-5), particularly infective

endocarditis (IE), hence the recommendation for prolonged IV treatment of all SAB manifestations. These recommendations have evolved mostly based on expert opinion, observational studies (6), and because there is a lack of high-quality evidence for the superiority or equivalence of shorter course treatment (7). Recommendations for the duration of SAB treatment depend on whether the SAB is uncomplicated or complicated. In general SAB is considered uncomplicated if there is quick clinical resolution, rapid clearance of bacteraemia, and no evidence of metastatic sites of infection, endocarditis, or prosthetic material that could potentially be infected (8). These patients traditionally receive a minimum of two weeks of IV therapy (8, 9). Patients who do not meet the above criteria have complicated SAB and generally receive 4-6 weeks (or more) of IV therapy (8, 10, 11). Prolonged IV treatment may be given in hospital or in the outpatient setting via a long-term venous catheter (e.g., through an Outpatient Parenteral Antibiotic Therapy [OPAT] program).

There is growing interest in early oral switch for SAB treatment (12-24). The timing of early oral switch varies depending on the condition being treated, but in most studies is after a median of 5 (15) to 17 (12) days of IV antibiotic. In SNAP, we will randomise eligible patients to early oral switch at Day 7 (+/- 2 days) or Day 14 (+/- 2 days).

Prolonged IV therapy has several disadvantages when compared with oral treatment. In some studies, IV antibiotics have a higher rate of adverse drug events (25, 26). Venous catheter-associated complications also occur in up to 20% of patients discharged with a long-term venous catheter (27-29). Oral antibiotic treatment is cheaper than IV (30). Patients treated with prolonged IV antibiotics tend to stay in hospital longer, even if they are eventually discharged to an OPAT program (12, 31), with further associated risk, cost and inconvenience. The secondary costs (financial, social and emotional) of prolonged IV antibiotic treatment should also be considered – for example, the impact on ability to work.

Many antibiotics have excellent oral absorption, meaning that oral administration achieves plasma and tissue concentrations comparable to IV administration. Other antibiotics with lower oral absorption may achieve acceptable concentrations for SAB treatment (after source control and a period of IV treatment) with optimisation of PK/PD characteristics e.g. through optimised dosing. Examples of antibiotics with high oral bioavailability include linezolid (around 100% bioavailability), ciprofloxacin (65-85%), levofloxacin (99%), clindamycin (60-90%), moxifloxacin (90%), rifampicin (>90%) and trimethoprim-sulfamethoxazole (70-90%) (1). These agents have good penetration into most tissues and body fluids. Linezolid achieves concentrations in bone that exceed the MIC90 for many susceptible organisms and therapeutic concentrations in drainage fluid surrounding an

operative site are maintained for >16 hours (32). Linezolid and clindamycin have traditionally been seen as ‘bacteriostatic’ rather than ‘bactericidal’ drugs, limiting the appetite for their use in IE treatment. However, there is little evidence to support the concept that bacteriostatic drugs have poorer clinical effectiveness than bactericidal drugs (33, 34).

Clinical evidence for the safety and effectiveness of early oral switch in SAB treatment is also accruing. Recently there have been several reviews/commentaries and major studies published on early oral switch in uncomplicated SAB (7, 13, 35), IE treatment (not exclusively *S. aureus* IE) (12, 36) and osteoarticular infection (not exclusively *S. aureus* disease) (37). This evidence is summarised below.

There are also several ongoing trials of early oral switch in SAB treatment (SABATO; SAB7; RODEO-1) (22-24).

6.2.2. Evidence for specific antibiotics that could be used for early oral switch.

Dagher et al. (13) have recently published a comprehensive review of the evidence for oral antibiotics in uncomplicated SAB (some studies reviewed also include patients with complicated SAB), summarising published studies grouped by oral antibiotic studied. The existing evidence is strongest for linezolid, followed by fluoroquinolones in combination with another agent. The summary below is taken in part from this article.

6.2.2.1. Linezolid

There is a significant body of literature supporting the use of linezolid for early oral switch in the treatment of uncomplicated methicillin-susceptible (MSSA) and methicillin-resistant (MRSA) SAB. IV or oral linezolid appears to be at least as effective as standard therapy (13). Evidence includes two compassionate use studies of linezolid in *S. aureus* infection (including 71 cases of SAB), showing effectiveness of 63-86% (38, 39); an early pooled analysis of five randomised controlled trials (RCTs) of linezolid compared with vancomycin for *S. aureus* infection, which included 144 patients with SAB and showed an equivalent percentage of patients achieving clinical cure (40); and another RCT and two cohort studies with 402 SAB patients between them comparing linezolid IV-to-oral switch with standard care, all showing equivalent proportions of patients cured (21, 41-43). In some situations, linezolid may appear superior to standard therapy, depending on the ‘standard therapy’ it is compared with (e.g. teicoplanin) (44). In most studies reviewed, a significant proportion of study participants switched to oral linezolid after a period of IV treatment (38-41). One study specifically examined early oral switch: in a propensity score matched cohort, 45 patients who switched to oral linezolid after 3-9 days of IV linezolid were compared with 90 patients who continued IV treatment (21). No significant

differences in cure (however defined) between IV, oral or IV-to-oral linezolid and standard treatment were noted in any of these studies. Only two of the above studies focused specifically on SAB (21, 43), however, and none were powered to conclude non-inferiority of oral linezolid to standard treatment for SAB.

6.2.2.2. *Fluoroquinolones and fluoroquinolones plus rifampicin*

S. aureus (and particularly MRSA) can exhibit high rates of resistance to fluoroquinolones (especially ciprofloxacin) or can rapidly develop *de novo* resistance. Until recently the FDA had recommended against the use of any fluoroquinolones alone for MRSA infections.

A large retrospective study of MSSA bacteraemia treatment using single antibiotics in propensity-matched cohorts found no difference in 30-day mortality between those who received levofloxacin or moxifloxacin alone compared with those who received IV nafcillin or cefazolin (HR 1.33, 95% CI 0.30-5.96)(45).

Most evidence is for a fluoroquinolone-rifampicin combination treatment strategy. A 1996 study by Heldman et al. showed that oral ciprofloxacin plus rifampicin for treatment of right-sided *S. aureus* IE in people who inject drugs (PWID) achieved similar microbiological and clinical cure rates compared with patients treated with standard therapy (IV oxacillin or vancomycin plus gentamicin) (46). Schrenzel et al. found that oral fleroxacin with rifampicin was equivalent to IV flucloxacillin or vancomycin for the treatment of SAB and deep-seated *S. aureus* infections, although there were very few patients with MRSA infection in this study (47). The RODEO-1 trial will evaluate oral levofloxacin and rifampicin after 10 days of IV therapy in the treatment of left-sided *S. aureus* IE; results are awaited (24).

6.2.2.3. *Trimethoprim-sulfamethoxazole (TMP-SMX)*

The quality of evidence for and against the use of TMP-SMX in treating SAB is poor. At least two studies have shown that TMP-SMX (either IV or oral) failed to meet non-inferiority criteria or performed worse than IV vancomycin in the treatment of invasive *S. aureus* infections in patients with both MRSA and MSSA bacteraemia (48, 49). However, both studies were investigating the use of TMP-SMX as initial treatment, rather than in the context of early oral switch, and there were only small numbers of patients with SAB. Patients included in the study by Paul et al. were not necessarily clinically stable and were not required to have negative blood cultures before commencing TMP-SMX. All participants studied by Markowitz et al. were PWID, and severity criteria were not well defined. Markowitz et al.

did not find a difference in outcomes for MRSA treatment with either vancomycin or TMP-SMX, leading the authors to suggest that TMP-SMX might be acceptable for treatment of MRSA infections.

Other studies of TMP-SMX versus vancomycin have demonstrated no difference in SAB relapse or 30-day mortality, but these studies too have had significant limitations. Goldberg et al. (50) described a small retrospective cohort study of TMP-SMX versus vancomycin for MRSA bacteraemia, and Tissot-Dupont et al. (18) describe populations of patients with *S. aureus* IE at their institution in the years before and after instituting an oral IE treatment regimen (TMP-SMX and clindamycin); however, only 40% of the oral group had their per-protocol drugs from the start of treatment and 19% interrupted the protocol treatment. The oral group also appeared to have less severe disease.

While not specifically evaluating TMP-SMX or individual antibiotics, the following studies included a specified number of patients who received TMP-SMX as treatment for SAB. Jorgensen et al. report a retrospective cohort study of 70 patients with MRSA bacteraemia discharged on oral antibiotics to complete treatment in lieu of a traditional OPAT program. There were no differences in 90-day clinical failure between patients completing treatment with IV antibiotics or oral antibiotics; 24 patients (34%) received TMP-SMX as oral step-down (20). Perez-Rodriguez et al. report a retrospective observational study evaluating 90-day recurrence in 201 patients with complicated and uncomplicated SAB (excluding IE), 125 (62%) of whom were treated with early oral switch after 8-17 days of IV antibiotic. No difference in the primary outcome was reported between the early oral switch group and the continued IV group. Oral TMP-SMX was used in 66% of patients (14).

TMP-SMX with rifampicin may be a promising combination. Harbarth et al. (51) found no significant difference in clinical cure rate between patients with MRSA infection at various sites treated with linezolid and those treated with IV-to-oral TMP-SMX with rifampicin. However, there were only 9 patients with MRSA bacteraemia in each group.

6.2.2.4. Beta-lactams

Traditionally there has been even less confidence with using oral beta-lactams for SAB treatment, but pharmacological and clinical evidence for their safety is also accruing. Beta-lactam antibiotics display time-dependent antimicrobial activity (expressed as the percentage of the dosing interval where the free drug concentration is above the minimum inhibitory concentration [MIC] of the organism [%fT>MIC]). Validated pharmacodynamic targets for early oral switch in uncomplicated SAB have not been established, but animal models suggest these may be as low as 24% fT>MIC (52-54). For successful treatment of complicated SAB a higher fT>MIC of >50% has been proposed, although

evidence for a PK/PD target in this setting is lacking (52, 54). In the case of flucloxacillin, fT>MIC of 24% may be achieved with an oral dose of 1g three to four times a day (55). Using probenecid in combination with a beta lactam allows fT>MIC of >50% to be achieved without resorting to impractically high or frequent oral beta-lactam dosing – for example, flucloxacillin 1g four times a day with probenecid 500mg four times a day - which is the same exposure found in some studies for patients taking flucloxacillin 2g IV every six hours (52). Other oral beta-lactams actually have good oral absorption. For example, the bioavailability of amoxicillin and cefalexin is 74-92% and 90% respectively (1). Beta-lactams also display a post-antibiotic effect for *S. aureus* that may add to their clinical efficacy.

There is relatively little published clinical evidence for the use of beta-lactams for early oral switch in SAB. Bupha-Intr et al. (15) describe early oral switch in 84 patients with uncomplicated SAB, predominantly MSSA bacteraemia. Most patients (86%) received an oral beta-lactam (mostly flucloxacillin) after a median of five days of IV treatment. There was one relapse in 90 days and no deaths, suggesting that oral beta-lactams (including flucloxacillin, with its comparatively lower oral bioavailability of around 50%) are safe and effective in this context. Thwaites et al. (56) report data from prospectively enrolled patients in the UK, Vietnam and Nepal with MSSA and MRSA bacteraemia. In the UK, 49% of patients switched to exclusive oral antibiotics at some point during treatment and 25% received oral antibiotics for more than half of the treatment course. The specific oral antibiotics used are not reported except where the authors comment on 14 patients (13 from the UK) who received no IV treatment at all; 11 of these patients received oral flucloxacillin and all survived to discharge. The POET study (Partial Oral Versus Intravenous Antibiotic Treatment of Endocarditis) randomised 400 stable patients with left sided IE to prolonged IV therapy or early oral switch (12). Early oral switch after a median of 17 days of IV treatment was non-inferior to prolonged IV treatment in terms of the primary outcome (a composite outcome of all-cause mortality, unplanned cardiac surgery, embolic events and relapse of blood cultures). POET included 87 patients with methicillin-susceptible *S. aureus* IE, 47 (54%) of whom were switched to oral antibiotics: 35 were treated with a beta-lactam (in combination with another agent) – most commonly dicloxacillin or amoxicillin. There was no difference in the primary outcome in the group of patients with *S. aureus* IE, whether treated with IV or oral antibiotics, although the study was under-powered to draw conclusions about early oral switch in this group. There were no patients with MRSA IE.

6.2.2.5. *Other studies*

Spellberg et al. (36) have recently reviewed the evidence for oral antibiotic treatment in IE (not limited to *S. aureus* IE, however) and concluded that the data support further investigation into early oral switch in IE treatment. As well as IE studies already mentioned, Mzabi et al. (57) describe a large retrospective cohort of 426 cases of definite and probable IE, including 81 cases of *S. aureus* IE (14 MRSA). Around 50% of patients in the overall cohort were switched to oral antibiotics when stable with no increase in mortality or relapse, although the oral group was less likely to have *S. aureus* IE. In the *S. aureus* IE group, 28 (35%) patients (two with MRSA IE) were switched to oral antibiotics. These patients had a median of 28 days (range 1-56 days) IV antibiotics before switching to oral treatment, which is significantly longer than we are proposing for SNAP. A variety of oral antibiotic regimens were used for treatment of staphylococcal IE (including coagulase negative staphylococcal IE) including, most commonly, the combinations clindamycin-rifampicin/fluoroquinolone (15 patients); fluoroquinolone-rifampicin (13 patients); and amoxicillin-rifampicin/fluroquinolone/clindamycin (9 patients).

Jorgensen et al. (20), mentioned above, suggested that early oral switch was not unsafe for stable MRSA bacteraemia patients who would otherwise be discharged on OPAT. As well as TMP-SMX, linezolid was used in 35/70 patients (50%) and clindamycin in 11/70 (16%) patients.

The OVIVA study (37) of 1054 participants with osteoarticular infection included 378 patients with microbiologically confirmed *S. aureus* disease, although those with SAB or IE were specifically excluded. Oral therapy was non-inferior to IV in this group, but these results cannot necessarily be extrapolated to patients with SAB and osteoarticular infection.

6.2.3. Potential adverse effects of early oral switch

6.2.3.1. Pharmacological factors

All recommended oral agents in SNAP have good to excellent bioavailability and tissue penetration. In many cases concentrations achieved are comparable with IV treatment (linezolid, fluoroquinolones, trimethoprim-sulfamethoxazole, rifampicin); where this is not the case, concentrations achieved should still be adequate to treat SAB, particularly where the patient is no longer bacteraemic, is stable, and has had good source control. For each of the recommended antibiotics in the SNAP early oral switch domain, pharmacokinetic factors (protein binding, half-life, absorption, penetration to likely site of infection) were considered relative to the ECOFF or MIC breakpoint for *S. aureus* to optimise dosing and maximise the chance of therapeutic success. The treating clinical team’s choice of oral antibiotic for step-down therapy will be influenced by the clinical scenario and specific properties of

the various oral antibiotics. The protocol will not mandate oral antibiotic choice for the early oral switch domain, but recommendations will be provided to aid the decision-making of the treating team.

Most IV treatment of SAB is with beta-lactams or glycopeptides. Although several oral antibiotics recommended for SNAP are in different antibiotic classes, they are all broadly used agents with known adverse effects. There is the risk of drug-drug interactions with some oral antibiotics. For example, rifampicin is a potent enzyme inducer and is notorious for interfering with the metabolism of other medications, sometimes with significant clinical effect e.g. rifampicin reduces the efficacy of warfarin. Most SNAP study participants will be reviewed by Infectious Diseases physicians or clinical microbiologists who are experienced in managing complex drug interactions. Serious adverse reactions (SARs), including those resulting from drug-drug interactions, will be collected as part of the SNAP core protocol.

6.2.3.2. Microbiological factors

A high *S. aureus* inoculum combined with potentially lower plasma concentrations achieved with some oral antibiotics may increase the risk of treatment failure. However, the protocol is designed to exclude unstable patients without adequate source control from the early oral switch domain.

6.2.3.3. Clinical factors

It may be difficult to differentiate uncomplicated from complicated SAB. A recent randomised trial of patients with 'uncomplicated' SAB showed that one-third were eventually found to have complicated SAB (4). The early oral switch protocol requires differentiation of uncomplicated from complicated SAB; participants with uncomplicated SAB will be switched to oral antibiotics earlier. Later recognition of a metastatic focus of infection may result in under-treatment. However, in this situation IV antibiotics are not likely to be effective either, good source control being a fundamental tenet of all infection treatment. In addition to source control, a key determinant of successful treatment is likely to be the duration of therapy, and IV and oral antibiotics are expected to be equally effective if given for the same duration.

6.2.3.4. Clinical follow-up and monitoring

Patients discharged from hospital on IV antibiotics are usually closely monitored by an OPAT team. One can be reasonably certain that patients on IV antibiotics under OPAT are adherent to treatment, or, if they are not, that this will quickly be brought to the treating clinician's attention. It is harder to

monitor adherence in patients on oral antibiotics. The frequent monitoring performed by a traditional OPAT program also allows clinical failure and treatment-related adverse effects to be identified quickly, which may not be the case for patients self-administering oral antibiotics in the community. However, increasing numbers of clinicians are practising early oral switch (e.g. for treatment of osteoarticular infection) and many sites now use their OPAT services to monitor patients on long-term oral antibiotic therapy in the same way that they monitor patients on IV therapy (sometimes referred to as 'COPAT' or Complex Outpatient Antibiotic Therapy). The early oral switch protocol recommends that SNAP participants receive the same monitoring and follow-up regardless of whether they are randomised to continued IV treatment or early oral switch.

6.2.4. Need for a clinical trial of early oral switch versus continued IV treatment in SAB

The reduction of unnecessary IV antibiotic use is a key feature of antimicrobial stewardship programs worldwide. There is emerging evidence for the efficacy of early oral switch in uncomplicated SAB and certain complicated SAB scenarios (e.g. IE). To date, the quality of this evidence has been too low (with a few exceptions) to effect a widespread change in treatment recommendations. We believe that the accumulation of evidence is sufficient for there to be clinical equipoise for randomising patients to early oral switch versus continued IV treatment within a large, well-designed, prospective, randomised, real-world trial such as SNAP. The traditional treatment paradigm – prolonged IV antibiotics often for six weeks or more – is associated with increased risk of adverse effects, expense, and use of increasingly stretched healthcare resources compared with oral antibiotic treatment. Demonstration of non-inferiority of early oral switch in SAB would change practice. Failure to demonstrate non-inferiority would also answer a very important clinical question.

7. DOMAIN OBJECTIVES

The objective of this domain is to determine the effectiveness of early switch to oral antibiotics ('early oral switch') compared with continued IV antibiotic treatment for patients with *Staphylococcus aureus* bacteraemia (SAB) requiring admission to hospital.

We hypothesise that the probability of all-cause mortality at 90 days after platform entry in the early oral switch group will be non-inferior to the group who continued with IV treatment, in each silo, for those participants eligible for an early oral switch. The following interventions will be available:

- Switch to oral antibiotics at platform **Day 7** (+/- 2 days) and platform **Day 14** (+/- 2 days) ('early oral switch'), where Day 1 is the calendar day of platform entry
- Continued intravenous antibiotic treatment ('continued IV treatment') – current standard of care

8. TRIAL DESIGN

This domain will be conducted as part of the SNAP trial (see Core Protocol Section 6). Only eligible patients will enter this domain. Treatment allocation will be at a fixed 1:1 ratio, as described in the Core Protocol Section 6.7.

8.1. Population

Patients with *S. aureus* bacteraemia admitted to participating hospitals.

8.2. Eligibility criteria

Patients are eligible for this domain if they meet all of the platform-level inclusion criteria and none of the platform-level exclusion criteria (see Core Protocol Section 6.5) AND all of the domain-level inclusion and none of the domain-level exclusion criteria. Patients eligible for SNAP may have conditions that exclude them from the early oral switch domain.

All platform-eligible participants will be randomised within the early oral switch domain at platform entry (to early oral switch or continued IV treatment) but the intervention allocation will remain hidden unless the participant meets the eligibility criteria for the early oral switch domain (see Sections 8.3.2 and 9.2.1).

Eligibility will be assessed at two time periods, with eligibility criteria that differ for each time period. The first assessment will be at platform **Day 7** (+/- 2 days) and the second at **Day 14** (+/- 2 days). Patients meeting eligibility and having their allocation revealed at Day 7 will not be assessed again at Day 14. Patients not meeting eligibility at platform Day 7 will be assessed again at Day 14. Patients not meeting eligibility at platform Day 14 will not be assessed again and should continue with standard of care.

8.2.1. Domain inclusion criteria

At platform **Day 7** (+/- 2 days):

- Clearance of SAB by platform Day 2: blood cultures negative for *S. aureus* from platform Day 2 onwards AND no known subsequent positive blood cultures
- Afebrile (<37.8°C) for the past 72 hours (at time of judging eligibility)
- Primary focus is either line related (either central or peripheral IV cannula) or skin and soft tissue, AND source control achieved (for 'line-related' this means line removed; for 'skin and soft tissue' means site PI considers source control to have been achieved and any abscess more than 2cm diameter has been drained)
- No evidence of metastatic foci (on clinical or radiological examination, but radiological imaging is not required to exclude metastatic foci if not clinically indicated)

At platform **Day 14** (+/- 2 days):

- Clearance of SAB by platform Day 5: blood cultures negative for *S. aureus* from platform Day 5 (+/-1 day) AND no known subsequent positive blood cultures. If the most recent blood culture from Day 2-4 is negative for *S. aureus*, blood cultures do not need to be repeated on Day 5 to fulfil eligibility criteria (Day 5 blood cultures will be assumed to be negative in this situation)
- Afebrile (<37.8°C) for the past 72 hours (at time of judging eligibility)
- Site PI has determined that source control is adequate*

*This could include patients for whom the aim of treatment is long-term suppression rather than cure, for example, infected pacemaker wire or prosthetic joint where removal of wire or prosthesis is not possible (i.e. source control is appropriate for the treatment aim). There may also be situations where surgical source control has been appropriately decided against (e.g. because it is judged to be unacceptably high-risk, or because an abscess is not amenable to drainage) and medical treatment with antibiotics and without surgery is deemed to be the appropriate treatment. Pulmonary abscess would be an example of an abscess not amenable to drainage.

8.2.2. Domain exclusion criteria

Domain-specific exclusion criteria are listed in the following order: first as general exclusion criteria for *all* patients in the early oral switch domain (i.e. when assessing eligibility at either time point); followed by exclusion criteria for patients having eligibility for early oral switch judged specifically at *Day 7.*

When judging eligibility at platform **Day 7** (+/- 2 days) and at **Day 14** (+/- 2 days), exclusion criteria are:

- Adherence to oral agents unlikely (as judged by site PI in consultation with the treating team)
- Unreliable gastrointestinal absorption (e.g. vomiting, diarrhoea, nil by mouth, anatomical reasons)
- There are no appropriate oral antibiotics due to contraindications, drug interactions, drug availability, or antibiotic resistance
- Ongoing IV therapy unsuitable e.g. no IV access
- Clinician deems not appropriate for early oral switch (provide reason)
- Patient no longer willing to participate in domain
    - In the days leading up to judging eligibility, it may be helpful to discuss with the patient the potential for continued IV treatment versus oral switch, to allow hospital discharge planning
- Clinical team deems that sufficient duration of antibiotic therapy has already been provided

When judging eligibility for early oral switch at platform **Day 7** (+/- 2 days), exclusion criteria are:

- Presence of prosthetic cardiac valve, pacemaker or other intracardiac implant
- Known presence of intravascular clot (excluding superficial peripheral IV line-related thrombophlebitis), graft or other intravascular prosthetic material
- Intravascular/intracardiac infections (e.g., endocarditis, mycotic aneurysm)
- Presence of other intracardiac abnormalities felt to put patient at increased risk of endocarditis (e.g., bicuspid aortic valve)

8.2.3. Intervention exclusion criteria

As this domain involves testing a strategy rather than individual antibiotic agents, there are no antibiotic agent specific exclusions.

8.3. Interventions

8.3.1. Early oral switch Interventions

- **Early oral switch**: switch to oral antibiotic at platform **Day 7** (+/- 2 days) or **Day 14** (+/- 2 days):
    - **Choice of antibiotic(s) used for early oral switch is at the discretion of the treating clinician. Table 1 and 2, below, give suggestions and recommendations for antibiotic choices but are not mandated for SNAP.**
    - **Table 1** shows antibiotics commonly used for *S. aureus* infection, which could be considered for early oral switch in SAB (this list is not exhaustive). Details of dosing

(including for renal impairment), bioavailability and other pharmacological properties, need for fasting, and safety in pregnancy are included.

- **Table 2** shows recommended oral antibiotics for each silo (susceptibility of *S. aureus*) i.e. PSSA, MSSA or MRSA
  - Choice of oral antibiotic is at the discretion of the treating clinician; however, we encourage investigators to prescribe oral antibiotics based on recommendations from **Table 2** (here the oral antibiotics are listed in the order of recommendation)
  - For PSSA and MSSA, investigators are encouraged to continue the antibiotic the patient was allocated in the backbone domain. For example, if allocated to penicillin for PSSA in the backbone domain, switch to oral amoxicillin. If allocated to (flu)cloxacillin in the backbone domain, switch to oral dicloxacillin / flucloxacillin.
- A single antibiotic is recommended in most situations. Situations where dual antibiotic treatment may be considered include:
  - If using fusidic acid + rifampicin or fluoroquinolone + rifampicin
  - Where a 'biofilm active' agent such as rifampicin may be desirable (e.g. prosthetic joint infection, prosthetic valve endocarditis). Rifampicin started for this indication before reveal of randomisation allocation may be continued regardless of allocation
  - The use of probenecid in combination with an oral beta-lactam
  - Either single or dual oral therapy is acceptable for endocarditis, at the discretion of the treating clinician

- **Continued IV antibiotic treatment**
  - If randomised to 'continued IV treatment', the choice of IV antibiotic used is at the discretion of the treating clinician. Where possible, patients should remain on the IV antibiotic allocated as part of the backbone antibiotic domain (e.g., penicillin, (flu)cloxacillin, or cefazolin) for the duration of treatment (with the exception of cefazolin in the combination treatment arm of the MRSA silo, which should be ceased on trial Day 7, as per the Backbone Domain: MRSA silo protocol)

Table 1. Antibiotic options for early oral switch in SAB – dosing, administration, pharmacological properties

Principles:

- For beta-lactams, maximum doses have been recommended to overcome theoretical issues with drug exposure (bioavailability). Lower doses in specific circumstances have been recommended in the footnotes.
- Dosing regimens to minimise patient inconvenience have been prioritised, as explained in footnotes.
- Doses are suggestions only and alternate doses used as standard local practice can be maintained.
- Contraindications, including significant drug interactions, are not listed and are the responsibility of the prescribing team to review and manage. Some considerations are provided to aid the choice of drug.
- We have not recommended dose changes for obesity or pregnancy in the setting of early oral switch (i.e. step-down therapy after a period of intravenous therapy/source control/clinical stability). Despite a potential effect of obesity and pregnancy on pharmacokinetics (increased volume of distribution), we will not proceed to dose adjustment for step-down therapy.
- With increased creatinine clearance in pregnancy, there is a theoretical concern that the concentration of the antibiotics may not be over the required MIC for a sufficient period of time. However, general practice in obstetric dosing of antibiotics is to dose at the highest end of the dosing range, as is currently planned in the SNAP study.

Drug	Standard Dose	Dose in renal impairment[1,2]	Bio-availability	Fasting	Protein binding	Pregnancy category[3]	Half life	Break point or ECOFF
Amoxicillin	1g 6-hourly [4,5]	CrCl 10 to 30mL/min and CRRT: 1g 8-hourly.<br>CrCl less than 10mL/min, HD and PD: 1g 12-hourly.	74-92%	No	17-20%	Safe to use all trimesters	1.2-1.5 hours	ND
Cefadroxil	1g 12-hourly	CrCl 10-50mL/min or CRRT: 1g stat then 500mg 12-hourly.<br>CrCl less than 10mL/min: 1g stat then 500mg 36-hourly.<br>HD: 1g stat and 1g post HD<br>PD: 500mg 24-hourly.	90%	No	20%	Safe to use all trimesters	1.5 hours	ND
Cefalexin	1g 6-hourly[6,7]	CrCl less than 10mL/min, HD or PD: 1g 12 hourly.<br>CRRT: standard dose.	90%	No	10-19%	Safe to use all trimesters	1 hour	8 (ECOFF)

[1] Dose derived from Australian Therapeutic Guidelines: Antibiotic v16, 2019, Sandford Guide and Licensed Product Information from FDA.
[2] HD: haemodialysis, PD: peritoneal dialysis.
[3] Please see Pregnancy appendix for further detail
[4] Dose derived from POET trial (Partial Oral versus Intravenous Antibiotic Treatment of Endocarditis) (12)
[5] Probenecid (dose: 500mg if CrCl 60 mL/min or more, 250mg if CrCl between 60 to 30 mL/min, do not use if CrCL less than 30 mL/min) may be co-administered with each dose of beta-lactam to improve drug exposure. Administer with amoxicillin 1g q6h or 1g q8h at the discretion of the treating clinician. We recommend giving probenecid with food to prevent nausea.
[6] Clinical efficacy in uncomplicated SAB has been demonstrated at a dose of 1g orally q8h (15)
[7] Probenecid (dose: 500mg if CrCl 60 mL/min or more, 250mg if CrCl between 60 to 30 mL/min, do not use if CrCL less than 30 mL/min) may be co-administered with each dose of beta-lactam to improve drug exposure. Administer with cefalexin 1g q6h or 1g q8h at the discretion of the treating clinician. We recommend giving probenecid with food to prevent nausea.

Ciprofloxacin PLUS rifampicin (use only in combination)	**Ciprofloxacin**750mg 12-hourly	CrCl less than 30mL/min, HD, PD: 750mg 24-hourly.<br>CRRT: 250 to 500mg 12-hourly.	70%	No[8]	20-40%	Avoid in pregnancy	4 hours	BP: 0.001<br>ECOFF: 1.0
	**Rifampicin:** Weight <60kg: 600 mg per day; weight >60kg: 900mg per day.[9,10,11]	No change to standard dose.	70-90%	Yes[12]	80%	Reasonable to use in trimester 1 and 2; monitoring required trimester 3 (liver function tests at baseline, Week 1, 2 and 4). May be associated with increased risk of haemorrhagic disorders in newborn.	3 hours	BP: 0.06<br>ECOFF: 0.016
Clindamycin	450mg 8-hourly[13]	No change to standard dose	55% or 90%	No	94%	Reasonable to use all trimesters	2.4 hours	0.25

[8] Ledergerber et al. Effect of standard breakfast on drug absorption and multiple-dose pharmacokinetics of ciprofloxacin (58)

[9] Doses above 600 mg per day should be divided into two doses.

[10] Use with caution in liver disease - can cause hepatotoxicity.

[11] Dose derived from the ARREST trial (59)

[12] Ideally, administer 30 minutes before or two hours after a meal.

[13] For oral administration 450mg is the maximum dose licensed by the TGA. Clindamycin dosed 8-hourly showed significantly longer bactericidal activity against *S. aureus* when compared to 12-hourly regimens, (87.5 to 100% versus 49.6 to 77.1%, P < 0.001) (60)

Cloxacillin	1g 6-hourly[14]	No change to standard dose.	32-50%	No[15]	94%	Reasonable to use all trimesters	0.5 hours	0.5
Dicloxacillin	1g 6-hourly[16,17]	CrCl less than 10mL/min, HD or PD: 1g q8h.<br>CRRT: standard dose.	35-76%	No[18]	95%	Reasonable to use all trimesters	0.7 hours	0.5
Doxycycline	100mg<br>12-hourly	No change to standard dose.	90%	No[19]	93%	Avoid in pregnancy	18 hours	ECOFF 0.5
Flucloxacillin	1g 6-hourly[20,21]	CrCl less than 10mL/min, HD or PD: 1g q8h.<br>CRRT: standard dose.	44-55%	No[22]	95%	Reasonable to use all trimesters	0.75 hours	0.5
Fusidic acid PLUS rifampicin	**Fusidic acid:** 500mg	No change to standard dose.	91%	No	95-99%	Data scarce in human pregnancy; avoid	8-10 hours	BP: 1

[14] Probenecid (dose: 500mg if CrCl 60 mL/min or more, 250mg if CrCl between 60 to 30 mL/min, do not use if CrCL less than 30 mL/min) may be co-administered with each dose of beta-lactam to improve drug exposure. Administer with cloxacillin 1g q6h at the discretion of the treating clinician. We recommend giving probenecid with food to prevent nausea.

[15] Product information advises administration in the fasting state to maximise bioavailability, but this may make adherence difficult. Data that show decreased clinical efficacy when administered with food are lacking. We have recommended a high dose to optimise drug concentrations if administration in the fasted state is not possible.

[16] Dose derived from POET trial (12)

[17] Probenecid (dose: 500mg if CrCl 60 mL/min or more, 250mg if CrCl between 60 to 30 mL/min, do not use if CrCL less than 30 mL/min) may be co-administered with each dose of beta-lactam to improve drug exposure. Administer with dicloxacillin 1g q6h or 1g q8h at the discretion of the treating clinician. We recommend giving probenecid with food to prevent nausea.

[18] Product information advises administration in the fasting state to maximise bioavailability, but this may make adherence difficult. Data that show decreased clinical efficacy when administered with food are lacking. We have recommended a high dose to optimise drug concentrations if administration in the fasted state is not possible.

[19] Taking doxycycline on an empty stomach can cause nausea.

[20] Clinical efficacy in uncomplicated SAB has been demonstrated at a dose of 1g orally q8h (15)

[21] Probenecid (dose: 500mg if CrCl 60 mL/min or more, 250mg if CrCl between 60 to 30 mL/min, do not use if CrCL less than 30 mL/min) may be co-administered with each dose of beta-lactam to improve drug exposure. Administer with flucloxacillin 1g q6h or 1g q8h at the discretion of the treating clinician (52)

[22] Although the product information recommends administration in the fasting state to maximise bioavailability, administration with food is unlikely to reduce efficacy in most situations (55). We have recommended a high dose of flucloxacillin to optimise drug concentrations.

(use in combination only)	8- to 12-hourly							ECOFF: 0.5
	**Rifampicin:** Weight <u><60kg: </u>600 mg per day; weight >60kg: 900mg per day.[23, 24]	No change to standard dose.	70-90%	Yes[25]	80%	Reasonable to use in trimesters 1 and 2; monitoring required in trimester 3 (liver function tests at baseline, Week 1, 2 and 4). May be associated with increased risk of haemorrhagic disorders in newborn).	3 hours	BP: 0.06<br>ECOFF: 0.016
Levofloxacin PLUS rifampicin (use in combination only)	**Levofloxacin:** 750mg daily	CrCl 20-49mL/min: 750mg q48h.<br>CrCl< 20mL/min,HD,PD: 750mg initial dose; then 500mg q48h<br>CRRT: 250mg 24-hourly[26]	99%	No	24-38	Avoid in pregnancy	7 hours	ECOFF 0.25
	**Rifampicin:** Weight <u><60kg: </u>600 mg per day; weight >60kg: 900mg per day.[27, 28]	No change to standard dose.	70-90%	Yes[29]	80%	Safe to use trimester 1 and 2; monitoring required trimester 3 (liver function tests at	3 hours	BP: 0.06<br>ECOFF: 0.016

[23] Doses above 600 mg per day should be divided into two doses.
[24] Use with caution in liver disease - can cause hepatotoxicity.
[25] Ideally, administer 30 minutes before or two hours after a meal.
[26] Malone RS et al. Pharmacokinetics of levofloxacin and ciprofloxacin during continuous renal replacement therapy in critically ill patients (61)
[27] Doses above 600 mg per day should be divided into two doses.
[28] Use with caution in liver disease - can cause hepatotoxicity.
[29] Ideally, administer 30 minutes before or two hours after a meal.

						baseline, Week 1, 2 and 4). May be associated with increased risk of haemorrhagic disorders in newborn).		
Linezolid	600mg 12-hourly[30, 31]	CrCl less than 10mL/min, HD or PD: 600mg 24-hourly[32].<br>CRRT: standard dose.	100%	No	30%	No data in human pregnancy; avoid.	5 hours	BP: 4<br>ECOFF: ND
Moxifloxacin PLUS rifampicin<br>(use in combination only)[33]	**Moxifloxacin:** 400mg daily	No change to standard dose.	89%	No	30-50	Avoid in pregnancy	10- 14 hours	ECOFF 0.5
	**Rifampicin:** Weight <u><60kg: </u>600 mg per day; weight >60kg: 900mg per day.[34, 35]	No change to standard dose.	70-90%	Yes[36]	80%	Safe to use trimester 1 and 2; monitoring required trimester 3 (liver function tests at baseline, Week 1, 2 and 4). May be associated with	3 hours	BP: 0.06<br>ECOFF: 0.016

30 Risk of haematological toxicity increases with use beyond 14 days (62)

31 Pyridoxine 50mg-100mg/day to prevent or delay anaemia can be considered if using linezolid for > 7 days; evidence for benefit conflicting (63)

32 The optimal dose of linezolid in renal impairment is unknown, alternative doses include 300 mg 12-hourly or 600 mg 12-hourly. Patients are at an increased risk of thrombocytopenia if continued on 600mg 12-hourly in the setting of renal impairment. Therapeutic drug monitoring aiming for a trough concentration between 2 and 7mg/L is recommended for patients on linezolid with renal impairment (64)

33 Rifampicin may reduce serum concentrations of moxifloxacin, though the clinical significance of this interaction remains uncertain. Consider using another quinolone in combination with rifampicin.

34 Doses above 600 mg per day should be divided into two doses.

35 Use with caution in liver disease - can cause hepatotoxicity.

36 Ideally, administer 30 minutes before or two hours after a meal.

						increased risk of haemorrhagic disorders in newborn).		
Tedizolid	200mg once daily	No change to standard dose.	91%	No	70-90%	Little data in pregnancy; should only be used if the benefit justifies the potential risk to the fetus.	12 hours	BP: $\leq$ 0.5
Trimethoprim plus sulfamethoxazole (TMP+SMX)	320/1600 mg 12-hourly<br>or<br>160/800 mg<br>8-hourly	CrCl 26-50mL/min: normal for 14 days, then 160/800mg 12-hourly.<br>CrCl 15 to 25mL/min: normal for 3 days, then 320/1600mg 24-hourly.<br>For CrCl less than 15mL/min: avoid use.[37]	70-90%	No	44/70%	Avoid in first and third trimesters	11 hours	2 (TMP)

[37] Sulfamethoxazole can cause pancreatic insulin release, resulting in clinically significant hypoglycaemia, particularly in patients with renal impairment, receiving high doses, or concomitantly taking a sulfonylurea (65)

Table 2. Hierarchy of recommended oral antibiotics for early oral switch by silo (i.e. susceptibility of S. aureus). Site PIs and treating clinicians are encouraged, but not mandated, to select the highest antibiotic on this list which is appropriate for a given patient.

Silo	Recommended oral antibiotic according to allocated backbone domain	
**PSSA**	**Benzylpenicillin**	**(Flu)cloxacillin**
	1. Amoxicillin<br>2. Flucloxacillin/dicloxacillin<br>3. Cefalexin/cefadroxil<br>4. Linezolid	1. Flucloxacillin/dicloxacillin<br>2. Amoxicillin<br>3. Cefalexin/cefadroxil<br>4. Linezolid
**MSSA**	**(Flu)cloxacillin**	**Cefazolin**
	1. Flucloxacillin/dicloxacillin<br>2. Cefalexin/cefadroxil<br>3. Linezolid	1. Cefalexin/cefadroxil<br>2. Flucloxacillin/dicloxacillin<br>3. Linezolid
**MRSA**	**Vancomycin/daptomycin**	**Vancomycin/daptomycin + cefazolin**
	1. Linezolid<br>2. Fluoroquinolone + rifampicin<br>3. TMP-SMX<br>4. Fusidic acid + rifampicin	1. Linezolid<br>2. Fluoroquinolone + rifampicin<br>3. TMP-SMX<br>4. Fusidic acid + rifampicin

8.3.2. Timing of initiation of Early Oral Switch domain

Participants enrolled in SNAP are randomised within the early oral switch domain at platform entry, but the reveal of allocation (early oral switch versus continued IV treatment) only occurs once eligibility for early oral switch is confirmed (see Section 9.3.1, Blinding).

Participants enrolled in SNAP will be assessed at platform Day 7 (+/-2 days) for eligibility for early oral switch. If the participant is eligible for early oral switch at Day 7, reveal of allocation will occur. The participant will switch to oral antibiotics or will continue IV antibiotics according to allocation.

For those not eligible for early oral switch at Day 7, eligibility will be assessed again at platform Day 14 (+/- 2 days). If the participant is eligible for early oral switch at Day 14, reveal of allocation will occur. The participant will switch to oral antibiotics or will continue with IV antibiotics according to allocation.

If participants are not eligible at Day 7 or at Day 14, then treatment is continued according to the treating clinician's discretion and there are no further assessments for eligibility. The allocation of domain intervention is never revealed for these participants.

8.3.3. Duration of administration of Early Oral Switch domain

Participants eligible for early oral switch at Day 7

Participants must have a minimum of a further five days of allocated study treatment strategy (whether IV or oral) within the early oral switch domain after eligibility is confirmed at platform Day 7. Participants will typically require a minimum of 14 days total antibiotic treatment from platform entry (Day 1).

Participants eligible for early oral switch at Day 14:

Participants must have a minimum of a further 12 days of allocated study treatment strategy (whether IV or oral) within the early oral switch domain after eligibility is confirmed at platform Day 14. Participants will typically require a minimum of four weeks total antibiotic treatment from platform entry. Following these 12 days of allocated study treatment (i.e., after approximately 4 weeks of total antibiotic treatment), treatment strategy may be further individualised (e.g., cease therapy, continue IV antibiotic, change to oral antibiotic, or continue oral antibiotic therapy). Data on the receipt and route of antibiotics will be collected.

8.4. Concomitant care

Participants will continue to receive all other routine clinical care. In the event that additional antibiotics are indicated (whether intravenous or oral) for a new or worsening infection, this will be at the discretion of the treating clinical team and will not constitute a protocol deviation. Concomitant antibiotics administered will be documented during the index hospital admission.

Adherence counselling should be undertaken according to local protocols. Adherence counselling should aim to ensure that participants understand the indication for treatment; the dose, route and frequency of antibiotic administration; the expected duration of treatment; potential side effects and what to do if these occur; and the monitoring required. Adjuncts to adherence such as mobile phone apps, patient diaries, pill count systems etc. could be considered if this is part of standard practice at participating sites.

Patient monitoring and follow-up

In addition to protocolised SNAP study follow-up, sites should continue to provide follow-up for SAB patients according to standard local protocols. The follow-up provided to SNAP participants randomised to oral antibiotics in the early oral switch domain should be of similar intensity to that provided to SAB patients on continued IV treatment. At a minimum, it is recommended that the clinical progress of patients be reviewed weekly while on antibiotic therapy (whether IV or oral), including assessments of infection-related symptoms, antibiotic-related side effects and complications, intravenous line-related complications, and adherence to antibiotic therapy. Adherence should be assessed according to local practice (e.g. OPAT monitoring, pill counts at clinic review etc.). Answers to a protocolised adherence question at Day 14 and Day 28 will also be collected (see 9.1.4 'Domain-specific study visit day details').

8.5. Endpoints

8.5.1. Primary endpoint

The primary endpoint for this domain is the platform primary endpoint (all-cause mortality at 90 days after platform entry) as specified in Core Protocol Section 6.8.

8.5.2. Secondary endpoints

All secondary platform endpoints as specified in the Core Protocol Section 6.8 apply to the early oral switch domain.

The domain-specific secondary endpoints are:

1. Number of days of IV antibiotic therapy in the total index hospitalisation (which includes OPAT), starting from platform entry, for those surviving until hospital discharge
2. Number of days alive and free of antibiotics by Day 42 from platform entry
    a. For all antibiotics
    b. For IV antibiotics
3. Peripherally inserted central catheter (PICC)/other central venous catheter complications requiring line removal, during the total index hospitalisation (which includes OPAT), starting from platform entry
    a. This outcome will be collected at total index hospital/OPAT discharge as a Y/N question. It will include any of the following: catheter-related blood stream infection; exit site infection; catheter-related superficial or deep venous thrombosis/thrombophlebitis, catheter blockage. It will NOT include PICC line rupture, leakage, displacement, or splitting unless it results in or occurs in addition to one of the above events.
4. Clinician-initiated change in treatment strategy from allocated EOS domain intervention (e.g. changed to IV antibiotics when allocated to oral antibiotics or vice versa) from reveal of EOS allocation until platform day 28 due to an adverse event deemed by the treating doctor/team to be of sufficient severity to change strategy.
5. Clinician-initiated change in treatment strategy from allocated EOS domain intervention (e.g. changed to IV antibiotics when allocated to oral antibiotics or vice versa) from reveal of EOS allocation until platform day 28 due to presumed lack of efficacy of strategy according to the treating doctor/team.

9. TRIAL CONDUCT

9.1. Domain-specific data collection

9.1.1. Microbiology

No specific additional microbiological testing will be performed for this domain. No additional microbiological data will be collected for this domain. Oral antibiotic choice for individual patients should be based on laboratory susceptibilities of the infecting *S. aureus* isolate.

9.1.2. Clinical data and sample collection

Additional domain-specific data will be collected:

- All participants in the platform should have eligibility screening completed at platform Day 7 and Day 14 (if not eligible at Day 7)
- Antibiotics prescribed (name, route, number of days):
    - During total index hospitalisation (collect data during hospitalisation and at hospital discharge)
    - Between index hospitalisation and platform Day 42 (data collection can occur up to Day 48)
    - Reasons for changes to the allocated strategy will be collected:
        - 1) clinical failure / poor efficacy; 2) drug-related side effects; 3) drug interaction-related complications; 4) PICC/central venous catheter related complications; 5) problems with administration/dosing/adherence; 6) patient preference; 7) other; 8) unknown
- Adherence
    - Assessment of adherence to treatment will be collected at platform Day 14 and Day 28 via review of medical notes and medication administration records or patient interview, to assess the number of days in the past week the patient has missed at least one dose of their antibiotics (0-1, 2-3, or >3).
- PICC/central venous catheter-related complications occurring up until total index hospital discharge (includes OPAT):
    - This outcome will be collected at total index hospital/OPAT discharge as a Y/N question. It will include any of the following: catheter-related blood stream infection; exit site infection; catheter-related superficial or deep venous thrombosis/ thrombophlebitis. It will NOT include PICC line rupture, leakage, displacement, or splitting unless it results in or occurs in addition to one of the above events.

9.1.3. Domain-specific study timeline

Table 3. Domain-specific schedule of visits and follow-up.

Platform Day	Day 1	Day 7 (±2)	Day 14 (±2)	Acute D/C	Total D/C[a]	Day 21 (±3)	Day 28 (±3)	Day 35 (±3)	Day 42 (±3)
Consent	X								
Eligibility assessment for EOS		X	X						
Allocation reveal if eligible		X	X						
Data on antibiotics +/- adherence			X[b]	X	X		X[b]		X[b]
Check clinical progress[c]		X	X			X	X	X	X

[a] Total discharge means the end of the total index hospital admission, which includes both inpatient and OPAT/HITH/rehab stay

[b] If participant is discharged at this timepoint, collect data from medical records where possible, or phone call to the participant.

[C] For those whose antibiotic treatment is ongoing, check that treating team have spoken to or seen the patient, to assess symptoms of infection, oral antibiotic adherence and adverse effects.

9.1.4. Domain-specific study visit day details

All core study visit details are specified in the Core Protocol (Section 8.8). Additional domain-specific study procedures are outlined below.

9.1.4.1. Day 7 (+/- 2)

In addition to the screening procedures outlined in the Core Protocol (Section 8.8), additional domain-specific screening procedures will occur as per the eligibility criteria outlined in Section 8.2. This will include checking that the patient still agrees to participate in this domain.

Additional domain-specific activities will include:

- Check clinical progress (both IV and oral groups)

9.1.4.2. Day 14 (+/- 2)

In addition to the activities outlined in the Core Protocol (Section 8.8), additional domain-specific screening procedures will occur as per the eligibility criteria outlined in Section 8.2. This will include checking that the patient still agrees to participate in this domain.

Additional domain-specific activities will include:

- Collection of data on antibiotics prescribed up to Day 14
- Assessment of adherence to treatment via review of medical notes and medication administration records or patient interview to assess the number of days in the past week the patient has missed at least one dose of their antibiotics (0-1, 2-3, or >3)
- Check clinical progress (both IV and oral groups)

9.1.4.3. Total and acute hospital discharge

Core activities from Day 2 until hospital discharge are outlined in the Core Protocol (Section 8.8).

Additional domain-specific activities will include:

- Collection of data on antibiotics administered during the index hospitalisation
- Collection of data on PICC/central venous catheter-related complications

9.1.4.4. Day 21 (+/- 3 days)

In addition to the activities outlined in the Core Protocol (Section 8.8), additional domain-specific activities will be conducted, including:

- Check clinical progress (both IV and oral groups)

9.1.4.5. Day 35 (+/- 3 days)

In addition to the activities outlined in the Core Protocol (Section 8.8), additional domain-specific activities will be conducted, including:

- Check clinical progress (both IV and oral groups)

9.1.4.6. Day 28 (+/-3days)

In addition to the activities outlined in the Core Protocol (Section 8.8), additional domain-specific activities will be conducted, including:

- Collection of data on antibiotics prescribed between the time of discharge or day 14 (whichever is later) until Day 28

- Assessment of adherence to treatment via review of medical notes and medication administration records or patient interview to assess the number of days in the past week the patient has missed at least one dose of their antibiotics (0-1, 2-3, or >3)
- Check clinical progress (both IV and oral groups)

9.1.4.7. Day 42-48

In addition to the activities outlined in the Core Protocol (Section 8.8), additional domain-specific activities will be conducted, including:

- Collection of data on antibiotic duration received between platform entry and Day 42
- Check clinical progress (both IV and oral groups)

9.2. Criteria for discontinuation

Refer to Core Protocol Section 8.10 for criteria for discontinuation of participation in the SNAP trial.

9.2.1. Blinding

At platform entry, participants will be randomised to early oral switch or continued IV therapy strategies. Investigators and participants will remain blinded to the allocation until the participant is judged to be eligible at platform **Day 7** or **Day 14** (if not eligible at Day 7). Once a participant is eligible, the allocation will be revealed and the investigator and participant will be unblinded. It is not practical for placebo oral or placebo IV infusions to be provided in this pragmatic trial, particularly given the wide range of antibiotics that could be used.

Trial investigators, site and study personnel will remain blinded to pooled domain outcomes and summaries until the DSMC has recommended terminating the cell or domain for non-inferiority or futility based on pre-specified decision criteria.

9.2.2. Unblinding

Unblinding is not relevant at the individual participant and site investigator level as once eligibility is reached, the allocation is not blinded.

10.STATISTICAL CONSIDERATIONS

10.1. *Estimands, endpoints, and intercurrent events*

10.1.1. Primary estimand

The primary estimand, endpoint, and intercurrent events strategy for this domain based on the SNAP primary endpoint (i.e. all-cause mortality 90 days after platform entry) and a treatment policy strategy, as specified in Statistical Analysis Appendix.

10.1.2. Secondary estimands

All core secondary estimands, endpoints, and intercurrent events strategies are specified in the Statistical Analysis Appendix.

The domain-specific secondary estimands, endpoints, and intercurrent events, are defined as follows:

Estimand/Objective/Target population	Endpoint/Population-level summaries	Intercurrent events strategy
Estimand C.1<br><br>To evaluate, within each relevant cell, the effect of intervention compared to the domain control, on the probability of **all-cause mortality at 90 days after platform entry**, in platform eligible participants who adhered to treatment.	Endpoint: All-cause mortality at 90 days after platform entry#.<br><br>Population summary: Log-odds ratio of the stated event between intervention and control groups within each relevant cell.	Principal stratum policy (per protocol principle), see Section 10.7.
Estimand C.2<br><br>To evaluate, within each relevant cell, the effect of the revealed randomised intervention compared to the domain control, on the **number of days of IV antibiotic therapy in the total index hospitalisation (which includes OPAT) following platform entry for patients**	Endpoint: Count of days where IV antibiotic therapy was received in the index hospitalisation (which includes OPAT) following platform entry<br><br>Population summary: Median difference in endpoint between intervention and control groups within each relevant cell	Treatment policy strategy (intent-to-treat principle)

**surviving until hospital discharge,** in domain eligible participants.		
**Estimand C.3**<br><br>To evaluate, within each relevant cell, the effect of the revealed randomised intervention compared to the domain control, on the number of **days alive and free of all antibiotics within the 42 days following platform entry,** in domain eligible participants.	Endpoint: Count of days alive and free from all antibiotics within the 42 days following platform entry. Participants who die within 42 days of platform entry will be recorded as 42 days, as they did not meet the survival criteria for this endpoint.<br><br>Population summary: As for estimand C.2.	Treatment policy strategy (intent-to-treat principle) and composite endpoint
**Estimand C.4**<br><br>To evaluate, within each relevant cell, the effect of the revealed randomised intervention compared to the domain control, on the number of **days alive and free of IV antibiotics within the 42 days following platform entry,** in domain eligible participants.	Endpoint: Count of days alive and free from IV antibiotics within the 42 days following platform entry. Participants who die within 42 days of platform entry will be recorded as 42 days, as they did not meet the survival criteria for this endpoint.<br><br>Population summary: As for estimand C.2.	Treatment policy strategy (intent-to-treat principle) and composite endpoint
**Estimand C.5**<br><br>To evaluate, within each relevant cell, the effect of the revealed randomised intervention compared to the domain control, on the **probability of peripherally inserted central catheter/other central venous catheter complications requiring line removal, during the total index hospitalisation (which includes OPAT) following platform entry,** in domain eligible participants.	Endpoint: An event defined by any of the following during total index hospitalisation, starting from platform entry:<br><br>- catheter-related blood stream infection<br>- exit site infection<br>- catheter-related superficial or deep venous thrombosis/thrombophlebitis<br>- catheter blockage.<br><br>It will NOT include PICC line rupture, leakage, displacement, or splitting unless it results in or occurs in addition to one of the above events.	Treatment policy strategy (intent-to-treat principle)

	Population summary: As for estimand C.1.	
**Estimand C.6**<br><br>To evaluate, within each relevant cell, the effect of the revealed randomised intervention compared to the domain control, on the **probability of clinician-initiated change in EOS domain treatment strategy from allocated intervention,** from reveal of EOS allocation until **28 days after platform entry, due to an adverse event deemed by the treating doctor/team to be of sufficient severity to change strategy,** in domain eligible participants.	Endpoint: Clinician-initiated change in EOS domain allocated intervention, from reveal of EOS allocation until 28 days after platform entry, due to an adverse event deemed by the treating doctor/team to be of sufficient severity to change.<br><br>Population summary: As for estimand C.1.	Treatment policy strategy (intent-to-treat principle)
**Estimand C.7**<br><br>To evaluate, within each relevant cell, the effect of the revealed randomised intervention compared to the domain control, on the **probability of clinician-initiated change in EOS domain treatment strategy from allocated intervention,** from reveal of EOS allocation until **28 days after platform entry, due to presumed lack of efficacy of strategy according to the treating doctor/team,** in domain eligible participants.	Endpoint: Clinician-initiated change in EOS domain allocated intervention, from reveal of EOS allocation until 28 days after platform entry, due to an perceived lack of efficacy according to the treating doctor/team.<br><br>Population summary: As for estimands C.1.	Treatment policy strategy (intent-to-treat principle)

10.2. Statistical modelling

10.2.1. Primary model

The population summary (log-odds ratio) for the binary primary endpoint (all-cause mortality at 90 days) will be modelled using a Bayesian binomial model with a logit link. See the Statistical Analysis Appendix.

10.2.2. Secondary models

The population summaries and models for all core secondary endpoints are specified in the Statistical Analysis Appendix.

The domain-specific endpoints C.1, C.5, C.6, and C.7 described in Section 10.1 are all binary and have log-odds population summaries that will be modelled using a Bayesian binomial model with a logit link. The domain-specific endpoints C.2, C.3, and C.4 described in Section 10.1 are continuous and, after any necessary transformations, will be modelled using a Bayesian linear model with normally distributed errors. See the Statistical Analysis Appendix.

10.3. Decision criteria

Stopping decisions in each cell in this domain are based on whether the posterior probabilities that the domain intervention is **non-inferior,** with respect to the domain control, is above, or below, pre-specified thresholds. Where a cell stopping decision is recommended because a posterior probability is below the pre-specified threshold, we say that it is futile to continue with the objective of demonstrating non-inferiority.

The primary objective for this domain is to determine if early oral switch is non-inferior to continued IV antibiotic treatment. Non-inferiority is defined as an OR < 1.2 for the primary endpoint (where an OR > 1 indicates an increase in mortality for early oral switch treatment compared to continued IV antibiotic treatment). A cell stopping decision will be recommended for non-inferiority if, at a pre-specified interim analysis, the posterior probability of non-inferiority for the primary estimand in that cell is greater than 99%. A cell stopping decision will be recommended of futility if, at a pre-specified interim analysis, the posterior probability of non-inferiority for the primary estimand in that cell is less than 1%.

Stopping decisions within this domain are made with respect to silos, i.e. stopping decisions are made about whether to continue random treatment allocation at the cell level. Stopping decisions in this

domain use population parameters in each silo specific 'cell' (a combination of silo and a domain) which will be estimated using Bayesian hierarchical models that allow information about the treatment effects in one silo to inform inference in the other silos within the same domain (i.e. information borrowing).

If, at any interim analysis, the thresholds for the decision criteria are not met within a cell, then recruitment into the cell will continue. In all other respects the decision criteria for this domain are those outlined in the Core Protocol (Section 9.12) and the Statistical Appendix.

10.4. Randomisation

Participants will be randomised at platform entry in a fixed 1:1 ratio within each of three silos. A participant's allocated intervention will be revealed at the time that domain-specific eligibility criteria are met at either platform Day 7 (+/- 2 days) or Day 14 (+/- 2 days). Participants judged to be not eligible at Day 7 will be reassessed at Day 14. Response adaptive randomisation may be applied if additional interventions within this domain are included in future versions of this DSA.

If participants are not eligible at platform Day 7 or at Day 14, then treatment is continued according to the treating clinician's discretion.

10.5. Interactions with other domains

An *a priori* interaction with the antibiotic backbone domain is not considered likely and will not be incorporated into the statistical models used to analyse this domain.

An *a priori* interaction with the adjunctive treatment domain is not considered likely and will not be incorporated into the statistical models used to analyse this domain.

10.6. Pre-specified secondary analyses

Pre-specified secondary analyses on the primary estimand will be performed by modifying the primary statistical model to include the following covariates and their treatment interactions:

1. Time at which eligibility occurred from platform entry
    a. Patients eligible for early oral switch at Day 7
        i. Given these patients will have uncomplicated disease, the mortality for these patients is likely to be lower than those not eligible at Day 7. Non-inferiority

may be more likely in this group also as these are low-risk patients who may even be safely treated without any further antibiotics.

b. Patients not eligible for early oral switch at Day 7 and eligible at Day 14
    i. Given these patients will have complicated disease, the mortality for these patients is likely to be higher than for those eligible at Day 7. There are fewer data and currently enrolling trials in this group (RODEO-1 is focused on endocarditis (24)) compared with the uncomplicated SAB group, where at least two trials are in progress (SABATO (22) and SAB7 (23)). Subgroup analyses of comparative efficacy of oral switch in both uncomplicated and complicated SAB will be important to understand which patients the SNAP trial findings should be applied to.

2. Endocarditis
    a. Left-sided endocarditis
        i. The group of patients with left-sided endocarditis is often more difficult to treat, being more likely to require valve surgery and prolonged treatment. The POET trial (12) involved patients with left-sided endocarditis. The primary outcome occurred in 6/87 patients (7%) with left-sided *S. aureus* IE, with no difference demonstrated between those who received early oral switch and those who received continued IV treatment.
    b. Right-sided endocarditis
        i. This is a specific population group (right-sided IE often occurs in people who inject drugs). Previous studies suggest that shorter treatment duration and early oral switch are safe in this group.
    c. No endocarditis

3. Clinician-intended oral antibiotic regimen if participant eligible for EOS domain, identified prior to reveal of EOS domain allocation. This will be captured at the time of oral switch. Where >1 oral antibiotic is prescribed, all prescribed antibiotics should be selected. Analyses will assess individual antibiotics as well as frequently used combinations and will be described in the statistical analysis plan. It may consist of:
    a. β-lactams
    b. Linezolid
    c. Rifampicin (rifampin)
    d. Quinolones

e. Trimethoprim-sulfamethoxazole
f. Clindamycin
g. Probenecid

10.7. *Principal stratum policy*

The principal stratum policy (**also known as a 'per protocol principle'**) for Estimand C.1 uses the population as described in the following subsections.

10.7.1. If allocated to early oral switch

If allocated at day 7, received ≥ 5 days of exclusively oral antibiotics during platform days 8-14 inclusive, or if allocated at day 14, received ≥ 10 days of exclusively oral antibiotics during platform days 15-28 inclusive.

10.7.2. If allocated to continued IV treatment

If allocated at day 7 to continued IV treatment, received ≥ 5 days IV antibiotics during platform days 8-14, or if allocated at day 14 to continued IV treatment, received ≥12 days IV antibiotics during platform days 15-28 inclusive.

11. ETHICAL CONSIDERATIONS

11.1. *Data Safety and Monitoring Committee (DSMC)*

The statistical analyses will evaluate non-inferiority and futility for non-inferiority (which may incorporate inferiority), with pre-specified stopping rules. The DSMC should be aware of the pre-specified decision criteria for non-inferiority and futility of early oral switch to oral antibiotics compared to continued IV antibiotics and may consider recommending stopping or changing aspects of the protocol if harm is demonstrated in the incidence of serious adverse reactions and for the secondary outcomes.

11.2. *Potential domain-specific adverse events*

Serious adverse reactions (SARs) should be captured in all participants in this domain irrespective of intervention allocation. As this domain tests a strategy rather than specific agents, SARs related to intervention strategy include:

- Antibiotic-related (serious adverse reactions): anaphylaxis, angioedema, drug rash with eosinophilia and systemic symptoms (DRESS), Stevens Johnson syndrome (SJS),

toxic epidermal necrolysis (TEN), acute generalised exanthematous pustulosis (AGEP), acute interstitial nephritis (AIN), drug-induced liver injury (DILI), serum sickness, beta-lactam-associated neutropenia

- Serious adverse events related to PICC/other central venous catheter complications requiring line removal
- Drug-drug interactions resulting in serious harm e.g. that are life-threatening or lead to death or permanent disability, hospital readmission or prolongation of admission

Other events should be recorded only where, in the opinion of the site investigator, the event might reasonably have occurred as a consequence of a study intervention strategy (i.e. if it is a serious adverse reaction, see Core protocol Section 10).

11.3. Domain-specific consent issues

Consent for the early oral switch domain will be taken at platform entry, along with consent for the platform and other domains. Patient agreement to participate in this domain will be re-confirmed at the time of eligibility assessment for this domain at platform Day 7 and / or Day 14, and re-confirmation of consent will be recorded in the database. If the patient no longer agrees to participate in the early oral switch domain, he/she will not be considered eligible at that time point. It may be helpful to discuss with the patient the continued IV treatment versus early oral switch allocations in advance of eligibility assessment, to allow for hospital discharge planning and to remind the participant of discussions at platform entry.

Participants will be made aware that randomisation to continued IV treatment within the early oral switch domain will not necessarily result in them staying in hospital for this treatment at sites where an OPAT program is available. Conversely, certain patient populations (e.g., PWID) will be made aware that randomisation to continued IV treatment may result in prolongation of their hospital stay (PWID are often not eligible for OPAT due to the risks of having long-term IV access in the community).

12.GOVERNANCE ISSUES

12.1. Funding of domain

Funding sources for the SNAP trial are specified in the Core Protocol Section 2.5. This domain has not received any additional domain-specific funding.

12.2. Funding of domain interventions and outcome measures

This domain and the interventions included have not received any additional domain-specific funding.

12.3. Domain-specific declarations of interest

All investigators involved in SNAP maintain a registry of interests on the SNAP website. These are updated periodically and publicly accessible on the study website.

13.REFERENCES

1. MacGregor RR, Graziani AL. Oral administration of antibiotics: a rational alternative to the parenteral route. Clin Infect Dis. 1997;24(3):457-67.

2. Fowler VG, Jr., Li J, Corey GR, Boley J, Marr KA, Gopal AK, et al. Role of echocardiography in evaluation of patients with Staphylococcus aureus bacteremia: experience in 103 patients. Journal of the American College of Cardiology. 1997;30(4):1072-8.

3. Rasmussen RV, Host U, Arpi M, Hassager C, Johansen HK, Korup E, et al. Prevalence of infective endocarditis in patients with Staphylococcus aureus bacteraemia: the value of screening with echocardiography. Eur J Echocardiogr. 2011;12(6):414-20.

4. Holland TL, Raad I, Boucher HW, Anderson DJ, Cosgrove SE, Aycock PS, et al. Effect of Algorithm-Based Therapy vs Usual Care on Clinical Success and Serious Adverse Events in Patients with Staphylococcal Bacteremia: A Randomized Clinical Trial. JAMA. 2018;320(12):1249-58.

5. Ringberg H, Thoren A, Lilja B. Metastatic complications of Staphylococcus aureus septicemia. To seek is to find. Infection. 2000;28(3):132-6.

6. Naber CK, Baddour LM, Giamarellos-Bourboulis EJ, Gould IM, Herrmann M, Hoen B, et al. Clinical consensus conference: survey on Gram-positive bloodstream infections with a focus on Staphylococcus aureus. Clin Infect Dis. 2009;48 Suppl 4:S260-70.

7. Eichenberger EM, Fowler VG, Jr., Holland TL. Duration of antibiotic therapy for Staphylococcus aureus bacteraemia: the long and the short of it. Clin Microbiol Infect. 2020;26(5):536-8.

8. Liu C, Bayer A, Cosgrove SE, Daum RS, Fridkin SK, Gorwitz RJ, et al. Clinical practice guidelines by the infectious diseases society of america for the treatment of methicillin-resistant Staphylococcus aureus infections in adults and children. Clinical infectious diseases : an official publication of the Infectious Diseases Society of America. 2011;52(3):e18-55.

9. Mermel LA, Allon M, Bouza E, Craven DE, Flynn P, O'Grady NP, et al. Clinical practice guidelines for the diagnosis and management of intravascular catheter-related infection: 2009 Update by the Infectious Diseases Society of America. Clin Infect Dis. 2009;49(1):1-45.

10. Baddour LM, Wilson WR, Bayer AS, Fowler VG, Jr., Tleyjeh IM, Rybak MJ, et al. Infective Endocarditis in Adults: Diagnosis, Antimicrobial Therapy, and Management of Complications: A Scientific Statement for Healthcare Professionals From the American Heart Association. Circulation. 2015;132(15):1435-86.

11. Habib G, Lancellotti P, Antunes MJ, Bongiorni MG, Casalta JP, Del Zotti F, et al. 2015 ESC Guidelines for the management of infective endocarditis: The Task Force for the Management of Infective Endocarditis of the European Society of Cardiology (ESC). Endorsed by: European Association for Cardio-Thoracic Surgery (EACTS), the European Association of Nuclear Medicine (EANM). Eur Heart J. 2015;36(44):3075-128.

12. Iversen K, Ihlemann N, Gill SU, Madsen T, Elming H, Jensen KT, et al. Partial Oral versus Intravenous Antibiotic Treatment of Endocarditis. The New England journal of medicine. 2019;380(5):415-24.

13. Dagher M, Fowler VG, Jr., Wright PW, Staub MB. A Narrative Review of Early Oral Stepdown Therapy for the Treatment of Uncomplicated Staphylococcus aureus Bacteremia: Yay or Nay? Open Forum Infect Dis. 2020;7(6):ofaa151.

14. Perez-Rodriguez MT, Sousa A, Moreno-Flores A, Longueira R, Dieguez P, Suarez M, et al. The benefits and safety of oral sequential antibiotic therapy in non-complicated and complicated Staphylococcus aureus bacteremia. International journal of infectious diseases : IJID : official publication of the International Society for Infectious Diseases. 2021;102:554-60.

15. Bupha-Intr O, Blackmore T, Bloomfield M. Efficacy of Early Oral Switch with beta-Lactams for Low-Risk Staphylococcus aureus Bacteremia. Antimicrob Agents Chemother. 2020;64(7).

16. Kaasch AJ, Rommerskirchen A, Hellmich M, Fatkenheuer G, Prinz-Langenohl R, Rieg S, et al. Protocol update for the SABATO trial: a randomized controlled trial to assess early oral switch therapy in low-risk Staphylococcus aureus bloodstream infection. Trials. 2020;21(1):175.

17. Hospenthal DR, Waters CD, Beekmann SE, Polgreen PM. Practice Patterns of Infectious Diseases Physicians in Transitioning From Intravenous to Oral Therapy in Patients With Bacteremia. Open Forum Infect Dis. 2020;7(12):ofz386.

18. Tissot-Dupont H, Gouriet F, Oliver L, Jamme M, Casalta JP, Jimeno MT, et al. High-dose trimethoprim-sulfamethoxazole and clindamycin for Staphylococcus aureus endocarditis. Int J Antimicrob Agents. 2019;54(2):143-8.

19. Al-Hasan MN, Rac H. Transition from intravenous to oral antimicrobial therapy in patients with uncomplicated and complicated bloodstream infections. Clin Microbiol Infect. 2020;26(3):299-306.

20. Jorgensen SCJ, Lagnf AM, Bhatia S, Shamim MD, Rybak MJ. Sequential intravenous-to-oral outpatient antibiotic therapy for MRSA bacteraemia: one step closer. J Antimicrob Chemother. 2019;74(2):489-98.

21. Willekens R, Puig-Asensio M, Ruiz-Camps I, Larrosa MN, Gonzalez-Lopez JJ, Rodriguez-Pardo D, et al. Early Oral Switch to Linezolid for Low-risk Patients With Staphylococcus aureus Bloodstream Infections: A Propensity-matched Cohort Study. Clin Infect Dis. 2019;69(3):381-7.

22. Kaasch AJ, Fatkenheuer G, Prinz-Langenohl R, Paulus U, Hellmich M, Weiss V, et al. Early oral switch therapy in low-risk Staphylococcus aureus bloodstream infection (SABATO): study protocol for a randomized controlled trial. Trials. 2015;16:450.

23. Thorlacius-Ussing L, Andersen CO, Frimodt-Moller N, Knudsen IJD, Lundgren J, Benfield TL. Efficacy of seven and fourteen days of antibiotic treatment in uncomplicated Staphylococcus aureus bacteremia (SAB7): study protocol for a randomized controlled trial. Trials. 2019;20(1):250.

24. Lemaignen A, Bernard L, Tattevin P, Bru JP, Duval X, Hoen B, et al. Oral switch versus standard intravenous antibiotic therapy in left-sided endocarditis due to susceptible staphylococci, streptococci or enterococci (RODEO): a protocol for two open-label randomised controlled trials. BMJ Open. 2020;10(7):e033540.

25. Heyd A, Haverstock D. Retrospective analysis of the safety profile of oral and intravenous ciprofloxacin in a geriatric population. Clinical therapeutics. 2000;22(10):1239-50.

26. Keren R, Shah SS, Srivastava R, Rangel S, Bendel-Stenzel M, Harik N, et al. Comparative effectiveness of intravenous vs oral antibiotics for postdischarge treatment of acute osteomyelitis in children. JAMA Pediatr. 2015;169(2):120-8.

27. Keller SC, Dzintars K, Gorski LA, Williams D, Cosgrove SE. Antimicrobial Agents and Catheter Complications in Outpatient Parenteral Antimicrobial Therapy. Pharmacotherapy. 2018;38(4):476-81.

28. Allison GM, Muldoon EG, Kent DM, Paulus JK, Ruthazer R, Ren A, et al. Prediction model for 30-day hospital readmissions among patients discharged receiving outpatient parenteral antibiotic therapy. Clin Infect Dis. 2014;58(6):812-9.

29. Spires SS, Rebeiro PF, Miller M, Koss K, Wright PW, Talbot TR. Medically Attended Catheter Complications Are Common in Patients With Outpatient Central Venous Catheters. Infect Control Hosp Epidemiol. 2018;39(4):439-44.

30. Waagsbo B, Sundoy A, Paulsen EQ. Reduction of unnecessary i.v. antibiotic days using general criteria for antibiotic switch. Scand J Infect Dis. 2008;40(6-7):468-73.

31. Mouwen AMA, Dijkstra JA, Jong E, Buijtels P, Pasker-de Jong PCM, Nagtegaal JE. Early switching of antibiotic therapy from intravenous to oral using a combination of education, pocket-sized cards and switch advice: A practical intervention resulting in reduced length of hospital stay. Int J Antimicrob Agents. 2020;55(1):105769.

32. Lovering AM, Zhang J, Bannister GC, Lankester BJ, Brown JH, Narendra G, et al. Penetration of linezolid into bone, fat, muscle and haematoma of patients undergoing routine hip replacement. J Antimicrob Chemother. 2002;50(1):73-7.

33. Wald-Dickler N, Holtom P, Spellberg B. Busting the Myth of "Static vs Cidal": A Systemic Literature Review. Clin Infect Dis. 2018;66(9):1470-4.

34. Nemeth J, Oesch G, Kuster SP. Bacteriostatic versus bactericidal antibiotics for patients with serious bacterial infections: systematic review and meta-analysis. J Antimicrob Chemother. 2015;70(2):382-95.

35. Holland TL. Early Oral Antibiotic Switch for Staphylococcus aureus Bacteremia: Many Are Called, but Few Are Chosen. Antimicrob Agents Chemother. 2020;64(7).

36. Spellberg B, Chambers HF, Musher DM, Walsh TL, Bayer AS. Evaluation of a Paradigm Shift From Intravenous Antibiotics to Oral Step-Down Therapy for the Treatment of Infective Endocarditis: A Narrative Review. JAMA internal medicine. 2020;180(5):769-77.

37. Li HK, Rombach I, Zambellas R, Walker AS, McNally MA, Atkins BL, et al. Oral versus Intravenous Antibiotics for Bone and Joint Infection. The New England journal of medicine. 2019;380(5):425-36.

38. Moise PA, Forrest A, Birmingham MC, Schentag JJ. The efficacy and safety of linezolid as treatment for Staphylococcus aureus infections in compassionate use patients who are intolerant of, or who have failed to respond to, vancomycin. J Antimicrob Chemother. 2002;50(6):1017-26.

39. Birmingham MC, Rayner CR, Meagher AK, Flavin SM, Batts DH, Schentag JJ. Linezolid for the treatment of multidrug-resistant, gram-positive infections: experience from a compassionate-use program. Clin Infect Dis. 2003;36(2):159-68.

40. Shorr AF, Kunkel MJ, Kollef M. Linezolid versus vancomycin for Staphylococcus aureus bacteraemia: pooled analysis of randomized studies. J Antimicrob Chemother. 2005;56(5):923-9.

41. Stevens DL, Herr D, Lampiris H, Hunt JL, Batts DH, Hafkin B. Linezolid versus vancomycin for the treatment of methicillin-resistant Staphylococcus aureus infections. Clin Infect Dis. 2002;34(11):1481-90.

42. Wilcox MH, Tack KJ, Bouza E, Herr DL, Ruf BR, Ijzerman MM, et al. Complicated skin and skin-structure infections and catheter-related bloodstream infections: noninferiority of linezolid in a phase 3 study. Clin Infect Dis. 2009;48(2):203-12.

43. Usery JB, Vo NH, Finch CK, Cleveland KO, Gelfand MS, Self TH. Evaluation of the treatment of methicillin-resistant Staphylococcus aureus bacteremia. Am J Med Sci. 2015;349(1):36-41.

44. Wilcox M, Nathwani D, Dryden M. Linezolid compared with teicoplanin for the treatment of suspected or proven Gram-positive infections. J Antimicrob Chemother. 2004;53(2):335-44.

45. Beganovic M, Cusumano JA, Lopes V, LaPlante KL, Caffrey AR. Comparative Effectiveness of Exclusive Exposure to Nafcillin or Oxacillin, Cefazolin, Piperacillin/Tazobactam, and Fluoroquinolones Among a National Cohort of Veterans With Methicillin-Susceptible Staphylococcus aureus Bloodstream Infection. Open Forum Infect Dis. 2019;6(7):ofz270.

46. Heldman AW, Hartert TV, Ray SC, Daoud EG, Kowalski TE, Pompili VJ, et al. Oral antibiotic treatment of right-sided staphylococcal endocarditis in injection drug users: prospective randomized comparison with parenteral therapy. The American journal of medicine. 1996;101(1):68-76.

47. Schrenzel J, Harbarth S, Schockmel G, Genne D, Bregenzer T, Flueckiger U, et al. A randomized clinical trial to compare fleroxacin-rifampicin with flucloxacillin or vancomycin for the treatment of staphylococcal infection. Clin Infect Dis. 2004;39(9):1285-92.

48. Paul M, Bishara J, Yahav D, Goldberg E, Neuberger A, Ghanem-Zoubi N, et al. Trimethoprim-sulfamethoxazole versus vancomycin for severe infections caused by meticillin resistant Staphylococcus aureus: randomised controlled trial. BMJ. 2015;350:h2219.

49. Markowitz N, Quinn EL, Saravolatz LD. Trimethoprim-sulfamethoxazole compared with vancomycin for the treatment of Staphylococcus aureus infection. Annals of internal medicine. 1992;117(5):390-8.

50. Goldberg E, Paul M, Talker O, Samra Z, Raskin M, Hazzan R, et al. Co-trimoxazole versus vancomycin for the treatment of methicillin-resistant Staphylococcus aureus bacteraemia: a retrospective cohort study. J Antimicrob Chemother. 2010;65(8):1779-83.

51. Harbarth S, von Dach E, Pagani L, Macedo-Vinas M, Huttner B, Olearo F, et al. Randomized non-inferiority trial to compare trimethoprim/sulfamethoxazole plus rifampicin versus linezolid for the treatment of MRSA infection. J Antimicrob Chemother. 2015;70(1):264-72.

52. Everts RJ, Begg R, Gardiner SJ, Zhang M, Turnidge J, Chambers ST, et al. Probenecid and food effects on flucloxacillin pharmacokinetics and pharmacodynamics in healthy volunteers. The Journal of infection. 2020;80(1):42-53.

53. Craig WA. Pharmacokinetic/pharmacodynamic parameters: rationale for antibacterial dosing of mice and men. Clin Infect Dis. 1998;26(1):1-10; quiz 1-2.

54. Turnidge JD. The pharmacodynamics of beta-lactams. Clin Infect Dis. 1998;27(1):10-22.

55. Gardiner SJ, Drennan PG, Begg R, Zhang M, Green JK, Isenman HL, et al. In healthy volunteers, taking flucloxacillin with food does not compromise effective plasma concentrations in most circumstances. PloS one. 2018;13(7):e0199370.

56. Thwaites GE, United Kingdom Clinical Infection Research G. The management of Staphylococcus aureus bacteremia in the United Kingdom and Vietnam: a multi-centre evaluation. PloS one. 2010;5(12):e14170.

57. Mzabi A, Kerneis S, Richaud C, Podglajen I, Fernandez-Gerlinger MP, Mainardi JL. Switch to oral antibiotics in the treatment of infective endocarditis is not associated with increased risk of mortality in non-severely ill patients. Clin Microbiol Infect. 2016;22(7):607-12.

58. Ledergerber B, Bettex JD, Joos B, Flepp M, Luthy R. Effect of standard breakfast on drug absorption and multiple-dose pharmacokinetics of ciprofloxacin. Antimicrob Agents Chemother. 1985;27(3):350-2.

59. Thwaites GE, Scarborough M, Szubert A, Nsutebu E, Tilley R, Greig J, et al. Adjunctive rifampicin for Staphylococcus aureus bacteraemia (ARREST): a multicentre, randomised, double-blind, placebo-controlled trial. Lancet. 2018;391(10121):668-78.

60. Klepser ME, Nicolau DP, Quintiliani R, Nightingale CH. Bactericidal activity of low-dose clindamycin administered at 8- and 12-hour intervals against Staphylococcus aureus, Streptococcus pneumoniae, and Bacteroides fragilis. Antimicrob Agents Chemother. 1997;41(3):630-5.

61. Malone RS, Fish DN, Abraham E, Teitelbaum I. Pharmacokinetics of levofloxacin and ciprofloxacin during continuous renal replacement therapy in critically ill patients. Antimicrob Agents Chemother. 2001;45(10):2949-54.

62. Boak LM, Rayner CR, Grayson ML, Paterson DL, Spelman D, Khumra S, et al. Clinical population pharmacokinetics and toxicodynamics of linezolid. Antimicrob Agents Chemother. 2014;58(4):2334-43.

63. Deng J, Su LX, Liang ZX, Liang LL, Yan P, Jia YH, et al. Effects of vitamin b6 therapy for sepsis patients with linezolid-associated cytopenias: a retrospective study. Curr Ther Res Clin Exp. 2013;74:26-32.

64. Kawasuji H, Tsuji Y, Ogami C, Kimoto K, Ueno A, Miyajima Y, et al. Proposal of initial and maintenance dosing regimens with linezolid for renal impairment patients. BMC Pharmacol Toxicol. 2021;22(1):13.

65. Ho JM, Juurlink DN. Considerations when prescribing trimethoprim-sulfamethoxazole. CMAJ. 2011;183(16):1851-8.
