# Supplementary material for: The Staphylococcus aureus Network Adaptive Platform Trial Protocol: New Tools for an Old Foe
Source: Clin Infect Dis. 2022 Jun 19;75(11):2027–34. doi: 10.1093/cid/ciac476 (PMC9710697; doi:10.1093/cid/ciac476)
Supplement: ciac476_Supplementary_Data [file ciac476_supplementary_data.zip › SNAP MRSA Domain-Specific Appendix.pdf]

Domain-Specific Appendix:

Backbone Domain: MRSA Silo

Staphylococcus aureus **Network Adaptive Platform trial (SNAP)**

MRSA Treatment Domain-Specific Appendix Version 1.0 dated 01 June 2021

Summary

In this domain of the SNAP trial, participants with *Staphylococcus aureus* bacteraemia (SAB) admitted to participating hospitals will be randomised to receive one of two interventions, depending on availability and acceptability:

- Intravenous vancomycin or daptomycin
- Intravenous vancomycin or daptomycin PLUS intravenous cefazolin (7 days)

At this participating site the following interventions have been selected within this domain:

☐ Intravenous vancomycin or daptomycin

☐ Intravenous vancomycin or daptomycin PLUS intravenous cefazolin (7 days)

**SNAP: Backbone Domain MRSA Silo Summary**	
Interventions	• Intravenous vancomycin OR daptomycin<br>• Intravenous vancomycin OR daptomycin PLUS intravenous Cefazolin (2g q8h IVI for 7 days)<br>See text for dosing, including adjustments for renal function.
Silos, domains, and cells	Platform eligible participants within the MRSA silo in the backbone antibiotic domain will be analysed. The population parameters for this silo-specific 'cell' (a combination of a silo and a domain) will be estimated and reported.
Evaluable treatment-by-treatment interactions	Treatment-treatment interactions are considered possible between this cell, and the cell corresponding to the MRSA silo in the adjunctive treatment domain.
Randomisation	Participants will be randomised at platform entry in a fixed 1:1 ratio within the cell. A patient's allocated intervention will be revealed at the time that both MRSA is microbiologically identified and domain-specific eligibility criteria are confirmed. Response adaptive randomization may be applied if additional interventions within this cell are added in future versions of this DSA.
Domain Specific Inclusions	Inclusion criteria are the same as the Platform (see Core Protocol Section 6.5)<br>and<br>1. MRSA confirmed microbiologically
Domain Specific Exclusions	Patients will be excluded from this domain if they meet any of the following criteria at the time of domain eligibility assessment:<br>1. >72 hours have elapsed from the time of index blood culture draw<br>2. Severe allergy to any beta-lactam (including cefazolin)<br>• Anaphylaxis/angioedema or severe delayed allergy<br>3. Non-severe rash to cefazolin<br>4. Severe allergy or non-severe rash to both vancomycin AND daptomycin<br>5. Treating team deems enrolment in the domain is not in the best interests of the patient
Intervention-specific exclusions	Not applicable (there are only 2 interventions)
Endpoints	Primary platform endpoint: All-cause mortality at 90 days from platform entry.<br>Secondary platform endpoints: refer to Core Protocol Section 6.8.

	Secondary domain-specific endpoints:<br>1. Acute kidney injury (Modified KDIGO stage 1 defined as an increase in serum creatinine of ≥26.5 μmol/L from platform entry (baseline) to day 5 OR Increase in serum creatinine by 1.5 times or more the level at platform entry (baseline) within 14 days of platform entry (patients with baseline end stage kidney disease excluded)<br>2. Renal replacement therapy at any stage up to platform day 90 (patients with baseline end stage kidney disease excluded)<br>3. Ongoing renal replacement therapy at platform day 90 (patients with baseline end stage kidney disease excluded)<br>4. Persistent bacteraemia at platform day 2<br>5. Persistent bacteraemia at platform day 5<br>6. Change in backbone antibiotic therapy (vancomycin or daptomycin) in the 14 days following platform entry due to an adverse event deemed by the treating doctor/team to be of sufficient severity to change therapy<br>7. Change in backbone antibiotic therapy (vancomycin or daptomycin) in the 14 days following platform entry due to presumed lack of efficacy according to the treating doctor/team<br>8. Cessation of allocated intervention before the end of platform day 7 due to an adverse event deemed by the treating doctor/team to be of sufficient severity to change therapy
Decision criteria	The primary objective for this cell is to determine if the cell intervention is **superior** to the cell control. Superiority is defined as an OR < 1 for the primary endpoint (where an OR > 1 indicates an increase in mortality for the intervention compared to the control). A cell stopping decision will be recommended for superiority if, at a pre-specified interim analysis, the posterior probability of superiority in this cell is greater than 99%.<br>A cell stopping decision of futility of the superiority objective will be recommended if, at a pre-specified interim analysis, the posterior probability of OR < 1/1.2 for the primary endpoint in this cell is less than 1%.<br>If, at any pre-specified interim analyses, the thresholds for the decision criteria are not met within a cell, then recruitment into the cell will continue.
Pre-specified secondary analyses	Pre-specified secondary analysis on the primary estimand will be performed by modifying the primary statistical model to include the following covariates and their treatment interactions:<br>1. Endocarditis<br>a. Left-sided endocarditis<br>b. Right-sided endocarditis<br>c. No endocarditis<br>2. Backbone agent (vancomycin vs. daptomycin)<br>3. End stage kidney failure at baseline yes or no<br>4. Vancomycin MIC >1 versus ≤1<br>5. MRSA sequence type

TABLE OF CONTENTS

1. ABBREVIATIONS 7

2. PROTOCOL APPENDIX STRUCTURE 8

3. MRSA TREATMENT DOMAIN-SPECIFIC APPENDIX VERSION 9

3.1. Version history 9

4. MRSA TREATMENT DOMAIN GOVERNANCE 9

4.1. Domain members 9

4.2. Contact Details 10

5. MRSA TREATMENT DOMAIN-SPECIFIC WORKING GROUP AUTHORIZATION 10

6. BACKGROUND AND RATIONALE 11

6.1. Domain definition 11

6.2. Domain-specific background 11

6.2.1. MRSA and standard therapy 11

6.2.2. Evidence for efficacy of β-lactam combination therapy for MRSA 11

6.2.3. Potential adverse effects of β-lactam combination therapy with cefazolin 12

6.2.4. Need for a clinical trial of combining cefazolin with standard therapy 12

7. DOMAIN OBJECTIVES 13

8. TRIAL DESIGN 13

8.1. Population 13

8.2. Eligibility criteria 13

8.2.1. Cell inclusion criteria 13

8.2.2. Cell exclusion criteria 14

8.2.3. Intervention exclusion criteria 14

8.3. Interventions 15

8.3.1. MRSA Backbone Domain Interventions 15

8.3.2. Vancomycin dosing 15

8.3.3. Daptomycin dosing 16

8.3.4. Cefazolin dosing in the combination arm 16

8.3.5. Timing of initiation of MRSA Backbone Domain 16

8.3.6. Duration of administration of MRSA Backbone Domain 17

8.4. Concomitant care 17

8.5. Endpoints 17

8.5.1. Primary endpoint 17

8.5.2. Secondary endpoint 18

9. TRIAL CONDUCT 19

9.1. Domain-specific data collection 19

9.1.1. Microbiology........ 19

9.1.2. Clinical data and sample collection 19

9.1.3. Domain-specific study timeline 19

9.1.4. Domain-specific study visit day details........ 20

9.1.4.1. Day 1-3 (screening for eligibility) 20

9.1.4.2. Day 1-7 20

9.1.4.3. Days 2, 5, 14 20

9.1.4.4. Day 90 21

9.2. Criteria for discontinuation 21

9.3. Blinding........ 21

9.3.1. Blinding........ 21

9.3.2. Unblinding 21

10. STATISTICAL CONSIDERATIONS 22

10.1. Estimands, endpoints, and intercurrent events 22

10.1.1. Primary estimand 22

10.1.2. Secondary estimands 22

10.2. Statistical modelling 24

10.2.1. Primary model 24

10.2.2. Secondary models 24

10.3. Decision criteria........ 25

10.4. Randomisation 25

10.5. Interactions with domains........ 25

10.6. Pre-specified secondary analyses........ 26

10.7 Principal stratum policy 27

10.6.1. If allocated to combination therapy 27

10.6.2. If allocated to standard therapy 27

11. ETHICAL CONSIDERATIONS 27

11.1. Data Safety and Monitoring Committee 27

11.2. Potential domain-specific adverse events........ 27

11.3. Domain-specific consent issues 28
12. GOVERNANCE ISSUES 28
12.1. Funding of domain 28
12.2. Funding of domain interventions and outcome measures 28
12.3. Domain-specific declarations of interest 28
13. REFERENCES 29

1. ABBREVIATIONS

AGEP	Acute generalised exanthematous pustulosis
AKI	Acute Kidney Injury
β-lactams	Beta-Lactam
BORSA	Borderline oxacillin resistant *S. aureus*
DRESS	Drug Rash with Eosinophilia and Systemic Symptoms
DSA	Domain-Specific Appendix
DSWG	Domain-Specific Working Group
DSMC	Data Safety and Monitoring Committee
EOS	Early oral switch
ESKD	End stage kidney disease
GTSC	Global Trial Steering Committee
IV	Intravenous
KDIGO	Kidney Disease Improving Global Outcomes
MRSA	Methicillin-Resistant Staphylococcus aureus
MSSA	Methicillin-Susceptible Staphylococcus aureus
RRT	Renal replacement therapy
RSA	Region-Specific Appendix
RSWG	Region-Specific Working Group
SAB	Staph aureus bacteraemia
SAR	Serious Adverse Reaction
SNAP	*Staphylococcus aureus* Network Adaptive Platform trial

2. PROTOCOL APPENDIX STRUCTURE

The structure of this protocol is different to that used for conventional trials because this trial is highly adaptive and the description of these adaptations is better understood and specified using a ‘modular’ protocol design. While all adaptations are pre-specified, the structure of the protocol is designed to allow the trial to evolve over time, for example by the introduction of new domains or interventions or both, and commencement of the trial in new geographical regions.

The protocol has multiple modules, in brief, comprising a Core Protocol (overview and design features of the study), a Statistical Analysis Appendix (details of the current statistical analysis plan and models, including simulations to support trial design), multiple Domain-Specific Appendices (DSA) (detailing all interventions currently being studied in each domain), and multiple Region-Specific Appendices (RSA) (detailing regional management and governance).

The Core Protocol contains all information that is generic to the trial, irrespective of the regional location in which the trial is conducted and the domains or interventions that are being tested. The Core Protocol may be amended but it is anticipated that such amendments will be infrequent.

The Core Protocol does not contain information about the intervention(s) within each domain, because one of the trial adaptations is that domains and interventions will change over time. Information about interventions, within each domain, is covered in a DSA. These Appendices are anticipated to change over time, with removal and addition of options within an existing domain, at one level, and removal and addition of entire domains, at another level. Each modification to a DSA will be subject to a separate ethics application for approval.

The Core Protocol does not contain detailed information about the statistical analysis or simulations, because the analysis model will change over time in accordance with the domain and intervention trial adaptations but this information is contained in the Statistical Analysis Appendix. These Appendices are anticipated to change over time, as trial adaptations occur. Each modification will be subject to approval from the Global Trial Steering Committee (GTSC) in conjunction with advice from the Statistical Subcommittee and the Data and Safety Monitoring Committee (DSMC).

The Core Protocol also does not contain information that is specific to a particular region in which the trial is conducted, as the locations that participate in the trial are also anticipated to increase over time. Information that is specific to each region that conducts the trial is contained within a RSA. This includes information related to local management, governance, and ethical and regulatory

aspects. It is planned that, within each region, only that region's RSA, and any subsequent modifications, will be submitted for ethical review in that region.

The current version of the Core Protocol, DSAs, RSAs, and the Statistical Analysis Appendix is listed in the Protocol Summary and on the study website (https://www.snaptrial.com.au/).

3. MRSA TREATMENT DOMAIN-SPECIFIC APPENDIX VERSION

The version of the MRSA Treatment Domain-Specific Appendix is in this document's header and on the cover page.

3.1. Version history

Version 1.0: Approved by the MRSA Domain-Specific Working Group (DSWG) on the 29th March 2021.

4. MRSA TREATMENT DOMAIN GOVERNANCE

4.1. Domain members

Chairs: David Lye

Members:
Steven Tong
Joshua Davis
Hong Foo
Matthew O'Sullivan
Jennie Johnstone
Susan Morpeth
Shirin Kalimuddin
Phil Britton
Neta Petersiel

4.2. Contact Details

Chairs:

Associate Professor David Lye

Department of Infectious Diseases

11 Jalan Tan Tock Seng

Singapore 308433

Phone (+65) 9006 6785

Email David_Lye@ttsh.com.sg

Project Manager:

Jocelyn Mora SNAP Clinical Trial Manager

The Peter Doherty Institute for Infection and Immunity

792 Elizabeth St Melbourne VIC AUSTRALIA

+61 (0) 38344 2554

jocelyn.mora@unimelb.edu.au

5. MRSA TREATMENT DOMAIN-SPECIFIC WORKING GROUP AUTHORIZATION

The MRSA Treatment Domain-Specific Working Group (DSWG) have read the appendix and authorize it as the official MRSA Treatment Domain-Specific Appendix for the SNAP trial. Signed on behalf of the committee,

Chair ______________________ Date 29 March 2021
David Lye

6. BACKGROUND AND RATIONALE

6.1. Domain definition

This is a domain within the SNAP trial to test the effectiveness of vancomycin or daptomycin plus cefazolin versus vancomycin or daptomycin alone in patients with methicillin-resistant *Staphylococcus aureus* bacteraemia who are admitted to a participating hospital.

6.2. Domain-specific background

MRSA bacteraemia is associated with high crude mortality (1) and the standard therapies of vancomycin or daptomycin have shortcomings. Better treatment strategies are needed. Studies spanning *in vitro* antimicrobial susceptibility testing to clinical trials consistently indicate combining a β-lactam with vancomycin or daptomycin leads to more rapid bacterial killing (2-4). The CAMERA2 trial demonstrated that combining vancomycin with (flu)cloxacillin reduced the duration of bacteraemia but did not reduce mortality (5). Acute kidney injury (AKI) was much more common with combination therapy. This domain will test whether combining standard therapy with cefazolin will retain the benefits of more rapid bacterial killing but without increasing the risk of acute kidney injury, and thus result in reductions in mortality.

6.2.1. MRSA and standard therapy

MRSA infections result in higher crude mortality than MSSA infections (1, 6). The current standard therapy for MRSA-B is vancomycin. Compared with β-lactams, vancomycin kills MSSA more slowly, has less penetration of infected tissue (2), and in observational studies is associated with poorer patient outcomes (6). Alternative agents to vancomycin have become available, including daptomycin and ceftaroline. However, daptomycin and ceftaroline have not been demonstrated to be superior to vancomycin for MRSA-B (7). Furthermore, these newer agents are prohibitively expensive. An additional strategy is combination antibiotic therapy.

6.2.2. Evidence for efficacy of β-lactam combination therapy for MRSA

In vitro and animal studies consistently show synergy between vancomycin and β-lactams for MRSA (2, 4). Although the combination of vancomycin and flucloxacillin or cefazolin did not reduce 90-day mortality in the CAMERA2 open-label randomised controlled trial, it reduced persistent bacteraemia at 5 days post randomisation (35/175 [20%] in standard, 19/170 [11%] in combination therapy; p = 0.02) (5).

The combination of vancomycin and cefazolin at human-simulated exposures improved the rate of kill against MRSA isolates and resulted in greater overall antibacterial effect (8). In a retrospective study of 237 patients with MRSA bacteraemia, after controlling for age, APACHE II score, infectious disease consult and infection source, vancomycin and cefazolin combination was associated with reduced odds of clinical failure (adjusted OR 0.425, 95% CI 0.228 to 0.792) (9). Beneficial combination with another cephalosporin (ceftaroline with daptomycin) versus vancomycin was also shown to be associated with reduced mortality in a phase 2 randomised controlled trial in MRSA bacteraemia (10).

6.2.3. Potential adverse effects of β-lactam combination therapy with cefazolin

CAMERA2 found that β-lactam combination therapy overall was associated with AKI. In a post-hoc assessment stratified by the β-lactam agent received, 30 (27%) of the 111 patients who received only flucloxacillin (25/90 [28%]) or cloxacillin (5/21 [24%]) developed AKI (using modified RIFLE criteria) compared with 1 (4%) of the 27 who received only cefazolin. Thus, the risk of AKI may be considerably lower when vancomycin is combined with cefazolin in comparison to combination with (flu)cloxacillin. However, this needs to be prospectively confirmed in a large number of patients.

Cefazolin is generally considered to be a well-tolerated antibiotic, which is broadly used for treatment of acute infections and for surgical prophylaxis. There are rare and mild adverse effects such as urticaria, skin reaction, diarrhea, vomiting, and transient neutropenia, and very rarely anaphylaxis. Cross reactions between cefazolin and other β-lactams are uncommon because cefazolin has a R1 side chain (the main determinant of cross reactivity) that is not shared with other registered β-lactams (11).

6.2.4. Need for a clinical trial of combining cefazolin with standard therapy

Since there is a signal of more rapid bacterial killing, and the lack of increased acute kidney injury (AKI) in patients receiving cefazolin (albeit a post-hoc finding), the next logical step is to investigate whether vancomycin plus cefazolin will improve clinically relevant outcomes. This domain will compare usual standard of care treatment with vancomycin or daptomycin with the combination of vancomycin or daptomycin plus cefazolin for patients with MRSA bacteraemia. If proven to be effective and safe, combining cefazolin with standard therapy would most likely be a cost effective and simple intervention to improve outcomes for MRSA bacteraemia.

7. DOMAIN OBJECTIVES

The objective of this domain is to determine the effectiveness of vancomycin or daptomycin plus cefazolin versus vancomycin or daptomycin alone for patients with methicillin-resistant *Staphylococcus aureus* bacteraemia (SAB) requiring admission to hospital.

We hypothesize that the probability of all-cause mortality at 90 days after platform entry will be **superior** based on the addition of cefazolin to standard therapy. The following interventions will be available:

- Intravenous vancomycin or daptomycin
- Intravenous vancomycin or daptomycin plus intravenous cefazolin

8. TRIAL DESIGN

This domain will be conducted as part of the SNAP trial (see Core Protocol Section 6). Treatment allocation will be at a fixed 1:1 ratio, as described in the Core Protocol Section 6.7.

8.1. Population

Patients with *S. aureus* bacteraemia admitted to a participating hospital.

8.2. Eligibility criteria

Patients are eligible for this domain if they meet all of the platform-level inclusion and none of the platform-level exclusion criteria (see Core Protocol Section 6.5) AND all of the cell-level inclusion and none of the cell-level exclusion criteria. Patients eligible for SNAP may have conditions that exclude them from the MRSA Backbone Treatment Domain.

8.2.1. Cell inclusion criteria

- MRSA confirmed microbiologically

Resistance to methicillin is usually associated with the presence of an alternative penicillin-binding protein, PBP2a or PBP2’ encoded by chromosomal genes *mecA* or *mecC*. Methicillin-resistance can be reliably determined by automated susceptibility testing (e.g., Vitek), phenotypic testing (e.g., disk diffusion), or molecular detection of the *mecA* gene (e.g., GeneXpert). Validated methods using any

of these techniques may be used to presumptively determine methicillin-resistance and thus eligibility for the MRSA silo.

BORSA (borderline oxacillin resistant *S. aureus)* show borderline resistance to penicillinase-resistant penicillins (e.g. oxacillin, cloxacillin and methicillin) but do not carry a modified PBP2a. For the purposes of SNAP, BORSA isolates are to be considered as MRSA.

See the Microbiology Appendix for further details.

8.2.2. Cell exclusion criteria

Patients will be excluded from this domain if they have any of the following:

1. Time to allocation reveal is >72 hours from the time of index blood culture draw
2. Severe allergy to any beta-lactam (including cefazolin)
    - Immediate severe allergy: Anaphylaxis/angioedema
    - Severe delayed allergy: Severe cutaneous adverse reaction (SCAR; including Steven Johnson Syndrome, Toxic Epidermal Necrolysis, Drug Rash with Eosinophilia and Systemic Symptoms (DRESS) and acute generalised exanthematous pustulosis (AGEP)), severe drug induced liver injury, proven allergic interstitial nephritis, immune-mediated haemolytic anaemia and other severe cytopenia.
3. Non-severe rash to cefazolin
    - Nausea, diarrhoea, headache and other non-specific symptoms are NOT allergies, they are drug intolerance, and they are not exclusion criteria. Similarly, a vague history of an allergy of unclear nature, or a family history of allergy are not exclusions.
4. Severe allergy or non-severe rash to both vancomycin AND daptomycin
    - Vancomycin infusion reaction (formerly known as “red man syndrome”) is due to direct histamine release and is not generally an allergy, and therefore is not considered an exclusion.
5. Treating team deems enrolment in the domain is not in the best interest of the patient

8.2.3. Intervention exclusion criteria

There are no intervention specific exclusions.

8.3. Interventions

8.3.1. MRSA Backbone Domain Interventions

- Intravenous vancomycin or daptomycin
- Intravenous vancomycin or daptomycin plus intravenous cefazolin 2g 8 hourly

The choice of vancomycin or daptomycin will be at the clinician's discretion. In CAMERA2, 349 of 352 patients received vancomycin and 13 of 352 receiving daptomycin. Therefore, it is expected that most patients will be prescribed vancomycin.

8.3.2. Vancomycin dosing

Sites may follow local guidelines for the use of vancomycin. In general, it would be expected that dosing follows similar principles as those in the Australian Therapeutic Guidelines: Antibiotic and the consensus guidelines from the American Society of Health-System Pharmacists, the Infectious Diseases Society of America, the Paediatric Infectious Diseases Society, and the Society of Infectious Diseases Pharmacists (12). This includes a loading dose of 25 mg/kg (up to 3000mg) if considered appropriate by the treating clinician, initial maintenance dosing at 15-20 mg/kg q12h, with subsequent adjustment to maintain area under the concentration-time curve (AUC) of 400 to 600 mg.hr/L OR trough levels at 10-20 mg/L, and the initial level taken 48-72 hours after the initiation of the first dose.

Table 1: Initial vancomycin dosing after loading, based on renal function, weight range 50 – 120kg. For weights outside this range, please seek expert advice.

**Creatinine clearance [NB1] (mL/min)**	**Starting maintenance dosage for average weight (70 kg) adults**	**Timing if trough (predose) plasma concentration measurement [NB2]**
More than 90	1.5 g 12-hourly	before the fourth dose
60 to 90	1 g 12-hourly	before the fourth dose
40 to less than 60	750 mg 12-hourly	before the fourth dose
20 to less than 40	1 g 24-hourly	before the third dose
Less than 20	1 g 48-hourly [NB3]	48 hours after the first dose
NB1: Use the Cockcroft-Gault formula (see #[Estimating glomerular filtration rate]) or online calculator #[Creatinine clearance calculator for adults] to approximate creatinine clearance.<br>NB2: If a loading dose is given, it is considered the first dose.<br>NB3: The clinical context determines whether the next dose is given before the trough concentration results is available, or withheld until the result is known.		

[1] From the Australian Therapeutic Guidelines: Principle of vancomycin use.

8.3.3. Daptomycin dosing

Although daptomycin is registered for use at 6mg/kg per day, many experts advise 8-10mg/kg per day for serious infections such as MRSA bacteraemia (13-15). Within this domain, where daptomycin is used we recommend that the higher 8-10mg/kg per day dosing be followed.

The suggested adjustment for renal function is:

GFR	Daptomycin Dose
>50ml/min	8-10mg/kg IVI q24h
11-50 ml/min	6-8mg/kg q24h IVI
<=10 but not on haemodialysis	8mg/kg q48h IVI
On continuous renal replacement therapy	8mg/kg q48h IVI
On haemodialysis	8mg/kg q48h IVI, dose after dialysis

8.3.4. Cefazolin dosing in the combination arm

The standard dose of cefazolin will be 2g IV q8h. See 'Dosing for backbone antibiotics' document for further details (including dosing in renal impairment). The summary of suggested dosing for renal impairment is:

Renal impairment	Dose
CrCl >40 ml/min	2g IV q8h
CrCl 20-40 mL/min	2g IV q12h
CrCl <20 mL/min	1g IV q24h
Continuous RRT	1g IV q8h or 2g IV q12h
Intermittent haemodialysis	2g IV after each dialysis session

8.3.5. Timing of initiation of MRSA Backbone Domain

Trial antibiotic(s) should be initiated as soon as possible following reveal of treatment allocation. If already receiving vancomycin or daptomycin, the timing of the next dose should be at the recommended interval from the last dose. If allocated to combination with cefazolin, unless already receiving cefazolin, the cefazolin should be given as soon as possible. If the patient is allocated to

combination with cefazolin and is already receiving cefazolin, the next dose should be at the recommended interval from the last dose.

8.3.6. Duration of administration of MRSA Backbone Domain

In addition to vancomycin or daptomycin, intravenous cefazolin will be added for calendar days 1 to 7 following platform entry (where platform entry is considered platform day 1). The cefazolin should be commenced as soon as the allocation within the MRSA silo is revealed as cefazolin and continue until the end of day 7 from platform entry. If the reveal of allocation is delayed (e.g., till day 2 or 3 of platform), the cefazolin will still be ceased on the completion of platform day 7.

The duration of vancomycin or daptomycin is guided by local guidelines and practice for methicillin-resistant *Staphylococcus aureus* bacteraemia.

If the patient is participating in the early oral switch domain, and is randomised to continued IV therapy, they should continue cefazolin until the end of study day 7, and continue vancomycin/daptomycin for the duration determined by the treating clinician. If they are participating in the early oral switch domain and are randomised to early oral switch, they may cease the cefazolin AND vancomycin/daptomycin at the protocol-determined time for EOS, which may be as early as study day 5. In this circumstance, there will be no oral replacement for cefazolin. Hence the duration of cefazolin may be variable depending on the day of identifying MRSA and participation and allocation within the early oral switch domain.

8.4. Concomitant care

Within the first 14 days, non-study β-lactams are discouraged as far as clinically possible. If broadening of antibiotic therapy is clinically indicated, non-β-lactam antibiotics are preferred (e.g., gram negative cover may be provided with ciprofloxacin or aminoglycoside, and anaerobic cover with metronidazole).. Ceftaroline should not be used within the first 14 days.

8.5. Endpoints

8.5.1. Primary endpoint

The primary endpoint for this domain is the platform primary endpoint (all-cause mortality at 90 days after platform entry) as specified in Core Protocol Section 6.8.

8.5.2. Secondary endpoint

All secondary platform endpoints as specified in the Core Protocol Section 6.8.

The domain-specific secondary endpoints will be:

1. Acute kidney injury defined using modified Kidney Disease Improving Global Outcomes (KDIGO) criteria.

   The Kidney Disease Improving Global Outcomes (KDIGO) guidelines for acute kidney injury (AKI) define AKI as:

   - Increase in serum creatinine by 0.3mg/dL (= 26.5 μmol/L) or more within 48 hours OR
   - Increase in serum creatinine to 1.5 times baseline or more within the last 7 days OR
   - Urine output less than 0.5 mL/kg/h for 6 hours

   For the purposes of SNAP, the modified KDIGO definition will be:

   - Increase in serum creatinine by 0.3mg/dL (= 26.5 μmol/L) or more at any time from platform entry (baseline) to day 5 OR
   - Increase in serum creatinine by 1.5 times or more the level at platform entry (baseline) within 14 days from platform entry

   As a pragmatic trial, data collected on serum creatinine will be mandated at platform entry (platform day 1 or the calendar day prior to platform entry) and days 5±1 and 14±3. Logistical complexities will make it difficult to consistently collect data for urine output.

2. Renal replacement therapy at any stage up to platform day 90 (patients with baseline end stage kidney disease excluded)
3. Ongoing renal replacement therapy at platform day 90 (patients with baseline end stage kidney disease excluded)
4. Persistent bacteraemia at platform day 2
5. Persistent bacteraemia at platform day 5 (+/-1 day)

   (patients with a negative blood culture at day 2 will be assumed to be negative at day 5 if there are no subsequent positive blood cultures from day 3 to day 5)
6. Change in backbone antibiotic therapy (vancomycin or daptomycin) in the 14 days following platform entry due to an adverse event deemed by the treating doctor/team to be of sufficient severity to change therapy

7. Change in backbone antibiotic therapy (vancomycin or daptomycin) in the 14 days following platform entry due to presumed lack of efficacy according to the treating doctor/team

8. Cessation of allocated intervention before the end of platform day 7 due to an adverse event deemed by the treating doctor/team to be of sufficient severity to change therapy

9. TRIAL CONDUCT

9.1. Domain-specific data collection

9.1.1. Microbiology

No additional microbiological testing is needed for this domain.

9.1.2. Clinical data and sample collection

Additional domain-specific data will be collected:

- Vancomycin levels per institutional therapeutic drug monitoring policies as measured by time to first therapeutic vancomycin levels. Data collected will therefore include the date and time of vancomycin doses and levels.
- Serum creatinine as clinically ordered and at days 5±1 and 14±3
- Initiation of renal replacement therapy at any time up to platform day 90

9.1.3. Domain-specific study timeline

Table 2: Domain-specific schedule of visits and follow-up, where day 1 refers to the day of platform entry

Platform Day	Day 1-3	Day 1-7	Day 2	Day 5	Day 14 (± 3 days)
Eligibility assessment on confirmation of MRSA	X				
Reveal of allocation	X				
Administration of study treatment	X[1]	X			
Serum creatinine[2]				X	X[3]
Vancomycin level			X	X	

[1] As soon as domain eligibility confirmed.
[2] Note that creatinine is being measured at platform entry as part of core protocol.
[3] Measuring serum creatinine on day 14+/3 days is only mandated during the total index hospital stay. If the patient has been discharged, it could still be collected as part of routine follow up if clinically indicated, but this is not protocol mandated.

9.1.4. Domain-specific study visit day details

All core study visit details are specified in the Core Protocol (Section 8.8). Data will be collected, as per the CRFs, on platform day 1, day 8-10 (for data from platform days 1-7), day 15-18 (for data from platform days 8-14), day 28, day 42, day 90, and acute and total discharge.

Additional domain-specific study procedures are outlined below.

9.1.4.1. Day 1-3 (screening for eligibility)

In addition to the screening procedures outlined in the Core Protocol (Section 8.8), additional domain-specific screening procedures will occur as per the eligibility criteria outlined in Section 8.2.

9.1.4.2. Day 1-7

In addition to the activities outlined in the Core Protocol (Section 8.8) additional domain-specific activities will be conducted, including:

- Ensuring appropriate dosing and administration of study drugs

9.1.4.3. Days 2, 5, 14

Core activities during the first 14 days from platform entry are outlined in the Core Protocol (Section 8.8).

Additional domain-specific activities will be conducted, including:

- Ensuring vancomycin levels are performed and recorded on days 2 and 5 (+/-1)
- Ensuring serum creatinine levels are performed and recorded on days 5 (+/-1) and 14 (+/-3)
- Reviewing and recording patient progress in relation to safety events and changes to the allocated antibiotic regimen during platform days 1-14

9.1.4.4. Day 90

In addition to the activities outlined in the Core Protocol (Section 8.8) additional domain-specific activities will be conducted, including:

- Assessment of any new or ongoing requirement for renal replacement therapy in the 90 days from platform entry, and for ongoing RRT at platform day 90

9.2. Criteria for discontinuation

Refer to Core Protocol Section 8.10 for criteria for discontinuation of participation in the SNAP trial.

9.3. Blinding

9.3.1. Blinding

At platform entry, participants will be randomized within this domain and silo to standard therapy (vancomycin or daptomycin) or combination therapy (vancomycin or daptomycin) PLUS cefazolin. Investigators and participants will remain blinded to the allocation until a microbiological identification of MRSA is made and the participant satisfies the domain eligibility criteria. Once a participant is eligible, the allocation will be revealed and the investigator and participant will be unblinded. The study drugs are used open label and therefore blinding is not relevant on a per patient basis. On a study-wide basis, investigators, site and study personnel will remain blinded to pooled domain outcomes and summaries until the DSMC has recommended terminating the cell or domain for non-inferiority, superiority or futility.

9.3.2. Unblinding

Unblinding is not relevant at the individual participant and site investigator level as once eligibility is reached, the allocation is not blinded.

10.STATISTICAL CONSIDERATIONS

10.1. Estimands, endpoints, and intercurrent events

10.1.1. Primary estimand

The primary estimand, endpoint, and intercurrent events strategy for this domain based on the SNAP primary endpoint (i.e. all-cause mortality 90 days after platform entry) and a treatment policy strategy, as specified in Statistical Analysis Appendix.

10.1.2. Secondary estimands

All core secondary estimands, endpoints, and intercurrent events strategies are specified in the Statistical Analysis Appendix.

The domain-specific secondary estimands, endpoints, and intercurrent events are defined as follows:

Estimand/Objective/Target population	Endpoint/Population-level summaries	Intercurrent events strategy
**Estimand A2.1**<br><br>To evaluate, within each relevant cell, the effect of the intervention compared to the domain control, on the probability of **all-cause mortality at 90 days after platform entry**, in platform eligible participants who adhere to treatment.	Endpoint: All-cause mortality at 90 days after platform entry#.<br><br>Population summary: Log-odds ratio of the stated event between intervention and control groups within each relevant cell.	Principal stratum policy (per protocol principle), see Section 10.7.
**Estimand A2.2**<br><br>To evaluate, within the MRSA cell, the effect of the revealed randomised intervention compared to the MRSA cell control, on the probability of **acute kidney injury defined using modified KDIGO criteria at 14 days** after **platform entry**, in platform eligible participants without end-stage kidney disease at platform entry.	Endpoint: An increase in serum creatinine by ≥0.3 mg/dl (≥26.5 μmol/l) from platform entry (baseline) to day 5 OR An increase in serum creatinine by ≥1.5 times or more the level at platform entry (baseline) at any time in the first 14 days from randomization (NB: urine volume <0.5 ml/kg/hour for 6 hours is not included in definition due to inadequate charting in most hospitals).<br><br>As a pragmatic trial, data collected on serum creatinine will be mandated at platform entry (day 1) and days 5±1 and 14±3. Logistical complexities will make it difficult to consistently collect data for urine output.<br><br>Population summary: As for estimand A2.1.	Treatment policy strategy (intent-to-treat principle)

**Estimand A2.3**<br><br>To evaluate, within the MRSA cell, the effect of revealed randomised intervention compared to the MRSA cell control, on probability of a **new requirement for renal replacement therapy (excluding prophylaxis) in the 90 days following platform entry**, in platform eligible participants without end-stage kidney disease at platform entry.	Endpoint: Clinician-indicated change in renal replacement therapy up to platform day 90.<br><br>Population summary: As for estimand A2.1.	Treatment policy strategy (intent-to-treat principle)
**Estimand A2.4**<br><br>To evaluate, within the MRSA cell, the effect of revealed randomised intervention compared to the MRSA cell control, on probability of a**n ongoing requirement for renal replacement therapy (excluding prophylaxis) at platform day 90**, in platform eligible participants without end-stage kidney disease at platform entry.	Endpoint: Clinician-indicated need for ongoing renal replacement therapy at platform day 90.<br><br>Population summary: As for estimand A2.1.	Treatment policy strategy (intent-to-treat principle)
**Estimand A2.5**<br><br>To evaluate, within the MRSA cell, the effect of revealed randomised intervention compared to the MRSA cell control, on the probability of a **persistent bacteraemia at platform day 2**, in platform eligible participants.	Endpoint: Clinician-indicated persistent bacteraemia at platform day 2.<br><br>Population summary: As for estimand A2.1.	Treatment policy strategy (intent-to-treat principle)
**Estimand A2.6**<br><br>To evaluate, within the MRSA cell, the effect of revealed randomised intervention compared to the MRSA cell control, on the probability of a **persistent bacteraemia at platform day 5 (+/- 1 day)**, in platform eligible participants.	Endpoint: Clinician-indicated persistent bacteraemia at platform day 5 (+/- 1 day). A patient with a negative blood culture at day 2 will be assigned no persistent bacteraemia at day 5 if that patient has no subsequent positive blood cultures from day 3 to day 5.<br><br>Population summary: As for estimand A2.1.	Treatment policy strategy (intent-to-treat principle)
**Estimand A2.7**<br><br>To evaluate, within the MRSA cell, the effect of revealed randomised intervention compared to the MRSA cell control, on the probability of a **change in backbone antibiotic therapy (vancomycin or daptomycin) due to an adverse event deemed by the treating doctor/team to be of sufficient severity to change therapy in the 14 days following platform entry**, in platform eligible participants.	Endpoint: Clinician-indicated change in backbone antibiotic therapy (vancomycin or daptomycin) due to adverse events or toxicity up to 14 days following platform entry.<br><br>Population summary: As for estimand A2.1.	Treatment policy strategy (intent-to-treat principle)

**Estimand A2.8**<br><br>To evaluate, within the MRSA cell, the effect of revealed randomised intervention compared to the MRSA cell control, on the probability of a **change in backbone antibiotic therapy (vancomycin or daptomycin) due to presumed lack of efficacy according to the treating doctor/team in the 14 days following platform entry**, in platform eligible participants.	Endpoint: Clinician-indicated change in backbone antibiotic therapy (vancomycin or daptomycin) due to lack of efficacy up to 14 days following platform entry.<br><br>Population summary: As for estimand A2.1.	Treatment policy strategy (intent-to-treat principle)
**Estimand A2.9**<br><br>To evaluate, within the MRSA cell, the effect of revealed randomised intervention compared to the MRSA cell control, on the probability of a **cessation of the allocated intervention due to an adverse event deemed by the treating doctor/team to be of sufficient severity to change therapy in the 7 days following platform entry**, in platform eligible participants.	Endpoint: Clinician-indicated cessation of the allocated intervention due to adverse event 7 days following platform entry.<br><br>Population summary: As for estimand A2.1.	Treatment policy strategy (intent-to-treat principle)

Platform entry is defined as the date that consent was obtained.

10.2. Statistical modelling

10.2.1. Primary model

The population summary (log-odds ratio) for the binary primary endpoint (all-cause mortality at 90 days) will be modelled using a Bayesian binomial model with a logit link. See the Statistical Analysis Appendix.

10.2.2. Secondary models

The population summaries for all core secondary endpoints as specified in the Core Protocol (Section 6.8.2) will be modelled as specified in the Statistical Analysis Appendix.

The domain-specific endpoints described in Section 10.1 are all binary and have log-odds population summaries that will be modelled using a Bayesian binomial model with a logit link. See the Statistical Analysis Appendix.

10.3. Decision criteria

Stopping decisions in this cell are based on whether the posterior probabilities that the intervention is **superior,** with respect to the cell control, is above, or below, a pre-specified threshold. Where a cell stopping decision is recommended because a posterior probability is below the pre-specified threshold, we say that it is futile to continue with the objective of demonstrating superiority.

The primary objective for this cell is to determine if vancomycin plus cefazolin is **superior** to vancomycin alone. Superiority is defined as an OR < 1 for the primary endpoint (where an OR > 1 indicates an increase in mortality for the vancomycin plus cefazolin compared to vancomycin alone). A cell stopping decision will be recommended for superiority if, at a pre-specified interim analysis, the posterior probability of superiority is greater than 99%. A cell stopping decision of futility of the superiority objective will be made if, at a pre-specified interim analysis, the posterior probability of OR <1/1.2 for the primary endpoint in this cell is less than 1%.

If, at any interim analysis, the thresholds for the decision criteria are not met within a cell, then recruitment into the cell will continue. In all other respects the decision criteria for this appendix are those outlined in the Core Protocol (Section 9.12) and the Statistical Appendix.

10.4. Randomisation

Participants will be randomised at platform entry in a fixed 1:1 ratio. A patient's allocated intervention will be revealed at the time that both MRSA is microbiologically identified, and domain and silo specific eligibility criteria are confirmed. Response adaptive randomization may be applied if additional interventions within this domain are included.

10.5. Interactions with domains

An *a priori* interaction with the adjunctive antibiotic domain is considered possible and will be incorporated into the statistical models used to analyse this domain. Beta-lactams can up-regulate toxin production and clindamycin may have a greater effect in cefazolin arm.

An *a priori* interaction with the early oral switch domain is not considered likely and will not be incorporated into the statistical models used to analyse this domain.

10.6. Pre-specified secondary analyses

Pre-specified secondary analyses on the primary estimand will be performed by modifying the primary statistical model to include the following covariates and their treatment interactions:

1) Endocarditis
    a. Left-sided endocarditis
        - The group of patients with left-sided endocarditis is often more difficult to treat, being more likely to require valve surgery and prolonged treatment.
    b. Right-sided endocarditis
        - This is a specific population group (right-sided IE often occurs in people who inject drugs).
    c. No endocarditis
2) Backbone antibiotic (vancomycin vs. daptomycin)
    a. Although synergy between both vancomycin and daptomycin with β-lactams has been demonstrated in laboratory experiments, there has not yet been clinical data to make a comparison of the comparative clinical effect. There is insufficient data to indicate the direction of a difference in clinical effect between vancomycin and daptomycin.
    b. If the backbone drug is changed by the treating team, the patient will be counted as receiving the backbone drug of which they received the most doses within the first 14 days
3) End stage kidney disease (ESKD); receiving haemodialysis at least once a week or continuous peritoneal dialysis) at baseline.
    a. These two groups are important to distinguish in light of patients with ESKD not being at risk for acute kidney injury or need for new renal replacement therapy. Any clinical efficacy impact of cefazolin may therefore be less likely offset by toxicity for patients with baseline ESKD compared to those without ESKD.
4) Vancomycin MIC ≤1 vs MIC >1.
    a. Laboratory data indicate that synergy between vancomycin or daptomycin and β-lactams is greater with increasing vancomycin or daptomycin MICs (the 'see-saw' effect)(16). Therefore, there may be a larger beneficial effect of combination therapy where the vancomycin MIC >1 compared to ≤1. The exact testing mechanism to determine the MIC is to be confirmed but will most likely use a reference testing method such as broth microdilution.
5) MRSA sequence type
    a. The underlying genetic background of the infecting MRSA may influence the MICs to vancomycin, β-lactams, potential synergy between vancomycin and β-lactams, and HCO_3 mediated resensitization of MRSA to β-lactams(17). Therefore, clinical responses to combination therapy may vary depending on the underlying genetic background.

b. In these exploratory analyses, isolates may be broadly categorised by multi-locus sequence typing, and finer distinctions may be made according to whole genome sequence analysis.

10.7 Principal stratum policy

The principal stratum policy (also known as a 'per protocol principle') for Estimand A2.1 uses the population as described in the following subsections.

10.6.1. If allocated to combination therapy

Received at least 80% of potential doses of cefazolin, rounded down to the nearest whole number. The number of potential doses is defined by the timing of allocation reveal and the renal function of the patient. For example, a patient with normal renal function with allocation reveal at lunch time on platform day 2 who survives to day 7 should receive 16 doses of cefazolin (1 on platform day 2, and 3 on each subsequent day up to and including day 7. If they received <12 doses, they will not be included under the principal stratum policy).

10.6.2. If allocated to standard therapy

Received ≤1 dose of any beta-lactam between time of allocation reveal and the end of day 14. If they received a dose of beta-lactam on the day of reveal, but not prior to it, they will still be included under the principal stratum policy.

11. ETHICAL CONSIDERATIONS

11.1. Data Safety and Monitoring Committee

The DSMC should be aware of the pre-specified decision criteria for superiority, inferiority, or futility of different interventions with respect to the primary endpoint. Determination of the optimal intervention may also involve secondary endpoints (Section 8.5.2).

11.2. Potential domain-specific adverse events

All treatment-related adverse events of particular interest for patients in this domain and silo are captured in the secondary endpoints (Section 8.5.2).

Other serious adverse reactions (SARs) should be reported only where, in the opinion of the site investigator, the event might reasonably have occurred as a consequence of a study intervention or study participation (see Core protocol Section 10).

11.3. Domain-specific consent issues

Consent for this domain will be sought at platform entry and will not require re-confirmation or repeat eligibility assessment at the time susceptibility is confirmed.

12. GOVERNANCE ISSUES

12.1. Funding of domain

Funding sources for the SNAP trial are specified in the Core Protocol Section 2.5. This domain has not received any additional domain-specific funding.

12.2. Funding of domain interventions and outcome measures

Interventions are considered standard of care and will be covered by hospital operating budgets. Outcome measures are pragmatic and do not deviate from routine testing performed during usual care for SAB. This domain and the interventions included have not received any additional domain-specific funding.

12.3. Domain-specific declarations of interest

All investigators involved in SNAP maintain a registry of interests on the SNAP website. These are updated periodically and publicly accessible on the study website.

13.REFERENCES

1. van Hal SJ, Jensen SO, Vaska VL, Espedido BA, Paterson DL, Gosbell IB. Predictors of mortality in Staphylococcus aureus Bacteremia. Clinical microbiology reviews. 2012;25(2):362-86.

2. Davis JS, Hal SV, Tong SY. Combination antibiotic treatment of serious methicillin-resistant *Staphylococcus aureus* infections. Semin Respir Crit Care Med. 2015;36(1):3-16.

3. Davis JS, Sud A, O'Sullivan MV, Robinson JO, Ferguson PE, Foo H, et al. Combination of Vancomycin and beta-Lactam Therapy for Methicillin-Resistant *Staphylococcus aureus* Bacteremia: A Pilot Multicenter Randomized Controlled Trial. Clin Infect Dis. 2016;62(2):173-80.

4. Abdul-Mutakabbir JC, Kebriaei R, Stamper KC, Sheikh Z, Maassen PT, Lev KL, et al. Dalbavancin, Vancomycin and Daptomycin Alone and in Combination with Cefazolin against Resistant Phenotypes of Staphylococcus aureus in a Pharmacokinetic/Pharmacodynamic Model. Antibiotics (Basel). 2020;9(10).

5. Tong SYC, Lye DC, Yahav D, Sud A, Robinson JO, Nelson J, et al. Effect of Vancomycin or Daptomycin With vs Without an Antistaphylococcal β-Lactam on Mortality, Bacteremia, Relapse, or Treatment Failure in Patients With MRSA Bacteremia: A Randomized Clinical Trial. JAMA. 2020;323(6):527-37.

6. Tong SYC, Davis JS, Eichenberger E, Holland TL, Fowler VG. *Staphylococcus aureus* Infections: Epidemiology, Pathophysiology, Clinical Manifestations, and Management. Clin Microbiol Rev. 2015;28(3):603-61.

7. Holmes NE, Tong SY, Davis JS, Hal SJ. Treatment of methicillin-resistant *Staphylococcus aureus*: vancomycin and beyond. Semin Respir Crit Care Med. 2015;36(1):17-30.

8. Hagihara M, Wiskirchen DE, Kuti JL, Nicolau DP. In vitro pharmacodynamics of vancomycin and cefazolin alone and in combination against methicillin-resistant Staphylococcus aureus. Antimicrob Agents Chemother. 2012;56(1):202-7.

9. Trinh TD, Zasowski EJ, Lagnf AM, Bhatia S, Dhar S, Mynatt R, et al. Combination Vancomycin/Cefazolin (VAN/CFZ) for Methicillin-Resistant *Staphylococcus aureus* (MRSA) Bloodstream Infections (BSI). Open Forum Infect Dis. 2017;4(Suppl 1):S281-S.

10. Geriak M, Haddad F, Rizvi K, Rose W, Kullar R, LaPlante K, et al. Clinical Data on Daptomycin plus Ceftaroline versus Standard of Care Monotherapy in the Treatment of Methicillin-Resistant Staphylococcus aureus Bacteremia. Antimicrob Agents Chemother. 2019;63(5).

11. Chaudhry SB, Veve MP, Wagner JL. Cephalosporins: A Focus on Side Chains and beta-Lactam Cross-Reactivity. Pharmacy (Basel). 2019;7(3).

12. Rybak MJ, Le J, Lodise TP, Levine DP, Bradley JS, Liu C, et al. Therapeutic monitoring of vancomycin for serious methicillin-resistant *Staphylococcus aureus* infections: A revised consensus guideline and review by the American Society of Health-System Pharmacists, the Infectious Diseases Society of America, the Pediatric Infectious Diseases Society, and the Society of Infectious Diseases Pharmacists. Am J Health Syst Pharm. 2020;77(11):835-64.

13. Figueroa DA, Mangini E, Amodio-Groton M, Vardianos B, Melchert A, Fana C, et al. Safety of high-dose intravenous daptomycin treatment: three-year cumulative experience in a clinical program. Clin Infect Dis. 2009;49(2):177-80.

14. Kullar R, Davis SL, Levine DP, Zhao JJ, Crank CW, Segreti J, et al. High-dose daptomycin for treatment of complicated gram-positive infections: a large, multicenter, retrospective study. Pharmacotherapy. 2011;31(6):527-36.

15. Rodvold KA, McConeghy KW. Methicillin-resistant Staphylococcus aureus therapy: past, present, and future. Clin Infect Dis. 2014;58 Suppl 1:S20-7.

16. Ortwine JK, Werth BJ, Sakoulas G, Rybak MJ. Reduced glycopeptide and lipopeptide susceptibility in Staphylococcus aureus and the "seesaw effect": Taking advantage of the back door left open? Drug resistance updates : reviews and commentaries in antimicrobial and anticancer chemotherapy. 2013;16(3-5):73-9.

17. Ersoy SC, Otmishi M, Milan VT, Li L, Pak Y, Mediavilla J, et al. Scope and Predictive Genetic/Phenotypic Signatures of Bicarbonate (NaHCO3) Responsiveness and beta-Lactam Sensitization in Methicillin-Resistant *Staphylococcus aureus*. Antimicrob Agents Chemother. 2020;64(5).
