# Supplementary material for: The Staphylococcus aureus Network Adaptive Platform Trial Protocol: New Tools for an Old Foe
Source: Clin Infect Dis. 2022 Jun 19;75(11):2027–34. doi: 10.1093/cid/ciac476 (PMC9710697; doi:10.1093/cid/ciac476)
Supplement: ciac476_Supplementary_Data [file ciac476_supplementary_data.zip › SNAP PICF- ADULT PROVIDING OWN CONSENT_v1.0_01JUNE2021.pdf]

Participant Information Sheet & Consent Form
ADULT PROVIDING OWN CONSENT

[Insert site name]

**Title**	*Staphylococcus aureus* **N**etwork **A**daptive **P**latform Trial (**SNAP**)
**Protocol Number**	CT19029
**Project Sponsor**	University of Melbourne
**Coordinating Principal Investigators**	A/Professor Steven Tong and Professor Josh Davis
**Site Principal Investigator**	*[Principal Investigator]*
**Associate Investigator(s)**	*[Associate Investigator(s)]*
**Location**	*[Location]*

You are invited to take part in a research study because you have a Staph aureus bloodstream infection. Please read this information to help you decide whether you want to take part.

For more information, click on the links in this document or if you are reading this on paper, scan this QR code with a smartphone camera:

SCAN ME

1. Why are we doing this study?

- Many kinds of antibiotics are used to treat *Staph aureus* bloodstream infections. These antibiotics are safe and work well.
- This study aims to find out which of the antibiotics currently used work best.
- 'Click here' to watch a video about *Staph aureus* bloodstream infections and the study.

2. Do I have to take part?

- If you do *not* want to take part that's OK. This will not affect the quality of care you receive.
- If you decide *not* to take part, you will be given the standard antibiotic treatments used in this hospital. This may be the same treatments you would have received in this study.
- If you take part, you are free to withdraw at any time without giving a reason.

3. If I take part, what will I need to do?

- You will be asked to sign a consent form.
- We will collect some information (like blood test results) from your medical records.
- You will be followed up for 90 days from the time you start treatment. We may collect information from you by telephone on Days 28, 42 and/or 90.
- You do not have to pay anything to be in the study, nor will you be paid. All medications, tests, and medical care for the study will be free of charge.
- 'Click here' to find out more about what being in the SNAP trial will mean for you

4. What will the study involve?

- There are different parts to this study. Each part is called a domain.
- This hospital is taking part in the following domain(s):

Delete this section if your site is not participating in the Antibiotic Backbone Domain

Antibiotic Backbone Domain

Three different types of bacterium (germ) cause *Staph aureus* bloodstream infections:

- Penicillin-susceptible *Staph aureus* **(PSSA)**
- Methicillin-susceptible *Staph aureus* **(MSSA)**
- Methicillin-resistant *Staph aureus* **(MRSA)**

Your infection is being caused by one of these germs

- You will be randomly allocated to your main (backbone) treatment depending on the type of germ that is causing your infection. This part of the study will help us find out which of these currently used antibiotics works best.
- Your doctors will give you more information about the antibiotics when they know what type of *Staph aureus* bloodstream infection you have.

**PSSA**	You will be randomly allocated to either Flucloxacillin or Penicillin
**MSSA**	You will be randomly allocated to either Flucloxacillin or Cefazolin
**MRSA**	You will be randomly allocated to either Vancomycin or Vancomycin + Cefazolin

- These antibiotics are given into a vein (IV treatment). Your doctor will decide how long this will last. This will range from 5 days to 6 weeks.
- Click here if you would like more information about this domain.

Delete this section if your site is not participating in the Adjunctive Treatment Domain

Adjunctive (Additional) Treatment Domain

- An antibiotic called Clindamycin stops *Staph aureus* germs from making toxins. This may limit the damage caused by *Staph aureus*.
- Adding Clindamycin to your main antibiotic treatment may help patients recover faster or improve outcomes but we do not know this for sure. This part of the study will help us find out.
- You will be randomly allocated to have clindamycin added to your main treatment, or not. If you are allocated to receive clindamycin, it will be given for 5 days.
- Click here if you would like more information about this domain.

Delete this section if your site is not participating in the Early Oral Switch Domain

Early Oral Switch Domain

- After 7 or 14 days of treatment (depending on how quickly you recover) you will be randomly allocated to either continue your antibiotics given into a vein (IV treatment) or switched to continue antibiotic treatment by mouth (oral treatment).
- We do not know whether it is better to switch early to oral treatment or to continue on IV treatment. This part of the study will help us find out.
- Click here if you would like more information about this domain.

5. What are the risks of taking part?

- All the treatments you may receive in this study are already widely used in normal care. The risks of being in the study are low.
- All antibiotics may have side effects. Most side effects are mild, such as diarrhoea, thrush and rash. Very rarely, more severe side effects might happen, such as liver or kidney inflammation, but these usually get better when the treatment is stopped.
- Your doctor will rule out any antibiotics you should *not* take based on your medical history.

6. What are the benefits of taking part?

- You may not benefit from this study, but it is possible the treatment you receive may work better, or more quickly, or be more convenient than treatment you would have otherwise received.
- Research like this also helps to continually improve the treatments and care provided to all patients now and in the future.
- This study is known as an *adaptive clinical trial*. In this type of study, the researchers analyse the results as the study goes on rather than just at the end. If the results show any of the treatments do not work as well as others, they will be dropped from the study. This means that after the study has been running for a while, there may be better odds of getting a better treatment.
- Click here if you want to find out more about adaptive clinical trials.

7. What will happen to the information collected for this study?

- The study team will use your details to contact you for the follow-up checks, and to send you information about the study results.
- As part of the study, we will also collect data for a registry of patients with *Staph aureus* bloodstream infection. This data will include the severity, management, and the outcome of your infection. The registry will be used to improve current practice and quality of care.
- Data collected for the study and for the registry will be stored in a secure database in accordance with the relevant Australian and *(insert name of state/territory)* privacy laws.
- Your name, address, and date of birth may be used to link your study information to existing datasets, such as hospital records, medications, emergency department, and registries of births, deaths, and marriages. This will allow longer follow-up of outcomes, like readmission to hospital, without us needing to contact you directly.
- Your information will be kept strictly confidential and only used for the study or for future research related to *Staph aureus* infections and related conditions. The only people allowed to look at information that could identify you (name, address, date of birth) will be your doctors, study staff, the data co-ordinator from the University of Melbourne, the unit who performs the linkage, and regulatory authorities who may want to check the study is carried out correctly.
- Click here if you would like more information about Data Collection and click here if you want to find out more about Data Linkage.

8. What will happen to the samples collected for this study?

- The Staph bacteria grown from your blood culture will be stored in a sample bank.
- Studies on the cultured Staph bacteria will help researchers better understand how the bacteria make people sick and how the bacteria respond to antibiotic treatment.

- No other samples (e.g., blood or urine) will be collected or stored for this study unless you are participating in a sub-study, which will be detailed in a separate information and consent form.

9. What if I withdraw from this research project?

- You can withdraw from the study at any time, just notify a member of the research team. They will discuss any health risks or special requirements linked to withdrawing.

If you do withdraw, you will have two options:

  - Withdraw from the study treatment ONLY - if you choose this option, you will receive standard treatment, and we will continue to collect data from you for the 90 days.
  - Withdraw from the study treatment *and* further data collection. We will keep any information we have collected about you up until you withdraw. If you do not agree with this, you should not join the study.

10. Who is organising this study?

- The study is being co-ordinated by a team based at the Doherty Institute at the University of Melbourne.
- The study is being funded by a research grant from Australia's National Health and Medical Research Council until the end of 2025.

11. Where can I find more information?

Click here for more information, including:

- More information on each of the antibiotics in the trial
- The contact information for the ethics committee that approved this study
- Information on complaints and compensation
- What happens if the trial is stopped unexpectedly and how you will be kept informed if new information arises during the study.

How to contact us
Here is the contact for your study doctor:<br><br>Dr ________________________<br>Telephone: ________________

Thank you for taking time to read this information sheet.
If at any time anything is unclear or you have any questions, please make sure to ask your doctor who would be happy to help and answer your questions.

Insert Header with institution's name or institution's letterhead

Consent Form

ADULT PROVIDING OWN CONSENT

[Insert site name]

**Title**	*Staphylococcus aureus* **N**etwork **A**daptive **P**latform Trial (**SNAP**)
**Protocol Number**	CT19029
**Project Sponsor**	University of Melbourne
**Coordinating Principal Investigators**	A/Professor Steven Tong and Professor Josh Davis
**Site Principal Investigator**	*[Principal Investigator]*
**Associate Investigator(s)**	*[Associate Investigator(s)]*
**Location**	*[Location]*

Consent Agreement

- I have read the Participant Information Sheet or someone has read it to me in a language that I understand.
- I understand the purposes, procedures and risks of the research described in the project.
- I give permission for my doctors, other health professionals, hospitals or laboratories outside this hospital to release information to *[Name of Institution]* concerning my condition and treatment for the purposes of this project. I understand that such information will remain confidential.
- I understand that the bacteria grown from my blood culture will be stored in a sample bank, and will be used for future studies related to *Staph aureus* bloodstream infections.
- I understand that my data will be added to the SNAP registry.
- I have had an opportunity to ask questions and I am satisfied with the answers I have received.
- I freely agree to participate in this research project as described and understand that I am free to withdraw at any time during the study without affecting my future health care.
- I understand that I will be given a signed copy of this document to keep.
- I understand that, if I decide to discontinue the study treatment, I may be contacted by telephone on Days 28, 42 and 90, and a member of the research team may access my medical records for collection of follow-up information.
- I agree to participate in:
  - All study domains that are available at this site ☐ Yes ☐ No

    OR
  - The antibiotic backbone domain ☐ Yes ☐ No
    [delete if hospital site not participating in antibiotic backbone domain]
  - The adjunctive treatment (clindamycin) domain ☐ Yes ☐ No
    [delete if hospital site not participating in adjunctive treatment domain]
  - The early oral switch domain ☐ Yes ☐ No
    [delete if hospital site not participating in early oral switch domain]

- I agree for my personal information to be linked with state/territory hospital, emergency department and births, deaths and marriages registries, and for the linked health information to be used for the purposes of this study ☐ Yes ☐ No

Declaration by Participant – ***For participants who have read the information***

Name of Participant (please print) ______________________________

Signature ______________________ Date ______________________

Declaration ***-Only if participant is not able to read the text and/or sign for themselves but has capacity to give consent***

Witness to the informed consent process:

Name (please print) ______________________________

Signature ______________________ Date ______________________

*Witness is not to be the Investigator, a member of the study team or their delegate. Witness must be 18 years or older.

Name of Interpreter if used (please print) ______________________________

Signature ______________________ Date ______________________

☐ Consent was obtained using interpretation by telephone

Declaration by Study Doctor/Senior Researcher†

I have given a verbal explanation of the research project, its procedures and risks and I believe that the participant has understood that explanation.

Name of Study Doctor/
Senior Researcher† (please print) ______________________________

Signature ______________________ Date ______________________

† A senior member of the research team must provide the explanation of, and information concerning, the research project.
