# Supplementary material for: The Staphylococcus aureus Network Adaptive Platform Trial Protocol: New Tools for an Old Foe
Source: Clin Infect Dis. 2022 Jun 19;75(11):2027–34. doi: 10.1093/cid/ciac476 (PMC9710697; doi:10.1093/cid/ciac476)
Supplement: ciac476_Supplementary_Data [file ciac476_supplementary_data.zip › SNAP PSSA_MSSA Backbone Domain-Specific Appendix.pdf]

Domain-Specific Appendix:

Backbone Domain: Penicillin and Methicillin-susceptible Silos

Staphylococcus aureus **Network Adaptive Platform trial (SNAP)**

PSSA/MSSA Treatment Domain-Specific Appendix Version 1.0 dated 01 October 2021

Summary

In this domain of the SNAP trial, participants with *Staphylococcus aureus* bacteraemia (SAB) admitted to participating hospitals will be randomised to receive one of up to 2 interventions depending on site availability and patient and clinical team acceptability:

1. Intravenous Penicillin G (Benzylpenicillin) vs. Intravenous (Flu)Cloxacillin for penicillin-susceptible *S. aureus* (PSSA)
2. Intravenous Cefazolin vs. Intravenous (Flu)Cloxacillin for methicillin-susceptible *S. aureus* (MSSA)

At this participating site the following interventions have been selected within this domain:

☐ Intravenous Penicillin G (Benzylpenicillin) vs. Intravenous (Flu)Cloxacillin for PSSA

☐ Intravenous Cefazolin vs. Intravenous (Flu)Cloxacillin for MSSA

<table>
<tr><th colspan="2">SNAP: MSSA/PSSA Backbone Domain Summary</th></tr>
<tr><td>Interventions</td><td>PSSA:
Intravenous (Flu)cloxacillin
Intravenous Benzylpenicillin/Penicillin G
MSSA:
Intravenous (Flu)cloxacillin
Intravenous Cefazolin
See text for dosing, including adjustments for renal function and severity/type of illness.

Duration of allocated antibiotic – Ideally, allocated antibiotics will be continued for the entire parenteral treatment duration. Participants should receive at least 14 days of allocated study drug (unless participating in the early oral switch domain where the minimum will be 5 days (uncomplicated) or 12 days (complicated).</td></tr>
<tr><td>Silos, domains, and cells</td><td>Platform eligible participants within the PSSA and MSSA silos in the backbone antibiotic domain will be analysed. The population parameters in each ‘cell’ (a combination of a silo and a domain) will be estimated and reported separately.</td></tr>
<tr><td>Evaluable treatment-by-treatment Interactions</td><td>Treatment-treatment interactions will not be evaluated between interventions in this domain and interventions in any other domain.</td></tr>
<tr><td>Randomisation</td><td>Participants will be randomised at platform entry in a fixed 1:1 ratio within each of the PSSA and MSSA cells. A patient’s allocated intervention will be revealed at the time that susceptibility results to define MSSA or PSSA are available and domain-specific eligibility criteria are confirmed. Response adaptive randomization may be applied if additional interventions within these cells are included in future versions of this DSA.</td></tr>
<tr><td>Domain Specific Inclusions</td><td>Inclusion criteria are the same as the Platform (see Core Protocol Section 6.5), and
For MSSA silo: Index blood culture isolate is methicillin-susceptible but penicillin resistant as per the Microbiology Appendix.
For PSSA silo: Index blood culture isolate is penicillin-susceptible as per the Microbiology Appendix. In short, this will require phenotypic disc testing with EUCAST (a P1 disc diffusion with a feathered zone >=26mm) OR CLSI (a P10 disc diffusion) defined criteria.</td></tr>
</table>

	Note that where trial sites are not testing for penicillin-susceptibility using a P disc, patients will be excluded from this backbone domain *only if* an automated testing method reports PSSA.
Domain Specific Exclusions	Patients will be excluded from this domain if they meet any of the following criteria at the time of domain eligibility assessment:<br>1. >72 hours have elapsed from the time of index blood culture draw<br>2. History of type I hypersensitivity reaction (i.e. anaphylaxis or angioedema) to any penicillin or cephalosporin<br>3. History of severe delayed reaction (e.g. allergic interstitial nephritis, cutaneous vasculitis, Stevens-Johnson, DRESS, etc.) to any penicillin or cephalosporin<br>4. PSSA silo: non-severe rash to any penicillin (unless patient has been subsequently de-labelled; this criteria does not include criteria 2 and 3 above), or<br>MSSA silo: non-severe rash to cefazolin or any penicillin (unless patient has been subsequently de-labelled)<br>5. Treating team deems enrolment in this domain is not in the best interest of the patient<br>6. Currently receiving maintenance dialysis (haemodialysis or peritoneal dialysis)
Endpoints	Primary platform endpoint: All-cause mortality at 90 days from platform entry.<br>Secondary platform endpoints: refer to Core Protocol Section 6.8.<br>Secondary domain-specific endpoints:<br>1. Acute kidney injury (Modified KDIGO stage 1 defined as an increase in serum creatinine of $\geq$26.5 μmol/L from platform entry (baseline) to day 5 OR Increase in serum creatinine by 1.5 times or more the level at platform entry (baseline) within 14 days of platform entry<br>2. Receipt of renal replacement therapy at any stage up to platform day 90<br>3. Ongoing renal replacement therapy at platform day 90<br>4. Hepatotoxicity - Grade 2 or above increase in ALT and/or GGT (>2.5x ULN), which was not present at platform entry, within 14 days after platform entry<br>5. Change in assigned backbone antibiotic during the total index hospitalisation, starting from reveal of allocated domain intervention, due to an adverse event deemed by the treating doctor/team to be of sufficient severity to change therapy<br>6. Change in assigned backbone antibiotic therapy during the total index hospitalisation, starting from reveal of allocated domain intervention, due to presumed lack of efficacy according to the treating doctor/team

	7. Peripherally inserted central catheter (PICC)/other central venous catheter complications requiring line removal, during the total index hospitalisation, starting from reveal of allocated domain intervention.
Decision criteria	The primary objective for this domain is to determine if penicillin is **non-inferior** to (flu)cloxacillin for the PSSA silo and if cefazolin is **non-inferior** to (flu)cloxacillin for the MSSA silo. Non-inferiority is defined as OR < 1.2 for the primary endpoint (where OR > 1 indicates an increase in mortality for penicillin in the PSSA silo or for cefazolin in the MSSA silo). Within each cell, if non-inferiority is demonstrated at a pre-specified interim analysis, which is defined as a posterior probability of non-inferiority in that cell greater than 99%, recruitment into the cell may continue to seek a conclusion of superiority, based on a recommendation from the DSMC and TSC. A cell stopping decision will be made for futility of the non-inferiority objective if, at a pre-specified interim analysis, the posterior probability of non-inferiority in that cell is less than 1%.<br><br>Superiority is defined as an OR < 1 for the primary endpoint. Within each cell, a stopping decision for superiority will be made if, at a pre-specified interim analysis, the posterior probability of superiority in that cell is greater than 99%. A cell stopping decision for futility of the superiority objective will be made if, at a pre-specified interim analysis, the posterior probability of OR < 1/1.2 for the primary endpoint in that cell is less than 1%.<br><br>If, at any interim analysis, the thresholds for the decision criteria are not met within a cell, then recruitment into the cell will continue.
Pre-specified secondary analyses	Pre-specified secondary analyses on the primary estimand will be performed by modifying the primary statistical model to include the following covariates and their treatment interactions:<br>1. Endocarditis<br>a. Left-sided endocarditis<br>b. Right-sided endocarditis<br>c. No endocarditis<br>2. For PSSA silo - presence of *blaZ* and absence of *blaZ*<br>3. For MSSA silo - presence of cefazolin inoculum effect (CIE) and absence of CIE<br>4. For MSSA silo - presence of flu(cloxacillin) inoculum effect (FIE) and absence of FIE<br>5. For MSSA silo - presence of type A beta-lactamase versus its absence<br>6. For MSSA silo – focus of infection includes central nervous system versus not

TABLE OF CONTENTS

1. ABBREVIATIONS ____ 9
2. PROTOCOL APPENDIX STRUCTURE ____ 11
3. PSSA/MSSA TREATMENT DOMAIN-SPECIFIC APPENDIX VERSION ____ 12
   3.1. Version history ____ 12
4. PSSA/MSSA TREATMENT DOMAIN GOVERNANCE ____ 12
   4.1. Domain members ____ 12
   4.2. Contact Details ____ 12
5. PSSA/MSSA TREATMENT DOMAIN-SPECIFIC WORKING GROUP AUTHORIZATION ____ 13
6. BACKGROUND AND RATIONALE ____ 14
   6.1. Domain definition ____ 14
   6.2. Domain-specific background ____ 14
      6.2.1. Benzylpenicillin (Penicillin G) vs. Flu(Cloxacillin) for the treatment PSSA bacteremia ____ 14
      6.2.2. Cefazolin vs. Flu(Cloxacillin) for the treatment of MSSA bacteremia ____ 14
7. DOMAIN OBJECTIVES ____ 15
8. TRIAL DESIGN ____ 16
   8.1. Population ____ 16
   8.2. Eligibility criteria ____ 16
      8.2.1. Cell inclusion criteria ____ 16
      8.2.2. Cell exclusion criteria – at the time of domain eligibility assessment ____ 17
      8.2.3. Intervention exclusion criteria ____ 17
   8.3. Interventions ____ 17
      8.3.1. PSSA Treatment Interventions ____ 17
      8.3.2. MSSA Treatment Interventions ____ 18
      8.3.3. Dose adjustments for both silos ____ 19
      8.3.4. Timing of initiation of PSSA/MSSA Treatment ____ 20
      8.3.5. Duration of administration of PSSA/MSSA Treatment ____ 20
   8.4. Concomitant care ____ 21

8.5. Endpoints 21
8.5.1. Primary endpoint 21
8.5.2. Secondary outcomes 21

9. TRIAL CONDUCT 22

9.1. Domain-specific data collection 22
9.1.1. Clinical data and sample collection 22
9.1.2. Domain-specific study timeline 23
9.1.3. Domain-specific study visit day details 23

9.2. Criteria for discontinuation 25

9.3. Blinding 25
9.3.1. Blinding 25
9.3.2. Unblinding 25

10. STATISTICAL CONSIDERATIONS 25

10.1. Estimands, endpoints, and intercurrent events 25
10.1.1. Primary estimand 25
10.1.2. Secondary estimands 25

10.2. Statistical modelling 28
10.2.1. Primary model 28
10.2.2. Secondary models 28

10.3. Decision criteria 28

10.4. Randomization 29

10.5. Interactions with interventions in other domains 29

10.6. Pre-specified secondary analyses 29

10.7. Principal stratum policy 31
10.7.1. PSSA silo 31
10.7.2. MSSA silo 31

11. ETHICAL CONSIDERATIONS 32

11.1. Data Safety and Monitoring Committee 32

11.2. Potential domain-specific adverse events 32

11.3. Domain-specific consent issues 32

12. GOVERNANCE ISSUES 33

12.1. Funding of domain 33

12.2. Funding of domain interventions and outcome measures 33

12.3. Domain-specific declarations of interest 33

13. REFERENCES 34

1. ABBREVIATIONS

AKI	Acute Kidney Injury
ALT	Alanine Aminotransferase
ASP	Anti-Staphylococcal Penicillin
CIE	Cefazolin Inoculum Effect
CNS	Central Nervous System
CRRT	Continuous Renal Replacement
DRESS	Drug Reaction with Eosinophilia and Systemic Symptoms
DSA	Domain-Specific Appendix
DSWG	Domain-Specific Working Group
DSMC	Data and Safety Monitoring Committee
eGFR	Estimated Glomerular Filtration Rate
ESC	European Society of Cardiology
FIE	(Flu)cloxacillin Inoculum Effect
GGT	Gamma-Glutamyl Transferase
GTSC	Global Trial Steering Committee
HITH	Hospital in the home
IDSA	Infectious Diseases Society of America
IE	Infective Endocarditis
ICU	Intensive Care Unit
IHD	Intermittent Haemodialysis
KDIGO	Kidney Disease Improving Global Outcomes
MSSA	Methicillin-susceptible *Staphylococcus aureus*
MIC	Minimum Inhibitory Concentration
OPAT	Outpatient Antimicrobial Therapy
PCR	Polymerase Chain Reaction
PICC	Peripherally inserted central catheter
PSSA	Penicillin-susceptible *Staphylococcus aureus*
RAR	Response Adaptive Randomization

RCT	Randomized Controlled Trial
RSA	Region-Specific Appendix
SAE	Serious Adverse Event
SNAP	*Staphylococcus aureus* Network Adaptive Platform trial

2. PROTOCOL APPENDIX STRUCTURE

The structure of this protocol is different to that used for conventional trials because this trial is highly adaptive and the description of these adaptations is better understood and specified using a 'modular' protocol design. While all adaptations are pre-specified, the structure of the protocol is designed to allow the trial to evolve over time, for example by the introduction of new domains or interventions or both, and commencement of the trial in new geographical regions.

The protocol has multiple modules, in brief, comprising a Core Protocol (overview and design features of the study), a Statistical Analysis Appendix (details of the current statistical analysis plan and models, including simulations to support trial design), multiple Domain-Specific Appendices (DSA) (detailing all interventions currently being studied in each domain), and multiple Region-Specific Appendices (RSA) (detailing regional management and governance).

The Core Protocol contains all information that is generic to the trial, irrespective of the regional location in which the trial is conducted and the domains or interventions that are being tested. The Core Protocol may be amended but it is anticipated that such amendments will be infrequent.

The Core Protocol does not contain information about the intervention(s) within each domain, because one of the trial adaptations is that domains and interventions will change over time. Information about interventions, within each domain, is covered in a DSA. These Appendices are anticipated to change over time, with removal and addition of options within an existing domain, at one level, and removal and addition of entire domains, at another level. Each modification to a DSA will be subject to a separate ethics application for approval.

The Core Protocol does not contain detailed information about the statistical analysis or simulations, because the analysis model will change over time in accordance with the domain and intervention trial adaptations but this information is contained in the Statistical Analysis Appendix. These Appendices are anticipated to change over time, as trial adaptations occur. Each modification will be subject to approval from the Global Trial Steering Committee (GTSC) in conjunction with advice from the Statistical Subcommittee and the Data and Safety Monitoring Committee (DSMC).

The Core Protocol also does not contain information that is specific to a particular region in which the trial is conducted, as the locations that participate in the trial are also anticipated to increase over time. Information that is specific to each region that conducts the trial is contained within a RSA. This includes information related to local management, governance, and ethical and regulatory aspects. It

is planned that, within each region, only that region's RSA, and any subsequent modifications, will be submitted for ethical review in that region.

The current version of the Core Protocol, DSAs, RSAs, and the Statistical Analysis Appendix is listed in the Protocol Summary and on the study website (https://www.snaptrial.com.au/).

3. PSSA/MSSA TREATMENT DOMAIN-SPECIFIC APPENDIX VERSION

The version of the PSSA/MSSA Treatment Domain-Specific Appendix is in this document's header and on the cover page.

3.1. Version history

Version 1.0: Approved by the PSSA/MSSA Treatment Domain-Specific Working Group (DSWG) on the 29th March 2021.

4. PSSA/MSSA TREATMENT DOMAIN GOVERNANCE

4.1. Domain members

Chairs: Todd C. Lee MD MPH FIDSA (McGill University, Canada)

Members:
Joshua Davis
Steven Tong
Andrew Henderson
Genevieve McKew
Nesrin Ghanem-Zoubi
Kate Grimwade
Sophia Archuleta
Brendan McMullan

4.2. Contact Details

Chair:

Dr. Todd C. Lee

McGill University Health Centre

1001 Decarie Blvd. E5-1820

Montreal, QC H4A3S1

Canada

Phone 514-934-1934

Email todd.lee@mcgill.ca

Project Manager:

Jocelyn Mora SNAP Clinical Trial Manager

The Peter Doherty Institute for Infection and Immunity

792 Elizabeth St Melbourne VIC AUSTRALIA

+61 (0) 38344 2554

jocelyn.mora@unimelb.edu.au

5. PSSA/MSSA TREATMENT DOMAIN-SPECIFIC WORKING GROUP AUTHORIZATION

The PSSA/MSSA Treatment Domain-Specific Working Group (DSWG) have read the appendix and authorize it as the official PSSA/MSSA Treatment Domain-Specific Appendix for the SNAP trial. Signed on behalf of the committee,

Chair 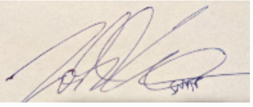 Date 29 March 2021

Todd Lee

6. BACKGROUND AND RATIONALE

6.1. Domain definition

This is a domain within the SNAP trial to test the effectiveness of benzylpenicillin (PSSA) or cefazolin (MSSA) vs. (flu)cloxacillin in patients with PSSA or MSSA bacteraemia who are admitted to a participating hospital.

6.2. Domain-specific background

6.2.1. Benzylpenicillin (Penicillin G) vs. Flu(Cloxacillin) for the treatment PSSA bacteremia

The re-emergence of penicillin-susceptible *S. aureus* bacteraemia has been documented worldwide. These isolates now comprise up to 25% of cases (1-5). Nonetheless, the optimal treatment of penicillin-sensitive disease remains unknown. For penicillin-susceptible *S. aureus* endocarditis, the Infectious Diseases Society of America (IDSA) guidelines recommend cloxacillin in favour of penicillin citing the concern for misclassifying low-level beta-lactamase producers as sensitive (6). However, the clinical implications of this in vitro misclassification are unknown, and modern disk diffusion testing minimizes any misclassification (3, 4, 7, 8). Clinical outcomes may be better with penicillin as it has a lower minimum inhibitory concentration (MIC) distribution, prolonged antibiotic concentration levels above the MIC, and higher levels of non-protein-bound drug in plasma (2). It may also have a better adverse event profile than cloxacillin including less phlebitis, hepatotoxicity, and renal toxicity (9). A systematic review identifies no RCTs addressing this question (10). In the largest retrospective study addressing this question, Australian investigators found an increased 30-day mortality (OR 1.06, 95% CI 1.01 to 1.1; p = 0.03) associated with flucloxacillin when compared to penicillin (11). Given rising rates of penicillin susceptibility in *S. aureus* bacteraemia worldwide (1, 3, 4, 12), there is strong interest in this question in the clinical community (13). This trial provides the opportunity to inform international treatment guidelines by evaluating penicillin which is a potentially safer, non-inferior, or even superior antibiotic compared with the more commonly used isoxalolyl penicillins.

6.2.2. Cefazolin vs. Flu(Cloxacillin) for the treatment of MSSA bacteremia

Methicillin-susceptible isolates are the most common type of *S. aureus* bacteraemia accounting for 60-80% of all episodes (2, 5, 14-16). In methicillin-susceptible *S. aureus* bacteraemia, there is a persistent international debate regarding the use of cefazolin versus anti-staphylococcal penicillins (ASPs), such as flucloxacillin, cloxacillin, oxacillin or nafcillin. The endocarditis guidelines from both the IDSA and European Society of Cardiology (ESC) favour ASPs (6, 17) citing concerns regarding cefazolin

stability in the presence of high levels of penicillinase, an in vitro phenomenon termed the inoculum effect (18, 19) that may be seen in high burden infections (6, 17).

MSSA strains produce a variety of beta-lactamases, to which ASPs are stable. Some MSSA strains demonstrate an in-vitro "inoculum effect" to cefazolin, whereby a high inoculum (107cfu/ml) is associated with a several-fold increase in MIC compared to a standard inoculum (105 cfu/ml) (20). This is usually due to the production of a type A beta-lactamase which is active in-vitro against cefazolin. This led to concerns about treatment failure with cefazolin in selected MSSA strains, and early case reports of the same (21, 22). However, cefazolin has some potential advantages over ASPs, including a longer half-life and a lower tendency to cause phlebitis and renal and liver toxicity.

In recent years, several small observational studies have been published that suggest that outcomes for patients with invasive MSSA infections treated with cefazolin are equivalent to or possibly even superior to those treated with ASPs (23-28), and that the safety profile is superior and cost of cefazolin lower (29, 30). A more recent larger retrospective observational study including 7,312 episodes of MSSA bacteraemia found a non-significantly lower mortality in those treated with cefazolin compared to those treated with flucloxacillin (adjusted OR 0.86, 95% CI 0.65 to 1.14). A subsequent systematic review and meta-analysis including data from 11,760 patients in 14 observational studies found a decreased chance of 30-day mortality in the cefazolin group [relative risk 0.70, 95% CI 0.54 to 0.91] (10).

Cefazolin also has logistical advantages with less frequent dosing allowing for less use of hospital resources and easier home intravenous therapy. We do not believe that the observational evidence definitively answers the question of whether cefazolin should be preferred. Even the best observational data may be confounded by severity or indication (31). There are too many examples where observational data has informed practice only to be the subject of a medical reversal when an RCT is performed (32-35). Consequently, international groups have called for a high quality RCT (10, 26). If the results of the observational studies are a true effect, we could save many lives and improve other patient outcomes by determining and then standardizing care with a potentially superior antibiotic like cefazolin.

7. DOMAIN OBJECTIVES

The objective of this domain is to determine the effectiveness of benzylpenicillin or cefazolin when compared to (flu)cloxacillin for patients with appropriately susceptible *Staphylococcus aureus* bacteraemia.

We hypothesize that the probability of all-cause mortality at 90 days after enrolment will be **non-inferior** based on the receipt of benzylpenicillin (in the PSSA silo) or cefazolin (in the MSSA silo) in place of (flu)cloxacillin. (Flu)cloxacillin is considered the current standard of care for both silos. Should non-inferiority be demonstrated for a cell (PSSA or MSSA silo-domain combination), the trial is designed to continue recruitment and seek superiority, if recommended by the GTSC and DSMC (see Statistical Analysis Appendix).

8. TRIAL DESIGN

This domain will be conducted as part of the SNAP trial (see Core Protocol Section 6). Treatment allocation will be at a fixed 1:1 ratio, as described in the Core Protocol Section 6.7.

8.1. Population

Patients with *S. aureus* bacteraemia admitted to a participating hospital.

8.2. Eligibility criteria

Patients are eligible for this domain if they meet all of the platform-level inclusion and none of the platform-level exclusion criteria (see Core Protocol Section 6.5) AND all of the cell-level inclusion and none of the cell-level exclusion criteria.

8.2.1. Cell inclusion criteria

- For MSSA silo: Index blood culture isolate is methicillin-susceptible as per the Microbiology Appendix.
- For PSSA silo: Index blood culture isolate is penicillin-susceptible as per the Microbiology Appendix. In short, this will require phenotypic disc testing with EUCAST (a P1 disc diffusion with zone >=26mm OR a P1 disc diffusion with zone >=26mm and the zone edge is NOT sharp) **OR** CLSI (a P10 disc diffusion) defined criteria.

Note that where trial sites are not testing for penicillin-susceptibility, patients with MSSA/PRSA can be included in the MSSA silo, but those with MSSA/PSSA (but not confirmed with a P-disc) will be excluded from the backbone domain. The rationale for this is that patients with MSSA but not tested with a P-disc may be truly PSSA (with no *blaZ*). If the cefazolin inoculum effect (CIE) is a clinically relevant entity, then including patients with an organism without *blaZ* (and hence cannot have a CIE phenotype), will bias towards non-inferiority of cefazolin compared to (flu)cloxacillin.

For PSSA, the requirement for laboratories to use an accredited phenotypic test for a penicillin-susceptible phenotype, is to ensure clinical safety according to internationally accepted guidelines. The automated antimicrobial susceptibility testing, and other phenotypic tests, have poor sensitivity for detection of *blaZ* compared to a gold standard of *blaZ* PCR. Therefore, patients could be placed at risk of treatment with benzylpenicillin when the infecting isolate is actually *blaZ* positive, unless these guidelines are followed.

8.2.2. Cell exclusion criteria – at the time of domain eligibility assessment

1. >72 hours have elapsed since the collection of the index blood culture (i.e. the time of collection of the first positive blood culture from the patient during this episode)
2. History of type I hypersensitivity reaction (i.e. anaphylaxis or angioedema) to any penicillin or cephalosporin
3. History of severe delayed reaction (e.g. allergic interstitial nephritis, cutaneous vasculitis, Stevens-Johnson, DRESS, etc.) to any penicillin or cephalosporin
4. PSSA silo: Non-severe rash to any penicillin (unless patient has been subsequently de-labelled; this criteria does not include criteria 2 and 3 above), *or*
   MSSA silo: Non-severe rash to cefazolin or any penicillin (unless patient has been subsequently de-labelled)
   - Nausea, diarrhoea, headache, and other non-specific symptoms are NOT allergies, they are drug intolerance, and they are not exclusion criteria. Similarly, a vague history of an allergy of unclear nature, or a family history of allergy are not exclusions.
5. Treating team deems enrolment in this domain is not in the best interest of the patient
6. Currently receiving maintenance dialysis (haemodialysis or peritoneal dialysis)
   - Acute renal replacement therapy (including CRRT, haemodialysis or peritoneal dialysis) are not exclusions. Such patients are eligible as long as appropriate vascular access is available or can be arranged.

8.2.3. Intervention exclusion criteria

There are no intervention specific exclusions.

8.3. Interventions

8.3.1. PSSA Treatment Interventions

Standard recommended adult dosing

Those randomized to (flu)cloxacillin, and flucloxacillin is available:

- (Flu)cloxacillin 2g every 6 hours intravenously

Those randomized to (flu)cloxacillin, and cloxacillin but not flucloxacillin is available:

- Cloxacillin 2g every 4 hours intravenously

Those randomized to penicillin can use one of two dosing regimens, at the treating clinicians' discretion:

- Benzylpenicillin (=Penicillin G) 1.8g (=3 million units) every 4 hours intravenously (preferred option)

  OR
- Benzylpenicillin (=Penicillin G) 2.4g (=4 million units) every 6 hours intravenously

For patients with critical illness (defined as being admitted to ICU or having septic shock), endocarditis or central nervous system infection (includes brain or spinal cord infection, subdural empyema or CNS device-related infection, but does not include epidural abscess)

- (Flu)cloxacillin 2g every 4 hours intravenously

OR

- Benzylpenicillin (=Penicillin G) 2.4g (=4 million units) every 4 hours intravenously

8.3.2. MSSA Treatment Interventions

Standard recommended adult dosing

Those randomized to (flu)cloxacillin, and flucloxacillin is available:

- (Flu)cloxacillin 2g every 6 hours intravenously

Those randomized to (flu)cloxacillin, and cloxacillin but not flucloxacillin is available:

- Cloxacillin 2g every 4 hours intravenously

Those randomised to cefazolin

- Cefazolin 2g every 8 hours intravenously

For patients with critical illness (defined as being admitted to ICU or having septic shock), endocarditis or central nervous system infection (includes brain or spinal cord infection, subdural empyema or CNS device-related infection, but does not include epidural abscess)

- (Flu)cloxacillin 2g every 4 hours intravenously

OR

- Cefazolin 2g every 6 hours intravenously

8.3.3. Dose adjustments for both silos

When critically ill patients have recovered (meaning no longer requiring mechanical ventilation OR vasopressors/inotropes for at least 24 hours) the dose may be decreased to standard dose. Otherwise, dose adjustments based on eGFR as follows:

Benzylpenicillin (Penicillin G):

GFR (mL/minute)	Standard Dose	High Dose (critical illness/IE)
>50	1.8g (3 million units) q4h IV OR 2.4g (4 million units) q6h IV	2.4g (4 million units) q4h IV
10-50	1.8g (3 million units) q6h IV	1.8g (3 million units) q4h IV
<10	Loading dose of 1.8g (3MU) IV then 1.2g (2MU) q8h IV	Loading dose of 2.4g (4MU) IV then 1.2g (2MU) q6h IV
CRRT*	1.2g (2 million units) q6h	1.8g (3 million units) q6h

**Continuous renal replacement therapy*

Flucloxacillin:

GFR (mL/minute)	Standard Dose	High Dose (critical illness/IE)
>50	2g q6h IV	2g q4h IV
10-50	2g q6h IV	2g q4h IV
<10	1g q6h IV	1g q6h IV
CRRT	2g q6h IV	2g q8h IV

Cloxacillin:

There is no renal dosage adjustment necessary for any level of GFR or for CRRT.

Cefazolin

GFR (mL/minute)	Standard Dose	High Dose (critical illness/IE)
>40	2g q8h IV	2g q6h IV
20-40	2g q12h IV	2g q12h IV
<20	1g q24h IV	1g q24h IV
CRRT	2g q12h IV	2g q12h IV

8.3.4. Timing of initiation of PSSA/MSSA Treatment

Treatment will be started as soon as possible after the local study team become aware of the organism's susceptibility, AND domain eligibility is confirmed. For MSSA, this means as soon as PSSA is excluded. For PSSA, this means as soon as PSSA is confirmed.

If the patient is already receiving the backbone antibiotic to which they have been randomly allocated (e.g. they have been randomised to IV flucloxacillin, and they are already receiving it), the next dose will be determined by the dosing interval recommended in sections 8.3.1 to 8.3.3. above. If they are not already receiving the allocated treatment, the first dose should be given as soon as possible, regardless of the timing of previous doses of other antibiotics.

8.3.5. Duration of administration of PSSA/MSSA Treatment

Ideally, allocated antibiotics will be continued for the entire parenteral treatment duration unless they need to be stopped due to a complication, for inefficacy, or due to a participation in another domain. For patients who remain a hospital inpatient, the allocated treatment should be continued for as long as possible with the total duration of therapy as clinically indicated. Where patients go to outpatient antimicrobial therapy (OPAT) / hospital in the home (HITH) programs the allocated antibiotic should continue while in OPAT/HITH. However, it is recognized that OPAT/HITH IV therapy may require a switch for cost/convenience issues.

The minimum protocol duration of allocated study treatment is 14 days for those not allocated to early oral switch, and 5 (uncomplicated) or 12 days (complicated) for those allocated to early oral switch. A clinician may choose to change the backbone agent earlier than these time points, for perceived toxicity, lack of efficacy, or economic reasons and the patient will still continue in the study and be analysed in the group to which they were originally allocated. However, such changes will be

discouraged, and site PIs will be asked to endeavour to complete the protocol-directed treatment durations wherever possible.

8.4. Concomitant care

All enrolled patients will receive the highest-quality guideline concordant care as described in the Core Protocol Section 6.7.4.

8.5. Endpoints

8.5.1. Primary endpoint

The primary endpoint for this domain is the platform primary endpoint (all-cause mortality at 90 days after platform entry) as specified in Core Protocol Section 6.8.

8.5.2. Secondary outcomes

All secondary platform endpoints as specified in the Core Protocol Section 6.8.

The domain-specific secondary endpoints will be:

1. Acute kidney injury defined using modified Kidney Disease Improving Global Outcomes (KDIGO) criteria.

   The KDIGO guidelines for acute kidney injury (AKI) define AKI as:

   - Increase in serum creatinine by 0.3mg/dL (= 26.5 µmol/L) or more within 48 hours OR
   - Increase in serum creatinine to 1.5 times baseline or more within the last 7 days OR
   - Urine output less than 0.5 mL/kg/h for 6 hours

   For the purposes of SNAP, a modified KDIGO definition will be used:

   - Increase in serum creatinine by 0.3mg/dL (= 26.5 µmol/L) or more at any time from platform entry (baseline) to day 5 OR
   - Increase in serum creatinine by 1.5 times or more the level at platform entry (baseline) within 14 days of platform entry.

   As a pragmatic trial, data collected on serum creatinine will be mandated at platform entry (platform day 1 or the calendar day prior to platform entry) and days 5±1 and 14±3. Logistical complexities will make it difficult to consistently collect data for urine output.

2. Renal replacement therapy at any stage up to platform day 90.
3. Ongoing renal replacement therapy at platform day 90.
4. Hepatotoxicity - Grade 2 or above increase in ALT and/or GGT (>2.5x ULN), which was not present at platform entry, within 14 days after platform entry.
5. Change in assigned backbone antibiotic therapy during the total index hospitalisation, starting from reveal of allocated domain intervention, due to an adverse event deemed by the treating doctor/team to be of sufficient severity to change therapy.
6. Change in assigned backbone antibiotic therapy during the total index hospitalisation, starting from reveal of allocated domain intervention, due to presumed lack of efficacy according to the treating doctor/team
7. Peripherally inserted central catheter (PICC)/other central venous catheter complications requiring line removal, during the total index hospitalisation, starting from reveal of allocated domain intervention.
    a. This outcome will be collected at total index hospital discharge as a Y/N question. It will include any of the following: catheter-related blood stream infection; exit site infection; catheter-related superficial or deep venous thrombosis/thrombophlebitis; catheter blockage. It will NOT include PICC line rupture, leakage, displacement, or splitting unless it results in or occurs in addition to one of the above events.

Note that “total index hospitalisation” includes Initial hospital admission to an acute inpatient facility, including HITH/OPAT and stepdown inpatient rehabilitation/post-acute care (if continuous with the initial inpatient admission)

9. TRIAL CONDUCT

9.1. Domain-specific data collection

9.1.1. Clinical data and sample collection

Additional data collection will include:

1. Administration of backbone therapies during the total index hospitalisation, starting from platform entry.
2. Creatinine values at platform day 5 (+/- 1 day), and platform day 14 (+/-3 days). We will also record the highest serum creatinine value during the total index hospitalisation and the final value during the total index hospitalisation (starting from platform entry).

3. ALT and/or GGT at platform day 5 (+/- 1 day), and platform day 14 (+/-3 days). We will record the highest ALT and/or GGT values during the total index hospitalisation and the final value during the total index hospitalisation (starting from domain entry, and considering all measurements, whether protocol defined or clinician initiated).
4. Change in allocated therapy and reasons for this (collected at platform day 7, 14, and acute and total index hospital discharge)
5. PICC line or other central venous catheter associated complications occurring during the total index hospitalisation, starting from domain entry.

9.1.2. Domain-specific study timeline

Table 1: Domain-specific schedule of visits and follow-up

Platform Day	Day 1-3	Day 2 until d/c	Day 5 (+/-1 day)	Day 14 (+/-3 days)
Check domain eligibility (confirmation of susceptibility results)	X			
Administration & dosing of allocated study treatment	X[a]	X[b]		
Creatinine measurement[c]			X	X[d]
ALT and/or GGT measurement[c]			X	X[d]

[a] As soon as domain eligibility confirmed.
[b] See section 8.3.5 for duration of allocated backbone therapy
[c] Note that creatinine and ALT/GGT are being measured at platform entry as part of the core protocol
[d] Measuring serum creatinine, ALT and/or GGT on day 14+/3 days is only mandated during the total index hospital stay. If the patient has been discharged, it could still be collected as part of routine follow up if clinically indicated, but this is not protocol mandated.

9.1.3. Domain-specific study visit day details

All core study visit details are specified in the Core Protocol (Section 8.8). Data will be collected, as per the CRFs, on platform day 1, day 8-10 (for data from platform days 1-7), day 15-18 (for data from platform days 8-14), day 28, day 42, day 90, and acute and total discharge.

Additional domain-specific study procedures are outlined below:

9.1.3.1. Screening

In addition to the screening procedures outlined in the Core Protocol (Section 8.8), additional domain-specific screening procedures will occur as per the eligibility criteria outlined in Section 8.2.

9.1.3.2. Day 1-3

In addition to the activities outlined in the Core Protocol (Section 8.8) additional domain-specific activities will be conducted, including:

- Confirmation of susceptibility result to ensure participant is in the correct silo
- Confirmation of domain-susceptibility eligibility criteria
- Initiation or continuation of study allocated treatment(s)

9.1.3.3. Day 2 till hospital discharge

Core activities on Day 2 until total index hospitalisation discharge are outlined in the Core Protocol (Section 8.8). In addition to this, domain-specific activities will be performed on day 2 until the participant is discharged from total index hospitalisation, including:

- Administration of study allocated treatment(s)

9.1.3.4. Day 5 (+/- 1 day)

In addition to the activities outlined in the Core Protocol (Section 8.8) additional domain-specific activities will be conducted on day 5:

- Measurement and recording of creatinine, ALT and/or GGT concentrations
- Recording of any change in allocated therapy, and reason why (see secondary outcomes 8.5.2)

9.1.3.5. Day 14 (+/- 3 days)

In addition to the activities outlined in the Core Protocol (Section 8.8) additional domain-specific activities will be conducted on day 5:

- Measurement and recording of creatinine, ALT and/or GGT concentrations
- Recording of any change in allocated therapy, and reason why (see secondary outcomes 8.5.2)

9.1.3.6. Total Index Hospital Discharge

In addition to the activities outlined in the Core Protocol (Section 8.8) additional domain-specific activities will be conducted on the day of total index hospital discharge, including:

- Record peak and latest creatinine, ALT and/or GGT concentrations taken during total index admission, starting from platform entry, including non-day 5 and 14 measurements obtained by the clinical team
- Record any change in allocated therapy, and reason why (see secondary outcomes 8.5.2)

- Record any PICC line or other central venous catheter-related events requiring line removal that occurred during index hospitalisation, starting from platform entry.

9.1.3.7. Day 90

In addition to the activities outlined in the Core Protocol (Section 8.8) additional domain-specific activities will be conducted on day 90, including:

- Record any new or ongoing requirement for renal replacement therapy during the first 90 days post platform entry

9.2. Criteria for discontinuation

Refer to Core Protocol Section 8.10 for criteria for discontinuation of participation in the SNAP trial.

9.3. Blinding

9.3.1. Blinding

The study drugs are used open label and therefore blinding is not relevant on a per patient basis. On a study-wide basis, investigators, site and study personnel will remain blinded to pooled domain outcomes and summaries until the DSMC has recommended terminating the cell or domain for non-inferiority, superiority or futility.

9.3.2. Unblinding

Not relevant.

10. STATISTICAL CONSIDERATIONS

10.1. Estimands, endpoints, and intercurrent events

10.1.1. Primary estimand

The primary estimand, endpoint, and intercurrent events strategy for this domain is the core SNAP primary endpoint (i.e. all-cause mortality 90 days after platform entry) as specified in Statistical Analysis Appendix.

10.1.2. Secondary estimands

All core secondary estimands, endpoints, and intercurrent events strategies are specified in the Statistical Analysis Appendix.

The domain-specific secondary estimands, endpoints, and intercurrent events are defined as follows:

Estimand/Objective/Target population	Endpoint/Population-level summaries	Intercurrent events strategy
**Estimand A1.1**<br>To evaluate, within each relevant cell, the effect of the intervention compared to the domain control, on the probability of **all-cause mortality at platform 90** in platform eligible participants who adhere to treatment.	<u>Endpoint</u>: All-cause mortality at 90 days after platform entry#.<br><u>Population summary</u>: Log-odds ratio of the stated event between intervention and control groups within each relevant cell.	Principal stratum policy (per protocol principle), see Section 10.7.
**Estimand A1.2**<br>To evaluate, within each relevant cell, the effect of revealed randomised intervention compared to the domain control, on the probability of **acute kidney injury defined using modified KDIGO criteria at platform day 14**, in platform eligible participants	<u>Endpoint</u>: An increase in serum creatinine by ≥0.3 mg/dl (≥26.5 µmol/l) compared to platform entry (baseline), during the first 14 days from platform entry OR an increase in serum creatinine by ≥1.5 times or more the level at platform entry (baseline) at any time in the first 14 days from platform entry (NB: urine volume <0.5 ml/kg/hour for 6 hours is not included in definition due to inadequate charting in most hospitals).<br>As a pragmatic trial, data collected on serum creatinine will be mandated at baseline (platform day 1 or the calendar day prior) and days 5±1 and 14±3. Logistical complexities will make it difficult to consistently collect data for urine output.<br><u>Population summary</u>: As for estimand A1.1.	Treatment policy strategy (intent-to-treat principle)
**Estimand A1.3**<br>To evaluate, within each relevant cell, the effect of revealed randomised intervention compared to the domain control, on probability of a **new requirement for renal replacement therapy (excluding**	<u>Endpoint</u>: Receipt of renal replacement therapy up to 90 days following platform entry.<br><u>Population summary</u>: As for estimand A1.1.	Treatment policy strategy (intent-to-treat principle)

**prophylaxis) in the 90 days following platform entry**, in platform eligible participants		
**Estimand A1.4**<br>To evaluate, within each relevant cell, the effect of revealed randomised intervention compared to the domain control, on probability of a**n ongoing requirement for renal replacement therapy (excluding prophylaxis) at platform day 90**, in platform eligible participants without end-stage kidney disease at platform entry.	Endpoint: Clinician-indicated need for ongoing renal replacement therapy at platform day 90.<br>Population summary: As for estimand A1.1.	Treatment policy strategy (intent-to-treat principle)
**Estimand A1.5**<br>To evaluate, within each relevant cell, the effect of revealed randomised intervention compared to the domain control, on the probability of **hepatoxicity occurring at any time after platform entry up until platform day 14**, in platform eligible participants	Endpoint: Grade 2 or above increase in ALT OR ALP/GGT (>3x ULN) occurring at any time after platform entry up until platform day 14<br>Population summary: As for estimand A1.1.	Treatment policy strategy (intent-to-treat principle)
**Estimand A1.6**<br>To evaluate, within each relevant cell, the effect of revealed randomised intervention compared to the domain control, on the probability of a **change in backbone antibiotic therapy due to an adverse event deemed by the treating doctor/team to be of sufficient severity to change therapy during the total index hospitalisation following platform entry**, in platform eligible participants.	Endpoint: Clinician-indicated change in backbone antibiotic therapy due to adverse events or toxicity during the total index hospitalisation following platform entry.<br>Population summary: As for estimand A1.1.	Treatment policy strategy (intent-to-treat principle)
**Estimand A1.7**<br>To evaluate, within each relevant cell, the effect of revealed randomised intervention compared to the domain control, on the probability of a **change in assigned backbone antibiotic therapy due to presumed lack of efficacy according to the treating doctor/team during the total index hospitalisation following platform entry**, in platform eligible participants	Endpoint: Clinician-indicated change in allocated backbone antibiotic (domain) therapy due to lack of efficacy or toxicity during the total index hospitalisation following platform entry.<br>Population summary: As for estimand A1.1.	Treatment policy strategy (intent-to-treat principle)
**Estimand A1.8**<br>To evaluate, within each relevant cell, the effect of revealed	Endpoint: 7.Peripherally inserted central catheter (PICC)/other central venous catheter complications requiring line	Treatment policy strategy (intent-to-treat principle)

randomised intervention compared to the domain control, on probability of Peripherally inserted central catheter (PICC)/other central venous catheter complications requiring line removal **during the total index hospitalization,** starting from reveal of allocated domain intervention, in platform eligible participants.	removal **during the total index hospitalization,** starting from reveal of allocated domain intervention,<br><br>Population summary: As for estimand A1.1.	

Platform entry is defined as the date that consent was obtained.

10.2. Statistical modelling

10.2.1. Primary model

The population summary (log-odds ratio) for the binary primary endpoint (all-cause mortality at 90 days) will be modelled using a Bayesian binomial model with a logit link. See the Statistical Analysis Appendix.

10.2.2. Secondary models

The population summaries for all core secondary endpoints as specified in the Core Protocol (Section 6.8.2) will be modelled as specified in the Statistical Analysis Appendix.

The domain-specific endpoints described in Section 10.1 are all binary and have log-odds population summaries that will be modelled using a Bayesian binomial model with a logit link as specified in the Statistical Analysis Appendix.

10.3. Decision criteria

Stopping decisions in each cell in this domain are based on whether the posterior probabilities that the intervention is **non-inferior** or **superior,** with respect to the relevant control, is above, or below, pre-specified thresholds. Where a cell stopping decision is recommended because a posterior probability is below the pre-specified threshold, we say that it is futile to continue with the objective of demonstrating non-inferiority or superiority, whichever is relevant.

The primary objective for this domain is to determine if penicillin is **non-inferior** to (flu)cloxacillin (for the PSSA silo) and if cefazolin is **non-inferior** to (flu)cloxacillin (for the MSSA silo). Non-inferiority is

defined as OR < 1.2 for the primary endpoint (where OR > 1 indicates an increase in mortality for penicillin in the PSSA silo or for cefazolin in the MSSA silo). Within each cell, if non-inferiority is demonstrated at a pre-specified interim analysis, which is defined as the posterior probability of non-inferiority in that cell of greater than 99%, recruitment into the cell may continue to seek a conclusion of superiority, based on a recommendation from the DSMC and TSC. A cell stopping decision will be made for futility of the non-inferiority objective if, at a pre-specified interim analysis, the posterior probability of non-inferiority in that cell is less than 1%.

Superiority is defined as an OR < 1 for the primary endpoint. Within each cell, a stopping decision for superiority will be made if, at a pre-specified interim analysis, the posterior probability of superiority in that cell is greater than 99%. A cell stopping decision for futility of the superiority objective will be made if, at a pre-specified interim analysis, the posterior probability of OR < 1/1.2 for the primary endpoint in that cell is less than 1%.

If, at any interim analysis, the thresholds for the decision criteria are not met within a cell, then recruitment into the cell will continue. In all other respects the decision criteria for this appendix are those outlined in the Core Protocol (Sections 9.12) and the Statistical Appendix.

10.4. Randomization

Participants will be randomised at platform entry in a fixed 1:1 ratio within each of the PSSA and MSSA cells. A patient's allocated intervention will be revealed at the time that susceptibility results to define MSSA or PSSA are available and domain-specific eligibility criteria are confirmed. Response adaptive randomization may be applied if additional interventions within this domain are included in future versions of this DSA.

10.5. Interactions with interventions in other domains

No interactions are considered likely between interventions in this domain, and those in any other domain

10.6. Pre-specified secondary analyses

Pre-specified secondary analysis on the primary estimand will be performed by modifying the primary statistical model to include the following covariates and their treatment interactions:

1) Endocarditis
    a. Left-sided endocarditis
    b. Right-sided endocarditis

c. No endocarditis

2) For PSSA silo - presence of *blaZ* and absence of *blaZ*
    a. Despite the testing protocols, some cases of *blaZ* positive strains might be misclassified into the PSSA silo. Determining whether this misclassification is clinically relevant will be important to study generalizability. *blaZ* presence will only be determined retrospectively in a central laboratory.
3) For MSSA silo - presence of cefazolin inoculum effect (CIE) and absence of CIE
    a. The CIE may only be present for a sub-group of isolates. If the CIE is clinically relevant, we would hypothesise that: 1) cefazolin is more likely to be non-inferior to anti-staphylococcal penicillins where the inoculum effect is absent; 2) cefazolin is less likely to be non-inferior to anti-staphylococcal penicillins where inoculum effect is present
    b. CIE will be determined in a reference laboratory and can be assessed in the manner decided by the microbiology working group based on a full and updated review of evidence at the time of the analysis.
4) For MSSA silo - presence of (flu)cloxacillin inoculum effect (FIE) and absence of FIE
    a. the FIE may only be present for a sub-group of isolates. If the FIE is clinically relevant, we would hypothesise that: 1) cefazolin is more likely to be non-inferior to anti-staphylococcal penicillins where the inoculum effect is present; 2) cefazolin is less likely to be non-inferior to anti-staphylococcal penicillins where inoculum effect is absent
    b. FIE will be determined in a reference laboratory and can be assessed in the manner decided by the microbiology working group based on a full and updated review of evidence at the time of the analysis.
5) For MSSA silo - presence of type A beta-lactamase versus its absence
    a. For same reasons as CIE, but using genotype instead of phenotype
6) For MSSA silo – focus of infection includes central nervous system versus not
    a. Central nervous system infection includes brain abscess, subdural empyema, ventriculitis, septic myelitis, and infected prosthetic material within the CNS (ventriculoperitoneal shunts, lumbar shunts, ventricular reservoirs, external ventricular drains, and deep brain or spinal stimulators). It does not include epidural abscess or vertebral/skull osteomyelitis in the absence of the above.
    b. Cefazolin is thought to have poor CNS penetration and is thus recommended to be avoided in CNS infections by some clinical guidelines. However, there is no strong clinical evidence to support this fear, and some PK data suggests that adequate

concentrations can be reached in CSF. Thus, if this concern is real, then the difference in the primary outcome between cefazolin and (flu)cloxacillin will be different (worse outcomes with cefazolin) in this subgroup compared with all other patients in the cell.

10.7. Principal stratum policy

The principal stratum policy (also known as a per protocol principle) for estimand A1.1 uses the target population as described in the following subsections.

10.7.1. PSSA silo

10.7.1.1. If allocated to penicillin

Received at least 12 days (full or part) of benzylpenicillin if not allocated to early oral switch at day 7, and received at least 5 days (full or part) of benzylpenicillin if allocated to early oral switch at day 7, AND received no more than one dose of (flu)cloxacillin between the time of domain eligibility confirmation and the end of the total index hospitalisation.

10.7.1.2. If allocated to (flu)cloxacillin

Received at least 12 days (full or part) of (flu)cloxacillin if not allocated to early oral switch at day 7 and received at least 5 days (full or part) of (flu)cloxacillin if allocated to early oral switch at day 7, AND received no more than one dose of benzylpenicillin between the time of domain eligibility confirmation and the end of the total index hospitalisation.

10.7.2. MSSA silo

10.7.2.1. If allocated to cefazolin

Received at least 12 days (full or part) of cefazolin if not allocated to early oral switch at day 7, and received at least 5 days (full or part) of cefazolin if allocated to early oral switch at day 7, AND received no more than one dose of (flu)cloxacillin between the time of domain eligibility confirmation and the end of the total index hospitalisation.

10.7.2.2. If allocated to (flu)cloxacillin

Received at least 12 days (full or part) of (flu)cloxacillin if not allocated to early oral switch at day 7, and received at least 5 days (full or part) of (flu)cloxacillin if allocated to early oral switch at day 7, AND received no more than one dose of cefazolin between the time of domain eligibility confirmation and the end of the total index hospitalisation.

11. ETHICAL CONSIDERATIONS

11.1. Data Safety and Monitoring Committee

The DSMC should be aware of the pre-specified decision criteria for superiority, inferiority, or non-inferiority of different interventions compared to the domain control and with respect to the primary endpoint. If non-inferiority is demonstrated, determination of the optimal intervention may involve secondary endpoints, such as changing therapy due to adverse drug events, renal failure, *C. difficile*, thrombophlebitis or PICC-line associated complications.

11.2. Potential domain-specific adverse events

The following possible treatment-related adverse events should be reported in all patients in this domain, irrespective of intervention allocation.

- See section 8.5.2

Other SAEs should be reported only where, in the opinion of the site investigator, the event might reasonably have occurred as a consequence of a study intervention (i.e. if it is a serious adverse reaction, see Core protocol Section 10).

11.3. Domain-specific consent issues

Consent for this domain will be sought at platform entry and will not require re-confirmation or repeat eligibility assessment at the time susceptibility is confirmed.

12. GOVERNANCE ISSUES

12.1. Funding of domain

Funding sources for the SNAP trial are specified in the Core Protocol Section 2.5. This domain has not received any additional domain-specific funding.

12.2. Funding of domain interventions and outcome measures

Interventions are considered standard of care and will be covered by hospital operating budgets. Outcome measures are pragmatic and do not deviate from routine testing performed during usual care for SAB.

12.3. Domain-specific declarations of interest

All investigators involved in SNAP maintain a registry of interests on the SNAP website. These are updated periodically and publicly accessible on the study website

13.REFERENCES

1. Butler-Laporte G, Lee TC, Cheng MP. Increasing Rates of Penicillin Sensitivity in Staphylococcus aureus. Antimicrob Agents Chemother. 2018;62(7).

2. Cheng MP, Rene P, Cheng AP, Lee TC. Back to the Future: Penicillin-Susceptible Staphylococcus aureus. Am J Med. 2016;129(12):1331-3.

3. Hagstrand Aldman M, Skovby A, L IP. Penicillin-susceptible Staphylococcus aureus: susceptibility testing, resistance rates and outcome of infection. Infect Dis (Lond). 2017;49(6):454-60.

4. Kanjilal S, Sater MRA, Thayer M, Lagoudas GK, Kim S, Blainey PC, et al. Trends in antibiotic susceptibility in Staphylococcus aureus in Boston, Massachusetts, 2000-2014. J Clin Microbiol. 2017.

5. Tong SY, Davis JS, Eichenberger E, Holland TL, Fowler VG, Jr. Staphylococcus aureus infections: epidemiology, pathophysiology, clinical manifestations, and management. Clin Microbiol Rev. 2015;28(3):603-61.

6. Baddour LM, Wilson WR, Bayer AS, Fowler VG, Jr., Tleyjeh IM, Rybak MJ, et al. Infective Endocarditis in Adults: Diagnosis, Antimicrobial Therapy, and Management of Complications: A Scientific Statement for Healthcare Professionals From the American Heart Association. Circulation. 2015;132(15):1435-86.

7. Pitkala A, Salmikivi L, Bredbacka P, Myllyniemi AL, Koskinen MT. Comparison of tests for detection of beta-lactamase-producing staphylococci. J Clin Microbiol. 2007;45(6):2031-3.

8. Henderson AW, D.; Francis, .F; Engler, C.; Glover, M.; Douglas, J.; Gore, L.; Harris, P.; Paterson, D.; Nimmo, G.; Norton, R. Prospective evaluation of beta-lactamase detection in penicillin susceptible Staphylococcus aureus by interpretation of the penicillin disc edge. bioRxiv. 2020.

9. Yok-Ai Q, Moreillon P. Staphylococcus aureus (Including Staphylococcal Toxic Shock Syndrome). Philadelphia, PA: Saunders; 2015.

10. Weis S, Kesselmeier M, Davis JS, Morris AM, Lee S, Scherag A, et al. Cefazolin versus anti-staphylococcal penicillins for the treatment of patients with Staphylococcus aureus bacteraemia. Clin Microbiol Infect. 2019;25(7):818-27.

11. Henderson A, Harris P, Hartel G, Paterson D, Turnidge J, Davis JS, et al. Benzylpenicillin versus flucloxacillin for penicillin-susceptible Staphylococcus aureus bloodstream infections from a large retrospective cohort study. Int J Antimicrob Agents. 2019.

12. Crane JK. Resurgence of penicillin-susceptible Staphylococcus aureus at a hospital in New York State, USA. J Antimicrob Chemother. 2014;69(1):280-1.

13. Tong SYC, Campbell A, Bowen AC, Davis JS. A survey of infectious diseases and microbiology clinicians in Australia and New Zealand about the management of Staphylococcus aureus bacteraemia. Clin Infect Dis. 2019.

14. Lam JC, Gregson DB, Robinson S, Somayaji R, Conly JM, Parkins MD. Epidemiology and Outcome Determinants of Staphylococcus aureus Bacteremia Revisited: A Population-Based Study. Infection. 2019.

15. Laupland KB, Church DL. Population-based epidemiology and microbiology of community-onset bloodstream infections. Clin Microbiol Rev. 2014;27(4):647-64.

16. Laupland KB, Ross T, Gregson DB. Staphylococcus aureus bloodstream infections: risk factors, outcomes, and the influence of methicillin resistance in Calgary, Canada, 2000-2006. J Infect Dis. 2008;198(3):336-43.

17. Habib G, Lancellotti P, Antunes MJ, Bongiorni MG, Casalta JP, Del Zotti F, et al. 2015 ESC Guidelines for the management of infective endocarditis: The Task Force for the Management of Infective Endocarditis of the European Society of Cardiology (ESC). Endorsed by: European Association for Cardio-Thoracic Surgery (EACTS), the European Association of Nuclear Medicine (EANM). Eur Heart J. 2015;36(44):3075-128.

18. Lee S, Kwon KT, Kim HI, Chang HH, Lee JM, Choe PG, et al. Clinical implications of cefazolin inoculum effect and beta-lactamase type on methicillin-susceptible Staphylococcus aureus bacteremia. Microb Drug Resist. 2014;20(6):568-74.

19. Miller WR, Seas C, Carvajal LP, Diaz L, Echeverri AM, Ferro C, et al. The Cefazolin Inoculum Effect Is Associated With Increased Mortality in Methicillin-Susceptible Staphylococcus aureus Bacteremia. Open Forum Infect Dis. 2018;5(6):ofy123.

20. Sabath LD, Garner C, Wilcox C, Finland M. Effect of inoculum and of beta-lactamase on the anti-staphylococcal activity of thirteen penicillins and cephalosporins. Antimicrobial agents and chemotherapy. 1975;8(3):344-9.

21. Nannini EC, Stryjewski ME, Singh KV, Bourgogne A, Rude TH, Corey GR, et al. Inoculum effect with cefazolin among clinical isolates of methicillin-susceptible Staphylococcus aureus: frequency and possible cause of cefazolin treatment failure. Antimicrobial agents and chemotherapy. 2009;53(8):3437-41.

22. Bryant RE, Alford RH. Unsuccessful treatment of staphylococcal endocarditis with cefazolin. JAMA : the journal of the American Medical Association. 1977;237(6):569-70.

23. Bai AD, Showler A, Burry L, Steinberg M, Ricciuto DR, Fernandes T, et al. Comparative effectiveness of cefazolin versus cloxacillin as definitive antibiotic therapy for MSSA bacteraemia: results from a large multicentre cohort study. The Journal of antimicrobial chemotherapy. 2015;70(5):1539-46.

24. Lee S, Choe PG, Song KH, Park SW, Kim HB, Kim NJ, et al. Is cefazolin inferior to nafcillin for treatment of methicillin-susceptible Staphylococcus aureus bacteremia? Antimicrobial agents and chemotherapy. 2011;55(11):5122-6.

25. Li J, Echevarria KL, Hughes DW, Cadena JA, Bowling JE, Lewis JS, 2nd. Comparison of cefazolin versus oxacillin for treatment of complicated bacteremia caused by methicillin-susceptible Staphylococcus aureus. Antimicrobial agents and chemotherapy. 2014;58(9):5117-24.

26. McDanel JS, Roghmann MC, Perencevich EN, Ohl ME, Goto M, Livorsi DJ, et al. Comparative Effectiveness of Cefazolin Versus Nafcillin or Oxacillin for Treatment of Methicillin-Susceptible Staphylococcus aureus Infections Complicated by Bacteremia: A Nationwide Cohort Study. Clinical infectious diseases : an official publication of the Infectious Diseases Society of America. 2017;65(1):100-6.

27. Rao SN, Rhodes NJ, Lee BJ, Scheetz MH, Hanson AP, Segreti J, et al. Treatment outcomes with cefazolin versus oxacillin for deep-seated methicillin-susceptible Staphylococcus aureus bloodstream infections. Antimicrobial agents and chemotherapy. 2015;59(9):5232-8.

28. Pollett S, Baxi SM, Rutherford GW, Doernberg SB, Bacchetti P, Chambers HF. Cefazolin versus Nafcillin for Methicillin-Sensitive Staphylococcus aureus Bloodstream Infection in a California Tertiary Medical Center. Antimicrobial agents and chemotherapy. 2016;60(8):4684-9.

29. Flynt LK, Kenney RM, Zervos MJ, Davis SL. The Safety and Economic Impact of Cefazolin versus Nafcillin for the Treatment of Methicillin-Susceptible Staphylococcus aureus Bloodstream Infections. Infect Dis Ther. 2017;6(2):225-31.

30. Youngster I, Shenoy ES, Hooper DC, Nelson SB. Comparative evaluation of the tolerability of cefazolin and nafcillin for treatment of methicillin-susceptible Staphylococcus aureus infections in the outpatient setting. Clinical infectious diseases : an official publication of the Infectious Diseases Society of America. 2014;59(3):369-75.

31. Kyriacou DN, Lewis RJ. Confounding by Indication in Clinical Research. JAMA. 2016;316(17):1818-9.

32. Echt DS, Liebson PR, Mitchell LB, Peters RW, Obias-Manno D, Barker AH, et al. Mortality and morbidity in patients receiving encainide, flecainide, or placebo. The Cardiac Arrhythmia Suppression Trial. N Engl J Med. 1991;324(12):781-8.

33. Hebert PC, Wells G, Blajchman MA, Marshall J, Martin C, Pagliarello G, et al. A multicenter, randomized, controlled clinical trial of transfusion requirements in critical care. Transfusion Requirements in Critical Care Investigators, Canadian Critical Care Trials Group. N Engl J Med. 1999;340(6):409-17.

34. Herrera-Perez D, Haslam A, Crain T, Gill J, Livingston C, Kaestner V, et al. A comprehensive review of randomized clinical trials in three medical journals reveals 396 medical reversals. Elife. 2019;8.

35. Hulley S, Grady D, Bush T, Furberg C, Herrington D, Riggs B, et al. Randomized trial of estrogen plus progestin for secondary prevention of coronary heart disease in postmenopausal women. Heart and Estrogen/progestin Replacement Study (HERS) Research Group. JAMA. 1998;280(7):605-13.
